# Sesquiterpene Coumarin Ethers and Phenylpropanoids from the Roots of *Ferula drudeana*, the Putative Anatolian Ecotype of the Silphion Plant †

**DOI:** 10.3390/molecules30091916

**Published:** 2025-04-25

**Authors:** Fadıl Kaan Kuran, Sarath P. D. Senadeera, Dongdong Wang, Ji-Yeon Hwang, Ekaterina Goncharova, Jennifer Wilson, Antony Wamiru, Brice A. P. Wilson, Nathanael Pruett, Lin Du, Chuong D. Hoang, John A. Beutler, Mahmut Miski

**Affiliations:** 1 Department of Pharmacognosy, Faculty of Pharmacy, Istanbul University, Istanbul 34116, Türkiye; kaankuran@istanbul.edu.tr; 2 Department of Pharmacognosy, Institute of Graduate Studies in Health Sciences, Istanbul University, Istanbul 34116, Türkiye; 3 Molecular Targets Program, National Cancer Institute, Frederick, MD 21702, USA; spdsenadeera@gmail.com (S.P.D.S.); dongdong.wang@nih.gov (D.W.); jiyeon.hwang@nih.gov (J.-Y.H.); katya.goncharova@nih.gov (E.G.); wilsonje@mail.nih.gov (J.W.); lin.du@nih.gov (L.D.); 4 Advanced Biomedical Computational Science, Frederick National Laboratory for Cancer Research, Frederick, MD 21702, USA; 5 Thoracic Surgery Branch, National Cancer Institute, Bethesda, MD 20892, USAchuong.hoang@nih.gov (C.D.H.)

**Keywords:** Apiaceae, *Ferula drudeana*, sesquiterpene coumarin ethers, phenylpropanoid, kidney cancer

## Abstract

Four new sesquiterpene coumarin ethers (**1**–**4**) and a new phenylpropanoid compound (**5**) were isolated from a hexane extract of the roots of *Ferula drudeana*, the putative Anatolian ecotype of the silphion plant, in addition to nineteen previously described sesquiterpene coumarins (**6**–**24**) and four known phenylpropanoid derivatives (**25**–**28**). The structures of these compounds were elucidated by extensive spectroscopic analysis and computational studies. The cytotoxic activities of all isolated compounds were evaluated on renal, malignant pleural mesothelioma (MPM) and colon cancer cell lines. While 11 sesquiterpene coumarin derivatives showed strong-to-moderate cytotoxic activity against the UO31 renal cancer cell line, 13 compounds showed strong cytotoxic activity against the MPM cell line, and four sesquiterpene coumarin derivatives displayed moderate cytotoxic activity against the colon cancer cell line.

## 1. Introduction

The genus *Ferula,* with more than 227 species, is the largest genus of the Apiaceae family [1]. Many *Ferula* species have been used as medicinal plants since ancient times. Pedanius Dioscorides mentioned five drugs: Ammoniakon (*F. tingitana* or *F. marmarica*), Chalbane (*F. gummosis*), Narthex (*F. communis*), Sagapenon (*F. persica*), and Silphion, in his *De Materia Medica* [2]. Silphion (a.k.a., silphium) was a well-known medicinal resin mainly obtained from the roots of the silphion plant, most likely a *Ferula* species growing in the Cyrenaic region of Libya ca. 2300 years ago [3]. Pliny the Elder gives details of 39 remedies made with silphion in his “Natural History” [4]. Dioscorides describes the use of silphion to treat intestinal and hormonal disorders, goiter, sciatica, epilepsy, tetanus, polyps, and malignant tumors [2]. However, silphion disappeared from commerce about the time of the Roman emperor Nero, after 500 years of robust trade around the Mediterranean [5].

**Figure 1:**
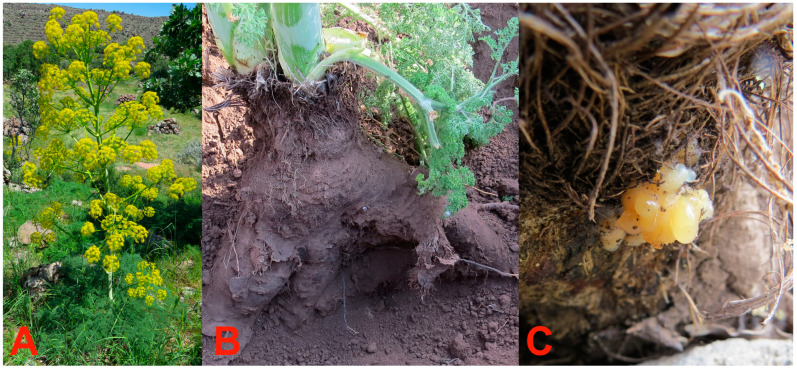
(**A**) The flowering stem of *Ferula drudeana*, (**B**) highly branched rhizomes of *F. drudeana*, (**C**) resin formation by solidification of the latex oozed from a small incision made at the top of the rhizome.

*Ferula drudeana* Korovin, a rare and endemic species growing near former Greek villages in the Cappadocia region of Anatolia, was proposed as an Anatolian ecotype of the silphion plant due to its unusual morphological features and distinct organoleptic characteristics of its resin [6]. Similar to the descriptions of Theophrastus [7] and Pliny the Elder [4] for the silphion plant, *F. drudeana* has highly branched, thick roots that produce resin with a distinct aroma when an incision is made at the top of the root (Figure 1). Although it is possible to collect resin from the roots of *F. drudeana*, due to the scarce availability of live *F. drudeana* plants, we concluded that large-scale resin collection would endanger the survival of the species. The total amount of the resin collected from a limited number of plants would not be enough to investigate its secondary metabolites and their biological activity thoroughly. Therefore, hexane, dichloromethane, and methanol extracts of the roots of *F. drudeana* were prepared, and their secondary metabolite profile was compared with the resin. Since the secondary metabolite profile of the hexane extract of the roots of *F. drudeana* was almost identical to that of the resin (Supplementary Materials Figure S168), compounds were isolated from this extract and investigated for their biological activities.

Here, we report the isolation and structure elucidation of the sesquiterpene coumarins and phenylpropanoids from the hexane extract of *Ferula drudeana* that may act as the active cytotoxic principles of silphion resin for the treatment of malignant tumors mentioned by Dioscorides [2].

## 2. Results

### 2.1. Characterization of Cytotoxic Compounds

**Figure 2:**
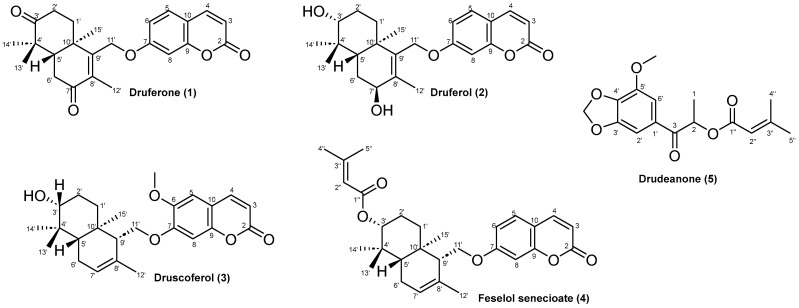
Structures of new compounds isolated from the roots of *F. drudeana*.

The sesquiterpene coumarin derivatives and phenylpropanoids isolated from *F. drudeana* (Figure 2) were characterized through detailed spectroscopic and spectrometric analyses, including ^1^H-NMR, ^13^C-NMR, 2D NMR (HSQC, COSY, HMBC, NOESY), HRESIMS, IR, and UV–Vis.

**Figure 3:**
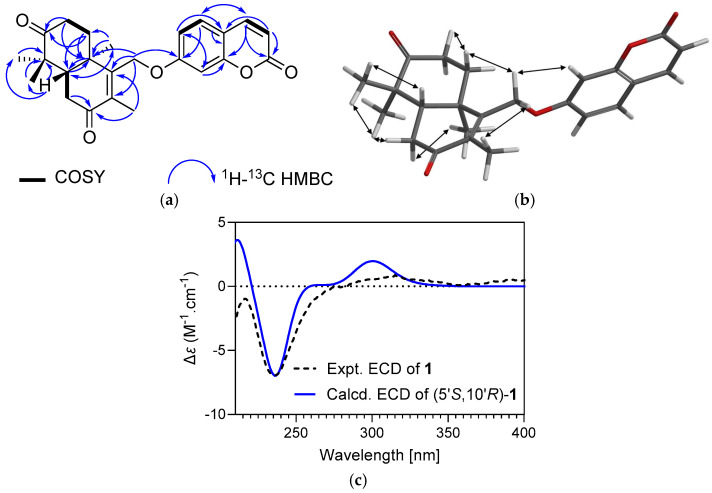
(**a**) COSY and HMBC correlations of **1**; (**b**) NOESY correlations of **1**; (**c**) experimental and calculated ECD spectra of **1**.

Druferone (**1**) was obtained as an amorphous white powder. The (+)-HRESIMS spectrum of **1** showed an [M + H]^+^ molecular ion peak at *m*/*z* 395.1855, indicating a molecular formula of C_24_H_26_O_5_. The ^1^H-NMR spectrum of **1** (Table 1) closely resembled that of the co-occurring ferubungeanol G (**13**) (Supplementary Materials Figures S2 and S60) [8]. A comparison of the ^1^H-NMR spectra between **1** and **13** (Supplementary Materials Figure S86) indicated the absence of the proton at the C3′ position in **13**, accompanied by downfield shifts of some protons previously observed in the upfield region. The ^13^C NMR spectrum of **1** (Supplementary Materials Figure S3) displayed a peak at δ_C_ 214.7, suggesting the presence of an additional carbonyl group compared to **13**. HMBC correlations from the deshielded protons H_2_-2′ at δ_H_ 2.70 and 2.52 to this carbonyl carbon signal (Figure 3a and Supplementary Materials Figure S6) indicated that the oxymethine at C-3′ in **13** was oxidized to a carbonyl group in **1**. NOESY correlations observed for **1** (Figure 3b and Supplementary Materials Figure S7) validated its relative stereochemistry as 5′S*, 10′R*. The absolute configuration of **1** was determined to be 5′*S*, 10′*R* by good agreement of the experimental and calculated ECD spectra (Figure 3c).

**Figure 4:**
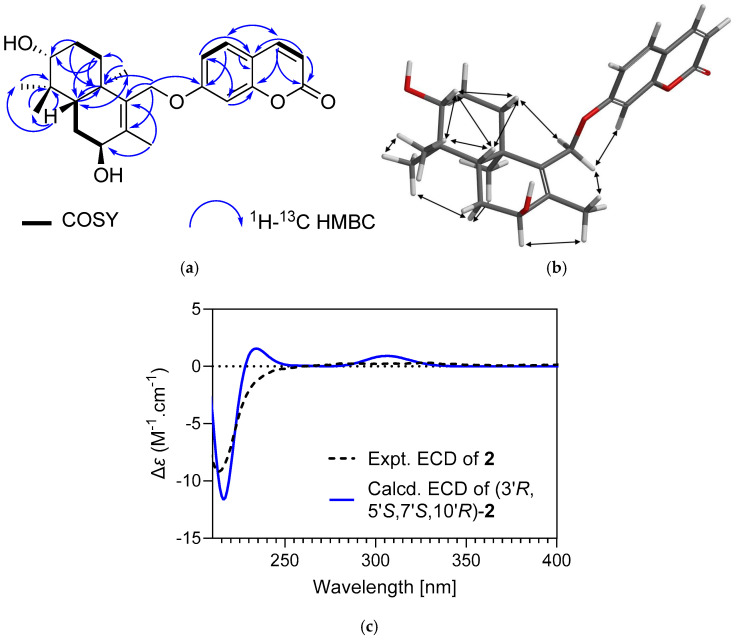
(**a**) Key COSY and HMBC correlations; (**b**) NOESY correlations; (**c**) experimental and calculated ECD spectra of **2**.

Druferol (**2**) was obtained as an amorphous white powder. The (+)-HRESIMS spectrum of **2** showed an [M + H]^+^ ion peak at *m*/*z* 399.2180, confirming a molecular formula of C_24_H_30_O_5_. Compound **2** exhibited close structural similarity to samarcandicin K (**14**) (Supplementary Materials Figures S11 and S62). The sole distinction between the two molecules lies in the stereochemistry of the hydroxyl group at C3′. The ^1^H-NMR spectral comparison between **2** and **14** (Supplementary Materials Figure S87) indicated a minor upfield shift in the resonance of H-3′ in **2**, observed as a doublet of doublets with coupling constants of 11.6 and 4.2 Hz, which corroborated the assignment of H-3′ to a β-axial position. Compound **2** was therefore determined to be the C3′ epimeric form of **14;**. NOESY correlations corroborate the stereochemical assignment illustrated in Figure 4b.

**Figure 5:**
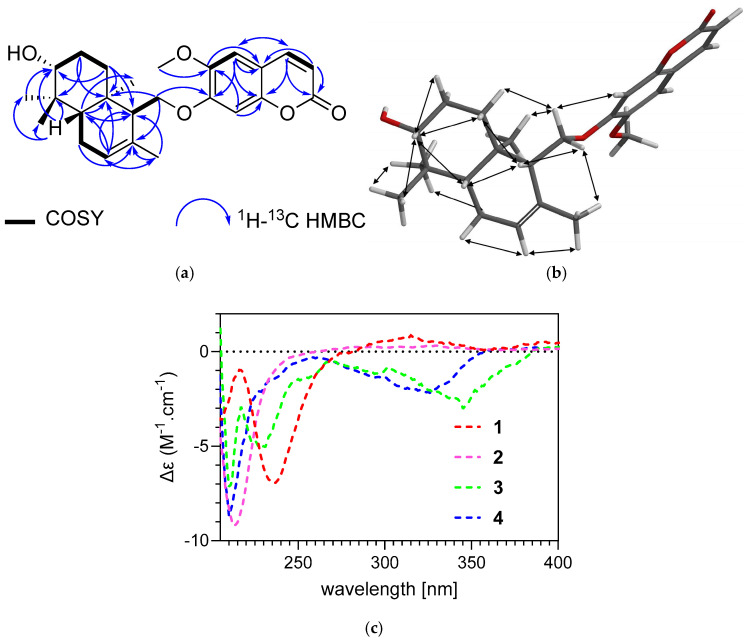
(**a**) Key COSY and HMBC correlations; (**b**) NOESY correlations; (**c**) experimental and calculated ECD spectra of **3** and **4** compared to **1** and **2**.

Druscoferol (**3**) was obtained as an amorphous white powder. The (+)-HRESIMS spectrum of **3** showed an [M + H]^+^ ion peak at *m*/*z* 383.2219, suggesting a molecular formula of C_25_H_32_O_5_ for **3**. The ^1^H-NMR spectrum of **3** (Table 1) showed high similarities to feselol (**11**), except for the absence of a 1,2,4-trisubstituted benzene ring and the presence of a 1,2,4,5-tetrasubstituted benzene ring and an additional methoxyl group (Supplemental Materials Figure S84). Detailed NMR analysis indicated that the H-6 in **11** was replaced by a methoxyl group in **3**. The assignment was supported by HMBC correlations from H-5, H-8, and 6-OCH_3_ to C-6 (Figure 5a and Supplementary Materials Figure S24). The NOESY correlations of **3** (Supplementary Materials Figure S25) provided strong evidence that **3** had the same relative configuration as **11**.

The relative configurations of the drimane sesquiterpenoid skeleton at the C-5′ and C-10′ positions of **3** and **4** as (5′S*,10R*) were consistent with those of **1** and **2** based on the NOESY data. The absolute configurations of **1** and **2** were determined by comparison of their experimental and calculated ECD spectra. The ECD spectra of **3** and **4** showed a strong negative Cotton effect at ~215 nm, in combination with the biogenetic relationship, suggesting the same (5′*S*, 10*R*) absolute configuration of the drimane sesquiterpenoids as that of **1** and **2**. We also relied on our previous assignment of the absolute configuration of samarcandin by crystallography and ECD [8].

**Figure 6:**
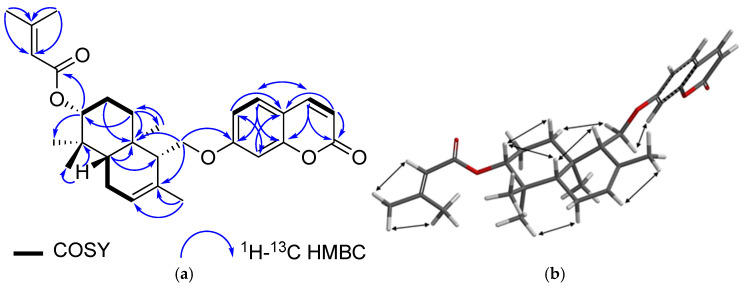
(**a**) Key COSY and HMBC correlations; (**b**) NOESY correlations of **4**.

Feselol senecioate (**4**) was obtained as an amorphous white powder. The (+)-HRESIMS spectrum of **4** showed an [M + Na]^+^ ion peak at *m*/*z* 487.2460, revealing a molecular formula of C_29_H_36_O_5_. The ^1^H-NMR spectrum of **4** (Table 2) exhibited a notable resemblance to that of feselol (**11**), except for the additional resonances for a senecioate substituent on 3α-OH based on crucial HMBC correlations from two broad doublet methyls resonating at δ_H_ 2.17 and δ_H_ 1.89 to the olefinic carbons C-2″ (δ_C_ 116.7) and C-3″ (δ_C_ 156.3) and from H-3′ and H-2″ (δ_H_ 5.68) to the senecioate carbonyl C-1″ (δ_C_ 166.5). The deshielded proton chemical shifts of 3′-methine (δ_H_ 4.57 in **4** vs. δ_H_ 3.27 in **11**) provided additional evidence for the esterification of this position. NOESY confirmed that its stereochemistry is the same as **11**. Accordingly, the feselol senecioate (**4**) structure is proposed, as shown in Figure 6.

The relative configurations of the drimane sesquiterpenoid skeleton at the C-5′ and C-10′ positions of **3** and **4** as (5′S*,10R*) were consistent with those of **1** and **2** based on the NOESY data. The absolute configurations of **1** and **2** were determined by comparison of their experimental and calculated ECD spectra. The ECD spectra of **3** and **4** showed a strong negative Cotton effect at ~215 nm, in combination with the biogenetic relationship, suggesting the same (5′*S*,10*R*) absolute configuration of the drimane sesquiterpenoids as that of **1** and **2**. We also relied on our previous assignment of the absolute configuration of samarcandin [8] by X-ray crystallography and ECD.

As in the case of all drimane type of sesquiterpene coumarins, the sesquiterpene coumarins of *Ferula drudena* were derived from the enzymatic cyclisation of umbelliprenin [or farnesyl ether of the corresponding coumarin derivative, e.g., druscoferol (**3**)] as their common precursor. Careful examination of the relative stereochemistries of sesquiterpene coumarins of *F. drudeana* (Figure 1 and Figure 2) confirm the *cis* orientation of C-15′-methyl, C11′-methylene groups in the drimane skeleton. Since all known sesquiterpene coumarins’ optical rotations were similar to those of published optical rotation data, this observation strongly suggests the absolute configurations of novel sesquiterpene coumarins as depicted in Figure 1, and the ECD data fully support this.

**Figure 7:**
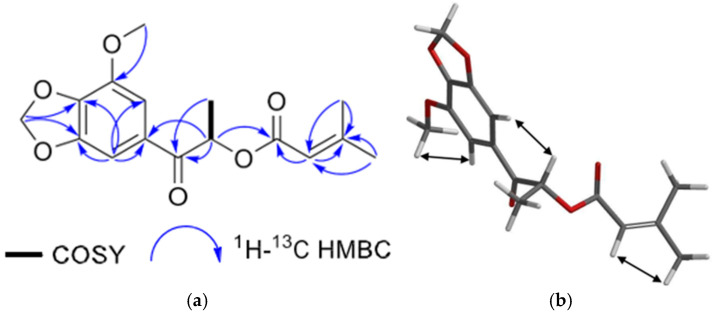
(**a**) Key COSY and HMBC correlations; (**b**) NOESY correlations of (**5**).

Drudeanone (**5**) was obtained as an amorphous white powder. The (+)-HRESIMS spectrum of **5** showed an [M + H]^+^ ion peak at *m*/*z* 307.1182, corresponding to a molecular formula of C_16_H_18_O_6_. The ^1^H-NMR spectrum of **5** (Table 2) resembles that of crocatone (**26**), (Supplementary Materials Figure S88) except for the additional resonances for a senecioic acid substituent on C-2 to form an ester based on crucial HMBC correlations from two broad doublet methys resonating at δ_H_ 2.14 and δ_H_ 1.91 to the olefinic carbons C-2″ (δ_C_ 115.4) and C-3″ (δ_C_ 158.8) and from H-2 (δ_H_ 5.87, q, 7.0, 1H) and H-2′′ (δ_H_ 5.80) to the senecionate carbonyl C-1″ (δ_C_ 165.9). Therefore, the structure of drudeanone (**5**) was determined as shown in Figure 7. Drudeanone did not show any optical rotation; therefore, it must be a racemic mixture of (+) and (−) isomers.

**Figure 8:**
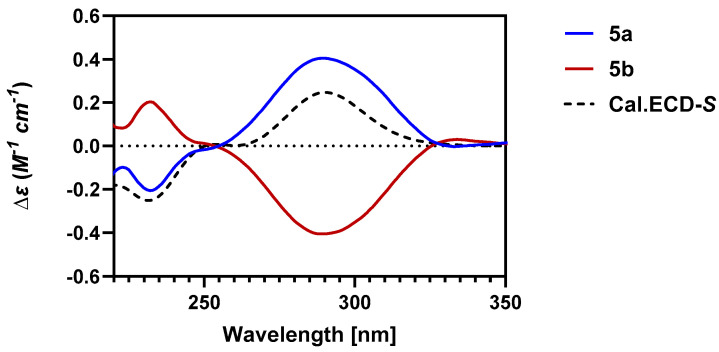
Comparison of the experimental and calculated ECD spectra for **5a** and **5b**.

**Scheme 1:**
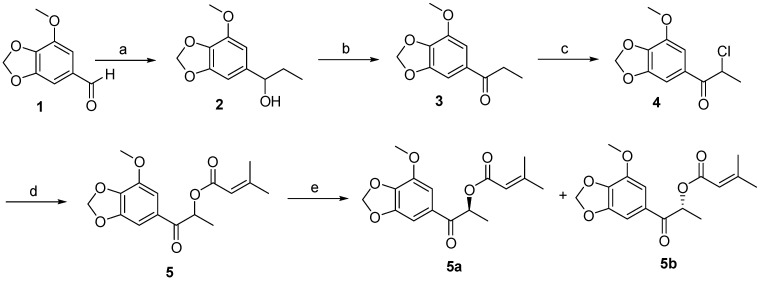
Synthesis and chiral separation of **5a** and **5b**.

We proceeded to synthesize the compound **5** as shown in Scheme 1. Chiral HPLC provided both enantiomers **5a** and **5b**. DFT calculations of ECD (Figure 8) identified the respective absolute configurations, with **5a** being the *S*-enantiomer. These purified compounds showed opposite optical rotations, as would be expected.

Conditions: (a) ethylmagnesium bromide, THF, 0 °C to RT, 4 h; (b) PCC, DCM, RT, 4 h; (c) I_2_, DMSO, 50 °C, 16 h; (d) 3-methylcrotonic acid, Et_3_N, MeCN, 80 °C, 16 h; (e) chiral HPLC, Lux Cellulose-2.

**Table 1:** ^1^H-NMR (600 MHz) and ^13^C-NMR (125 MHz) shifts of druferone (**1**), druferol (**2**), and druscoferol (**3**) (in CDCl_3_, δ in ppm, and *J* in Hz).

Position	Druferone (1)		Druferol (2)		Druscoferol (3)	
	δ_H_, Mult (*J* in Hz)	δ_C_	δ_H_, Mult (*J* in Hz)	δ_C_	δ_H_, Mult (*J* in Hz)	δ_C_
**2**	-	161.1	-	161.6	-	161.8
**3**	6.29 (1H, d; *9.4*)	113.8	6.25 (1H, d, *9.4*)	113.5	6.26 (1H, d, *9.4*)	113.7
**4**	7.64 (1H, d, *9.4*)	143.4	7.64 (1H, d, *9.4*)	143.8	7.60 (1H, d, *9.4*)	143.7
**5**	7.41 (1H, d, *9.2*)	129.1	7.37 1H, d, *9.2*)	129.1	6.84 (1H, s)	109.1
**6**	6.85, m	113.2	6.86 (1H, dd, *9.2*; *2.4*)	113.4	-	147.3
**7**	-	161.5	-	162.9	-	152.8
**8**	6.86, m; 1H	101.5	6.84 (1H, d, *2.4*)	101.7	6.82 (1H, s)	101.3
**9**	-	155.3	-	156.2	-	150.4
**10**	-	113.8	-	112.9	-	111.8
**1′** **α**	2.08, (2H, m)	34.3	1.71 (1H, m)	34.4	2.01 (1H, m)	37.9
**1′** **β**			1.49 (1H, br dd, *14.2, 5.07*)		1.36 (1H, td, *13.0, 4.5*)	
**2′** **α**	2.70 (1H, ddd, *15.8, 11.5, 7.0*)	34.5	1.73 (1H, m)	27.8	1.64 (1H, ddd, *13.0, 3.5, 1.7*)	27.7
**2′** **β**	2.52 (1H, m)		1.63 (1H, m)		1.66 (1H, m)	
**3′**	-	214.7	3.33 (1H, dd, *11.6, 4.2*)	78.9	3.27 (1H, dd, *11.2, 4.4*)	79.2
**4′**	-	47.2	-	39.0	-	39.1
**5′**	2.33 (1H, dd, *13.9, 4.0*)	49.7	1.45 (1H, dd, *12.5, 2.6*)	45.2	1.28 (1H, dd, *11.7, 5.1*)	49.8
**6′α**	2.60 (1H, dd, *17.0, 13.9*)	35.8	1.85–1.79 (2H, m)	28.7	2.02 (2H, m)	23.9
**6′β**	2.55 (1H, t, *4.58*)					
**7′α**	-	199.3	4.08 (1H, br dd, *4.4, 1.7*)	70.4	5.54 (2H, br s)	124.0
**7′β**	-		-			
**8′**	-	135.9	-	135.8	-	132.5
**9′**	-	154.3	-	139.4	2.32 (1H, dd, *6.8*, *3.3*)	53.9
**10′**	-	39.6	-	39.0	-	35.9
**11′α**	4.70 (1H, d, *9.9*)	64.8	4.51 (1H, d, *9.84*)	64.5	3.97 (1H, dd, *9.6, 6.8*)	68.5
**11′β**	4.64 (1H, d, *9.9)*		4.42 (1H, d, *9.84*)		4.18 (1H, dd, *9.6, 3.3*)	
**12′**	1.88 (3H, s)	12.0	1.83 (3H, s)	17.4	1.70 (3H, s)	21.9
**13′**	1.15 (3H, s)	21.4	1.00 (3H, s)	19.3	0.88 (3H, s)	15.1
**14′**	1.14 (3H, s)	26.0	0.84 (3H, s)	15.8	0.89 (3H, s)	15.6
**15′**	1.35 (3H, s)	18.4	1.06 (3H, s)	28.3	0.99 (3H, s)	28.4
**-OCH_3_**	-	-	-	-	3.85 (3H, s)	56.9

**Table 2:** 1H-NMR (600 MHz) and 13C-NMR (125 MHz) shifts of feselol senecioate (**4**) and drudeanone (**5**) (in CDCl3, δ in ppm, and *J* in Hz).

Position	Feselol Senecioate (4)		Drudeanone (5)	
	δ_H_, Mult (*J* in Hz)	δ_C_	δ_H_, Mult (*J* in Hz)	δ_C_
**1**	-	-	1.51 (3H, d, *7.0*)	17.5
**2**	-	161.1	5.87 (1H, q, *7.0*)	70.5
**3**	6.24 (1H, d, *9.4*)	112.9	-	195.4
**4**	7.63 (1H, d, *9.4*)	143.3	-	-
**5**	7.36 (1H, d, *8.5*)	128.6	-	-
**6**	6.82 (1H, dd, *8.5*; *2.4*)	113.0	-	-
**7**	-	161.8	-	-
**8**	6.80 (1H, d, *2.4*)	101.5	-	-
**9**	-	155.8	-	-
**10**	-	112.4	-	-
**1′** **α**	2.02 (1H, m)	37.4	-	140.3
**1′** **β**	1.42 (1H, dd, *13.5, 4.3*)		-	
**2′** **α**	1.72 (2H, m)	34.5	7.15 (1H, d, *1.5*)	103.1
**2′** **β**				
**3′**	4.57 (1H, dd, *11.4, 4.3*)	79.4	-	149.1
**4′**	-	37.5	-	140.3
**5′**	1.38 (1H, dd, *10.7*; *5.7*)	49.5	-	143.8
**6′α**	2.02 (2H, m)	23.0	7.28 (1H, d, *1.5*)	109.5
**6′β**				
**7′**	5.55 (br d, *5.2*)	123.5	-	-
**8′**	-	132.2	-	-
**9′**	2.23 (1H, br s)	53.5	-	-
**10′**	-	35.5	-	-
**11′α**	4.16 (1H, dd, *9.7*; *3.5*)	66.8	-	-
**11′β**	4.01 (1H, dd *9.7*; *5.7*)		-	-
**12′**	1.69 (3H, br s)	21.5	-	-
**13′**	0.93 (3H, s)	14.7	-	-
**14′**	0.97 (3H, s)	16.4	-	-
**15′**	0.89 (3H, s)	27.9	-	-
**1′′**	-	166.7	-	165.9
**2′′**	5.68 (1H, d, *1.3*)	116.7	5.8 (1H, br d, *1.3*)	115.4
**3′′**	-	156.3	-	158.8
**4′′**	2.17; br d; *1.3*; 3H	20.1	2.14 (3H, br d, *1.3*)	20.5
**5′′**	1.89; br d; *1.3*; 3H	27.3	1.91 (3H, br d, *1.3*)	27.6
**-O-CH_2_-O**	-	-	6.06 (2H, br q, *1.5*)	102.6
**-O-CH_3_**	-	-	3.93 (3H, s)	56.8

**Figure 9:**
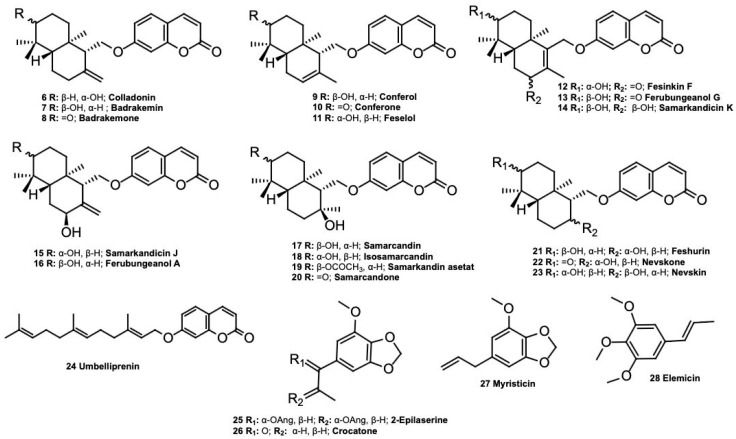
Known compounds isolated from *Ferula drudeana* roots.

The known compounds colladonin (**6**) [9], badrakemin (**7**) [9], badrakemone (**8**) [8], conferol (**9**) [8], conferone (**10**) [8], feselol (**11**) [8], fesinkin F (**12**) [10], ferubungeanol G (**13**) [10], samarcandicin K (**14**) [10], samarcandicin J (**15**) [10], ferubungeanol A (**16**) [10], samarcandin (**17**) [8], isosamarcandin (**18**) [11], samarcandin acetate (**19**) [8], samarcandone (**20**) [8], feshurin (**21**) [12], nevskone (**22**) [13], nevskin (**23**) [12], umbelliprenin (**24**) [14], 2-epilaserine (**25**) [15], crocatone (**26**) [16], myristicin (**27**) [17], and elemicin (**28**) [18] (Figure 9) were identified by comparison of their spectroscopic data with those of the literature data.

A review of the biological activities of related known compounds suggests that several traditional medicinal applications of silphion, including anti-inflammatory, antiproliferative, antimycobacterial, immunomodulatory, selective estrogen receptor modulatory (e.g., aphrodisiac and emmenagogue), and cardioprotective effects, may be attributed to these compounds (Table 3).

### 2.2. Cytotoxic Activity

The compounds isolated from *Ferula drudeana* were tested against kidney cancer cell lines (UO31 and A498) [74], mesothelioma cell lines representing two distinct histological subtypes of the disease MB24 (sarcomatoid) and MB52 (epithelioid) [75], together with a normal pleural mesothelial cell line NP1 [76] and a colon cancer cell line, (HCT116). The results are given in Table 4.

## 3. Discussion

In continuation of our preliminary study on the secondary metabolites of *F. drudeana* [77], an endemic species of Türkiye, subsequent isolation studies performed on the hexane extract of the roots of *F. drudeana* led to the identification of 23 sesquiterpene coumarin ethers and five phenylpropanoid derivatives. These molecules were evaluated for their cytotoxic activities using cell growth assays on kidney cancer cell lines (UO31, A498), DPM cell lines (MB52 and MB24), together with a normal pleural mesothelial cell line (NP1), and one colon cancer cell line (HCT116).

The results of cytotoxicity assays demonstrate that umbelliprenin (**24**) is the most cytotoxic molecule to kidney cancer cell lines, exhibiting IC_50_ values of 34 μM and 13 μM against A498 and UO31, respectively, but it showed no cytotoxicity in any of the other tested cell lines. Ferubungeanol A (**16**) exhibited considerable cytotoxicity against MPM cell lines, with IC_50_ values of 30 μM on MB52, 14 μM on NP1, and 22 μM on MB24.

The results of cytotoxicity assays demonstrate that umbelliprenin (**24**) is the most cytotoxic molecule to kidney cancer cell lines, exhibiting IC_50_ values of 34 μM and 13 μM against A498 and UO31, respectively, but it showed no cytotoxicity in any of the other tested cell lines. Ferubungeanol A (**16**) exhibited considerable cytotoxicity against MPM cell lines, with IC_50_ values of 30 μM on MB52, 14 μM on NP1, and 22 μM on MB24.

Ferubungeanol A (**16**) also demonstrated notable cytotoxicity against the UO31 cell line, with an IC_50_ value of 22 μM. Structurally, ferubungeanol A (**16**) is a derivative of badrakemin (**7**), bearing a hydroxyl group at the C-7′ position. In contrast, badrakemin (**7**) exhibited cytotoxic activity only against the UO31 cell line, with an IC_50_ value of 39 μM, and showed no activity against other cell lines. These findings suggested that the hydroxyl group at the C-7′ position plays a crucial role in enhancing the cytotoxic activity, highlighting its importance in structure-activity relationships.

Colladonin (**6**) is the C-3′ epimer of badrakemin (**7**). Although epimerization slightly enhances the activity of colladonin (**6**) against the cell lines, the comparable activity levels between colladonin (**6**) and badrakemin (**7**) suggest that this structural modification does not appreciably affect their cytotoxic potential. A comparable example can be observed with the molecules feselol (**11**) and conferol (**9**), which are also C-3′ epimers. The relationship between their activities mirrored that of colladonin (**6**) and badrakemin (**7**), further supporting the limited impact of C-3′ epimerization on cytotoxic activity. Samarcandin (**17**) and isosamarcandin (**18**) are also C-3′ epimers. In contrast to the colladonin-badrakemin and feselol-conferol epimer pairs, a noteworthy difference in cytotoxic activity is observed in the samarcandin-isosamarcandin epimer pair. While samarcandin (**17**) exhibited no cytotoxic activity against any tested cell lines, isosamarcandin (**18**) showed activity against MB52 (8.5 μM), NP1 (15 μM), and MB24 (25 μM) cell lines. The results indicate that the α-configuration of the hydroxyl group at the C-3′ position in isosamarcandin (**18**) significantly enhanced its cytotoxic activity, and these findings underscore the critical role of the α-hydroxyl group at the C-3′ position in modulating cytotoxic activity.

Druscoferol (**3**), a novel compound isolated from the roots of *Ferula drudeana*, is a derivative of feselol (**11**), wherein the coumarin moiety is scopoletin rather than umbelliferone. A comparison of the cytotoxic activities of druscoferol (**3**) and feselol (**11**) across all tested cell lines revealed highly similar results. Druscoferol (**3**) exhibited cytotoxicity against MB52 (IC_50_: 18 μM), MB24 (IC_50_: 18 μM), NP1 (IC_50_: 20 μM), HCT116 (IC_50_: 24 μM), and UO31 (IC_50_: 5.0 μM) cell lines, demonstrating an activity profile comparable to that of feselol (**11**). These findings suggest that methoxy substitution of the coumarin moiety does not significantly affect the cytotoxic activity of these compounds across the tested cell lines. Ferubungeanol G (**13**) emerged as another notable cytotoxic sesquiterpene coumarin, demonstrating activity, particularly against MB52 (IC_50_: 17 μM), NP1 (IC_50_: 15 μM), MB24 (IC_50_: 22 μM), and HCT116 (IC_50_: 26 μM) cell lines. The cytotoxic activity of feshurin (**21**), the C-8′ epimer of samarcandin (**17**), exhibited significant cytotoxicity in certain cell lines. Conversely, samarcandone (**20**), a derivative formed by oxidation of the hydroxyl group at the C-3′ position of samarcandin (**17**) into a ketone, showed no cytotoxic activity in any tested cell lines. Nevskone (**22**), the ketone derivative of feshurin (**21**), exhibited substantial cytotoxicity in specific cell lines. These results demonstrate that configurational and substitutional differences significantly influence the cytotoxic activity of these molecules.

## 4. Materials and Methods

### 4.1. General Experimental Procedures

LC-MS analyses were carried out using an 6130 Quadrupole LC/MS system (Agilent Technologies^®^, Santa Clara, CA, USA). UV–Vis spectra were recorded with a Nanodrop 2000 C (Thermo Scientific, Waltham, MA, USA) using a 1 mm quartz cuvette. IR spectra were measured on a Alpha FT-IR spectrometer (Bruker^®^, Billerica, MA, USA). NMR spectra were obtained using a Bruker^®^ Avance III spectrometer operating at 600 MHz for ^1^H and 150 MHz for ^13^C, respectively, with deuterated chloroform as the solvent. High-resolution electrospray ionization mass spectrometry (HRESIMS) data were collected with an Agilent^®^ 6530 Accurate Mass Q-TOF instrument. Optical rotation measurements were performed on a Autopol V Plus^®^ polarimeter using dichloromethane or methanol as the solvent (Rudolph Analytical, Hackettstown, NJ, USA). Initial fractionation was conducted using a Sephadex LH-20 column (5 × 100 cm, 25–100 µm, (Sigma Chem. Co., GE Healthcare, Chicago, IL, USA). Further purification of the compounds was achieved using a HPLC 2050 system (Gilson^®^, Saint-Avé, France). Chromatographic procedures utilized solvents, including hexane, dichloromethane, methanol, and acetonitrile, which were purchased from Sigma-Aldrich.

### 4.2. Plant Material

The plant material used in this study was collected from Mount Hasan in Aksaray Province, Türkiye, on 28 May 2019. The plant material was identified as *Ferula drudeana* Korovin by Professors Emine Akalin and Mahmut Miski. The herbarium specimen was archived in the herbarium of Istanbul University Faculty of Pharmacy (ISTE 115986).

### 4.3. Extraction and Isolation

The dried and coarsely powdered roots (900 g) of *F. drudeana* were extracted by maceration at room temperature with hexane (2 × 1 L) for 1 h in a Soxhlet extractor. After maceration, the plant material was further subjected to continuous extraction with hexane to extract medium-polar compounds such as sesquiterpene coumarins and phenyl propanoids. The solvent was evaporated under reduced pressure in a rotary evaporator at 35 °C, and a hexane extract of ca. 28 g was obtained, which was subjected to a silica (0.2–0.5 mm) open column to obtain 18 fractions. Each fraction was checked with LC-MS and combined to yield 10 fractions. Fractions that contain sesquiterpene coumarins and phenyl propanoids were fractionated on a Sephadex LH-20 column (5 × 100 cm) using a mobile-phase (hexane: dichloromethane: methanol with 7:4:1) solvent system. The secondary metabolite profile of each fraction was examined by LC-MS, and similar fractions were combined to yield 30 fractions. All fractions that were more than 20 mg were further purified on a reverse-phase preparative HPLC with gradient elution at a flow rate of 9 mL/min for 70 min. Fractions with less than 20 mg were subjected to reverse-phase semi-preparative HPLC purification at a flow rate of 4 mL/min for 70 min. During HPLC purification, chromatograms were observed at 200–600 nm, 210 nm, 254 nm, 280 nm, and 366 nm wavelengths. Phenomenex^®^ Luna C_18_ columns (21.2 × 150 mm and 10 × 250 mm) were used for purification. Acetonitrile and water were used as the mobile phase. The mobile phase systems were modified based on the characteristics of fractions.

Druferone (**1**): Amorphous white powder, [α]^24^_D_: −11° (c, 0.4, MeOH); UV (c, 0.015 mg/mL) (MeOH) λ_max_ (log ε) nm: 247 (sh) (4.09), 324 (4.09). IR υ_max_ (cm^−1^): 2959, 2920, 2850, 1734, 1707. ^1^H- and ^13^C-NMR, see Table 1. HRESIMS *m*/*z* [M + H]^+^ 395.1855 (calculated for C_24_H_27_O_5_: 395.1853).

Druferol (**2**): Amorphous white powder, [α]^24^_D_: −24° (c, 2.0, MeOH); UV (c, 0.015 mg/mL) (MeOH) λ_max_ (log ε) nm: 202 (4.58), 327 (4.07). IR υ_max_ (cm^−1^): 3431, 3085, 2959, 2930, 2869, 1728. ^1^H- and ^13^C-NMR data, see Table 1. HRESIMS *m*/*z* [M + H]^+^ 399.2180 (calculated for C_24_H_31_O_5_: 399.2166).

Druscoferol (**3**): Amorphous white powder, [α]^24^_D_: −55.91° (c, 8.5, MeOH); UV (c, 0.015 mg/mL) (MeOH) λ_max_ (log ε) nm: 232 (4.75), 299 (sh) (4.29), 345 (4.56). IR υmax (cm^−1^): 3461, 3083, 2960, 2932, 2854, 1718. 1H- and ^13^C-NMR data, see Table 1. HRESIMS *m*/*z* [M + H]^+^ 413.2336 (calculated for C_25_H_33_O_5_: 413.2323).

Feselol senecioate (**4**): Amorphous white powder, [α]^24^_D_: −107.06° (c, 3.4, MeOH); UV (c, 0.015 mg/mL) (MeOH) λ_max_ (log ε) nm: 220 (sh) (4.45), 326 (4.12). IR υ_max_ (cm^−1^): 3082, 2969, 2940, 2923, 2853, 1735. ^1^H- and ^13^C-NMR data, see Table 2. HRESIMS *m*/*z* [M + Na]^+^ 487.2460 (calculated for C_29_H_36_O_5_Na: 487.2455).

Drudeanone (**5**): Amorphous white powder, [α]^24^_D_: 0° (c, 3.5, MeOH); UV (c, 0.02 mg/mL) (MeOH) λ_max_ (log ε) nm: 212 (5.23), 304 (4.52). FT-IR υ_max_ (cm^−1^): 2939, 1694. ^1^H- and ^13^C-NMR data, see Table 2. HRESIMS *m*/*z* [M + H]^+^ 307.1182 (calculated for C_16_H_18_O_6_: 307.1181).

### 4.4. Cytotoxic Activity Studies

The XTT bioactivity test is an in vitro colorimetric cytotoxic activity test developed by the NCI MTP Assay Development and Screening Section [74] and used for this study. Kidney (A498, UO31) and colon (HCT116) cancer cell lines were used during the tests. These cell lines were obtained from the NCI Developmental Therapeutics Program. RPMI-1640 (Roswell Park Memorial Institute, Buffalo, NY, USA) medium, 10% FBS (fetal bovine serum), 1% glutamine, and 1% penicillin/streptomycin solutions were used for cell growth and treatment. Transfers were performed under laminar airflow in a sterile environment. The suspension containing the cells was seeded into 384-well plates with a volume of 45 µL with 3.5 × 10^5^ cells per well. Then, the plate was incubated at 37 °C and 5% CO_2_ for 24 h. The pure compounds prepared in DMSO were added in a 10-concentration series and incubated for another 48 h. After incubation, 10 µL of the tetrazolium salt XTT (2,3-bis [2-methoxy-4-nitro-5-sulfophenyl]-2H-tetrazolium-5-carboxanolide) solution was applied to the cells. After 4 h of incubation, dead cells were not stained with formazan dye, while viable cells could be counted in the EnVision plate reader using UV absorbance at 450 nm and 650 nm. Sanguinarine chloride hydrate was used as a positive control in the experiment. All assays involved duplicate wells for each sample concentration.

The other cytotoxicity assay was performed on diffuse pleural mesothelioma (DPM) cell lines representing two distinct histological subtypes of the disease MB52 (epithelioid) and MB24 (sarcomatoid) as well as NP1, a normal pleural mesothelioma cell line for comparison. This assay was developed by the National Cancer Institute, Thoracic Surgery Branch, Bethesda, and the methodologies for generating the NP1, MB24, and MB52 cell lines have been published [75,76], respectively. For this assay, the molecules were dissolved in DMSO and prepared at a stock concentration of 6 mM. The stock solution was diluted 200-fold to achieve an initial test concentration of 30 μM, and growth was assessed at 72 h. Sanguinarine chloride hydrate was also used as a positive control in the experiment, with an IC_50_ value of 1 μM for all three cell lines. All assays involved duplicate wells for each sample concentration.

### 4.5. Computational Details

Conformational searches were performed in an energy window of 5 kcal/mol using MacroModel, Schrödinger 2024 for **1** and **2**. The low-energy conformers were optimized with Gaussian 16 using DFT/B3LYP/DGDZVP in MeOH for **1** and **2**. The optimized conformers with Boltzmann distributions (%) greater than 1% were further submitted to the ECD calculation to generate the simulated ECD spectra using TDDFT/M062X/DGDZVP in MeOH for **1**. The calculated ECD spectra were further averaged based on the Boltzmann distributions to afford the theoretical ECD spectra for **1**.

## 5. Conclusions

This study represents the first comprehensive isolation study on the roots of *Ferula drudeana*, an endemic species of Türkiye. A total of 28 compounds were isolated, and their cytotoxic activities were evaluated using cell-based assays developed at the National Cancer Institute. As a result, 11 of the sesquiterpene coumarin derivatives demonstrated measurable cytotoxicity.

## Figures and Tables

**Table 3:** Biological activities of the known secondary metabolites of *Ferula drudeana*.

Secondary Metabolite	Biological Activities
**Colladonin (6)**	Cytotoxic against the COLO205 (colon), KM12 (colon), A498 (kidney carcinoma), UO31 (renal), TC32 (Ewing’s sarcoma) [19], HCT116 (human colorectal), HT-29 (human colorectal) [20], MCF-7, K-562 [11], A-549, U87MG, PC-3, and HEK293 [21] cancer cell lines, acetylcholinesterase (AChE) and butyrylcholinesterase (BChE) [22], carbonic anhydrase enzyme inhibition [23], endocannabinoid system modulator [24], α-amylase inhibition [25].
**Badrakemin (7)**	Cytotoxic against the KM12 (colon), A498 (kidney carcinoma), and UO31 (renal) cancer cell lines [19], acetylcholinesterase (AChE), butyrylcholinesterase (BChE), and tyrosinase (TYR) inhibition activity [22,26].
**Badrakemone (8)**	Cytotoxic against the UO31 (renal) [19], COLO205, K-562, MCF-7, HUVEC [8], A-549, U87MG, PC-3, and HEK293 [21] cancer cell lines, cancer chemoprevention [27], weak matrix metalloproteinase inhibitor [28], antivitiligo [10], anti-inflammatory [29], α-amylase inhibition [25].
**Conferol (9)**	Cytotoxic against the COLO205, K-562, MCF-7, and HUVEC [8] cell lines, urease inhibitor [30], modulators of multi-drug resistance in clinical isolates of *Escherichia coli* and *Staphylococcus aureus* [31], antileishmanial [32], antiviral against influenza A (H1N1) virus, cytotoxic against the HepG2 (hepatocellular carcinoma), Hep3B (hepatocellular carcinoma), and MCF-7 (estrogen-responsive mammalian adenocarcinoma) cancer cell lines [33].
**Conferone (10)**	Urease inhibitor [30], cytotoxic activity against the CH1 (ovarian), A549 (lung), SK-MEL-28 (melanoma) [34], COLO205, K-562, MCF-7 [8], and MT-2 [35] cell lines, cancer chemoprevention [27], antivitiligo [10].
**Feselol (11)**	Cytotoxic against COLO205, K-562, MCF-7, and HUVEC 7 [8], cancer chemoprevention [27], potential aphrodisiac [36], anti-inflammatory [27], antivitiligo [10].
**Fesinkin F (12)**	Cytotoxic against the HeLa cell line [10], antivitiligo [10], anti-inflammatory [27].
**Ferubungeanol G (13)**	Antivitiligo [10].
**Samarcandicin K (14)**	Antivitiligo [10].
**Samarcandicin J (15)**	Antivitiligo [10].
**Samarcandin (17)**	Potential aphrodisiac [36], antifungal [37], cytotoxic against the COLO205, K-562, MCF-7, HUVEC [8], AGS (human gastric carcinoma), and WEHI-164 (fibrosarcoma) cancer cell lines [38], active in NCI yeast anticancer drug-screen assays [39], potential antiviral activity against Ebola virus [40,41], antidiabetic [42], acetylcholinesterase (AChE) and carbonic anhydrase enzyme inhibition activity [23].
**Isosamarcandin (18)**	Cytotoxic against the COLO205, K-562, MCF-7, and HUVEC cancer cell lines [11], anti-inflammatory [29].
**Samarcandin Acetate (19)**	Potential aphrodisiac [36], cytotoxic against the COLO205, K-562, MCF-7, HUVEC [8] HeLa, and HT-29 cell lines [10], antivitiligo [10].
**Samarcandone (20)**	Anti-inflammatory [29], cytotoxic against the COLO205, K-562, MCF-7, and HUVEC cell lines [8].
**Feshurin (21)**	Cytotoxic against Mino cell line [43], antivitiligo [44].
**Nevskin (23)**	Cytotoxic against MV-4-11, Mino cell line [43].
**Umbelliprenin (24)**	Antigenotoxic [45], antioxidant, anti-inflammatory, lipoxygenase inhibitor [46], matrix metalloproteinase inhibitor [28], antitumor [47], cytotoxic activity against the CH1 (ovarian), A549 (lung), SK-MEL-28 (melanoma) [34], M4Beu (metastatic pigmented malignant melanoma), QU-DB (large cell lung) [48], COLO205, HCT116, A498 and UO31 (renal) [49], and MCF-7 [50] cancer cell lines, modulator of melanogenesis [51], antihypertension [52], cancer chemoprevention [27], antiangiogenic [53], antimetastatic and immunostimulatory [54], acetylcholinesterase (AChE) and carbonic anhydrase enzyme inhibition activity [23], antifungal [55], adjunctive agent for the treatment of pancreatic cancer [56], antineuroinflammatory, anxyolitic [57], anti-inflammatory effects by inhibiting TNF-α [58], butyrlcholinesterase (BChE) activity [22], antidiabetic [42].
**2-Epilaserine (25)**	Cytotoxicity against HL-60 cell line [15].
**Crocatone (26)**	Cytotoxicity against the HeLa and K562 cell lines [59], inhibition of abnormal proliferation of pulmonary artery smooth muscle cells (PASMCs) [60], spasmolytic [16], antifeedant [61].
**Myristicin (27)**	Cancer chemopreventive agent [62], anti-inflammatory [63], antimicrobial against *B. subtilis*, *E. coli*, and *S. aureus* [64], hepatoprotective [65], cytoprotective against hypoxia-induced apoptosis and endoplasmic reticulum stress [66], downregulates expression of pro-inflammatory cytokines [67], cytotoxic against the SK-N-SH (human neuroblastoma) cancer cell line [68], antiproliferative [69], insecticidal against *Culex pipiens* and *Aedes aegypti* [70], antifungal [71], cytotoxic against MCF-7 [72], MAO inhibitory [73].
**Elemicin (28)**	Antifungal [71].

**Table 4:** IC_50_ values of compounds isolated from *Ferula drudeana*.

Compound	IC_50_ (µM)					
	A498	UO31	MB24	MB52	NP1	HCT116
Druferone (**1**)	>50	>50	>30	**29**	**28**	>30
Druferol (**2**)	>50	>50	>30	>30	>30	>30
Druscoferol (**3**)	>50	**35**	**18**	**18**	**20**	**27**
Feselol senecioate (**4**)	>50	>50	>30	>30	>30	>30
Drudeanone (**5**)	>50	>50	>30	>30	>30	>30
Colladonin (**6**)	>50	**33**	**25**	**29**	**24**	>30
Badrakemin (**7**)	>50	**39**	>30	>30	>30	>30
Badrakemone (**8**)	>50	**35**	**27**	**24**	**25**	>30
Conferol (**9**)	>50	>50	**24**	>30	**27**	>30
Conferone (**10**)	>50	>50	>30	>30	>30	>30
Feselol (**11**)	>50	**31**	**23**	**20**	**21**	**28**
Fesinkin F (**12**)	NT *	NT	**28**	**19**	**24**	>30
Ferubungeanol G (**13**)	>50	**39**	**22**	**17**	**15**	**26**
Samarcandicin K (**14**)	>50	>50	>30	>30	>30	>30
Samarcandicin J (**15**)	>50	>50	>30	>30	>30	>30
Ferubungeanol A (**16**)	>50	**22**	**22**	**30**	**14**	>30
Samarcandin (**17**)	>50	**43**	>30	>30	>30	>30
Isosamarcandin (**18**)	NT	NT	**25**	**8.5**	**15**	>30
Samarcandin acetate (**19**)	>50	**37**	>30	>30	>30	>30
Samarcandone (**20**)	>50	>50	>30	>30	>30	>30
Feshurin (**21**)	>50	**48**	**27**	**21**	**24**	>30
Nevskone (**22**)	>50	>50	**12**	**7.0**	**11**	**22**
Umbelliprenin (**24**)	**34**	**13**	>30	>30	>30	>30
2-Epilaserine (**25**)	>50	>50	>30	>30	>30	>30
Crocatone (**26**)	>50	>50	>30	>30	>30	>30
Myristicin (**27**)	>50	>50	>30	>30	>30	>30
Elemicin (**28**)	>50	>50	>30	>30	>30	>30

* NT = Not tested.

## Data Availability

Data is available on request to the corresponding authors.

## Supplementary Materials

The following supporting information can be downloaded at https://www.mdpi.com/article/10.3390/molecules30091916/s1, Figure S1–S168. Spectroscopic data for compounds, detailed biological results, comparative HPLC traces for hexane extract of root and resin, and synthesis of compound **5**; Table S1. DFT/B3LYP/DGDZVP optimized conformers of model structure **5a** submitted for ECD simulations at TDDFT/B3LYP/DGDZVP in gas phase. Reference [78].
