# Supplementary material for: Sesquiterpene Coumarin Ethers and Phenylpropanoids from the Roots of Ferula drudeana, the Putative Anatolian Ecotype of the Silphion Plant
Source: Molecules. 2025 Apr 25;30(9):1916. doi: 10.3390/molecules30091916 (PMC12073360; doi:10.3390/molecules30091916)
Supplement: Supplementary file 1 [file molecules-30-01916-s001.zip › molecules-3545163-supplementary.pdf]

SUPPLEMENTARY MATERIAL

Sesquiterpene Coumarin Ethers and Phenylpropanoids Isolated from the Roots of *Ferula drudeana* Korovin, the Putative Anatolian Ecotype of the Silphion Plant

Fadıl Kaan Kuran [1,2,3], Sarath P. D. Senadeera [3], Dongdong Wang [3], Ji-Yeon Hwang [3], Ekaterina Goncharova [3,4], Jennifer Wilson [3], Antony Wamiru [3], Brice A. P. Wilson [3], Nathanael Pruett [5], Lin Du [3], Chuong D. Hoang [5], John A. Beutler [3,*] and Mahmut Miski [1,*]

[1] Department of Pharmacognosy, Faculty of Pharmacy, Istanbul University, Istanbul 34116, Türkiye;
kaankuran@istanbul.edu.tr

[2] Department of Pharmacognosy, Institute of Graduate Studies in Health Sciences, Istanbul University,
Istanbul 34116, Türkiye

[3] Molecular Targets Program, National Cancer Institute, Frederick, MD 21702, USA;
spdsenadeera@gmail.com (S.P.D.S.); dongdong.wang@nih.gov (D.W.);
jiyeon.hwang@nih.gov (J.-Y.H.); katya.goncharova@nih.gov (E.G.);
wilsonje@mail.nih.gov (J.W.); lin.du@nih.gov (L.D.)

[4] Advanced Biomedical Computational Science, Frederick National Laboratory for Cancer Research,
Frederick, MD 21702, USA

[5] Thoracic Surgery Branch, National Cancer Institute, Bethesda, MD 20892, USA;
chuong.hoang@nih.gov

* Correspondence: beutlerj@mail.nih.gov (J.A.B.); mahmut.miski@gmail.com (M.M.)

Table of Contents

Figure S1: Structures of sesquiterpene coumarins and phenylpropanoids from Ferula drudeana.......... 7
Figure S2: ^{1}H NMR spectrum (600 MHz, $CDCl_3$) of druferone (**1**) 8
Figure S3: ^{13}C NMR spectrum (150 MHz, $CDCl_3$) of druferone (**1**) 8
Figure S4: COSY spectrum of druferone (**1**).. 9
Figure S5: HSQC spectrum of druferone (**1**) 9
Figure S6: HMBC spectrum of druferone (**1**) 10
Figure S7: NOESY spectrum of druferone (**1**).. 10
Figure S8: HRESIMS spectrum of druferone (**1**)... 11
Figure S9: IR spectrum of druferone (**1**)... 11
Figure S10: UV spectrum of druferone (**1**) 12
Figure S11: ^{1}H NMR spectrum (600 MHz, $CDCl_3$) of druferol (**2**) 12
Figure S12: ^{13}C NMR spectrum (150 MHz, $CDCl_3$) of druferol (**2**) 13
Figure S13: COSY spectrum of druferol (**2**) 13
Figure S14: HSQC spectrum of druferol (**2**) 14
Figure S15: HMBC spectrum of druferol (**2**) 14
Figure S16: NOESY spectrum of druferol (**2**)... 15
Figure S17: HRESIMS spectrum of druferol (**2**) 15
Figure S18: IR spectrum of druferol (**2**).. 16
Figure S19: UV spectrum (MeOH) of druferol (**2**) 16
Figure S20: ^{1}H NMR spectrum (600 MHz, $CDCl_3$) of druscoferol (**3**)... 17
Figure S21: ^{13}C NMR spectrum (150 MHz, $CDCl_3$) of druscoferol (**3**).. 17
Figure S22: COSY spectrum of druscoferol (**3**).. 18
Figure S23: HSQC spectrum of druscoferol (**3**).. 18
Figure S24: HMBC spectrum of druscoferol (**3**)... 19
Figure S25: NOESY spectrum of druscoferol (**3**) 19
Figure S26: HRESIMS spectrum of druscoferol (**3**) 20
Figure S27: IR spectrum of druscoferol (**3**) 20
Figure S28: UV spectrum (MeOH) of druscoferol (**3**)... 21
Figure S29: ^{1}H NMR spectrum (600 MHz, $CDCl_3$) of feselol senecioate (**4**).................................... 21
Figure S30: ^{13}C NMR spectrum (150 MHz, $CDCl_3$) of feselol senecioate (**4**).................................. 22
Figure S31: COSY spectrum of feselol senecioate (**4**)... 22
Figure S32: HSQC spectrum of feselol senecioate (**4**)... 23
Figure S33: HMBC spectrum of feselol senecioate (**4**).. 23
Figure S34: NOESY spectrum of feselol senecioate (**4**) 24
Figure S35: HRESIMS spectrum of feselol senecioate (**4**) 24
Figure S36: IR spectrum of feselol senecioate (**4**) 25
Figure S37: UV spectrum (MeOH) of feselol senecioate (**4**).. 25
Figure S38: ^{1}H NMR spectrum (600 MHz, $CDCl_3$) of drudeanone (**5**) 26
Figure S39: ^{13}C NMR spectrum (150 MHz, $CDCl_3$) of drudeanone (**5**) 26

Figure S40: COSY spectrum of drudeanone (**5**) 27
Figure S41: HSQC spectrum of drudeanone (**5**) 27
Figure S42: HMBC spectrum of drudeanone (**5**) 28
Figure S43: NOESY spectrum of drudeanone (**5**) 28
Figure S44: UV spectrum (MeOH) of drudeanone (**5**) 29
Figure S45: IR spectrum of drudeanone (**5**) 29
Figure S46: HRESIMS spectrum of drudeanone (**5**) 30
Figure S47: ^{1}H NMR spectrum (600 MHz, $CDCl_3$) of colladonin (**6**) 30
Figure S48: LC-MS spectrum of colladonin (**6**) 31
Figure S49: ^{1}H NMR spectrum (600 MHz, $CDCl_3$) of badrakemin (**7**) 32
Figure S50: LC-MS spectrum of badrakemin (**7**) 33
Figure S51: ^{1}H NMR spectrum (600 MHz, $CDCl_3$) of badrakemone (**8**) 33
Figure S52: LC-MS spectrum of badrakemone (**8**) 34
Figure S53: ^{1}H NMR spectrum (600 MHz, $CDCl_3$) of conferol (**9**) 34
Figure S54: LC-MS spectrum of conferol (**9**) 35
Figure S55: ^{1}H NMR spectrum (600 MHz, $CDCl_3$) of conferone (**10**) 35
Figure S56: LC-MS spectrum of conferone (**10**) 36
Figure S57: ^{1}H NMR spectrum (600 MHz, $CDCl_3$) of feselol (**11**) 36
Figure S58: LC-MS spectrum of feselol (**11**) 37
Figure S59: ^{1}H NMR spectrum (600 MHz, $CDCl_3$) of fesinkin F (**12**) 37
Figure S60: ^{1}H NMR spectrum (600 MHz, $CDCl_3$) of ferubungeanol G (**13**) 38
Figure S61: LC-MS spectrum of ferubungeanol G (**13**) 38
Figure S62: ^{1}H NMR spectrum (600 MHz, $CDCl_3$) of samarkandicin K (**14**) 39
Figure S63: LC-MS spectrum of samarkandicin K (**14**) 39
Figure S64: ^{1}H-NMR spectrum (600 MHz, $CDCl_3$) of samarkandicin J (**15**) 40
Figure S65: ^{1}H NMR spectrum (600 MHz, $CDCl_3$) of ferubungeanol A (**16**) 40
Figure S66: LC-MS spectrum of ferubungeanol A (**16**) 41
Figure S67: ^{1}H NMR spectrum (600 MHz, $CDCl_3$) of samarcandin (**17**) 41
Figure S68: LC-MS spectrum of samarcandin (**17**) 42
Figure S69: ^{1}H NMR spectrum (600 MHz, $CDCl_3$) of isosamarcandin (**18**) 42
Figure S70: ^{1}H NMR spectrum (600 MHz, $CDCl_3$) of samarcandin acetate (**19**) 43
Figure S71: LC-MS spectrum of samarcandin acetate (**19**) 43
Figure S72: ^{1}H NMR spectrum (600 MHz, $CDCl_3$) of samarcandone (**20**) 44
Figure S73: LC-MS spectrum of samarcandone (**20**) 44
Figure S74: ^{1}H NMR spectrum (600 MHz, $CDCl_3$) of feshurin (**21**) 45
Figure S75: LC-MS spectrum of feshurin (**21**) 45
Figure S76: ^{1}H NMR spectrum (600 MHz, $CDCl_3$) of feshurone (nevskone) (**22**) 46
Figure S77: ^{1}H NMR spectrum (600 MHz, $CDCl_3$) of nevskin (**23**) 46
Figure S78: ^{1}H NMR spectrum (600 MHz, $CDCl_3$) of umbelliprenin (**24**) 47
Figure S79: LC-MS spectrum of umbelliprenin (**24**) 47
Figure S80: ^{1}H NMR spectrum (600 MHz, $CDCl_3$) of 2-epilaserine (**25**) 48
Figure S81: ^{1}H NMR spectrum (600 MHz, $CDCl_3$) of crocatone (**26**) 48
Figure S82: ^{1}H NMR spectrum (600 MHz, $CDCl_3$) of myristicin (**27**) 49
Figure S83: ^{1}H NMR spectrum (600 MHz, $CDCl_3$) of elemicin (**28**) 49
Figure S84: Stacked ^{1}H NMR spectra of feselol (**11**, top) and druscoferol (**3**, bottom) 50
Figure S85: Stacked ^{1}H NMR spectra of feselol (**11**, top) and feselol senecioate (**4**, bottom) 50
Figure S86: Stacked ^{1}H NMR spectra of druferone (**1**, bottom) and ferubungeanol G (**13**, top) 51
Figure S87: Stacked ^{1}H NMR spectra of druferol (**2**, top) and samarcandicin K (**14**, bottom) 51

Figure S88: Stacked ^{1}H NMR spectra of crocatone (**26**, top) and drudeanone (**5**, bottom) 52
Figure S89: Concentration-dependent cytotoxic effects of druferone (**1**) on A498 and UO31 cell lines 52
Figure S90: Concentration-dependent cytotoxic effects of druferol (**2**) on A498 and UO31 cell lines 53
Figure S91: Concentration-dependent cytotoxic effects of druscoferol (**3**) on A498 and UO31 cell lines 53
Figure S92: Concentration-dependent cytotoxic effects of feselol senecioate (**4**) on A498 and UO31 cell lines........ 54
Figure S93: Concentration-dependent cytotoxic effects of drudeanone (**5**) on A498 and UO31 cell lines 54
Figure S94: Concentration-dependent cytotoxic effects of colladonin (**6**) on A498 and UO31 cell lines 55
Figure S95: Concentration-dependent cytotoxic effects of badrakemin (**7**) on A498 and UO31 cell lines 55
Figure S96: Concentration-dependent cytotoxic effects of badrakemone (**8**) on A498 and UO31 cell lines 56
Figure S97: Concentration-dependent cytotoxic effects of conferol (**9**) on A498 and UO31 cell lines56
Figure S98: Concentration-dependent cytotoxic effects of conferone (**10**) on A498 and UO31 cell lines 57
Figure S99: Concentration-dependent cytotoxic effects of feselol (**11**) on A498 and UO31 cell lines 57
Figure S100: Concentration-dependent cytotoxic effects of ferubungeanol G (**13**) on A498 and UO31 cell lines........ 58
Figure S101: Concentration-dependent cytotoxic effects of samarcandicin K (**14**) on A498 and UO31 cell lines........ 58
Figure S102: Concentration-dependent cytotoxic effects of samarcandicin J (**15**) on A498 and UO31 cell lines........ 59
Figure S103: Concentration-dependent cytotoxic effects of ferubungeanol A (**16**) on A498 and UO31 cell lines........ 59
Figure S104: Concentration-dependent cytotoxic effects of samarcandin (**17**) on A498 and UO31 cell lines 60
Figure S105: Concentration-dependent cytotoxic effects of samarcandin Acetate (**19**) on A498 and UO31 cell lines........ 60
Figure S106: Concentration-dependent cytotoxic effects of sSamarcandone (**20**) on A498 and UO31 cell lines........ 61
Figure S107: Concentration-dependent cytotoxic effects of feshurin (**21**) on A498 and UO31 cell lines 61
Figure S108: Concentration-dependent cytotoxic effects of nevskone (**22**) on A498 and UO31 cell lines 62
Figure S109: Concentration-dependent cytotoxic effects of umbelliprenin (**24**) on A498 and UO31 cell lines........ 62
Figure S110: Concentration-dependent cytotoxic effects of 2-epilaserine (**25**) on A498 and UO31 cell lines 63
Figure S111: Concentration-dependent cytotoxic effects of crocatone (**26**) on A498 and UO31 cell lines 63
Figure S112: Concentration-dependent cytotoxic effects of myristicin (**27**) on A498 and UO31 cell lines 64
Figure S113: Concentration-dependent cytotoxic effects of elemicin (**28**) on A498 and UO31 cell lines 64

Figure S114: Concentration-dependent cytotoxic effects of druferone (**1**) on MB24, MB52, and NP1 cell lines 65
Figure S115: Concentration-dependent cytotoxic effects of druferol (**2**) on MB24, MB52, and NP1 cell lines 65
Figure S116: Concentration-dependent cytotoxic effects of druscoferol (**3**) on MB24, MB52, and NP1 cell lines 66
Figure S117: Concentration-dependent cytotoxic effects of feselol senecioate (**4**) on MB24, MB52, and NP1 cell lines 66
Figure S118: Concentration-dependent cytotoxic effects of drudeanone (**5**) on MB24, MB52, and NP1 cell lines 67
Figure S119: Concentration-dependent cytotoxic effects of colladonin (**6**) on MB24, MB52, and NP1 cell lines 67
Figure S120: Concentration-dependent cytotoxic effects of badrakemin (**7**) on MB24, MB52, and NP1 cell lines 68
Figure S121: Concentration-dependent cytotoxic effects of badrakemon (**8**) on MB24, MB52, and NP1 cell lines 68
Figure S122: Concentration-dependent cytotoxic effects of conferol (**9**) on MB24, MB52, and NP1 cell lines 69
Figure S123: Concentration-dependent cytotoxic effects of conferone (**10**) on MB24, MB52, and NP1 cell lines 69
Figure S124: Concentration-dependent cytotoxic effects of feselol (**11**) on MB24, MB52, and NP1 cell lines 70
Figure S125: Concentration-dependent cytotoxic effects of fesinkin F (**12**) on MB24, MB52, and NP1 cell lines 70
Figure S126: Concentration-dependent cytotoxic effects of ferubungeanol G (**13**) on MB24, MB52, and NP1 cell lines 71
Figure S127: Concentration-dependent cytotoxic effects of samarcandicin K (**14**) on MB24, MB52, and NP1 cell lines 71
Figure S128: Concentration-dependent cytotoxic effects of samarcandicin J (**15**) on MB24, MB52, and NP1 cell lines 72
Figure S129: Concentration-dependent cytotoxic effects of ferubungeanol A (**16**) on MB24, MB52, and NP1 cell lines 72
Figure S130: Concentration-dependent cytotoxic effects of samarcandin (**17**) on MB24, MB52, and NP1 cell lines 73
Figure S131: Concentration-dependent cytotoxic effects of isosamarcandin (**18**) on MB24, MB52, and NP1 cell lines 73
Figure S132: Concentration-dependent cytotoxic effects of samarcandin acetate (**19**) on MB24, MB52, and NP1 cell lines 74
Figure S133: Concentration-dependent cytotoxic effects of samarcandone (**20**) on MB24, MB52, and NP1 cell lines 74
Figure S134: Concentration-dependent cytotoxic effects of feshurin (**21**) on MB24, MB52, and NP1 cell lines 75
Figure S135: Concentration-dependent cytotoxic effects of feshurone (nevskone) (**22**) on MB24, MB52, and NP1 cell lines 75
Figure S136: Concentration-dependent cytotoxic effects of umbelliprenin (**24**) on MB24, MB52, and NP1 cell lines 76
Figure S137: Concentration-dependent cytotoxic effects of 2-epilaserine (**25**) on MB24, MB52, and NP1 cell lines 76

Figure S138: Concentration-dependent cytotoxic effects of crocatone (**26**) on MB24, MB52, and NP1 cell lines 77
Figure S139: Concentration-dependent cytotoxic effects of myristicin (**27**) on MB24, MB52, and NP1 cell lines 77
Figure S140: Concentration-dependent cytotoxic effects of elemicin (**28**) on MB24, MB52, and NP1 cell lines 78
Figure S141: Concentration-dependent cytotoxic effects of druferone (**1**) on HCT-116 cell line 78
Figure S142: Concentration-dependent cytotoxic effects of druferol (**2**) on HCT-116 cell line 79
Figure S143: Concentration-dependent cytotoxic effects of druscoferol (**3**) on HCT-116 cell line 79
Figure S144: Concentration-dependent cytotoxic effects of feselol senecioate (**4**) on HCT-116 cell line 80
Figure S145: Concentration-dependent cytotoxic effects of drudeanone (**5**) on HCT-116 cell line 80
Figure S146: Concentration-dependent cytotoxic effects of colladonin (**6**) on HCT-116 cell line 81
Figure S147: Concentration-dependent cytotoxic effects of badrakemin (**7**) on HCT-116 cell line 81
Figure S148: Concentration-dependent cytotoxic effects of badrakemone (**8**) on HCT-116 cell line 82
Figure S149: Concentration-dependent cytotoxic effects of conferol (**9**) on HCT-116 cell line 82
Figure S150: Concentration-dependent cytotoxic effects of conferone (**10**) on HCT-116 cell line 83
Figure S151: Concentration-dependent cytotoxic effects of feselol (**11**) on HCT-116 cell line 83
Figure S152: Concentration-dependent cytotoxic effects of fesinkin F (**12**) on HCT-116 cell line 84
Figure S153: Concentration-dependent cytotoxic effects of ferubungeanol G (**13**) on HCT-116 cell line 84
Figure S154: Concentration-dependent cytotoxic effects of samarcandicin K (**14**) on HCT-116 cell line 85
Figure S155: Concentration-dependent cytotoxic effects of samarcandicin J (**15**) on HCT-116 cell line 85
Figure S156: Concentration-dependent cytotoxic effects of ferubungeanol A (**16**) on HCT-116 cell line 86
Figure S157: Concentration-dependent cytotoxic effects of samarcandin (**17**) on HCT-116 cell line 86
Figure S158: Concentration-dependent cytotoxic effects of isosamarcandin (**18**) on HCT-116 cell line 87
Figure S159: Concentration-dependent cytotoxic effects of samarcandin acetate (**19**) on HCT-116 cell line 87
Figure S160: Concentration-dependent cytotoxic effects of samarcandone (**20**) on HCT-116 cell line 88
Figure S161: Concentration-dependent cytotoxic effects of feshurin (**21**) on HCT-116 cell line 88
Figure S162: Concentration-dependent cytotoxic effects of nevskone (**22**) on HCT-116 cell line 89
Figure S163: Concentration-dependent cytotoxic effects of umbelliprenin (**24**) on HCT-116 cell line 89
Figure S164: Concentration-dependent cytotoxic effects of 2-epilaserine (**25**) on HCT-116 cell line 90
Figure S165: Concentration-dependent cytotoxic effects of crocaton (**26**) on HCT-116 cell line 90
Figure S166: Concentration-dependent cytotoxic effects of myristicin (**27**) on HCT-116 cell line 91
Figure S167: Concentration-dependent cytotoxic effects of elemicin (**28**) on HCT-116 cell line 91
Figure S168: HPLC profiles of the resin and the hexane extract of F. drudeana 92

Experimental: Synthesis and chiral separation of 1-(7-methoxybenzo[d][1,3]dioxol-5-yl)-1-oxopropan-2-yl 3-methylbut-2-enoate (5) 93

Table S1: DFT/B3LYP/DGDZVP optimized conformers of model structure **5a** submitted for ECD simulations at TDDFT/B3LYP/DGDZVP in gas phase..94

Supplemental Reference...99

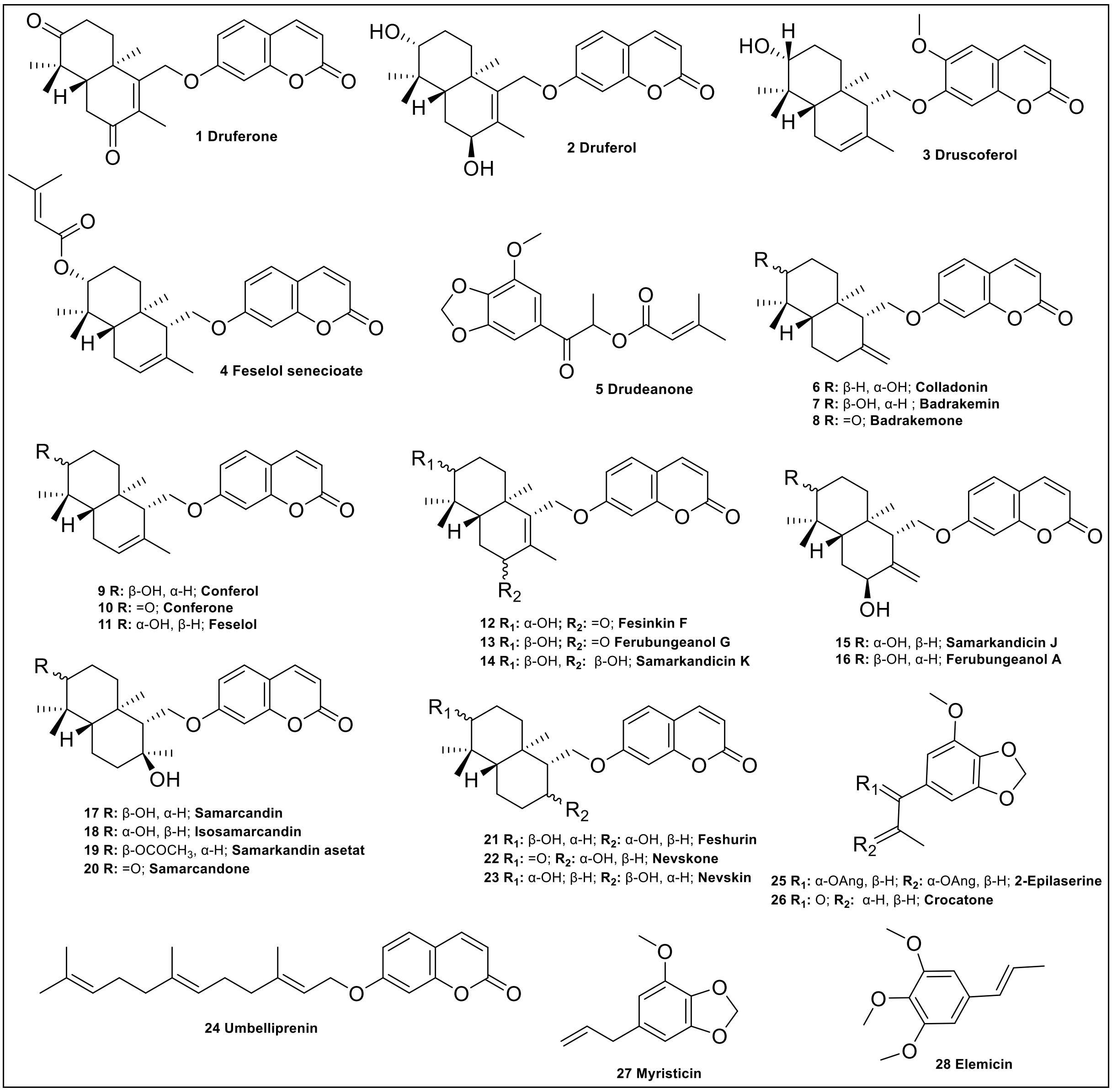

Figure S1: Structures of sesquiterpene coumarins and phenylpropanoids from *Ferula drudeana.*

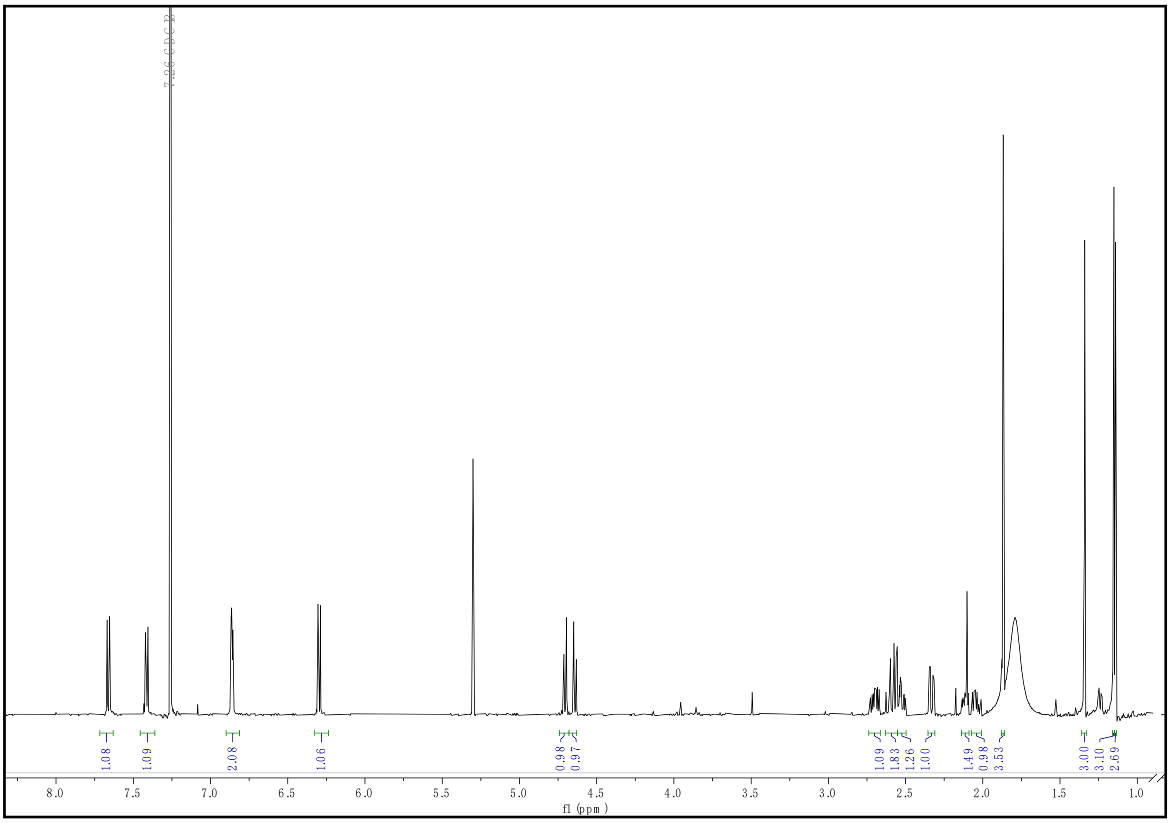

Figure S2: ^{1}H NMR spectrum (600 MHz, $CDCl_3$) of druferone (**1**).

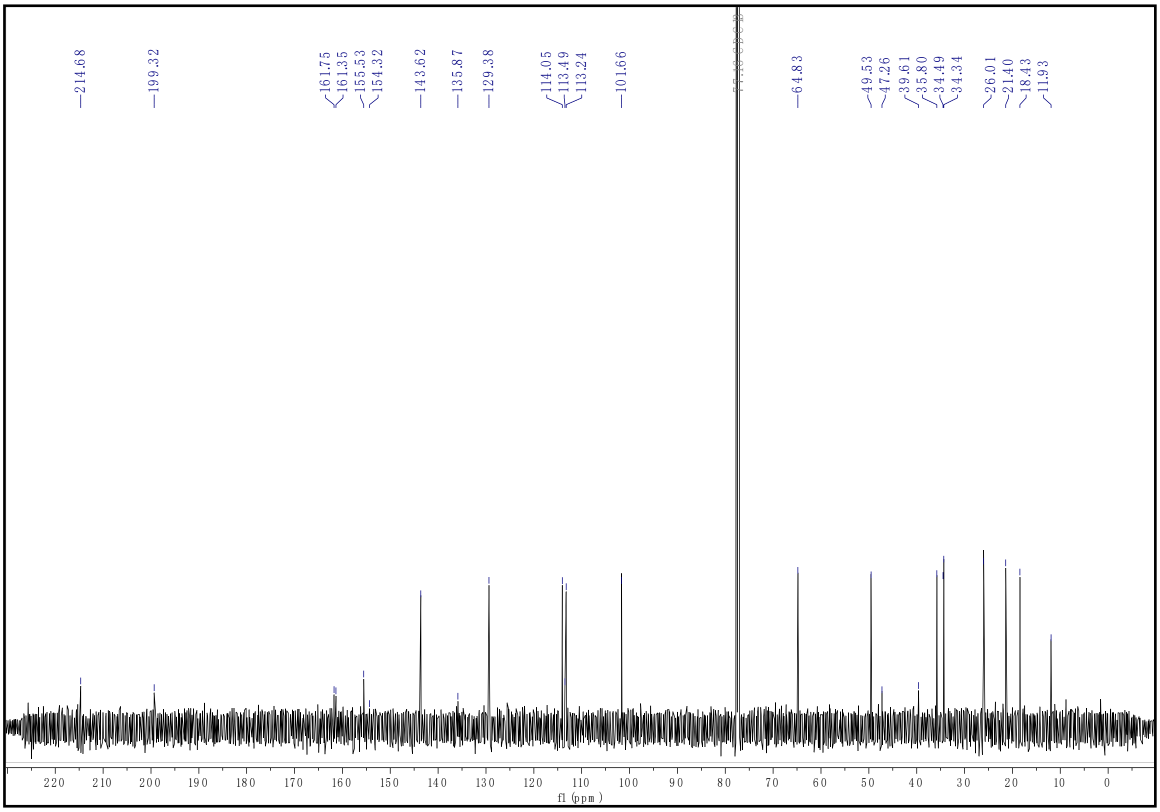

Figure S3: ^{13}C NMR spectrum (150 MHz, $CDCl_3$) of druferone (**1**).

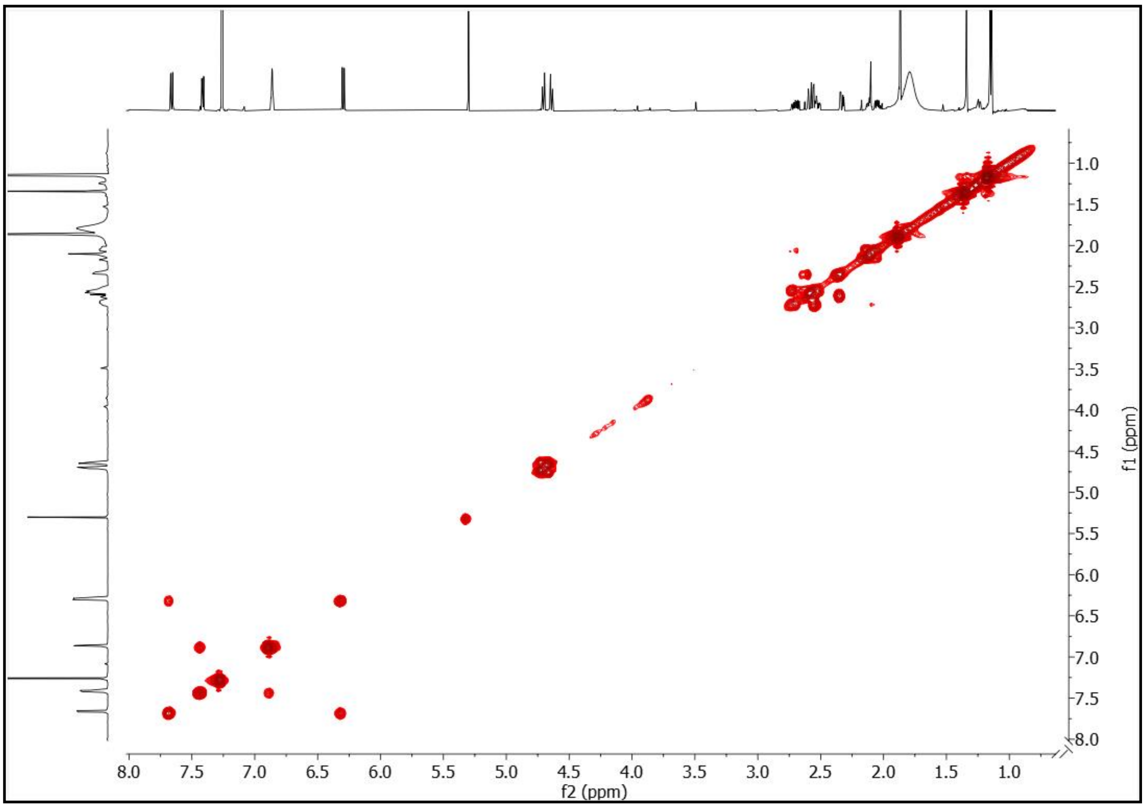

Figure S4: COSY spectrum of druferone (**1**).

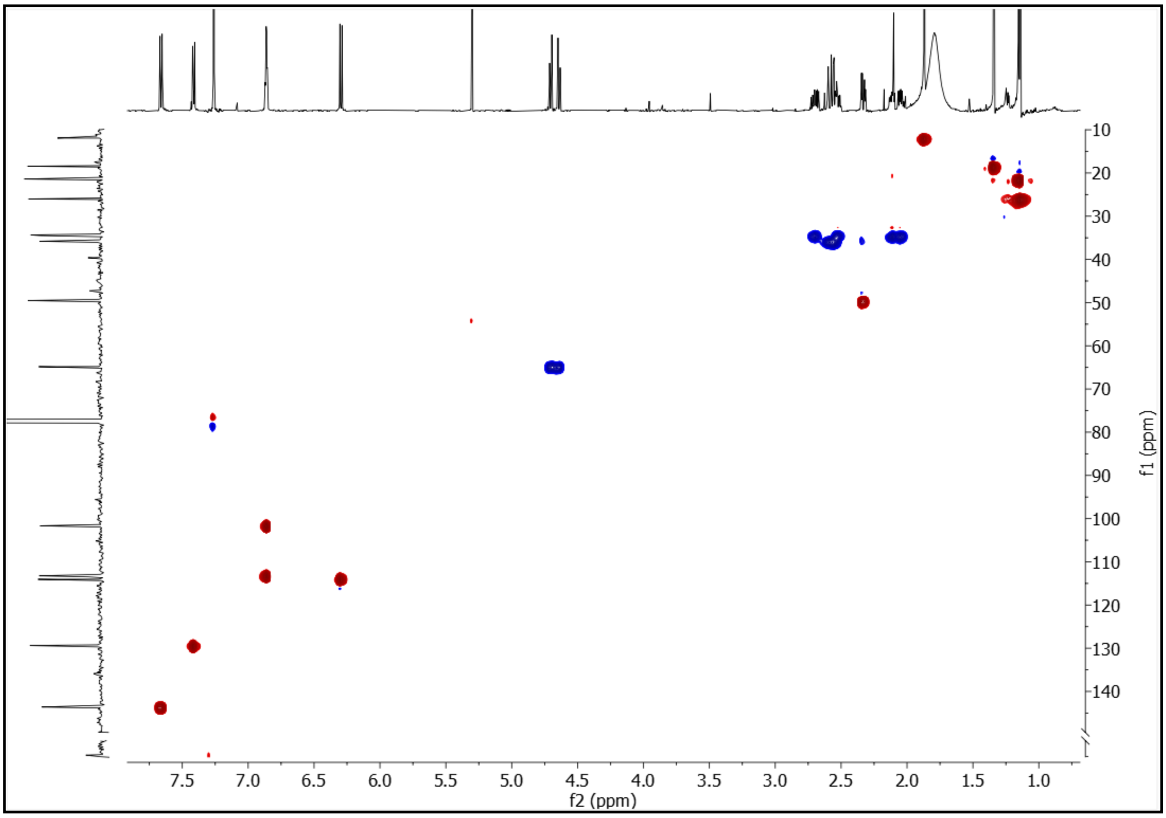

Figure S5: HSQC spectrum of druferone (**1**).

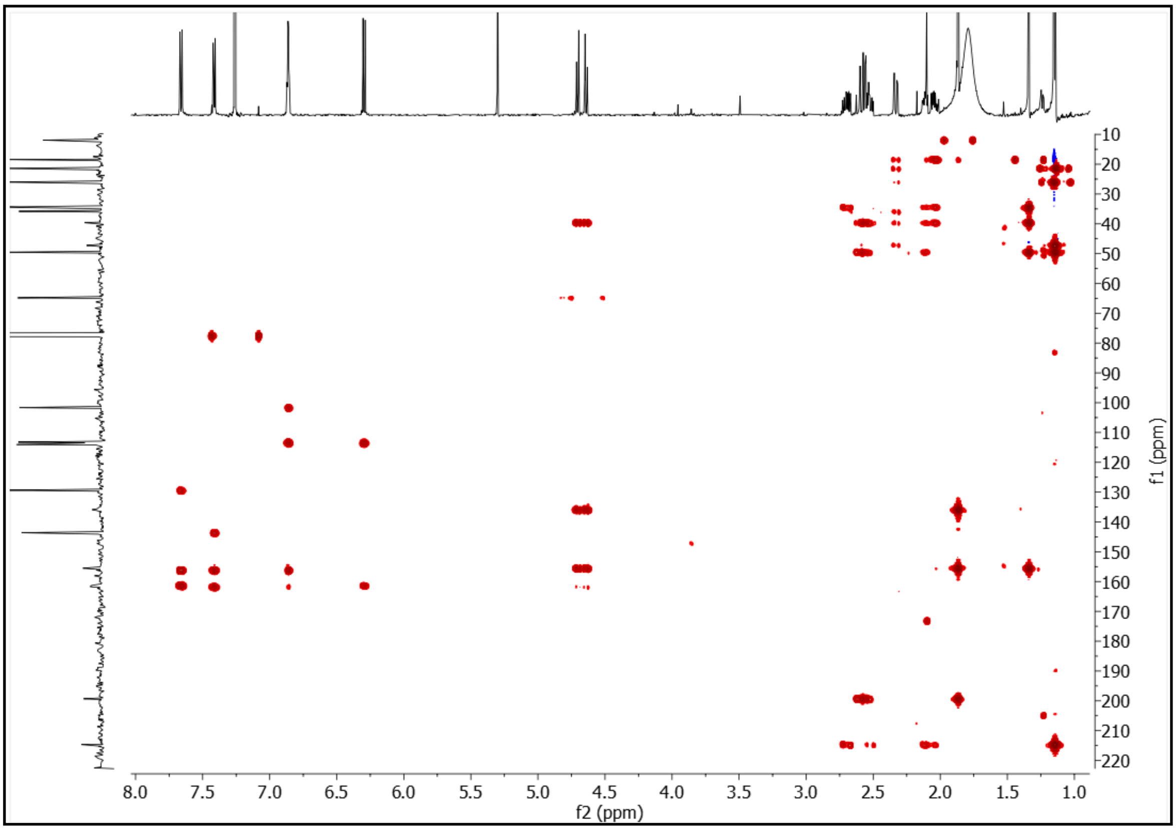

Figure S6: HMBC spectrum of druferone (**1**).

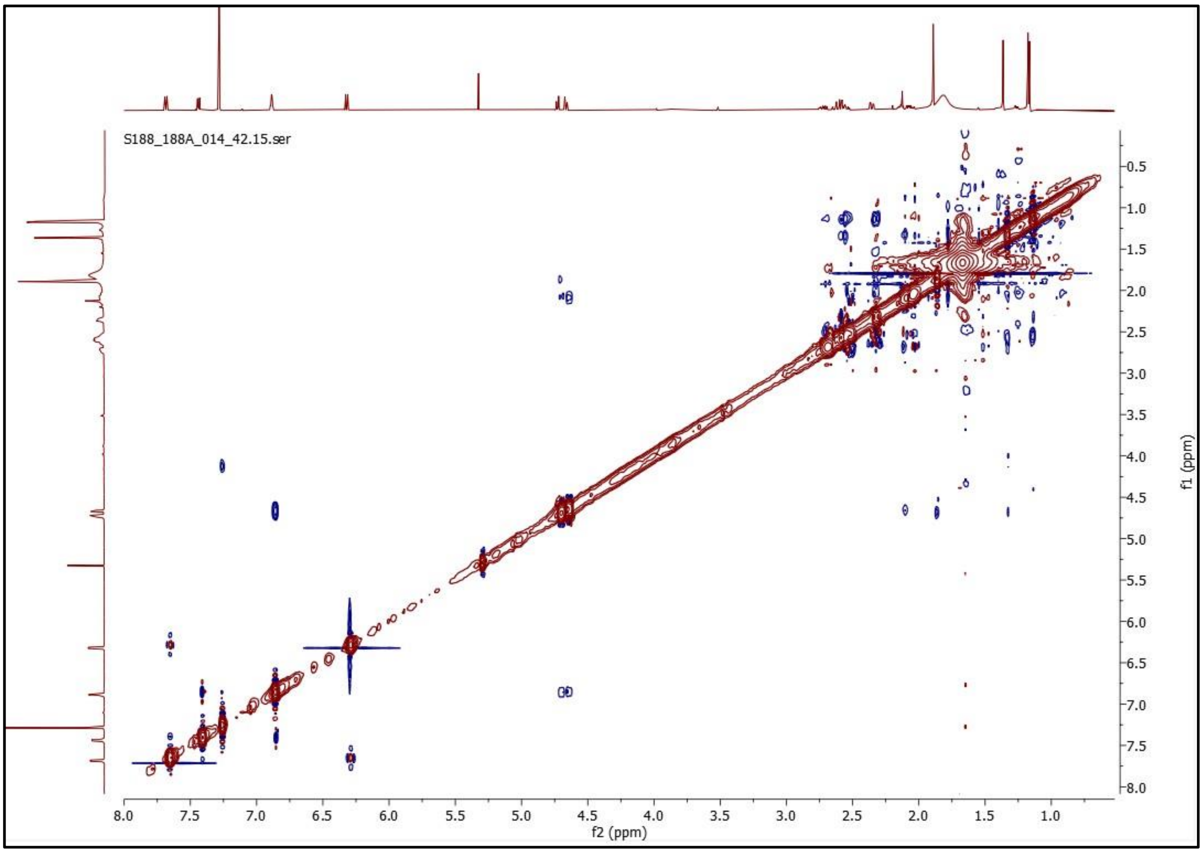

Figure S7: NOESY spectrum of druferone (**1**).

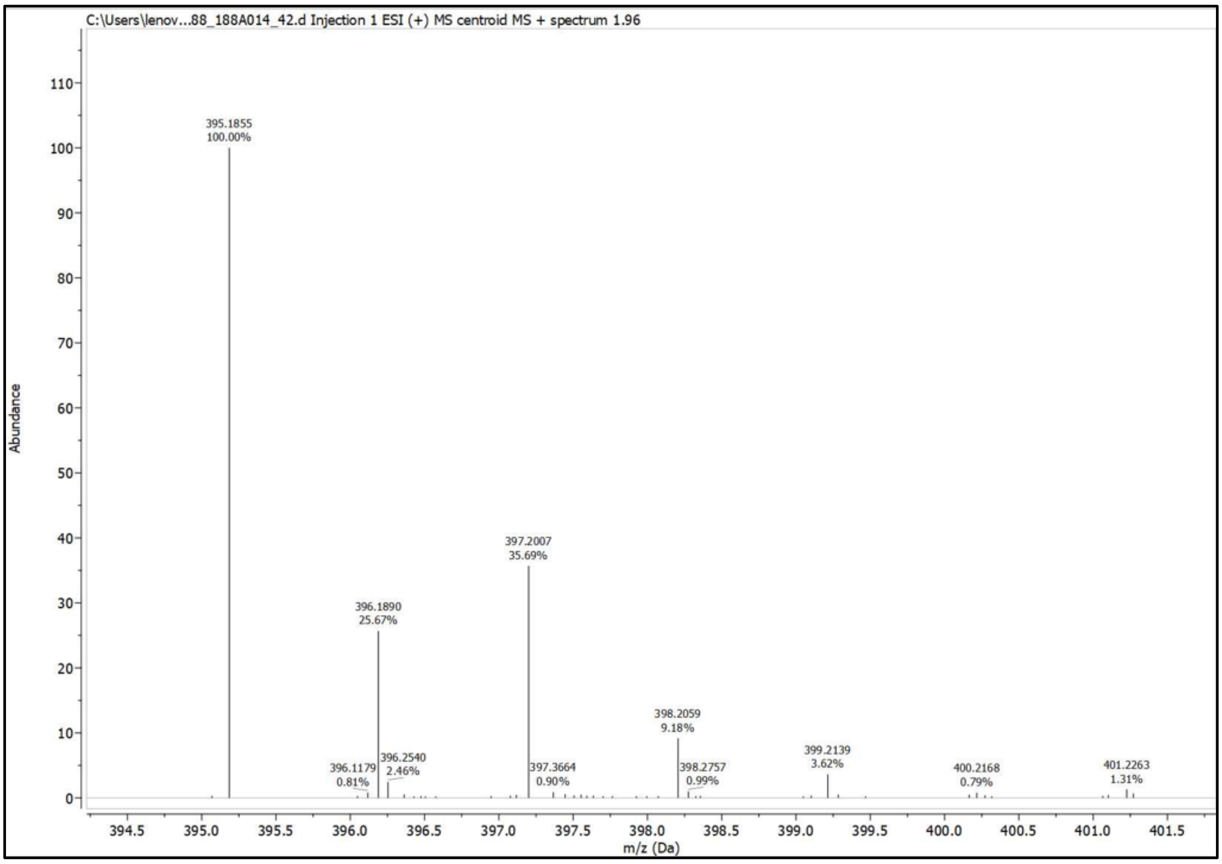

Figure S8: HRESIMS spectrum of druferone (**1**).

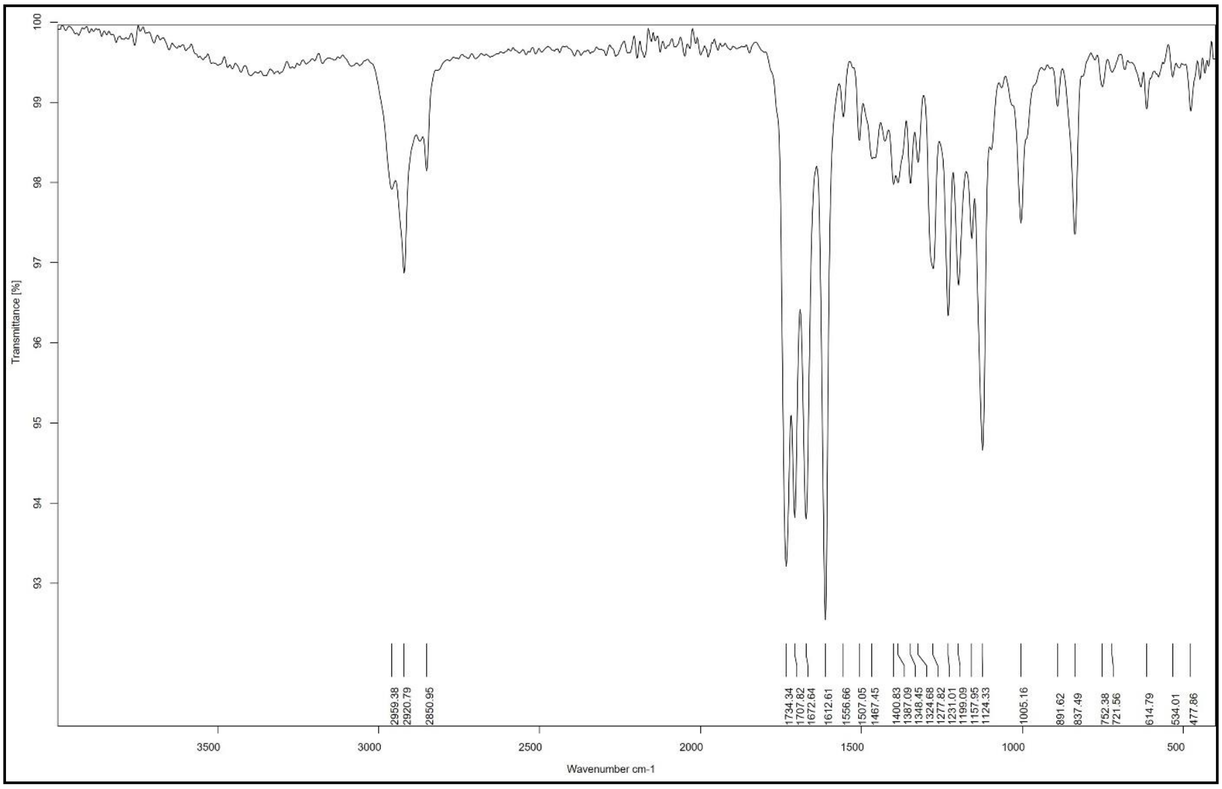

Figure S9: IR spectrum of druferone (**1**).

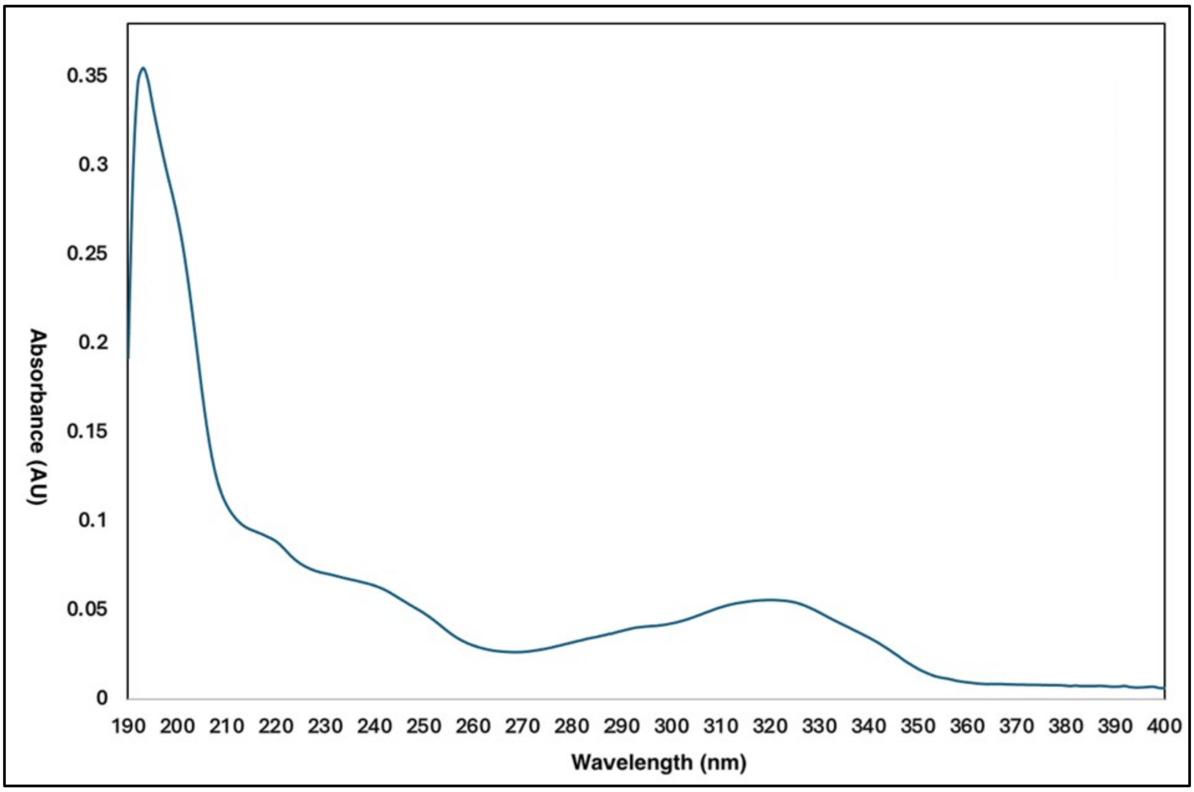

Figure S10: UV spectrum of druferone (**1**).

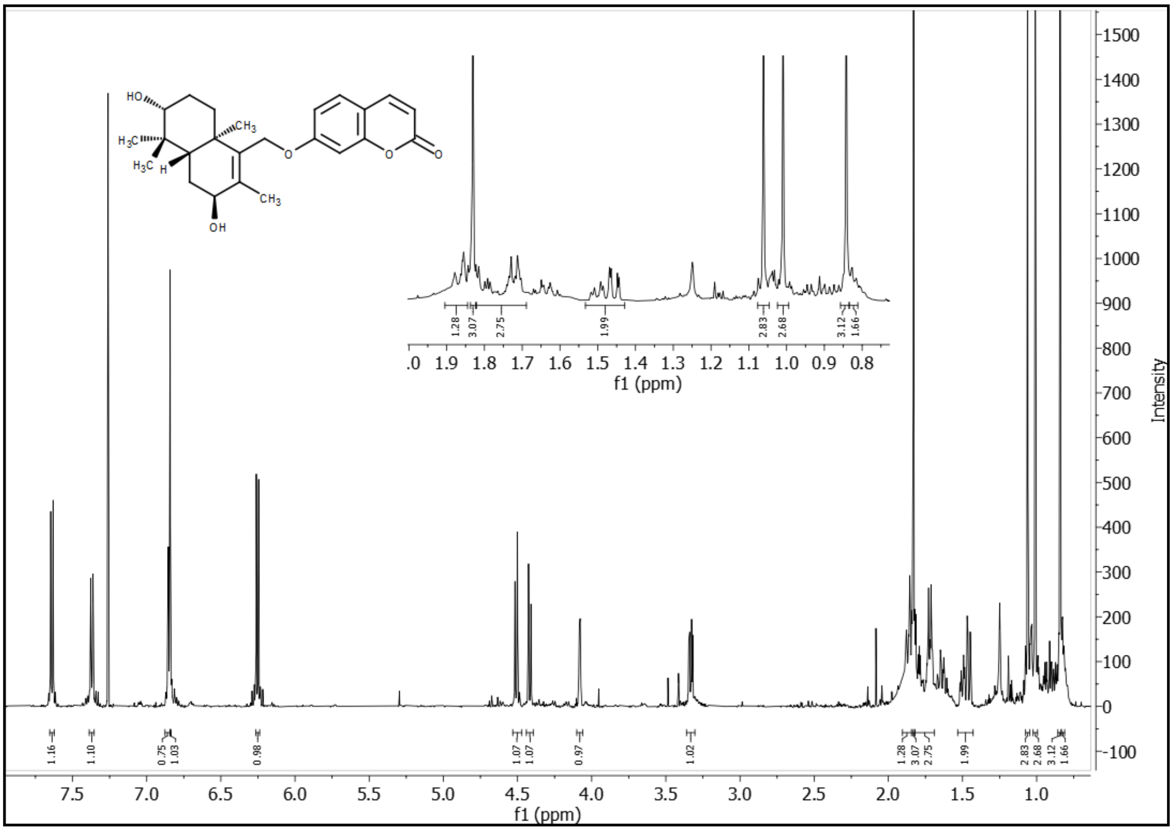

Figure S11: ^{1}H NMR spectrum (600 MHz, $CDCl_3$) of druferol (**2**).

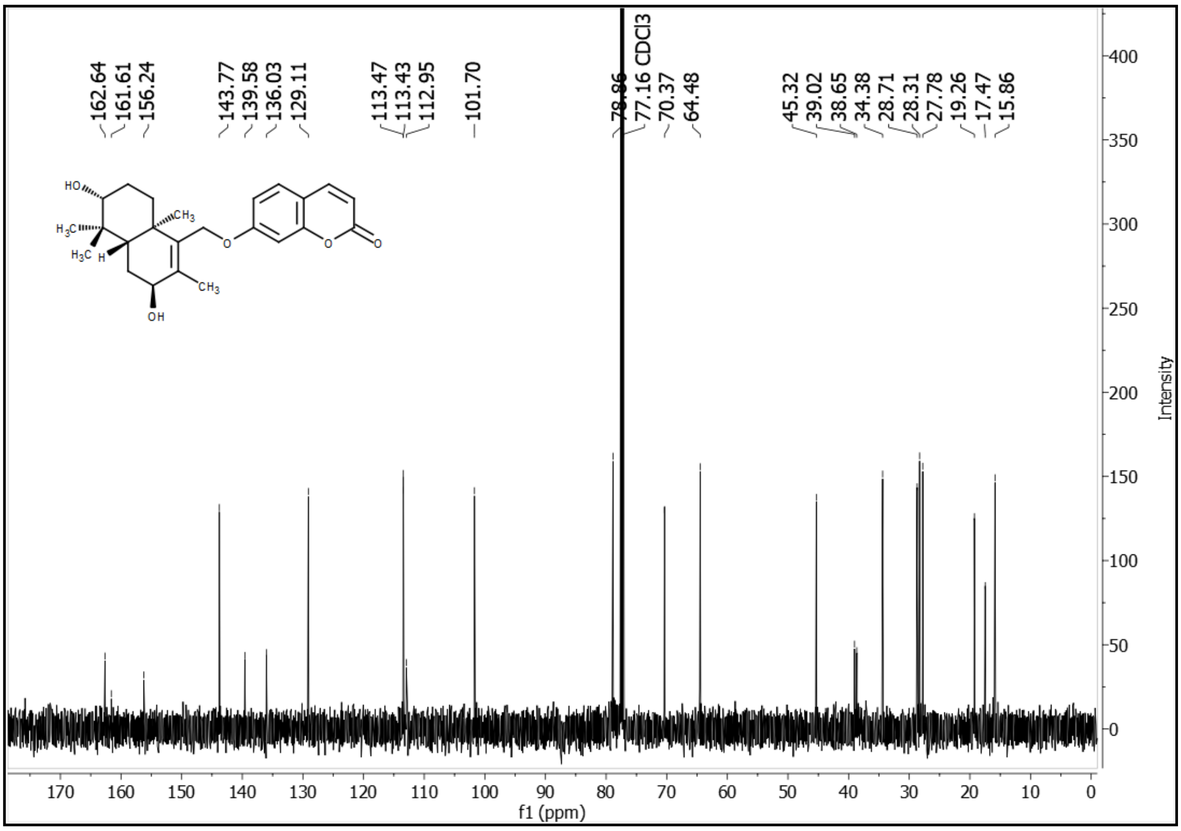

Figure S12: ^{13}C NMR spectrum (150 MHz, $CDCl_3$) of druferol (**2**).

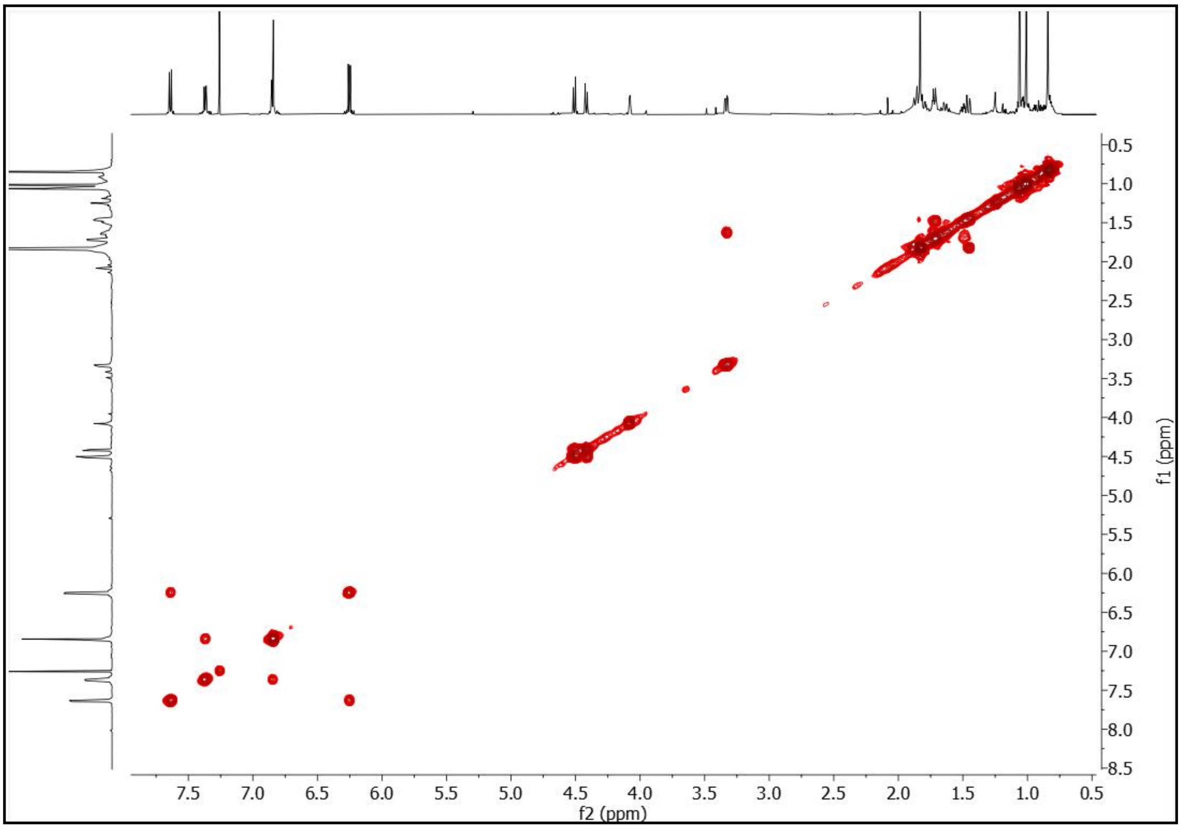

Figure S13: COSY spectrum of druferol (**2**).

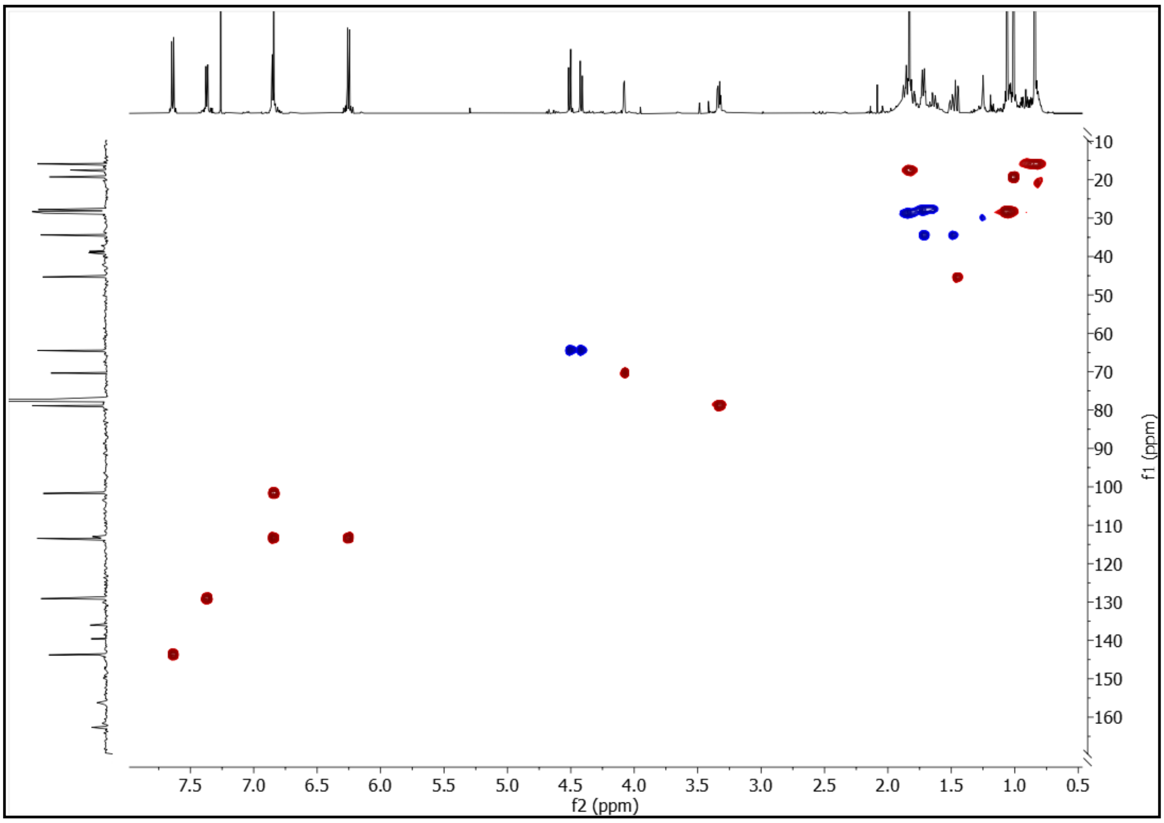

Figure S14: HSQC spectrum of druferol (**2**).

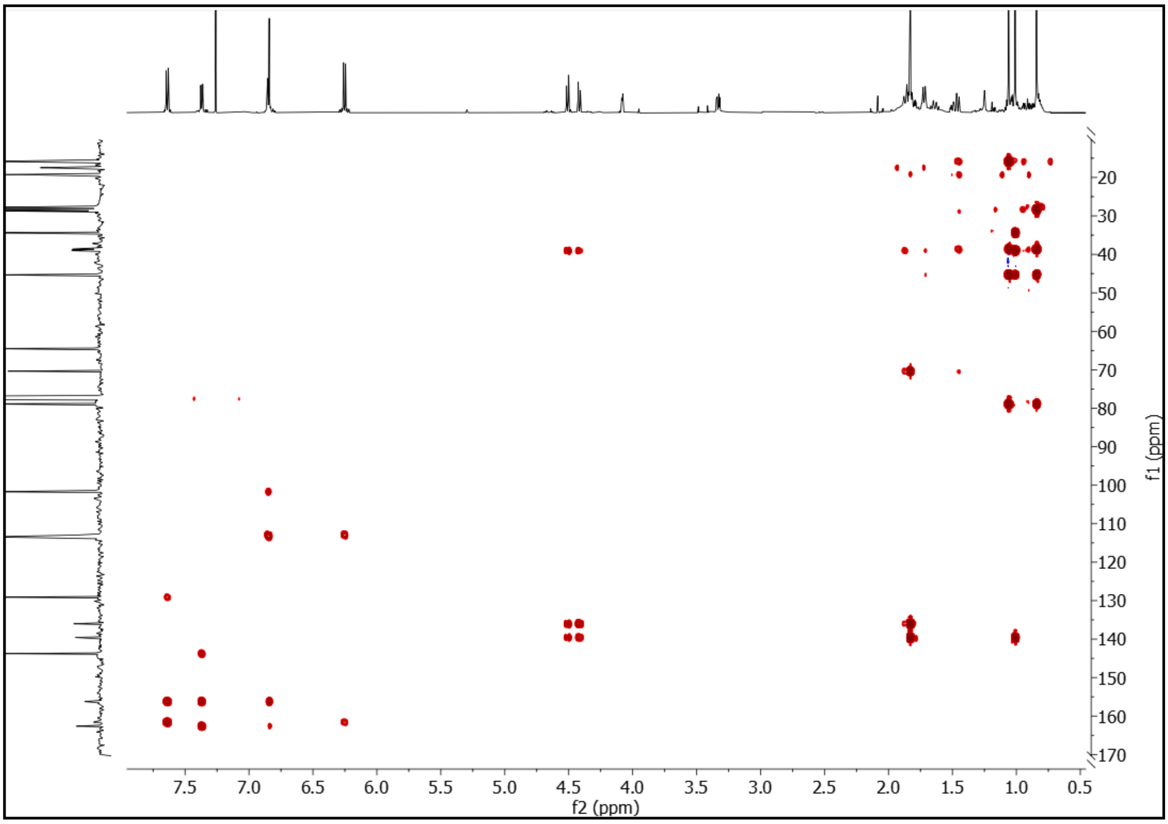

Figure S15: HMBC spectrum of druferol (**2**).

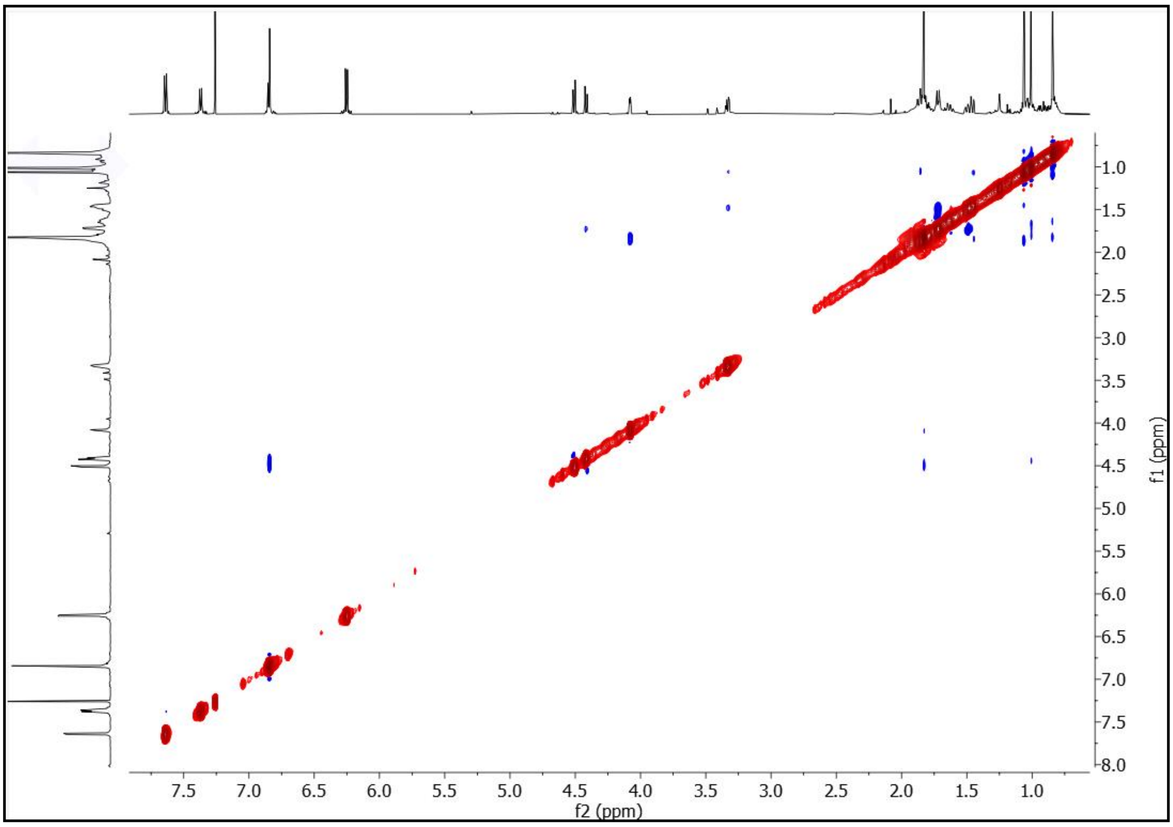

Figure S16: NOESY spectrum of druferol (**2**).

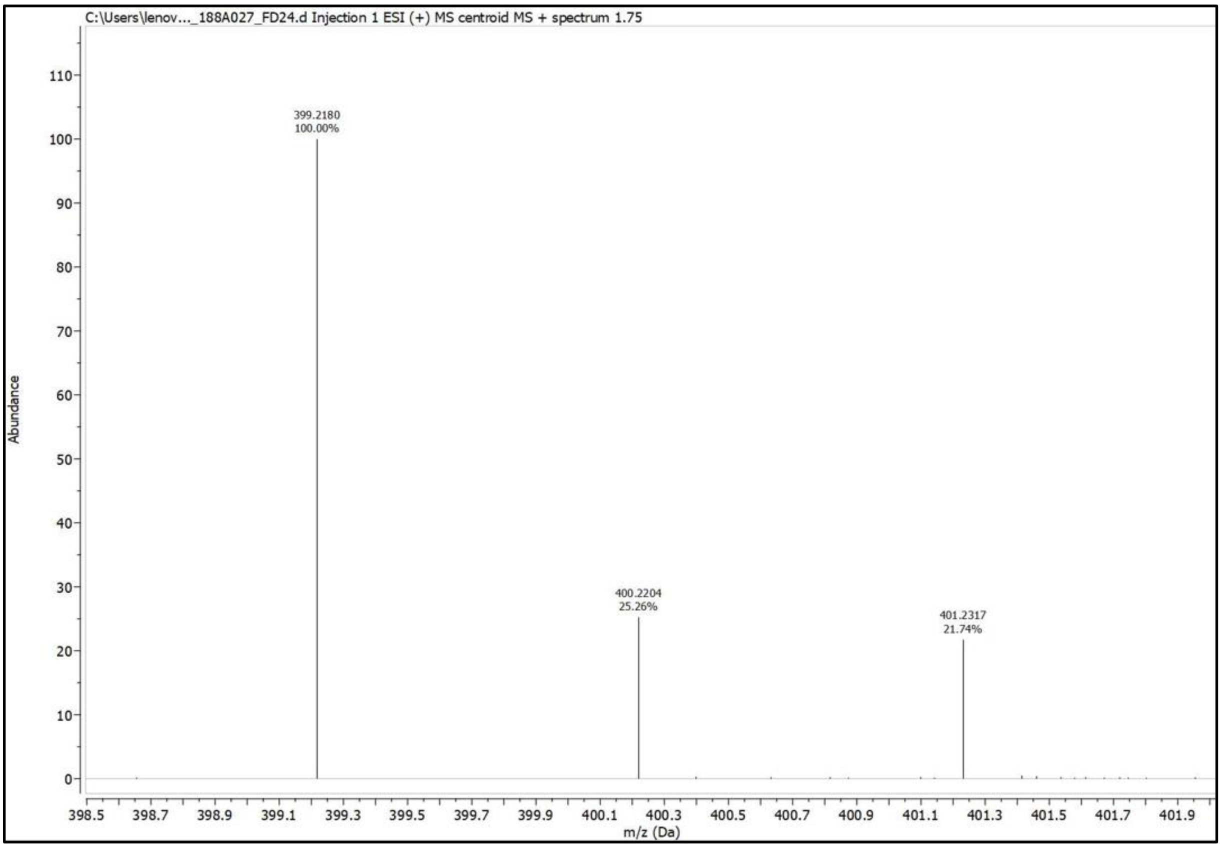

Figure S17: HRESIMS spectrum of druferol (**2**).

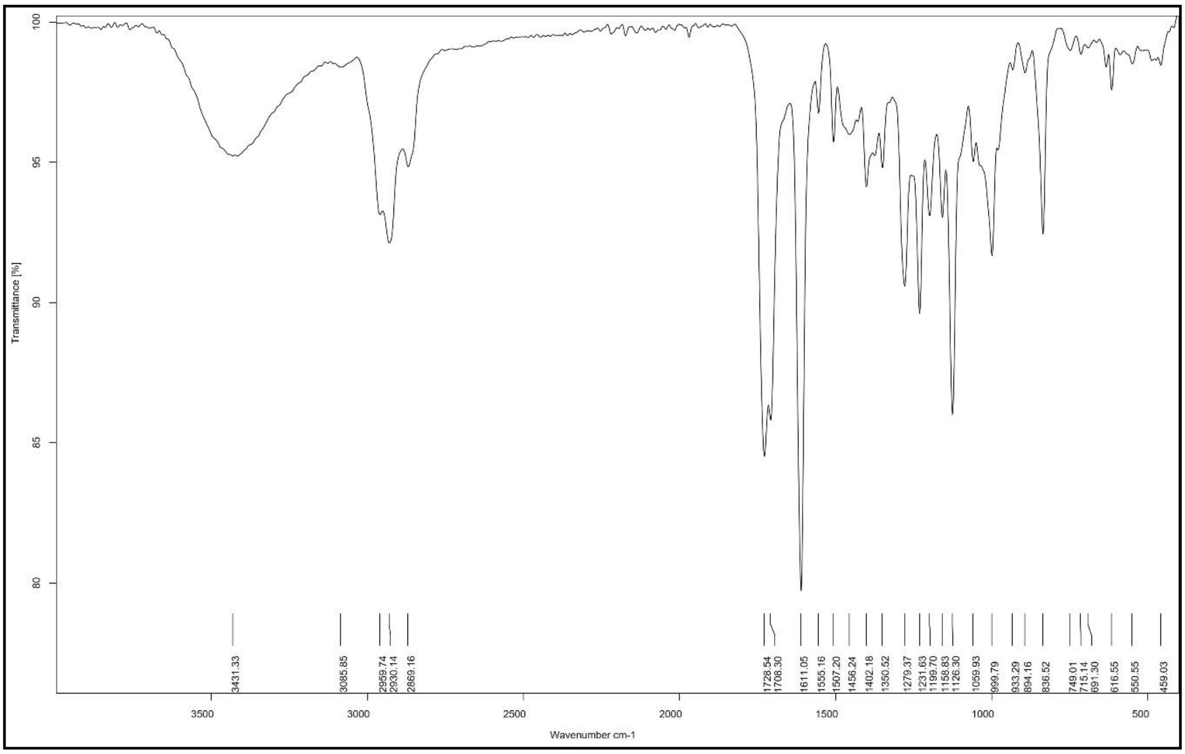

Figure S18: IR spectrum of druferol (**2**).

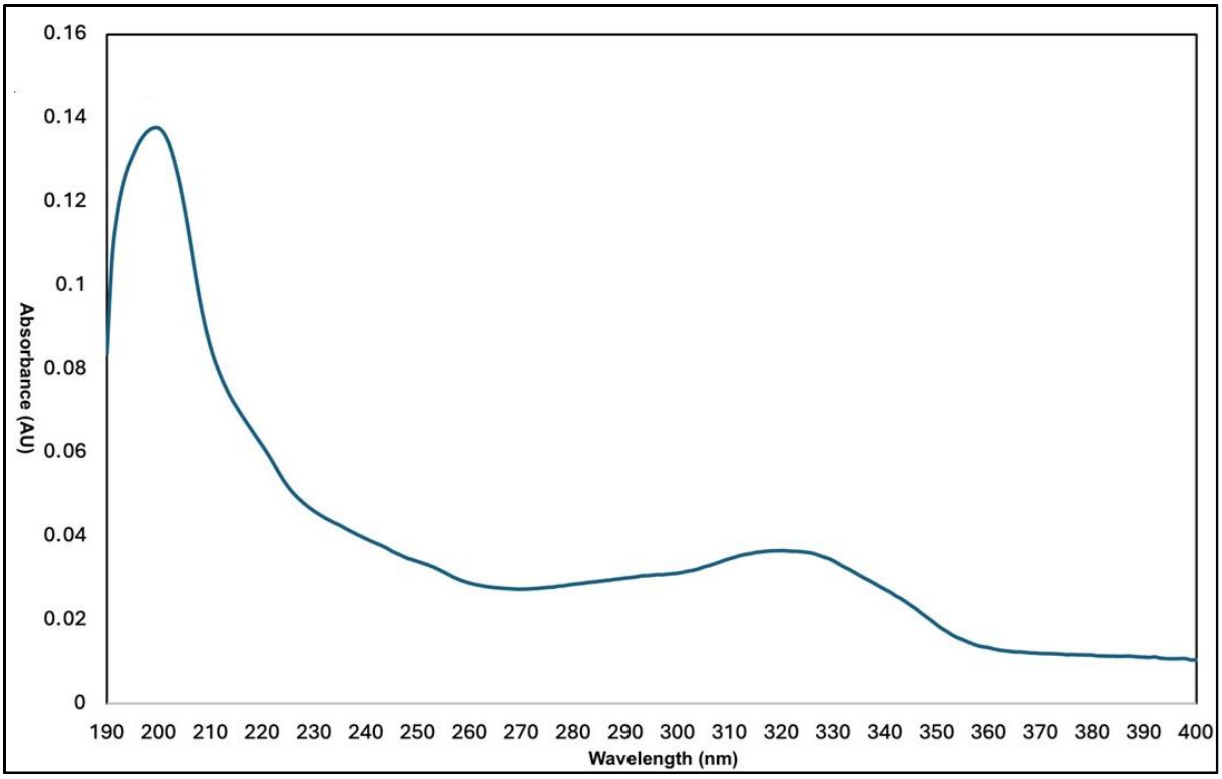

Figure S19: UV spectrum (MeOH) of druferol (**2**).

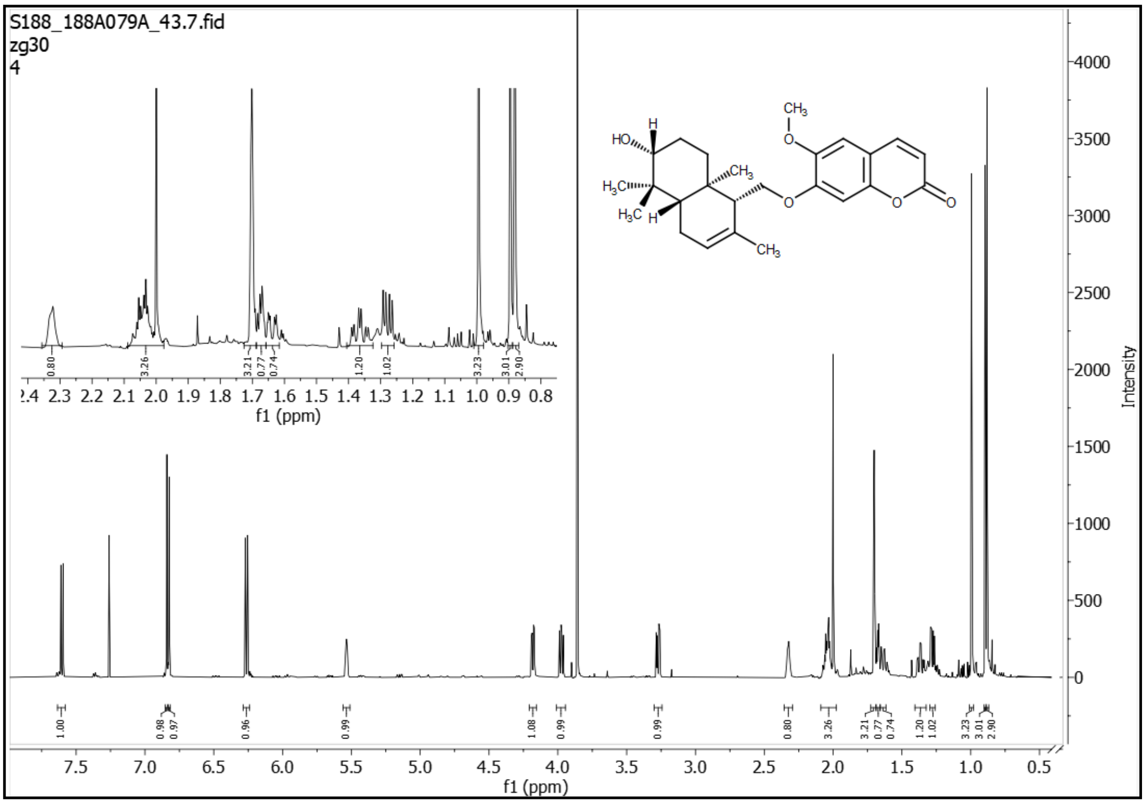

Figure S20: ^{1}H NMR spectrum (600 MHz, $CDCl_3$) of druscoferol (**3**).

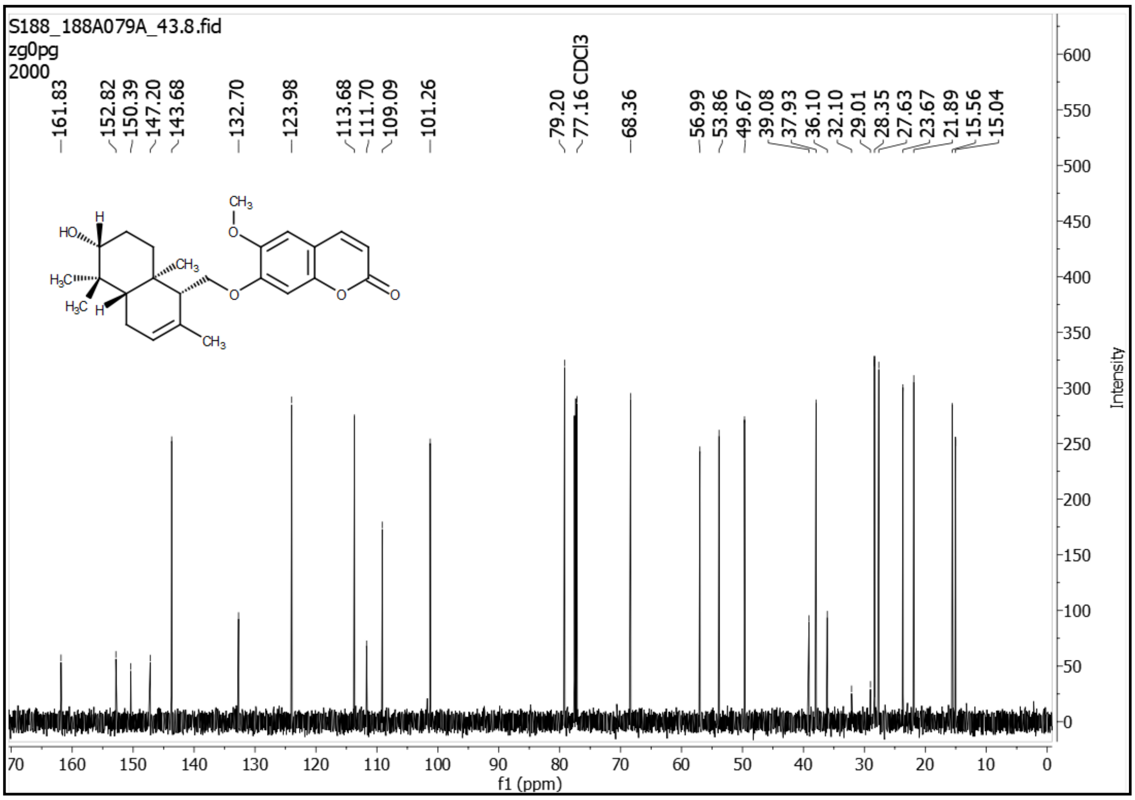

Figure S21: ^{13}C NMR spectrum (150 MHz, $CDCl_3$) of druscoferol (**3**).

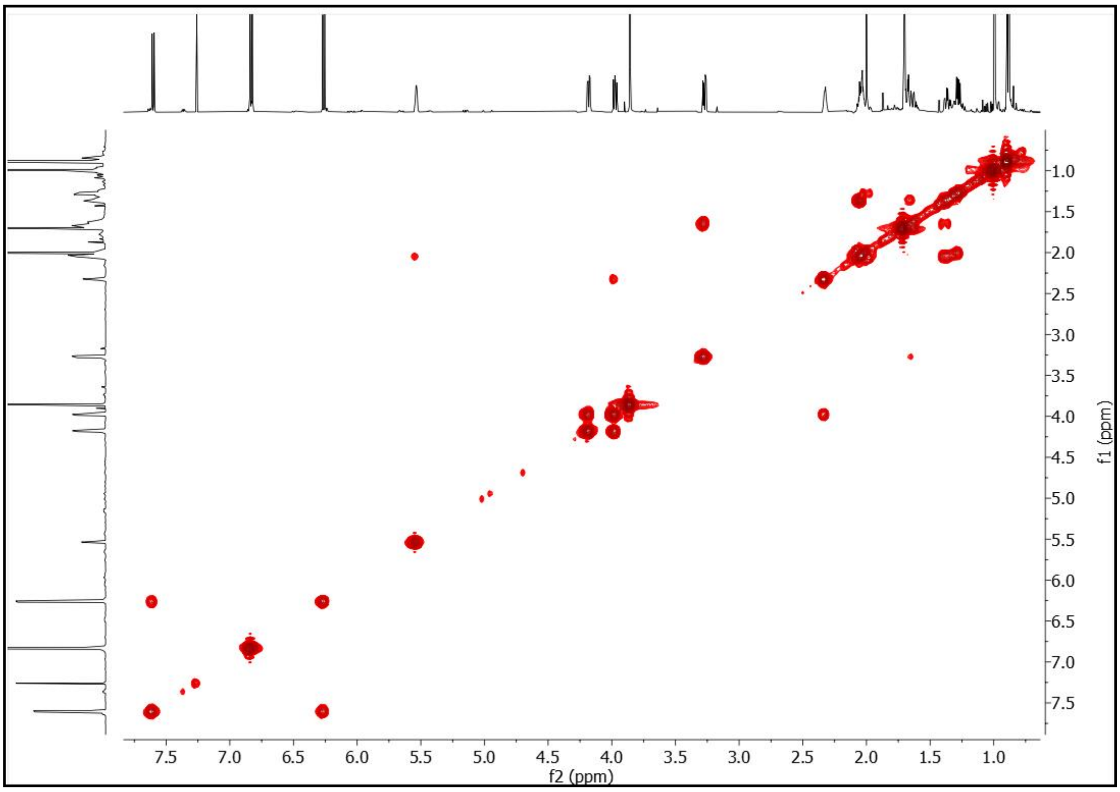

Figure S22: COSY spectrum of druscoferol (**3**).

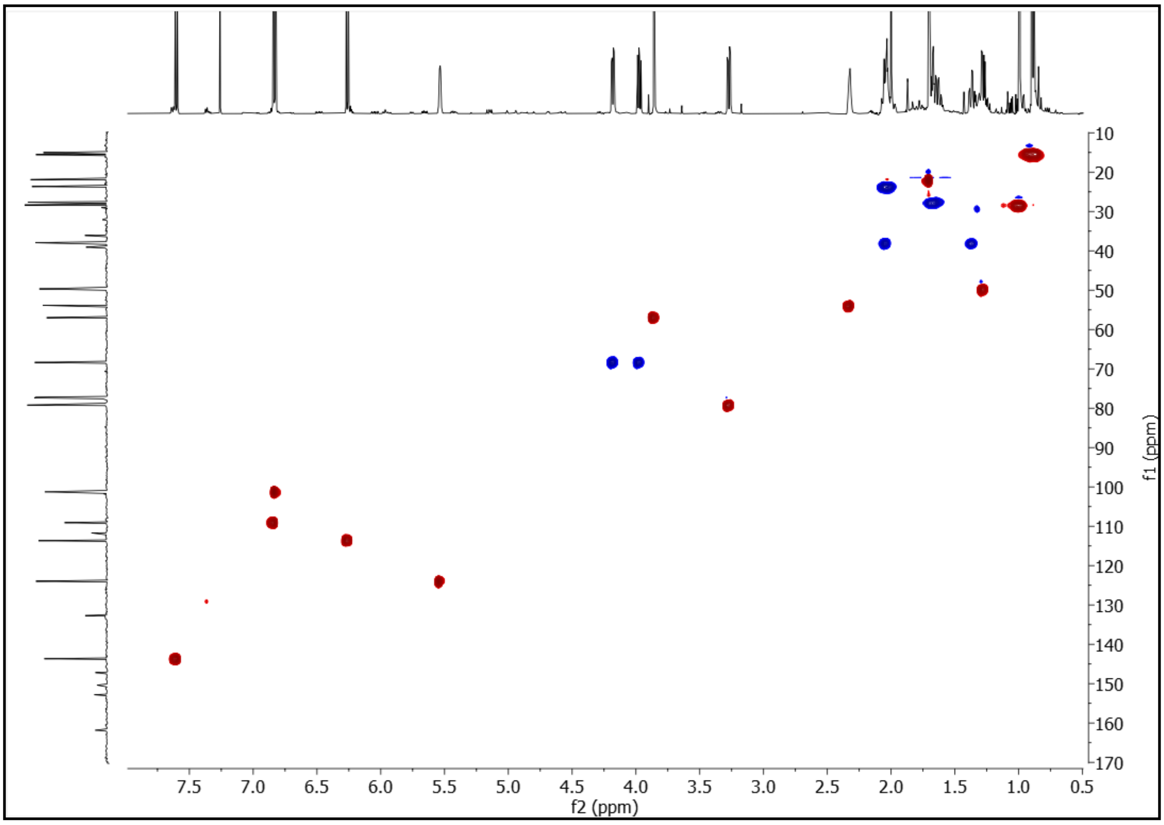

Figure S23: HSQC spectrum of druscoferol (**3**).

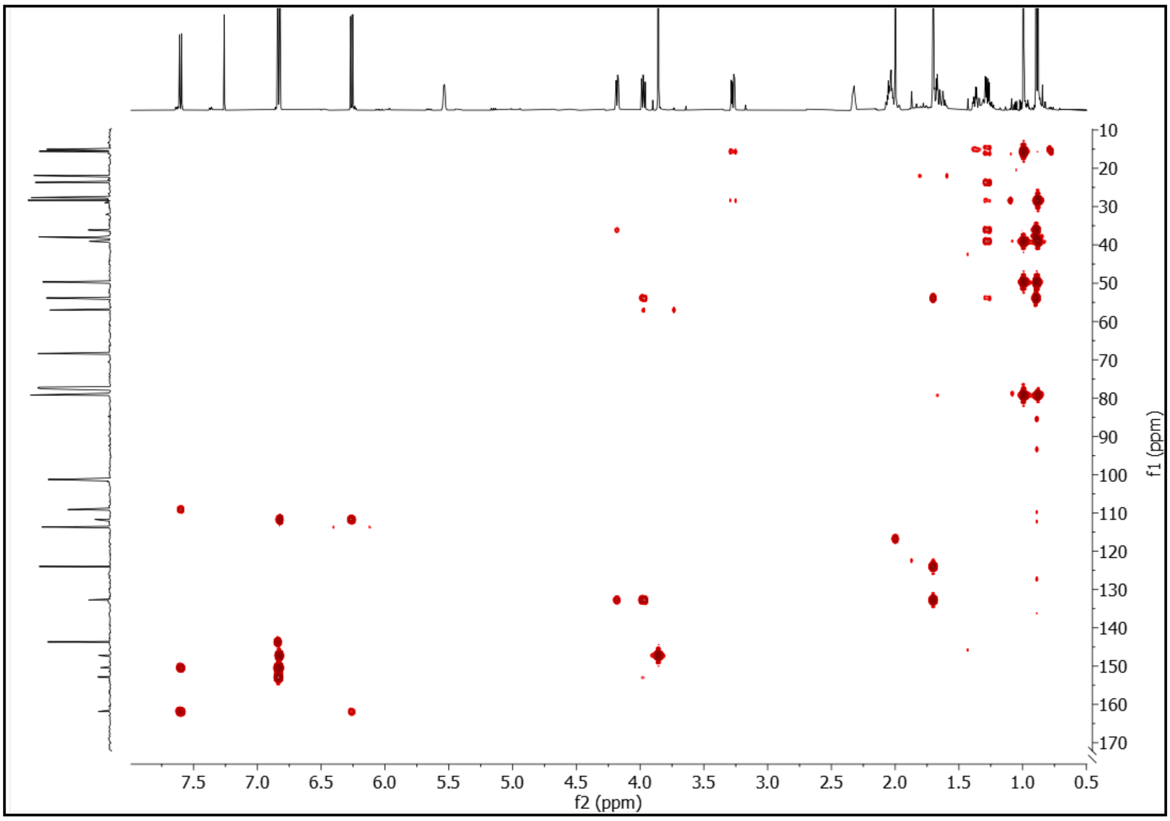

Figure S24: HMBC spectrum of druscoferol (**3**).

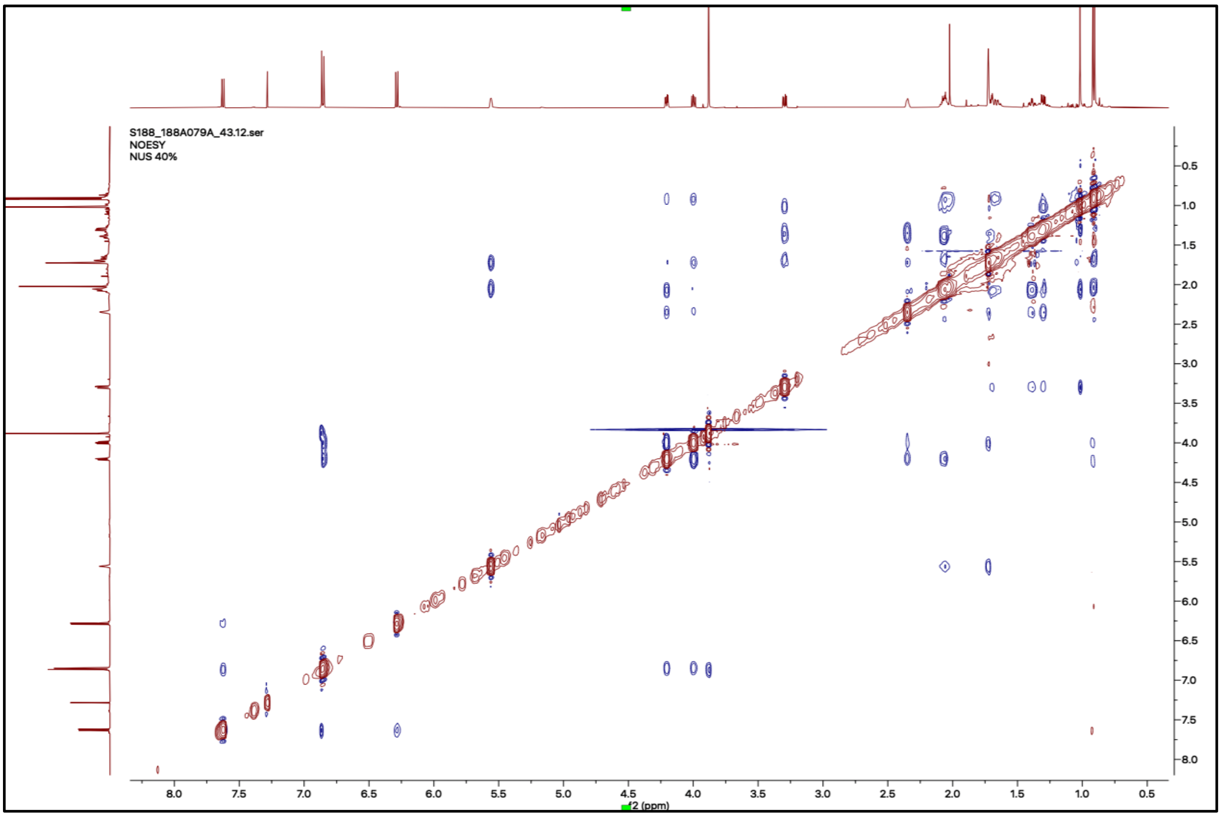

Figure S25: NOESY spectrum of druscoferol (**3**).

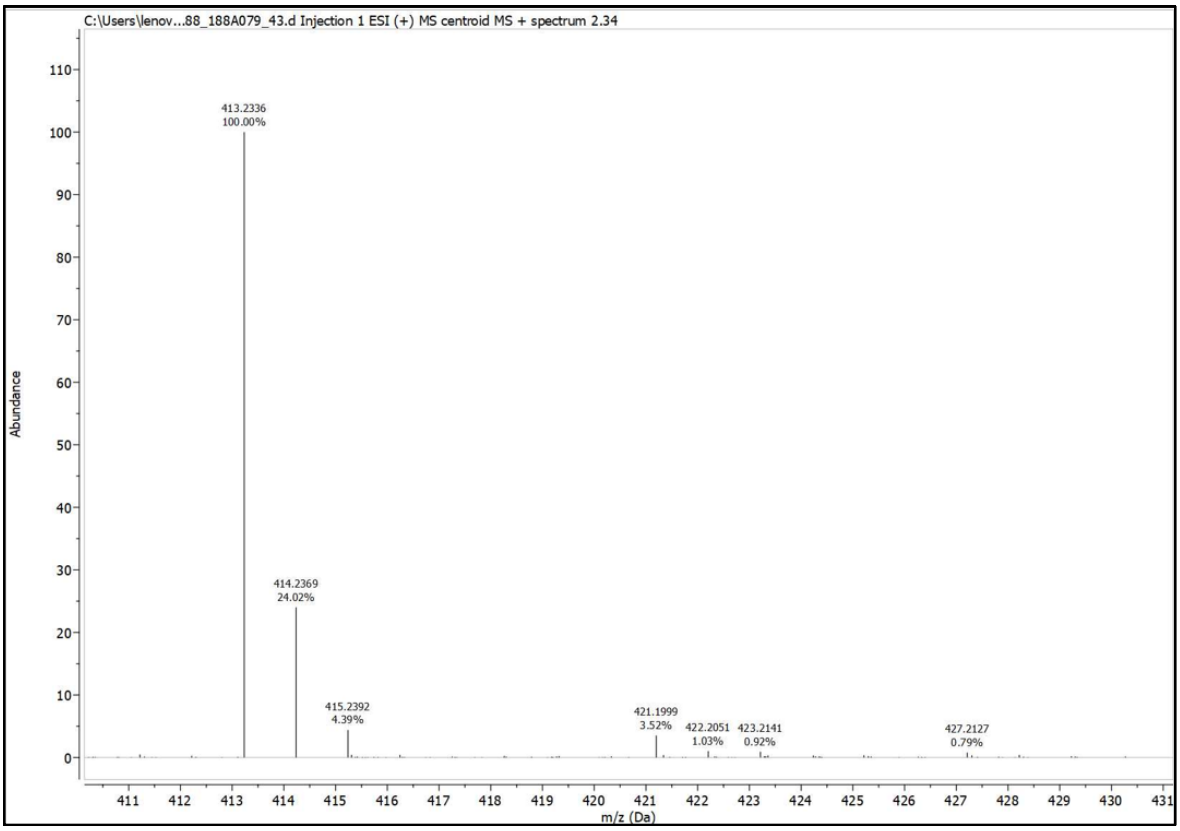

Figure S26: HRESIMS spectrum of druscoferol (**3**).

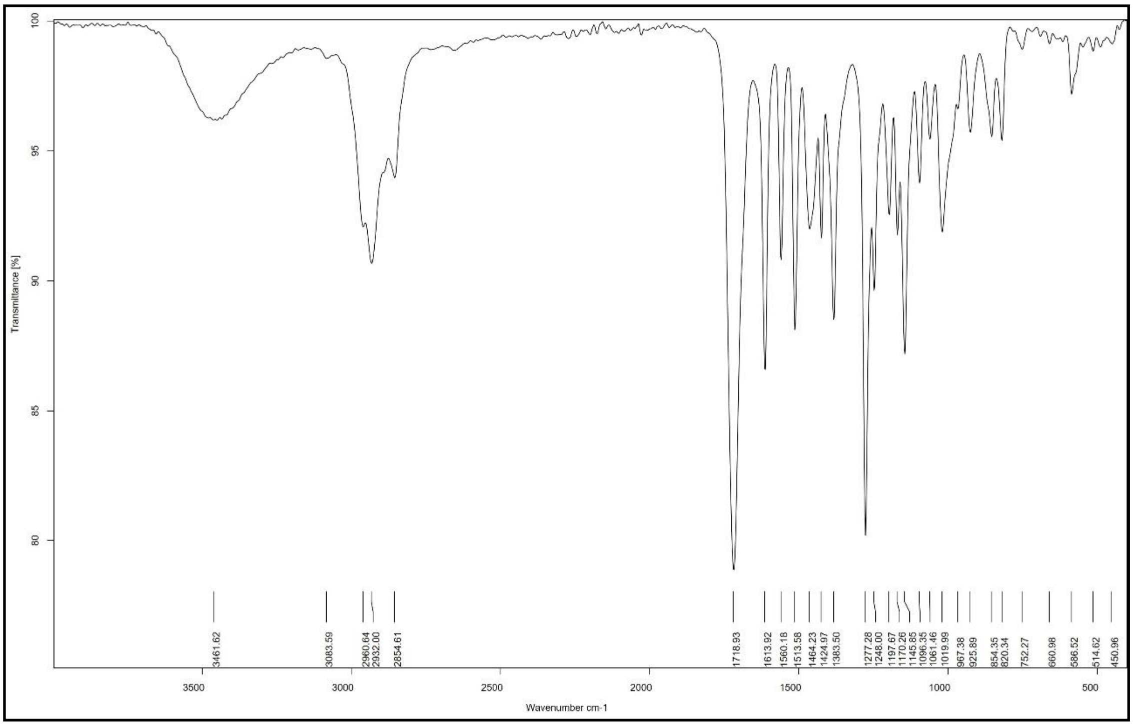

Figure S27: IR spectrum of druscoferol (**3**).

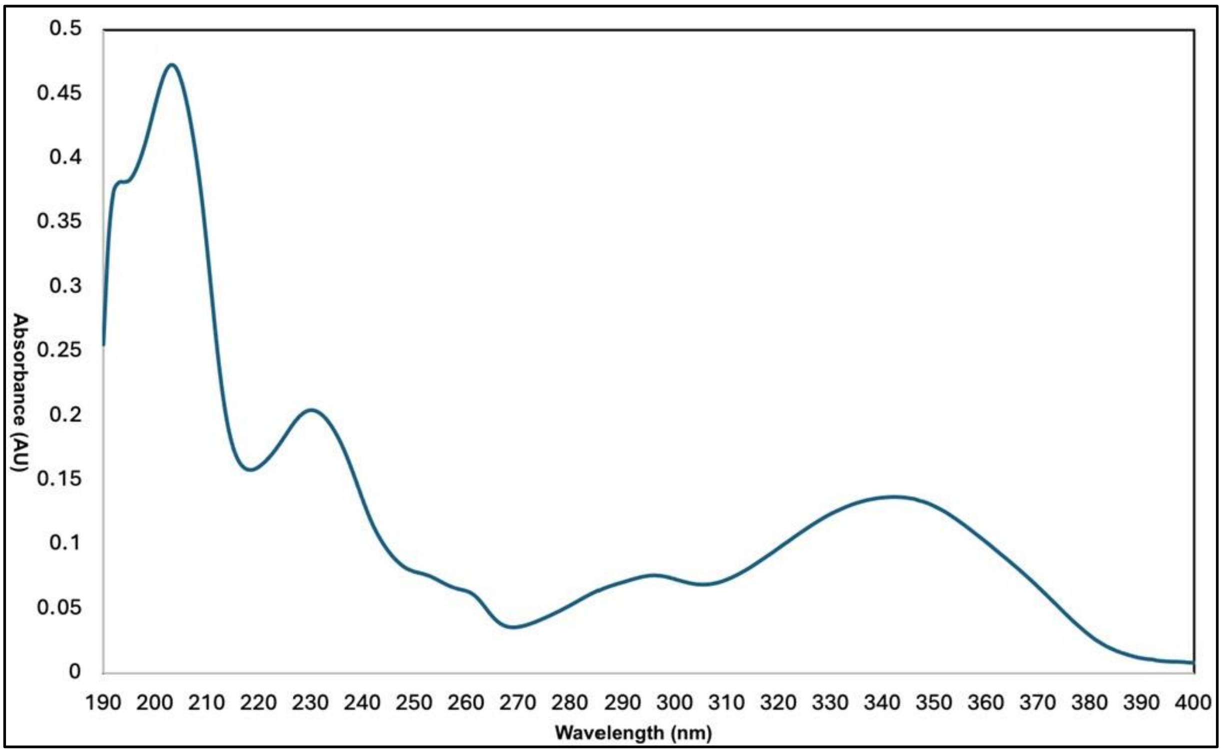

Figure S28: UV spectrum (MeOH) of druscoferol (**3**).

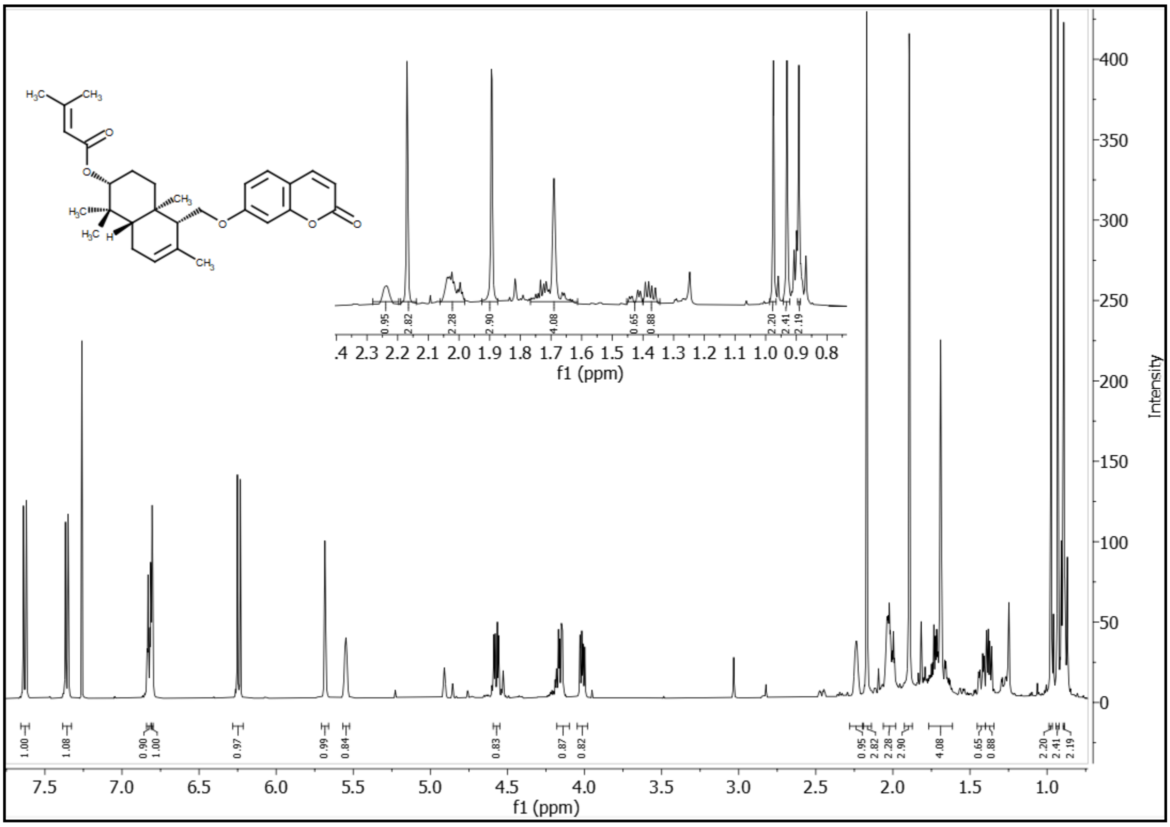

Figure S29: ^{1}H NMR spectrum (600 MHz, $CDCl_3$) of feselol senecioate (**4**).

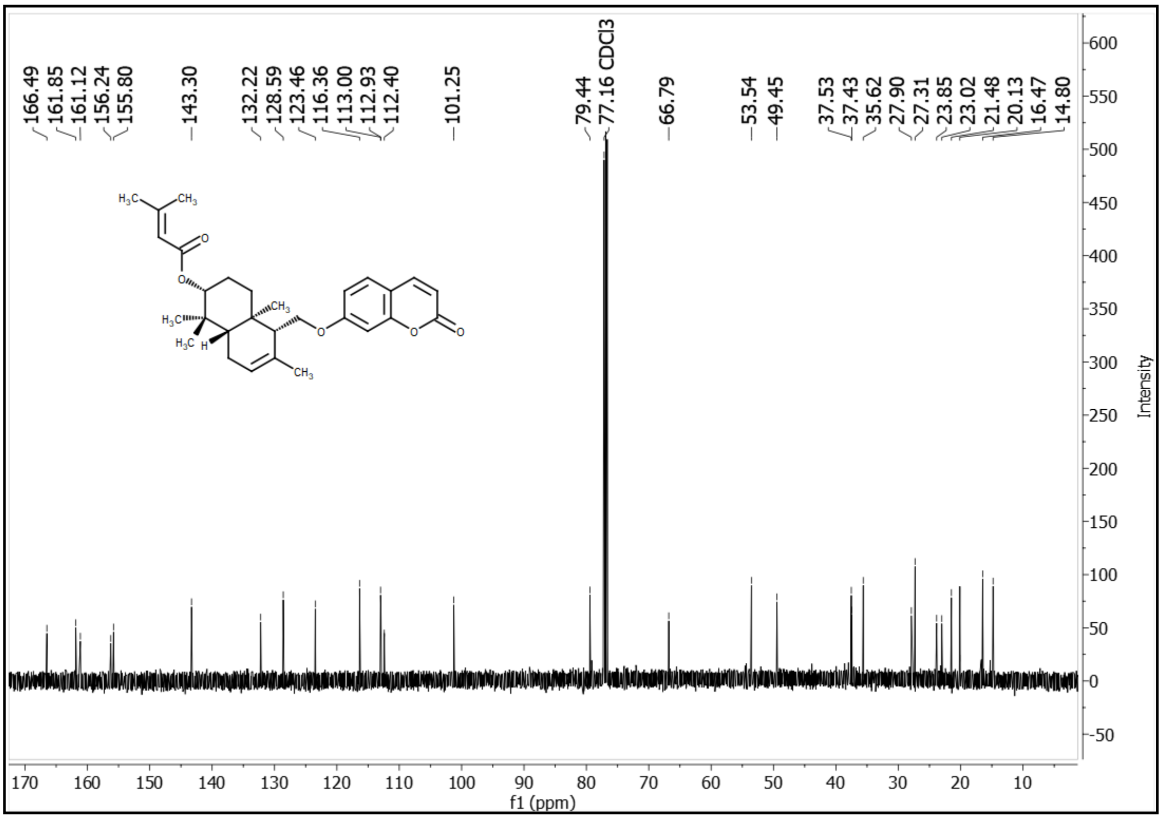

Figure S30: ^{13}C NMR spectrum (150 MHz, $CDCl_3$) of feselol senecioate (**4**).

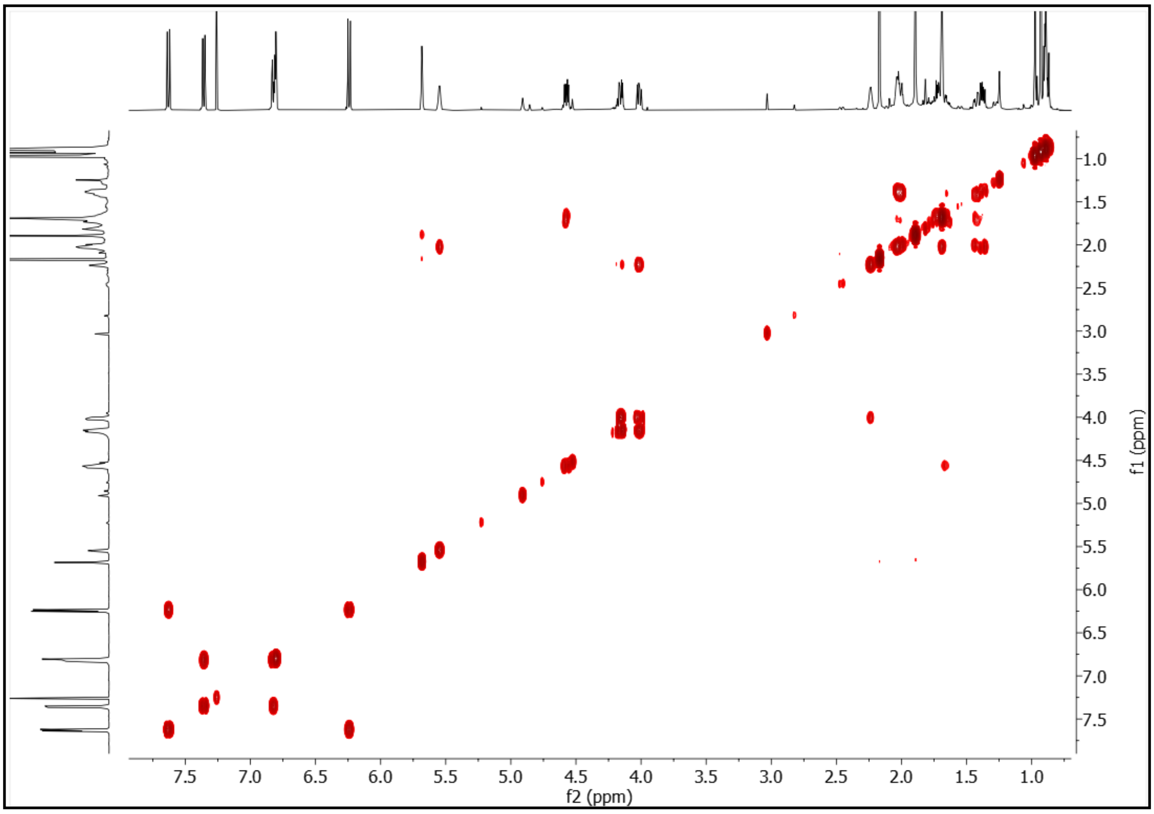

Figure S31: COSY spectrum of feselol senecioate (**4**).

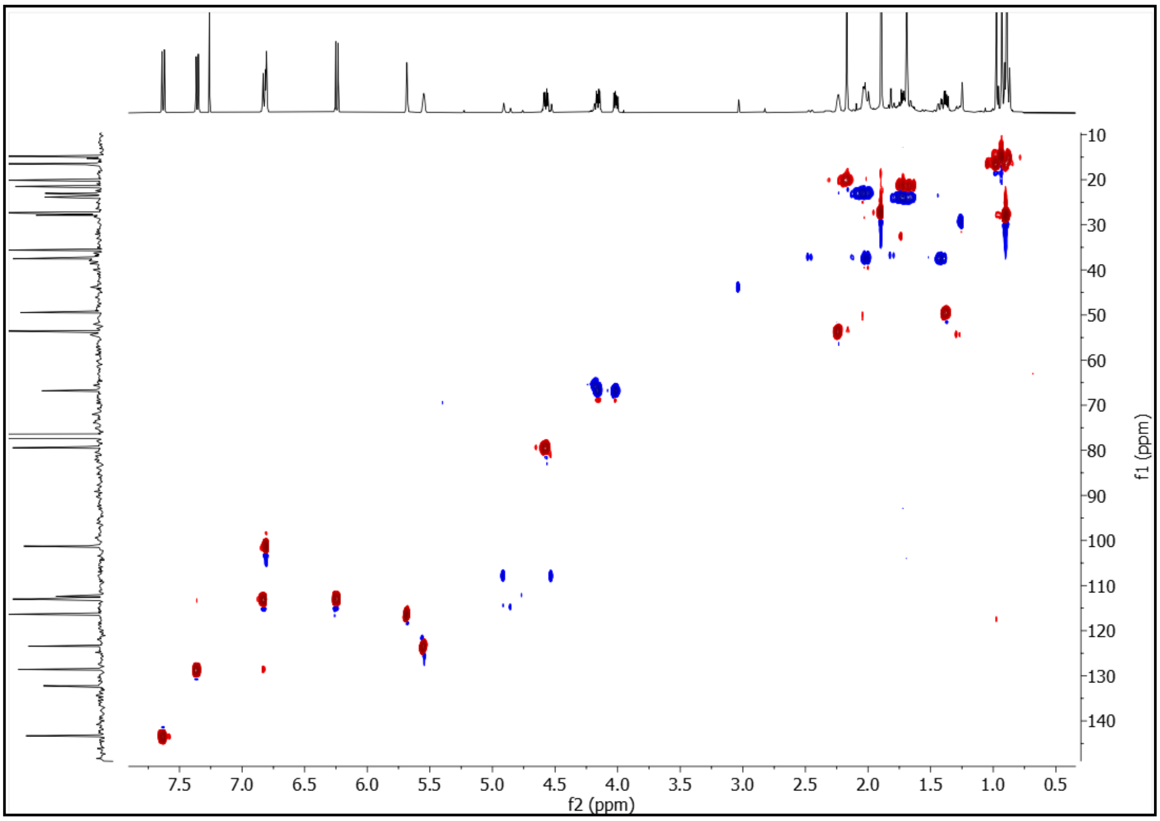

Figure S32: HSQC spectrum of feselol senecioate (**4**).

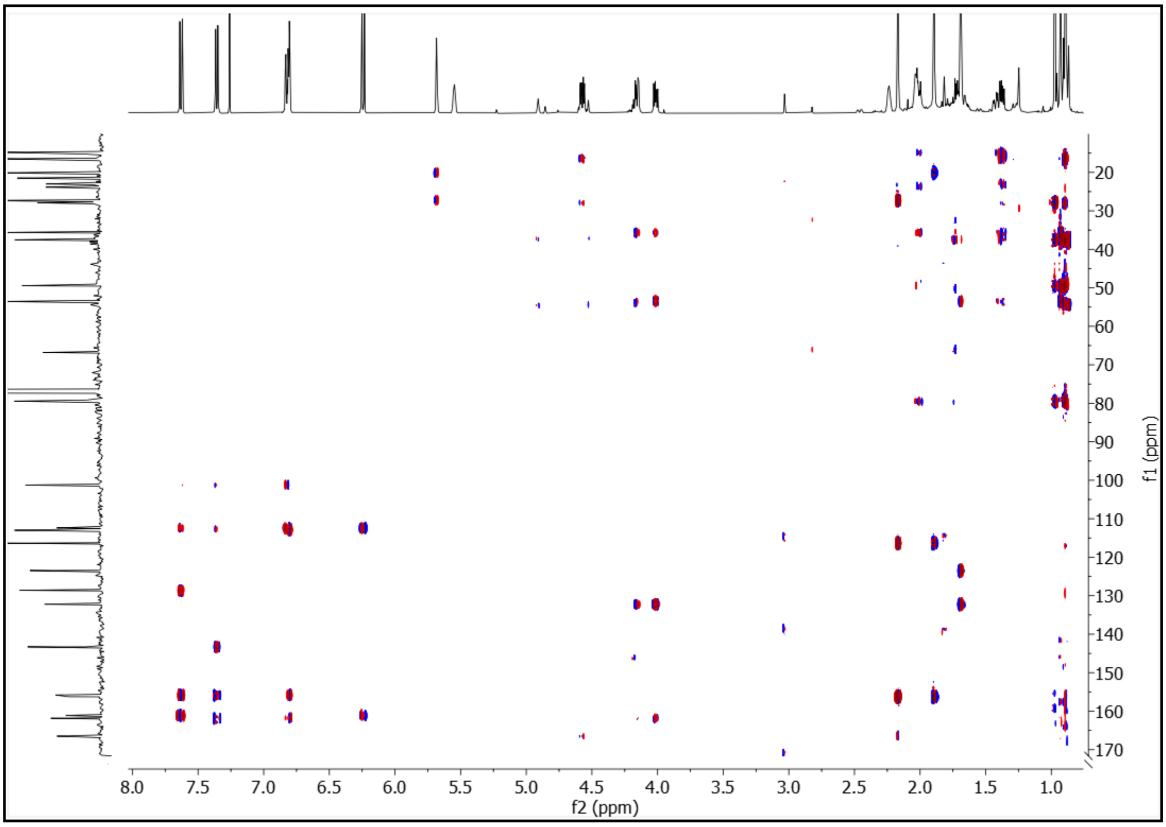

Figure S33: HMBC spectrum of feselol senecioate (**4**).

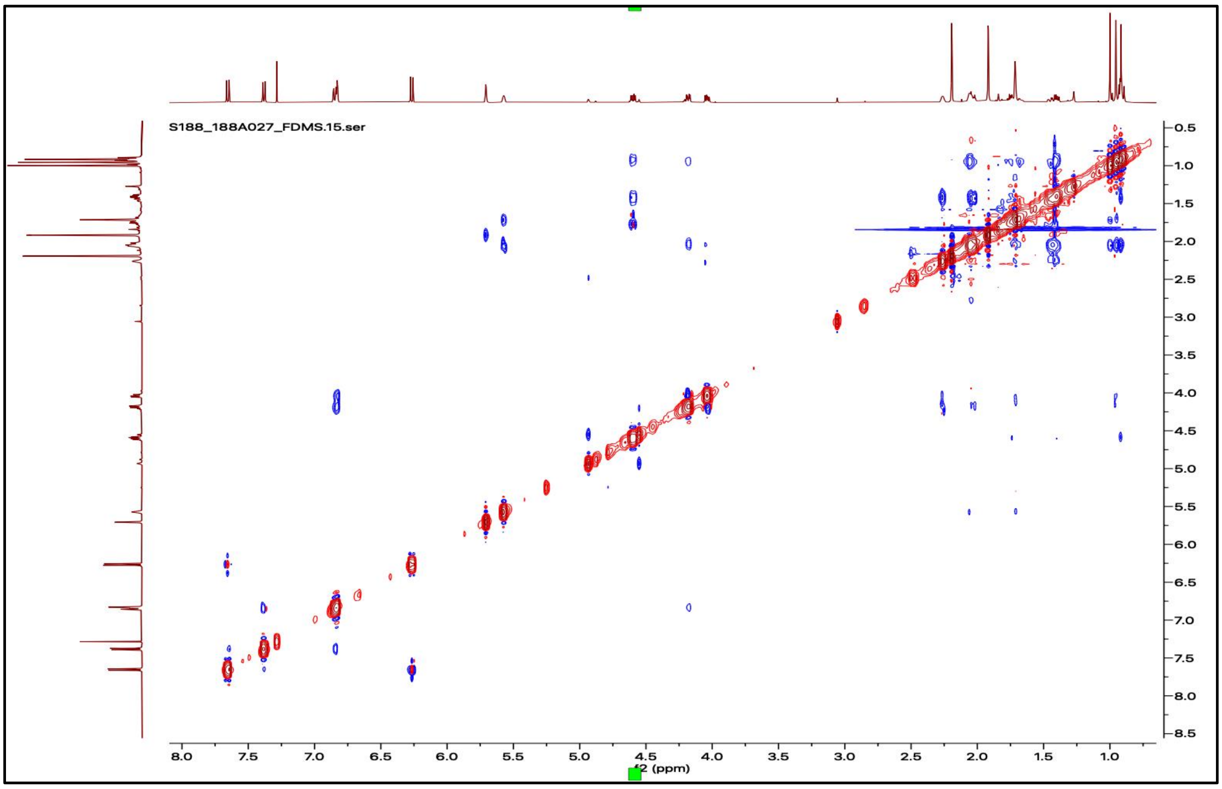

Figure S34: NOESY spectrum of feselol senecioate (**4**).

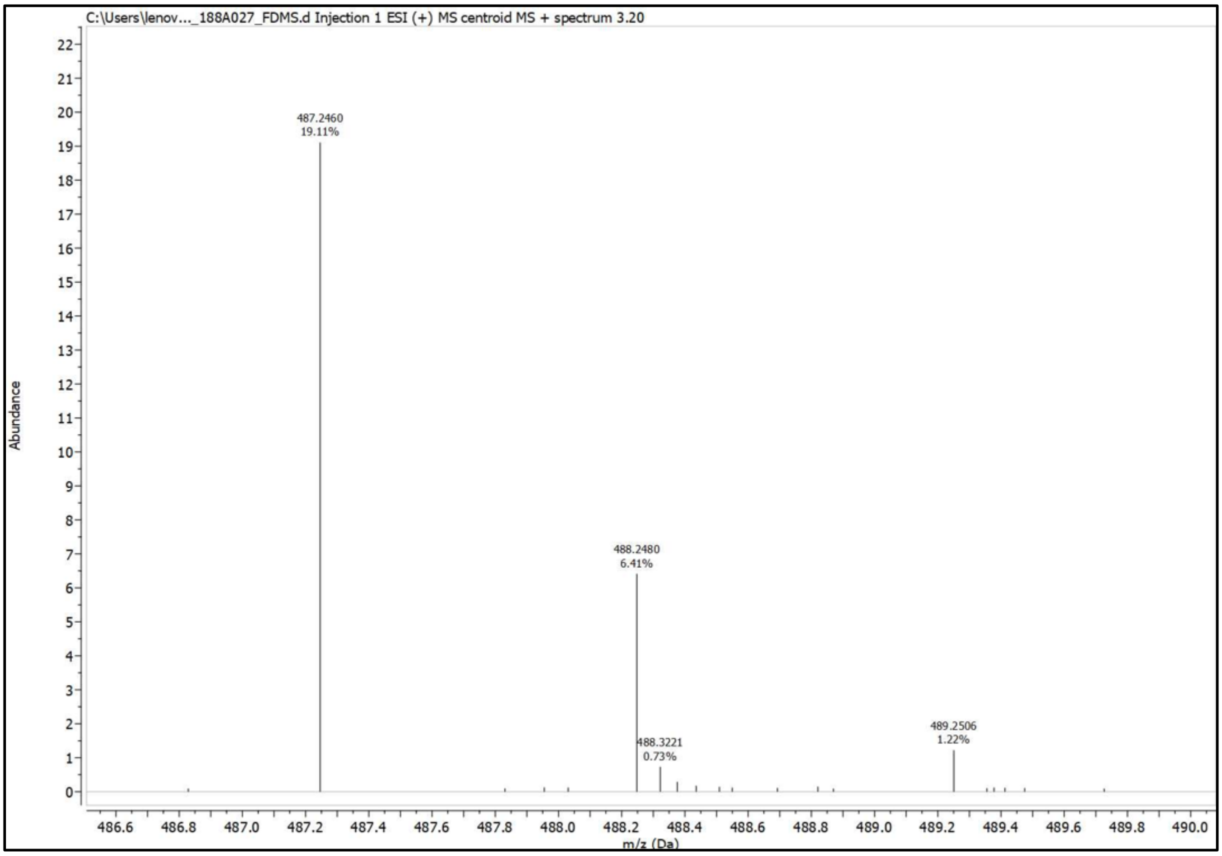

Figure S35: HRESIMS spectrum of feselol senecioate (**4**).

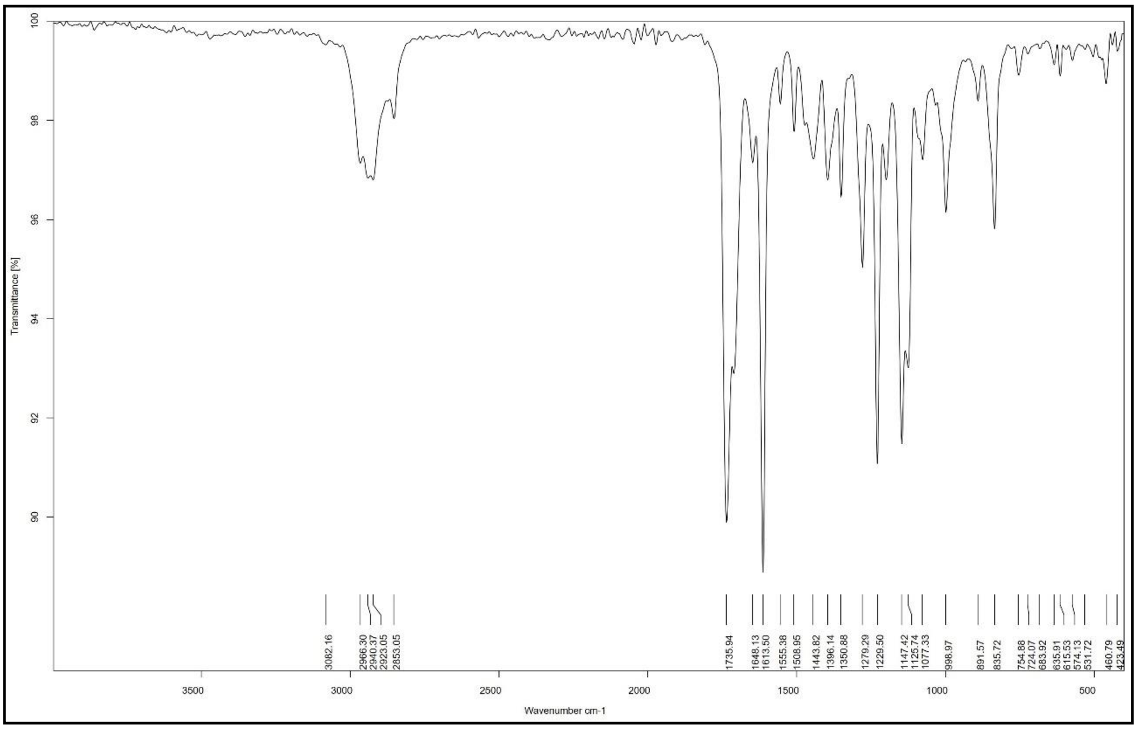

Figure S36: IR spectrum of feselol senecioate (**4**).

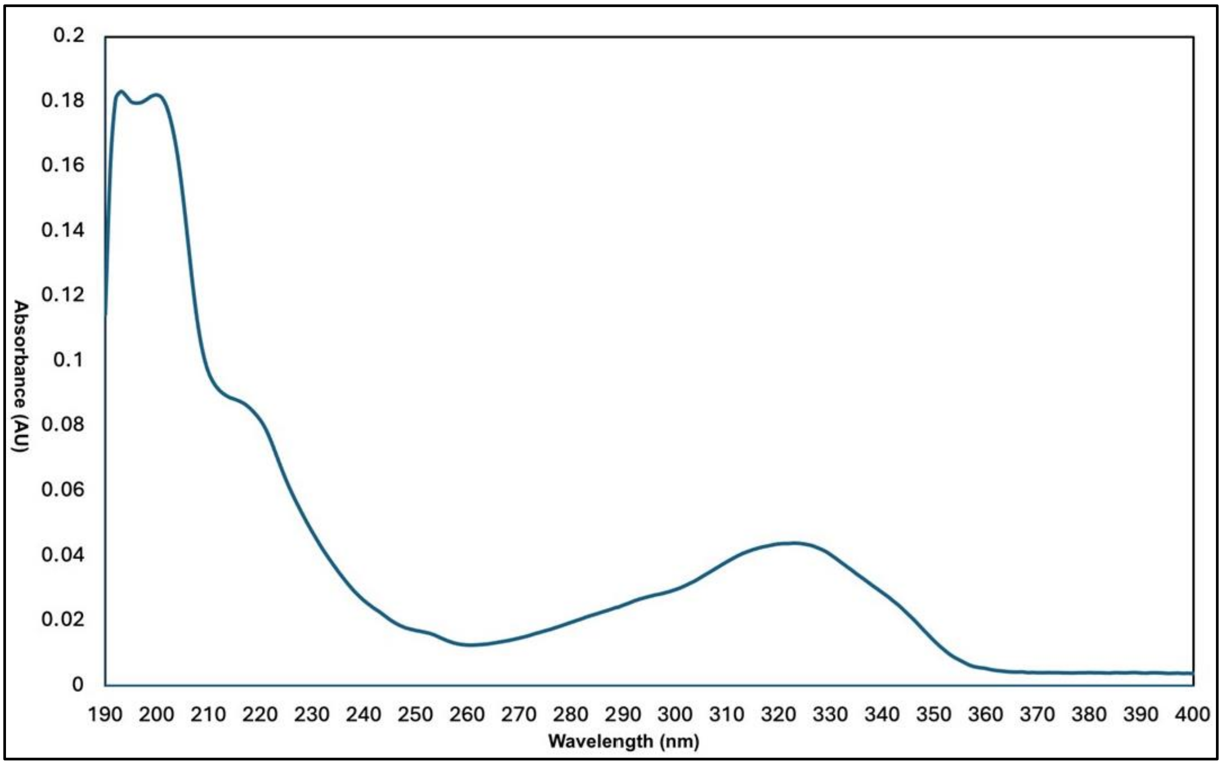

Figure S37: UV spectrum (MeOH) of feselol senecioate (**4**).

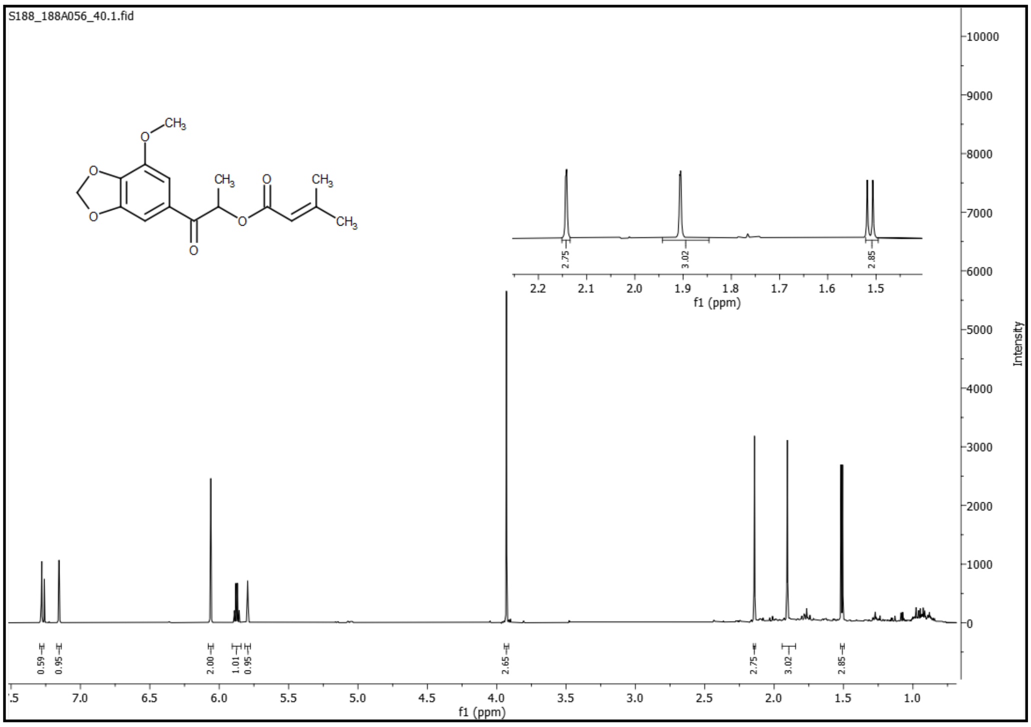

Figure S38: ^{1}H NMR spectrum (600 MHz, $CDCl_3$) of drudeanone (**5**).

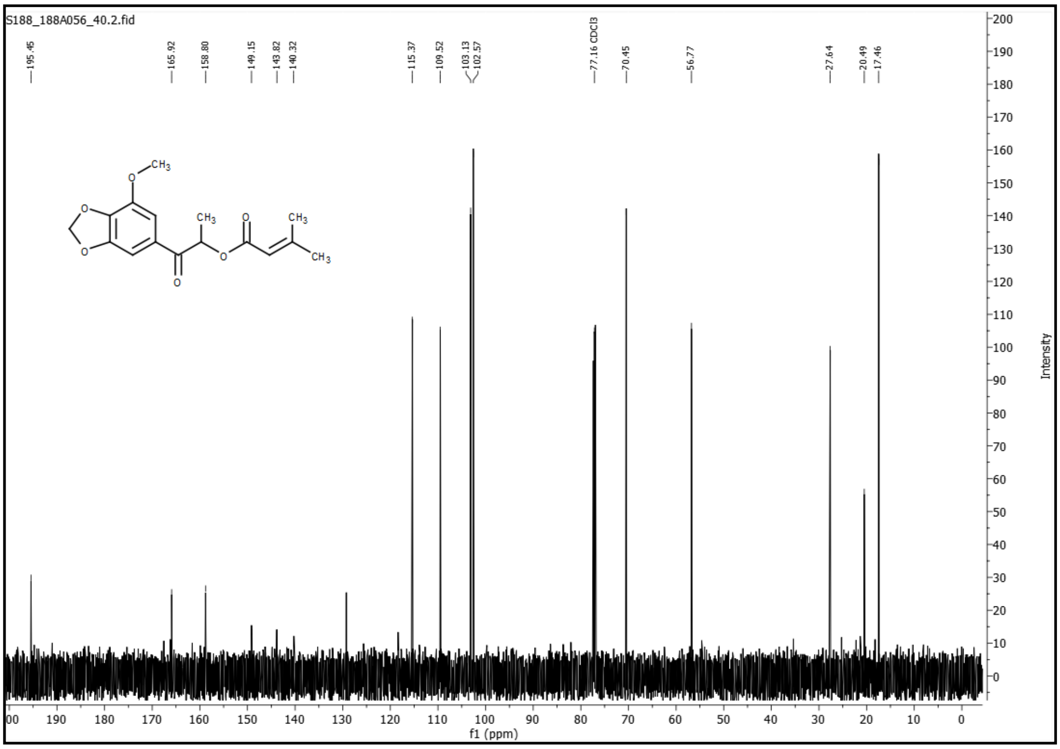

Figure S39: ^{13}C NMR spectrum (150 MHz, $CDCl_3$) of drudeanone (**5**).

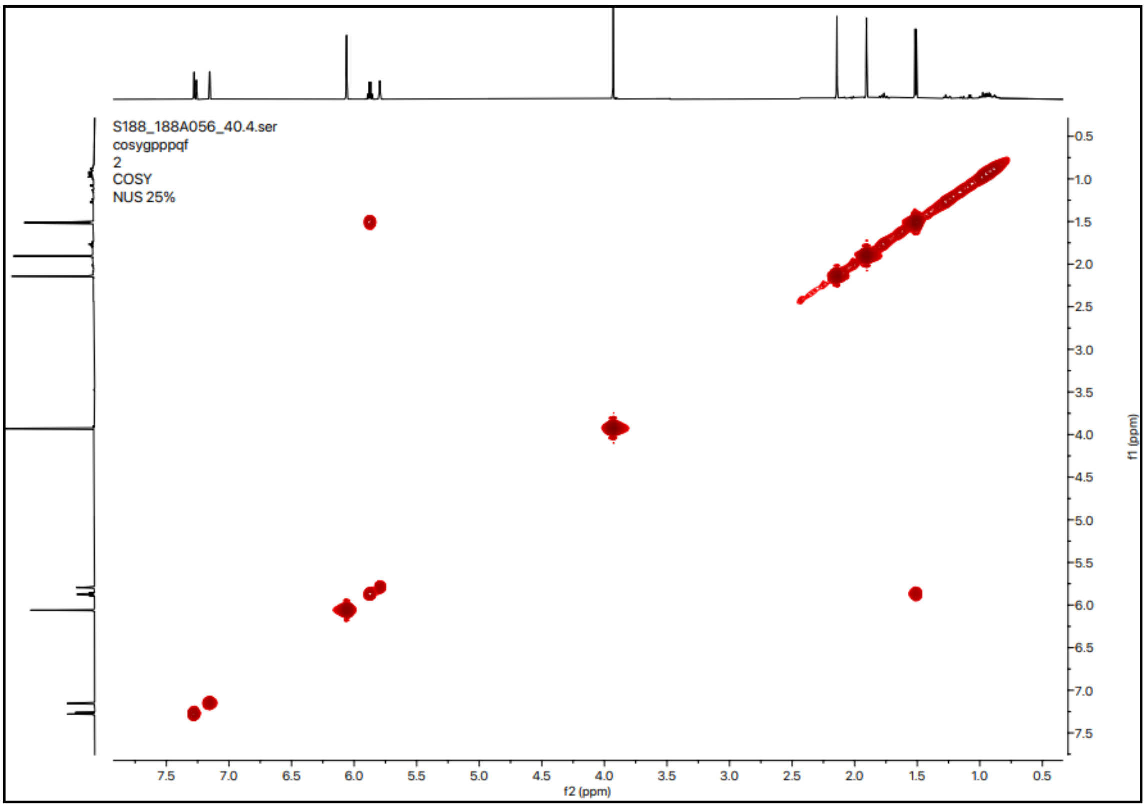

Figure S40: COSY spectrum of drudeanone (**5**).

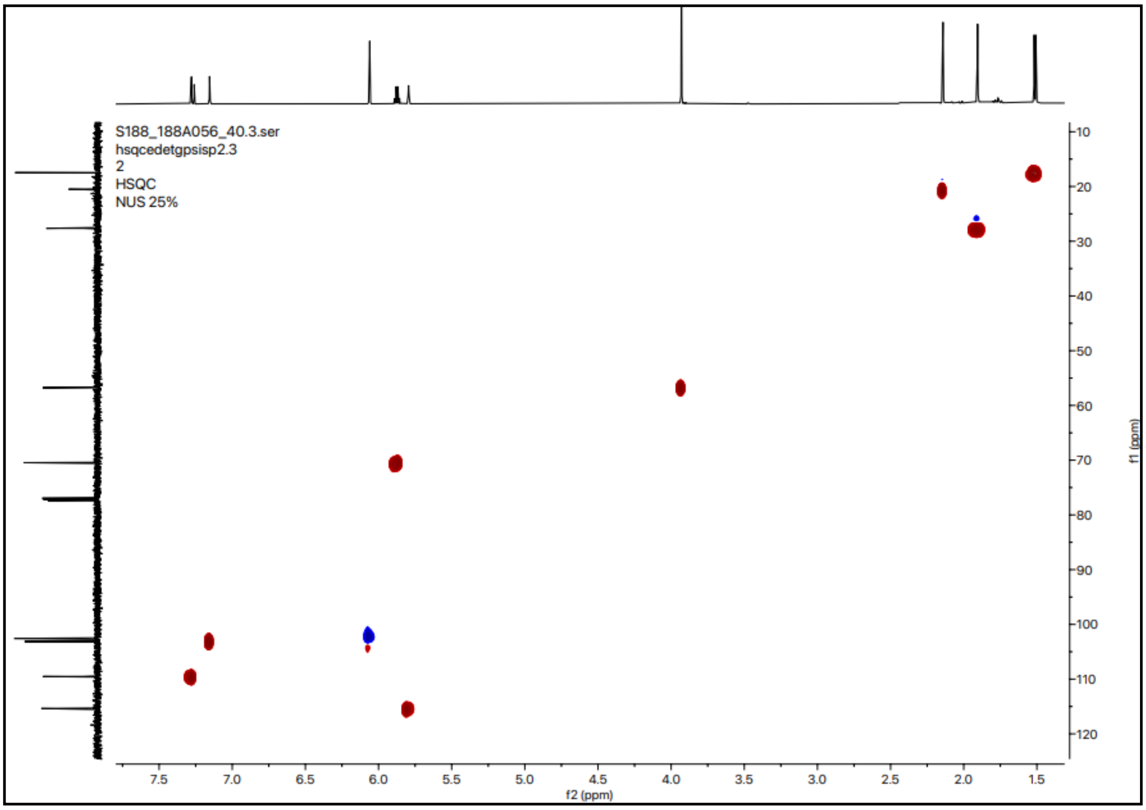

Figure S41: HSQC spectrum of drudeanone (**5**).

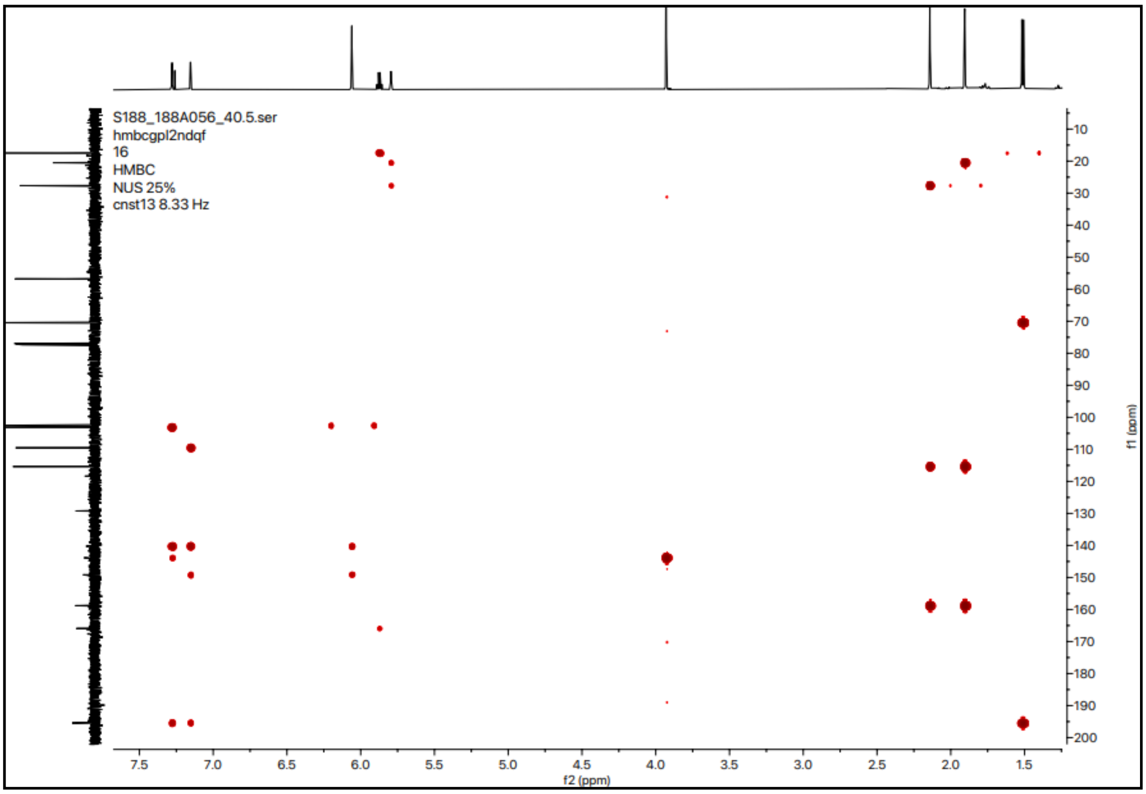

Figure S42: HMBC spectrum of drudeanone (**5**).

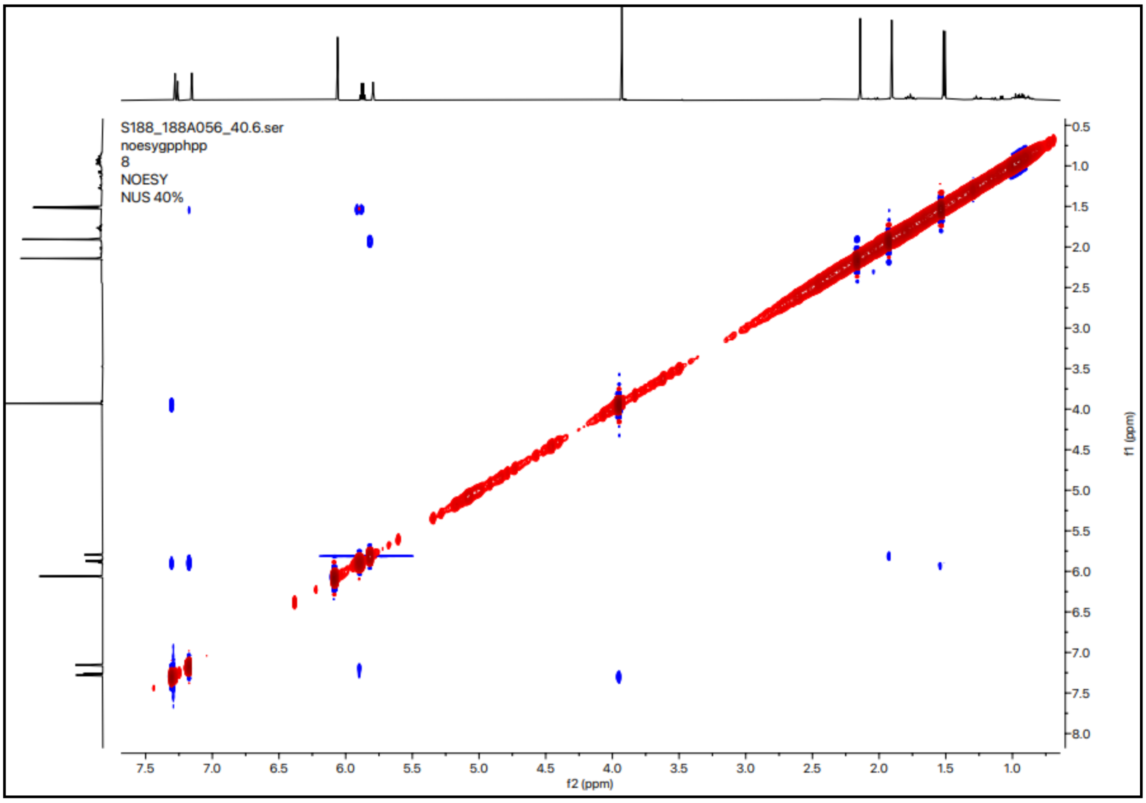

Figure S43: NOESY spectrum of drudeanone (**5**).

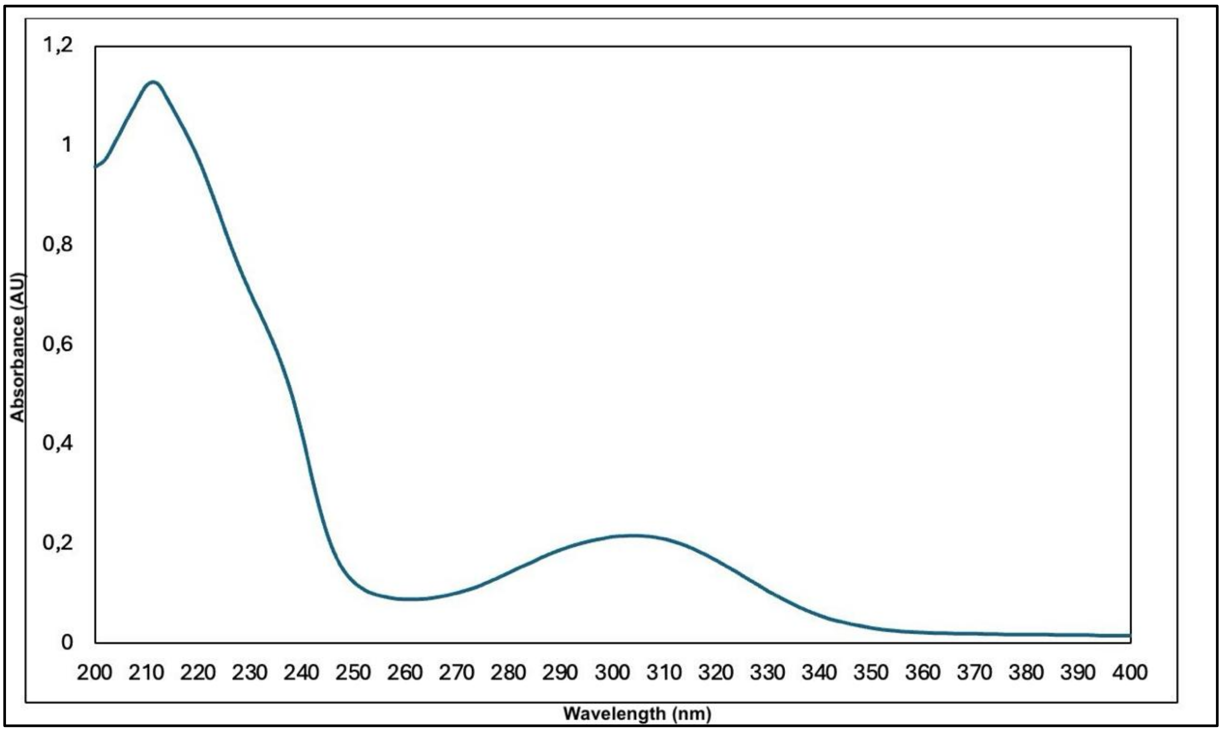

Figure S44: UV spectrum (MeOH) of drudeanone (**5**).

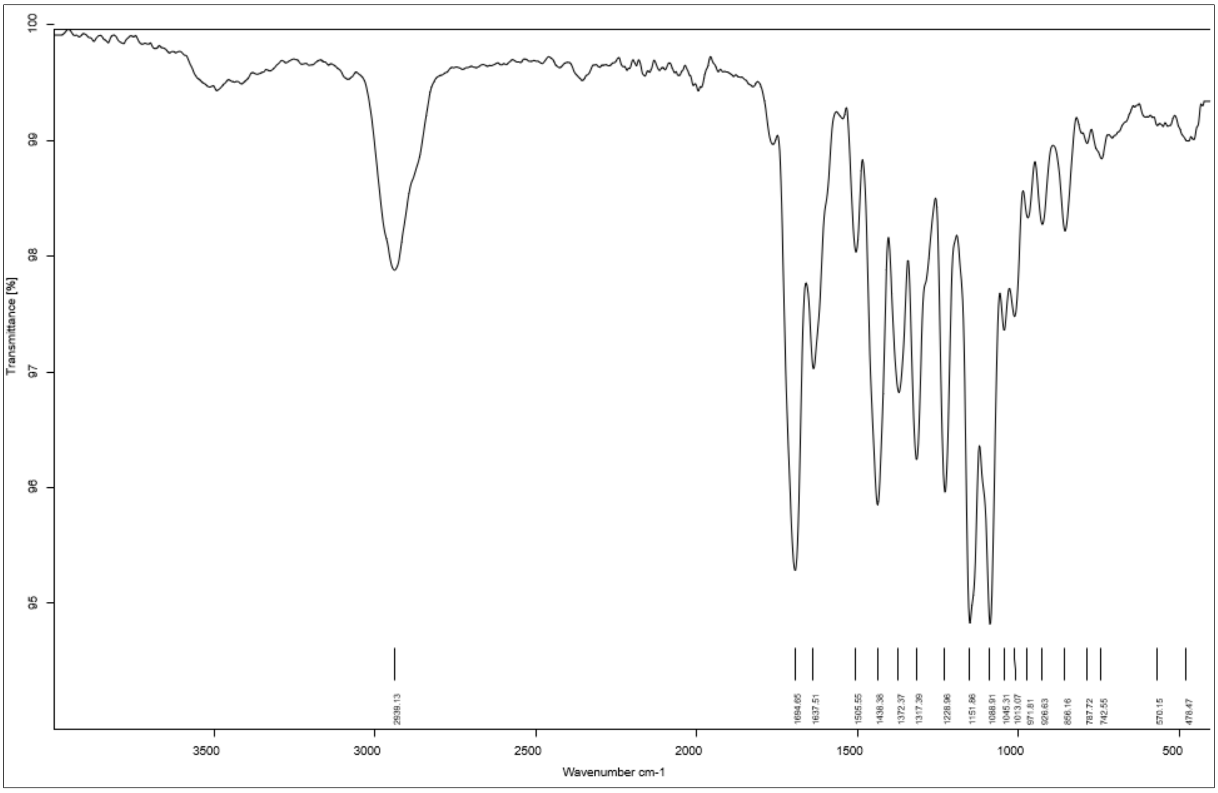

Figure S45: IR spectrum of drudeanone (**5**).

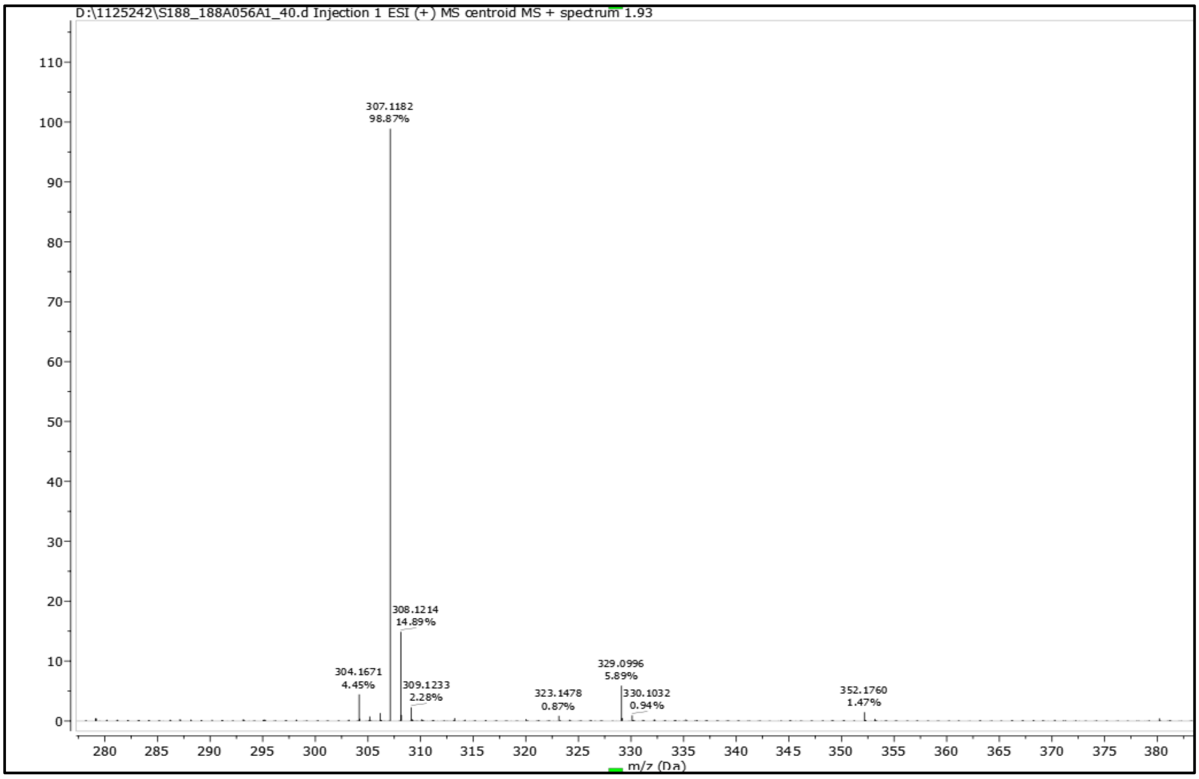

Figure S46: HRESIMS spectrum of drudeanone (**5**).

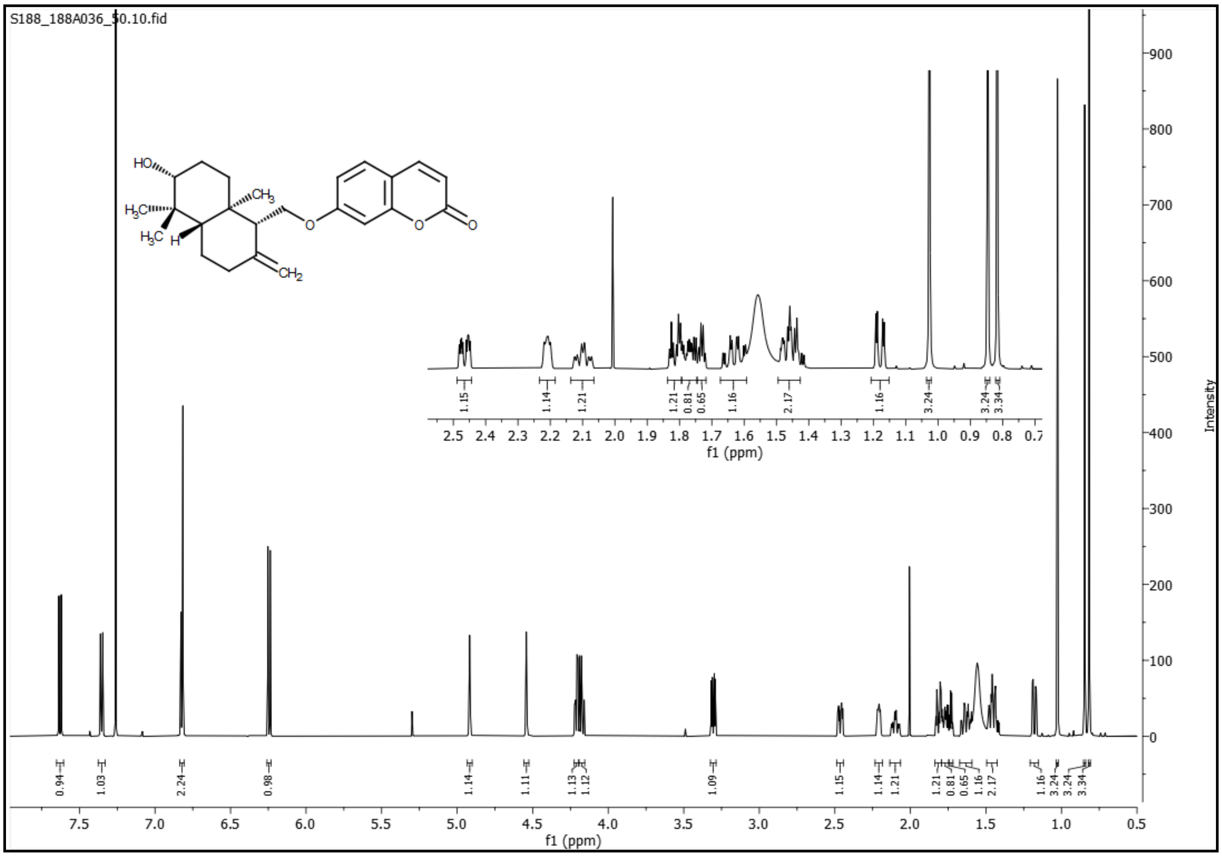

Figure S47: ^{1}H NMR spectrum (600 MHz, $CDCl_3$) of colladonin (**6**).

Colladonin: ^{1}H NMR (600 MHz, $CDCl_3$) δ (ppm): 7.63 (d, *J*: 9.4 Hz, 1H, C_4-H), 7.35 (d, *J*: 9.2 Hz, 1H, C_5-H), 6.84-6.81 (m, 2H, C_6-H ve C_8-H), 6.24 (d, *J*: 9.44 Hz, 1H, C_3-H), 4.92 (br s, 1H, $C_{12'\beta}$-H), 4.54 (br s, 1H, $C_{12'\alpha}$-H), 4.21 (dd, *J*: 9.7; 4.2 Hz, 1H, $C_{11'\beta}$-H), 4.17 (dd, *J*: 9.7; 7.6 Hz, 1H, $C_{11'\alpha}$-H), 3.3 (dd, *J*: 11.73; 4.3 Hz, 1H, $C_{3'}$-H), 2.46 (ddd, *J*: 13.14; 4.34; 2.40 Hz, 1H, $C_{7'\beta}$-H), 2.21 (br dd, *J*: 7.30; 3.8 Hz, 1H, $C_{9'}$-H), 2.10 (td, *J*: 13.2; 5.21, 1H, $C_{7'\alpha}$-H), 1.81 (dt, *J*: 13.3; 3.6 Hz, 1H, $C_{1'\beta}$-H), 1.79-1.75 (m, 1H, $C_{6'\beta}$-H), 1.73 (q, *J*: 3.8 Hz, 1H, $C_{2'\beta}$-H), 1.63 (qd, *J*: 13.2; 3.4 Hz, 1H, $C_{2'\alpha}$-H), 1.49-1.43 (m, 2H, $C_{1'\alpha}$-H, and $C_{6'\alpha}$-H), 1.18 (dd, *J*: 12.53; 2.76 Hz, 1H, $C_{5'}$-H), 1.03 (s, 3H, $C_{14'}\underline{H_3}$), 0.85 (s, 3H, $C_{15'}\underline{H_3}$), 0.82 (s, 3H, $C_{13'}\underline{H_3}$).

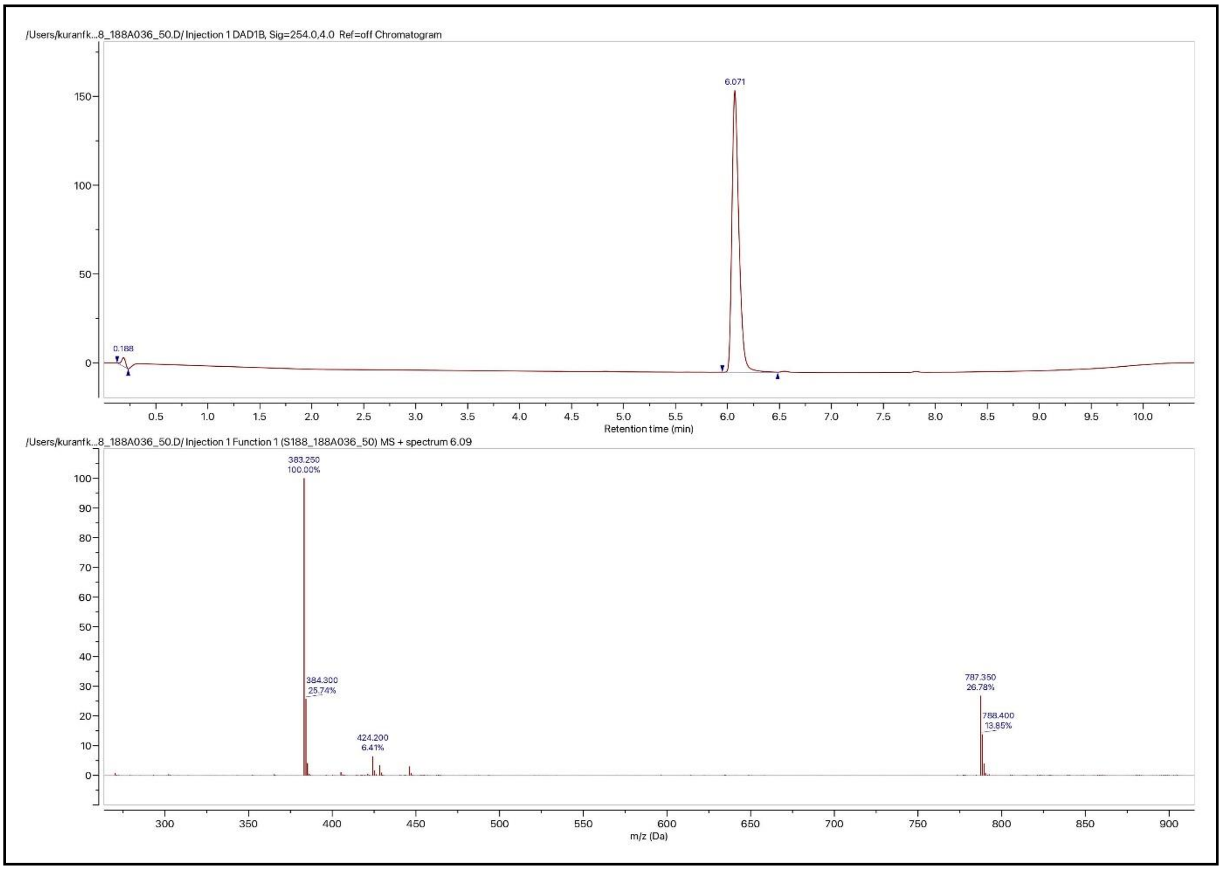

Figure S48: LC-MS spectrum of colladonin (**6**).

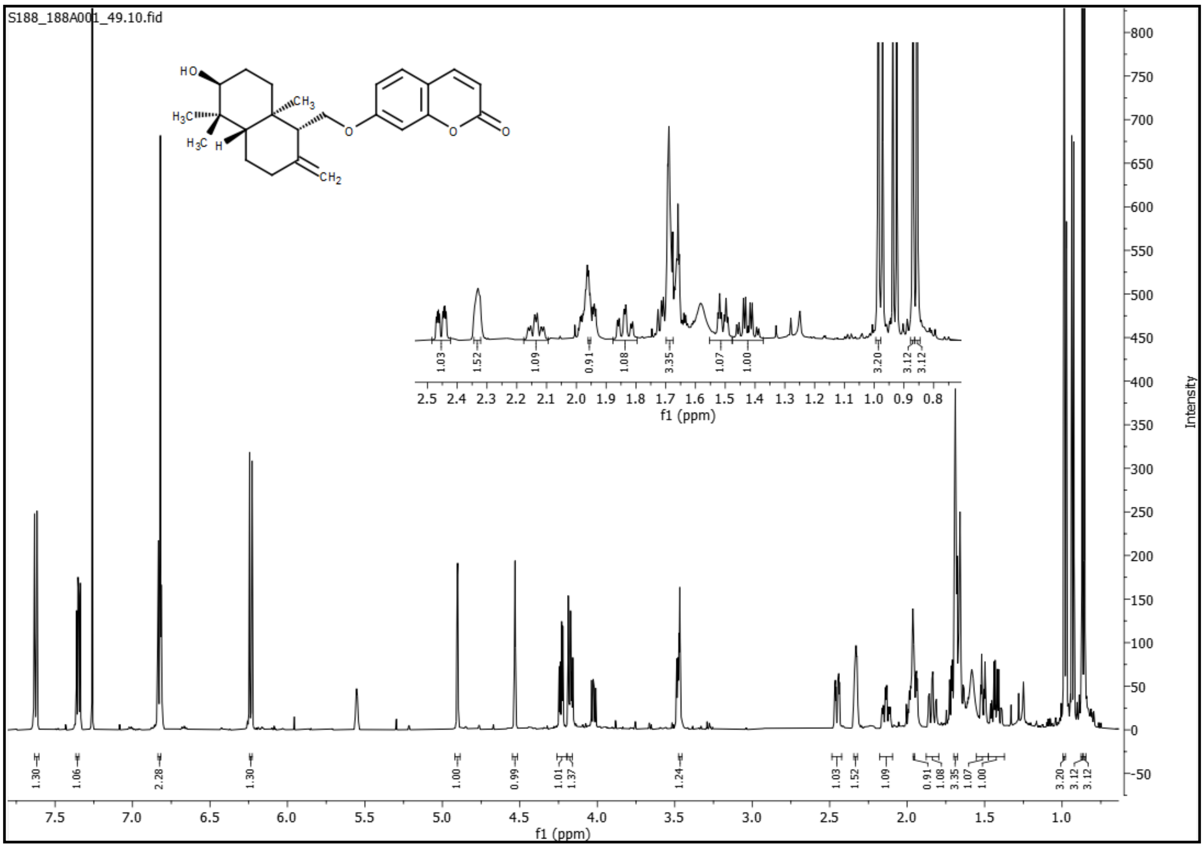

Figure S49: ^{1}H NMR spectrum (600 MHz, $CDCl_3$) of badrakemin (**7**).

Badrakemin: ^{1}H NMR (600 MHz, $CDCl_3$) δ (ppm): 7.62 (d, *J*: 9.4 Hz, 1H, C_4-H), 7.35 (d, *J*: 8.4 Hz, 1H, C_5-H), 6.84-6.82 (m, 2H, C_6-H, and C_8-H), 6.23 (d, *J*: 9.4 Hz, 1H, C_3-H), 4.90 (d, *J*: 1.5 Hz, 1H, $C_{12'\beta}$-H), 4.53 (d, *J*: 1.4 Hz, 1H, $C_{12'\alpha}$-H), 4.23 (dd, *J*: 9.68; 4.1 Hz, 1H, $C_{11'\beta}$-H), 4.18 (dd, *J*: 9.7; 7.7 Hz, 1H, $C_{11'\alpha}$-H), 3.47 (t, *J*: 2.9 Hz, 1H, $C_{3'}$-H), 2.45 (ddd, *J*: 13.18; 4.38; 2.31 Hz, 1H, $C_{7'\beta}$-H), 2.33 (dd, *J*: 7.8; 4.2 Hz, 1H, $C_{9'}$-H), 2.14 (td, *J*: 13.15; 5.06 Hz, 1H, $C_{7'\alpha}$-H), 1.96-1.94 (m, 1H, $C_{2'\beta}$-H), 1.84 (td, *J*: 13.48; 3.57 Hz, 1H, $C_{1'\beta}$-H), 1.70-1.68 (m, 3H, $C_{2'\alpha}$-H, $C_{5'}$-H, and $C_{6'\beta}$-H), 1.51 (dt, *J*: 12.94, 3.51 Hz, $C_{1'\alpha}$-H), 1.42 (qd, *J*: 13.31; 4.42 Hz, 1H, $C_{6'\alpha}$-H), 0.99 (s, 3H, $C_{14'}\underline{H_3}$), 0.86 ($C_{15'}\underline{H_3}$), 0.87 (s, 3H, $C_{13'}\underline{H_3}$).

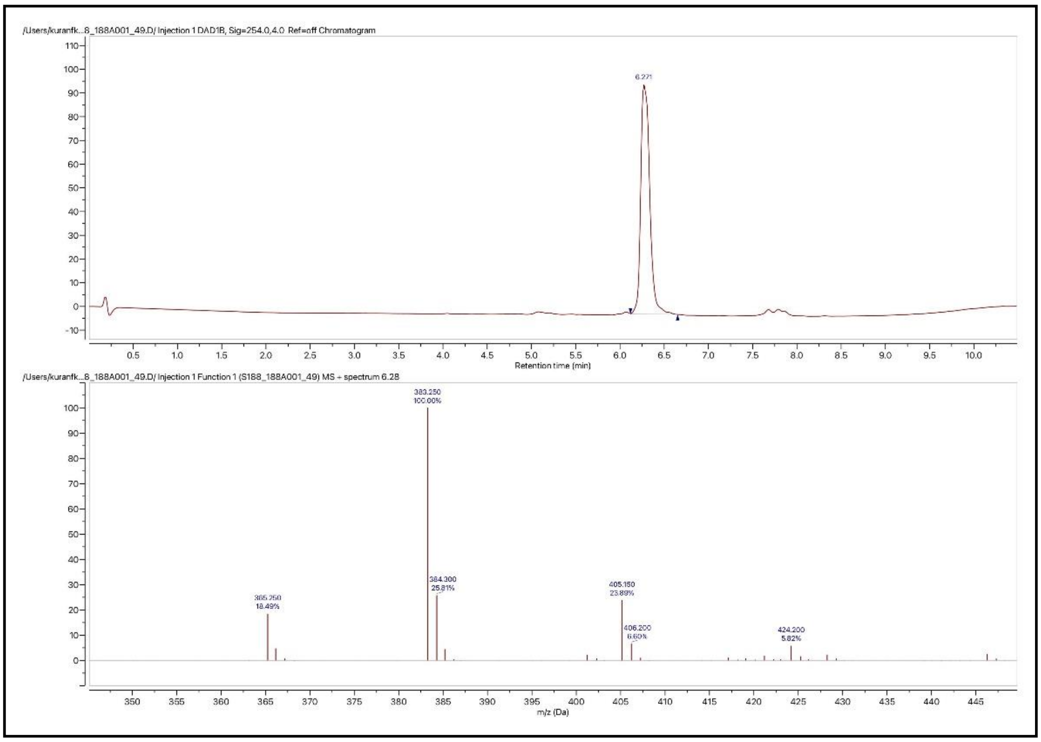

Figure S50: LC-MS spectrum of badrakemin (**7**).

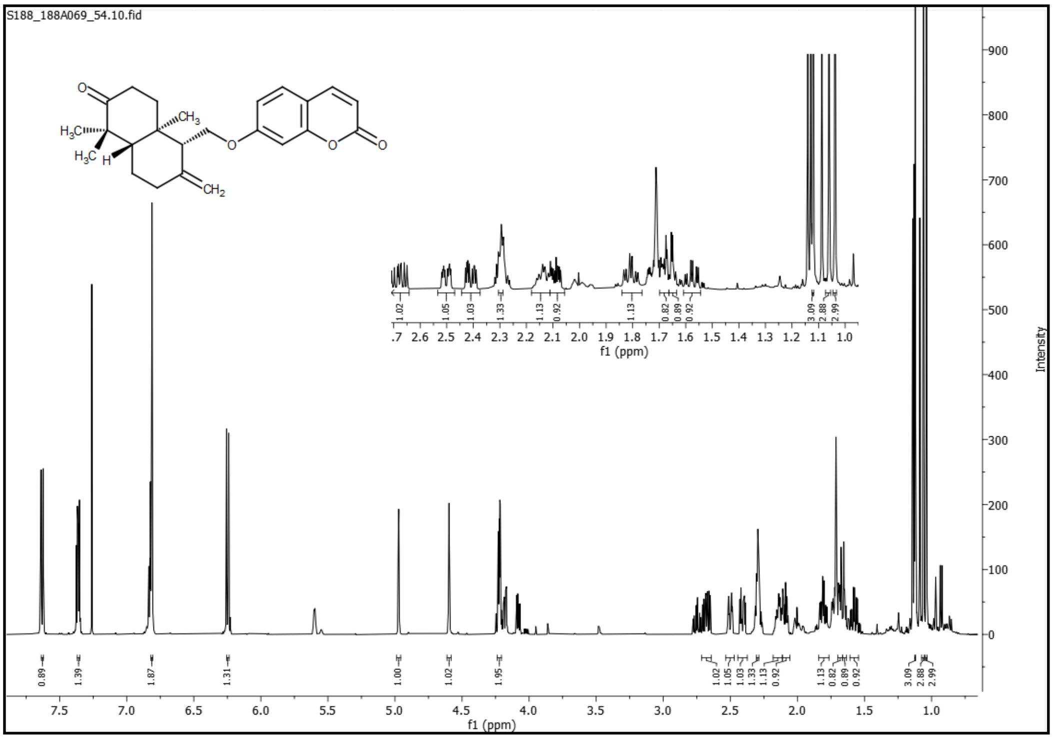

Figure S51: ^{1}H NMR spectrum (600 MHz, $CDCl_3$) of badrakemone (**8**).

Badrakemone: ^{1}H NMR (600 MHz, $CDCl_3$) δ (ppm): 7.63 (d, *J*: 9.5 Hz, 1H, C_4-H), 7.36 (d, *J*: 9.3 Hz, 1H, C_5-H), 6.83-6.80 (m, 2H, C_6-H, and C_8-H), 6.25 (d, *J*: 9.5 Hz, 1H, C_3-H), 4.97 (br s, 1H, $C_{12'\beta}$-H), 4.60 (br s, 1H, $C_{12'\alpha}$-H), 4.24- 4.18 (m, 2H, $C_{11'\alpha}$-H, and $C_{11'\beta}$-H), 2.68 (tdd, *J*: 15.3; 13.2; 6.2 Hz, 1H, $C_{2'\alpha}$-H), 2.5 (ddd, *J*: 13.1; 4.2; 2.4 Hz, 1H, $C_{7'\beta}$-H), 2.41 (ddd, *J*: 15.3; 5.3; 3.4 Hz, 1H, $C_{2'\beta}$-H), 2.3 (t, *J*: 5.9 Hz, 1H, $C_{9'}$-H), 2.15 (td, *J*: 13.3; 3.4 Hz, 1H, $C_{7'\alpha}$-H), 2.09 (ddd, *J*: 13.3; 6.3; 3.5 Hz, 1H, $C_{1'\alpha}$-H), 1.81 (td, *J*: 13.3; 5.4 Hz, 1H, $C_{1'\beta}$-H), 1.70-1.66 (m, 1H, $C_{6'\beta}$-H), 1.65 (dd, *J*: 12.5; 2.7 Hz, 1H, $C_{5'}$-H), 1.58 (dt, *J*: 12.7; 4.1 Hz, 1H, $C_{6'\alpha}$-H), 1.12 (s, 3H, $C_{13'}\underline{H_3}$), 1.06 (s, 3H, $C_{14'}\underline{H_3}$), 1.04 (s, 3H, $C_{15'}\underline{H_3}$).

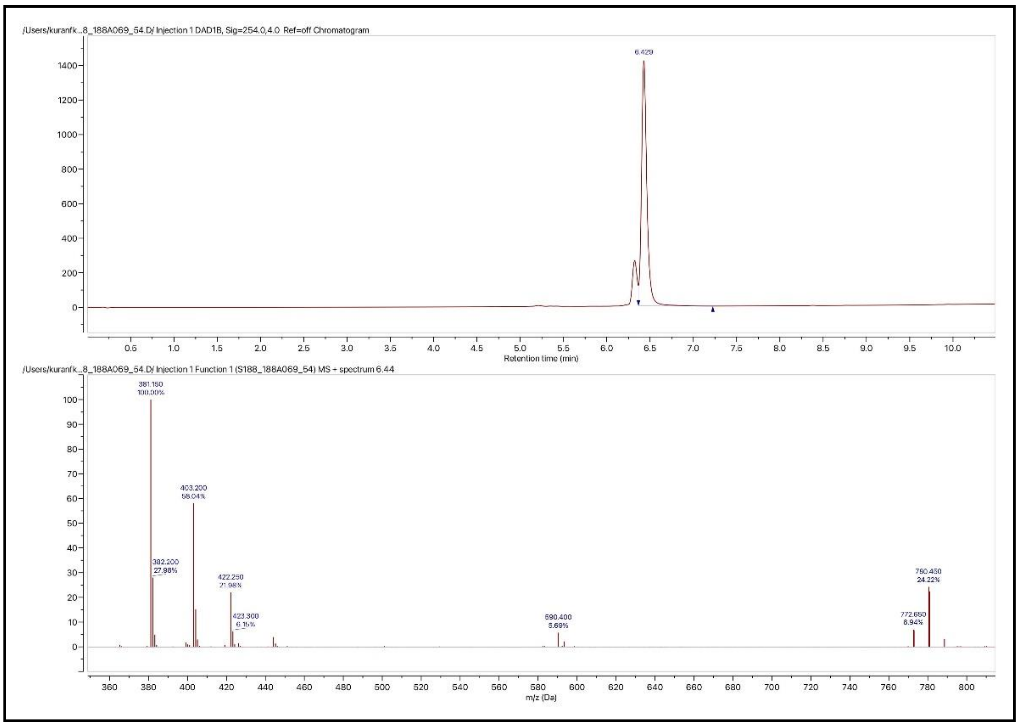

Figure S52: LC-MS spectrum of badrakemone (**8**).

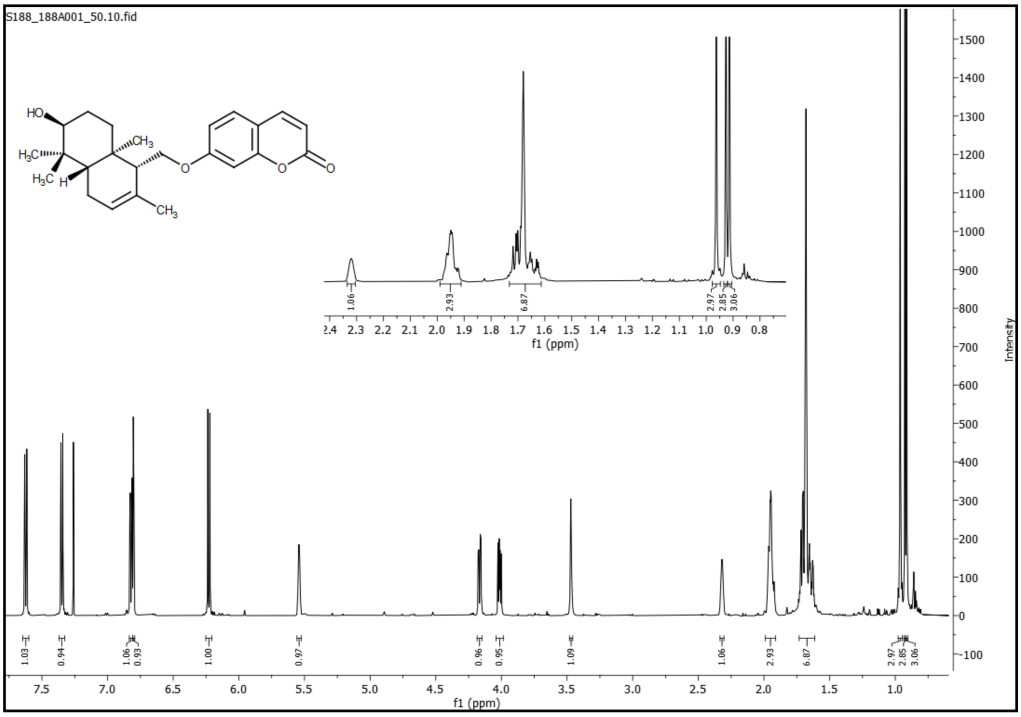

Figure S53: ^{1}H NMR spectrum (600 MHz, $CDCl_3$) of conferol (**9**).

Conferol: ^{1}H NMR (600 MHz, $CDCl_3$) δ (ppm): 7.62 (d, *J*: 9.4 Hz, 1H, C_4-H), 7.35 (d, *J*: 8.5 Hz, 1H, C_5-H), 6.82 (dd, *J*: 8.5; 2.4 Hz, 1H, C_6-H), 6.80 (d, *J*: 2.4 Hz, 1H, C_8-H), 6.23 (d, *J*: 9.4 Hz, 1H, C_3-H), 5.54 (br s, 1H, $C_{7'\alpha}$-H), 4.17 (dd, *J*: 9.6; 3.4 Hz, 1H, $C_{11'\beta}$-H), 4.01 (dd, *J*: 9.6; 5.9 Hz, 1H, $C_{11'\alpha}$-H), 3.47 (br t, *J*: 2.3 Hz, 1H, $C_{3'}$-H), 2.32 (br s, 1H, $C_{9'}$-H), 1.99-1.91 (m, 3H, $C_{2'\alpha}$-H, $C_{6'\alpha}$-H, and $C_{6'\beta}$-H), 1.73-1.61 (m, 4H, $C_{1'\alpha}$-H, $C_{1'\beta}$-H, $C_{2'\beta}$-H, and $C_{5'}$-H), 1.68 (br s, 3H, $C_{12'}\underline{H_3}$), 0.96 (s, 3H, $C_{14'}\underline{H_3}$), 0.93 (s, 3H, $C_{13'}\underline{H_3}$), 0.91 (s, 3H, $C_{15'}\underline{H_3}$).

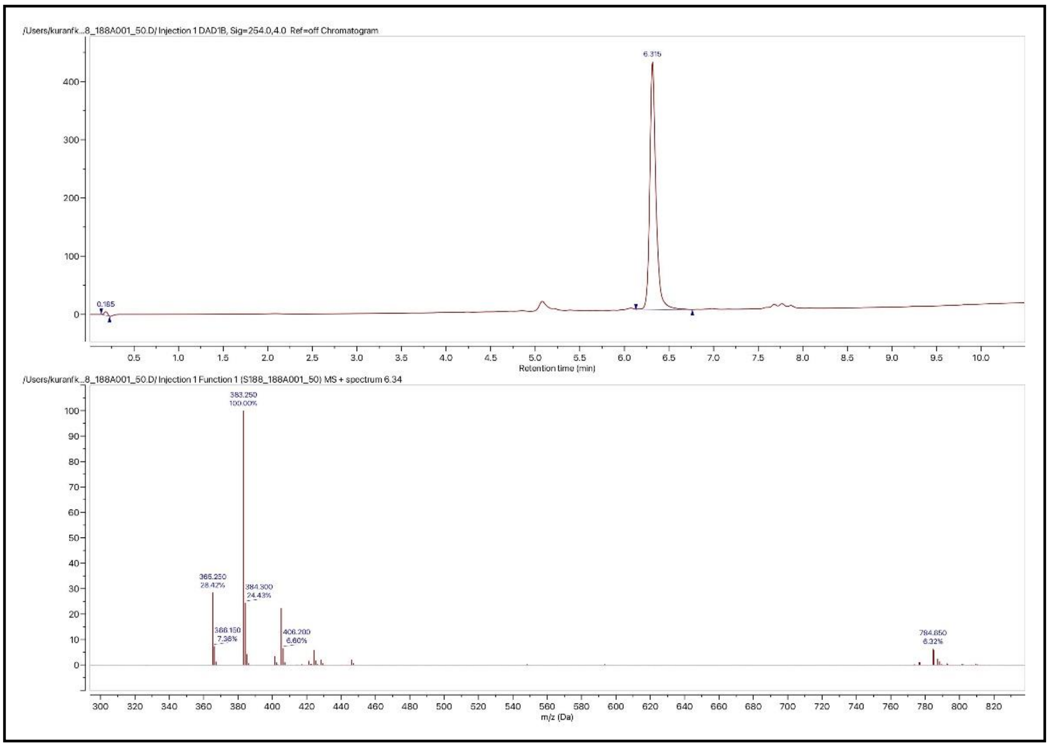

Figure S54: LC-MS spectrum of conferol (**9**).

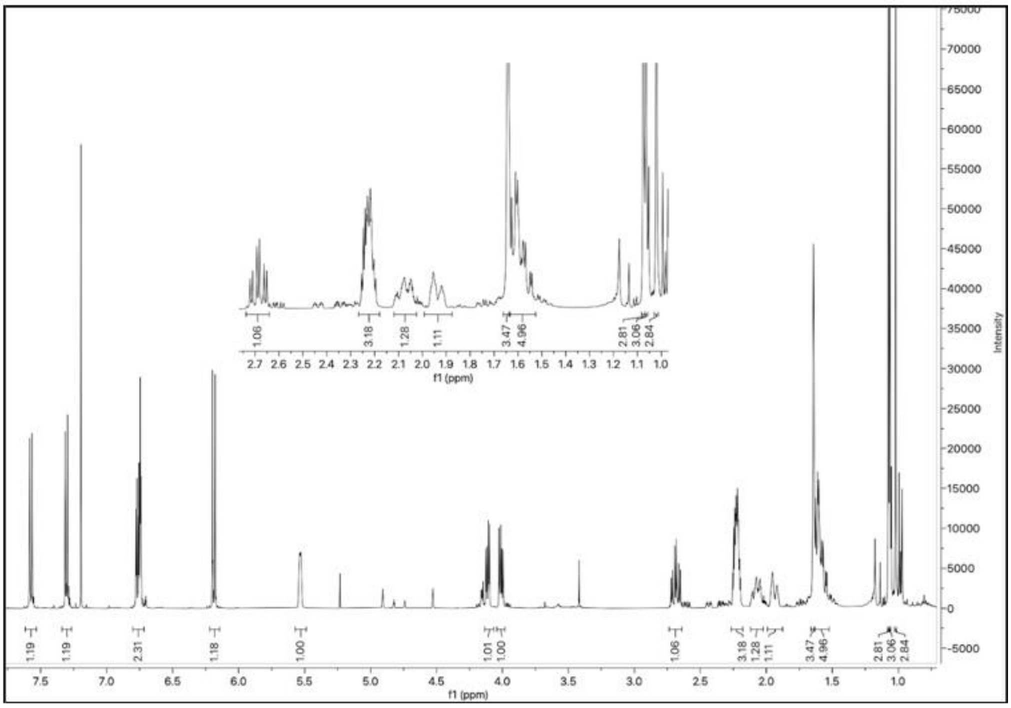

Figure S55: ^{1}H NMR spectrum (600 MHz, $CDCl_3$) of conferone (**10**).

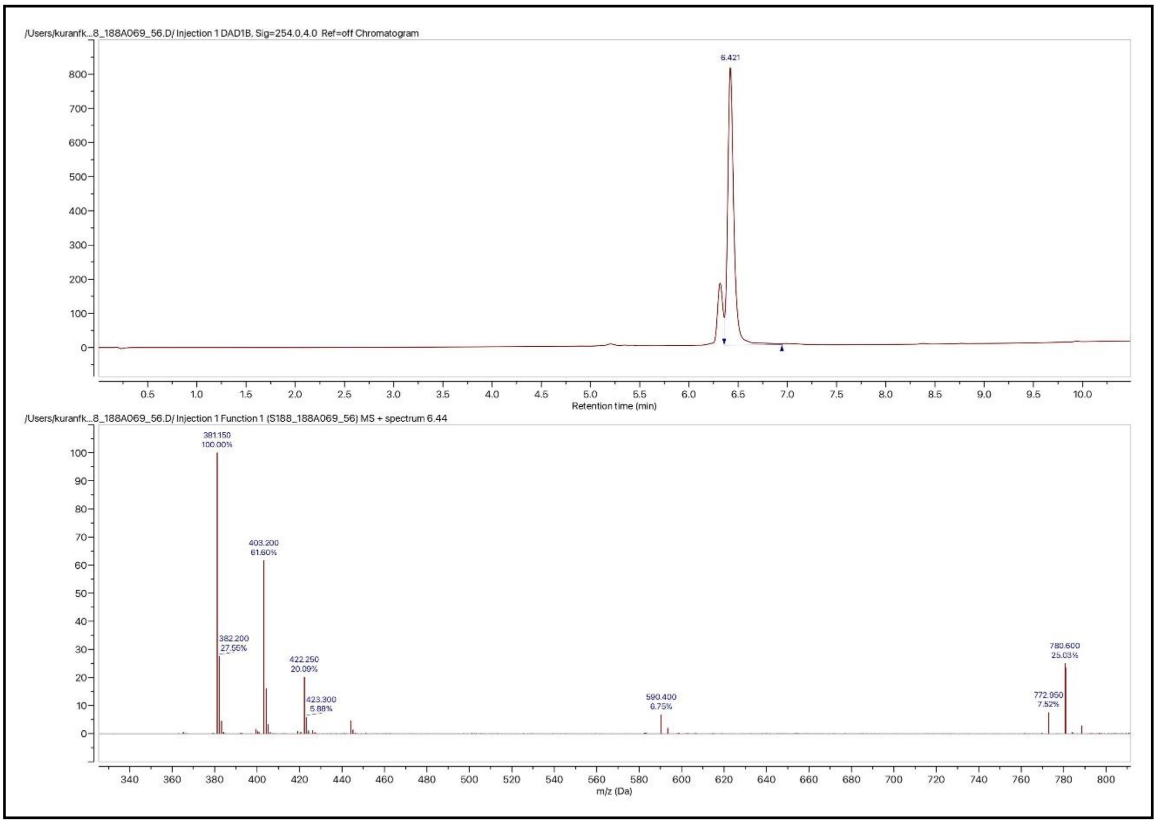

Figure S56: LC-MS spectrum of conferone (**10**).

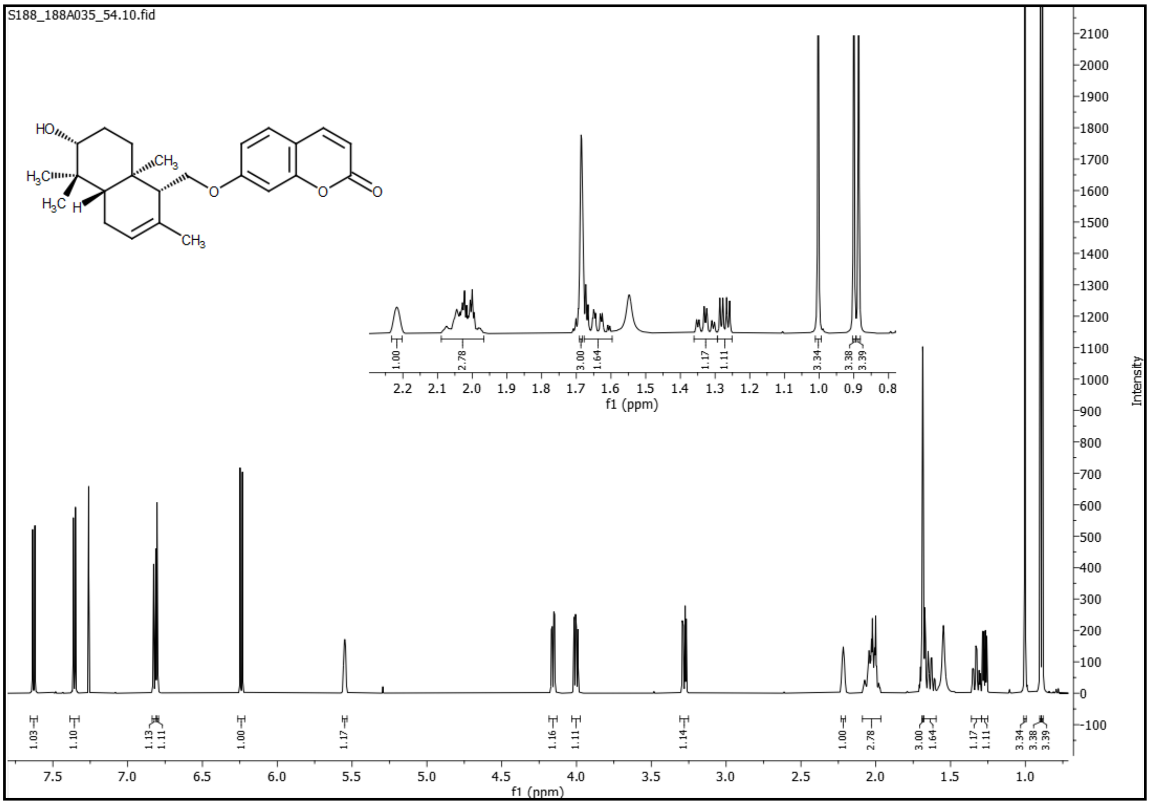

Figure S57: ^{1}H NMR spectrum (600 MHz, $CDCl_3$) of feselol (**11**).

Feselol: ^{1}H NMR (600 MHz, $CDCl_3$) δ (ppm): 7.63 (d, *J*: 9.4 Hz, 1H, C_4-H), 7.36 (d, *J*: 8.45 Hz, 1H, C_5-H), 6.82 (dd, *J*: 8.45; 2.4 Hz, 1H, C_6-H), 6.8 (d, *J*: 2.4 Hz, 1H, C_8-H), 6.24 (d, *J*: 9.4 Hz, 1H, C_3-H), 5.55 (br s, 1H, $C_{7'\alpha}$-H), 4.16 (dd, *J*: 9.6; 3.4 Hz, 1H, $C_{11'\beta}$-H), 4.00 (dd, *J*: 9.6; 5.9 Hz, 1H, $C_{11'\alpha}$-H), 3.28 (dd, *J*: 11.3; 4.4 Hz, 1H, $C_{3'}$H), 2.22 (br s, 1H, $C_{9'}$-H), 2.09-1.96 (m, 3H, $C_{1'\alpha}$H, $C_{6'\alpha}$H, and $C_{6'\beta}$H), 1.69 (br s, 3H, $C_{12'}\underline{H_3}$), 1.68-1.60 (m, 2H, $C_{2'\alpha}$-H, and $C_{2'\beta}$-H), 1.33 (td, *J*: 13.2; 4.3 Hz, 1H, $C_{1'\beta}$-H), 1.27 (dd, *J*: 11.6; 5.2 Hz, 1H, $C_{5'}$-H), 1.00 (s, 3H, $C_{13'}\underline{H_3}$), 0.9 (s, 3H, $C_{14'}\underline{H_3}$), 0.89 (s, 3H, $C_{15'}\underline{H_3}$).

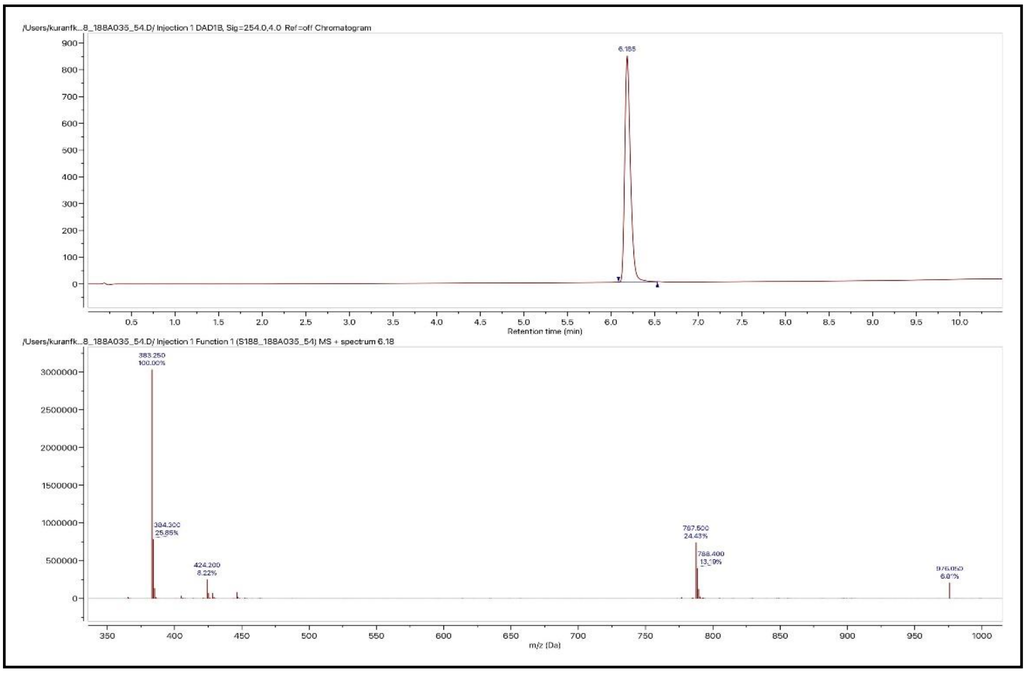

Figure S58: LC-MS spectrum of feselol (**11**).

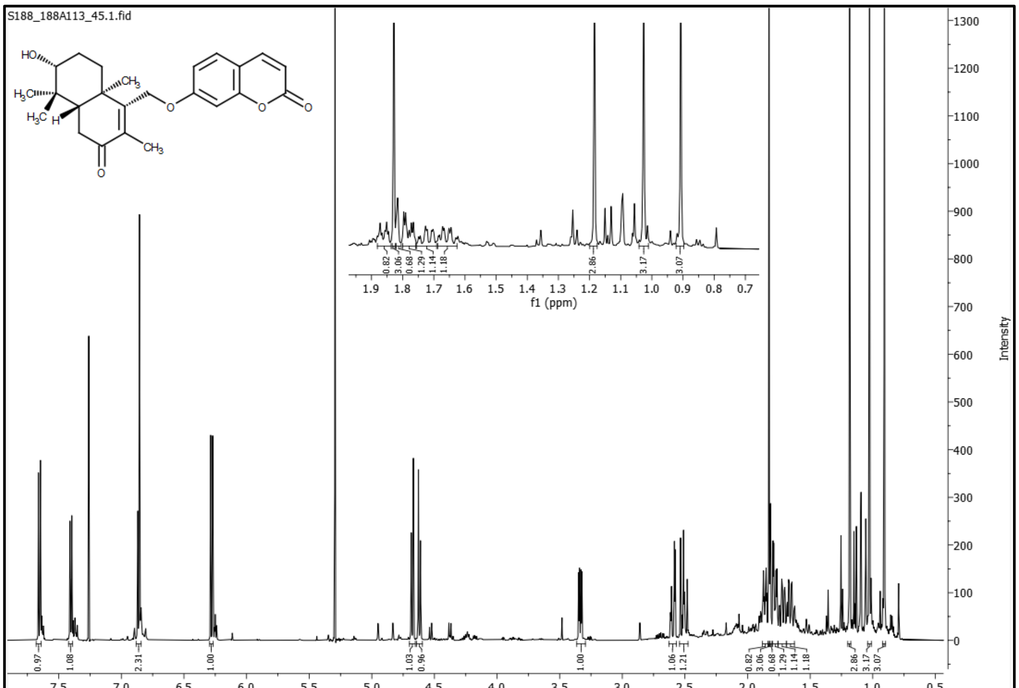

Figure S59: ^{1}H NMR spectrum (600 MHz, $CDCl_3$) of fesinkin F (**12**).

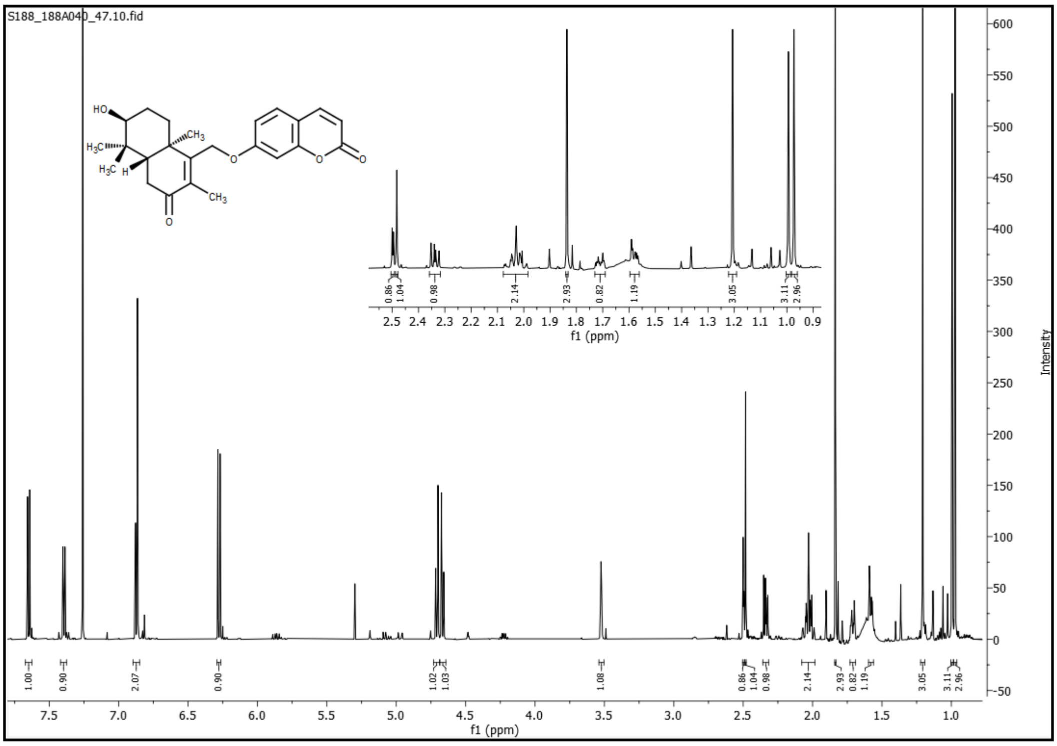

Figure S60: ^{1}H NMR spectrum (600 MHz, $CDCl_3$) of ferubungeanol G (**13**).

Ferubungeanol G: ^{1}H NMR (600 MHz, $CDCl_3$) δ (ppm): 7.64 (d, *J*: 9.4 Hz, 1H, C_4-H), 7.39 (d, *J*: 8.4 Hz, 1H, C_5-H), 6.90-6.85 (m, 2H, C_6-H, and C_8-H), 6.28 (d, *J*: 9.4 Hz, 1H, C_3-H), 4.71 (d, *J*: 9.9 Hz, 1H, $C_{11'\alpha}$-H), 4.67 (d, *J*: 9.9 Hz, $C_{11'\beta}$-H), 3.52 (br s, 1H, $C_{3'}$H), 2.5 (d, *J*: 3 Hz, 1H, $C_{6'\alpha}$-H), 2.48 (br s, 1H, $C_{6'\beta}$-H), 2.34 (dd, *J*: 10.6; 7.5 Hz, 1H, $C_{5'}$-H), 2.08-1.98 (m, 2H, $C_{1'\beta}$-H, and $C_{2'\alpha}$-H), 1.84 (s, 3H, $C_{12'}\underline{H_3}$), 1.73-1.69 (m, 1H, $C_{2'\beta}$-H), 1.60-1.56 (m, 1H, $C_{1'\alpha}$-H), 1.21 (s, 3H, $C_{15'}\underline{H_3}$), 0.99 (s, 3H, $C_{13'}\underline{H_3}$), 0.97 (s, 3H, $C_{14'}\underline{H_3}$).

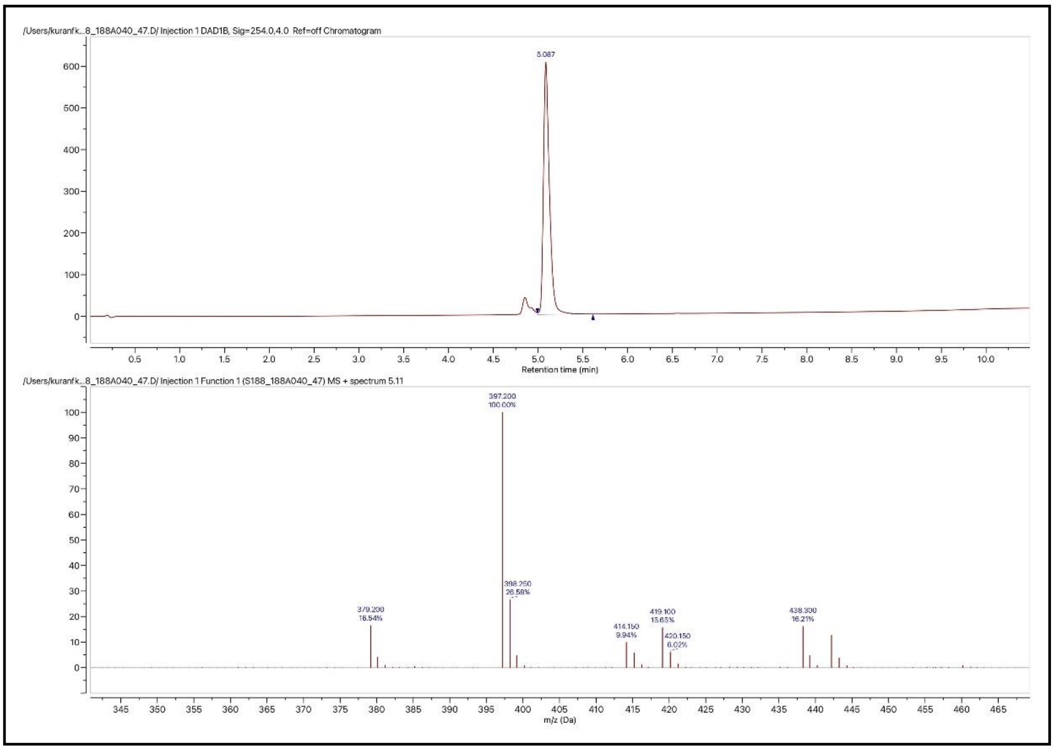

Figure S61: LC-MS spectrum of ferubungeanol G (**13**).

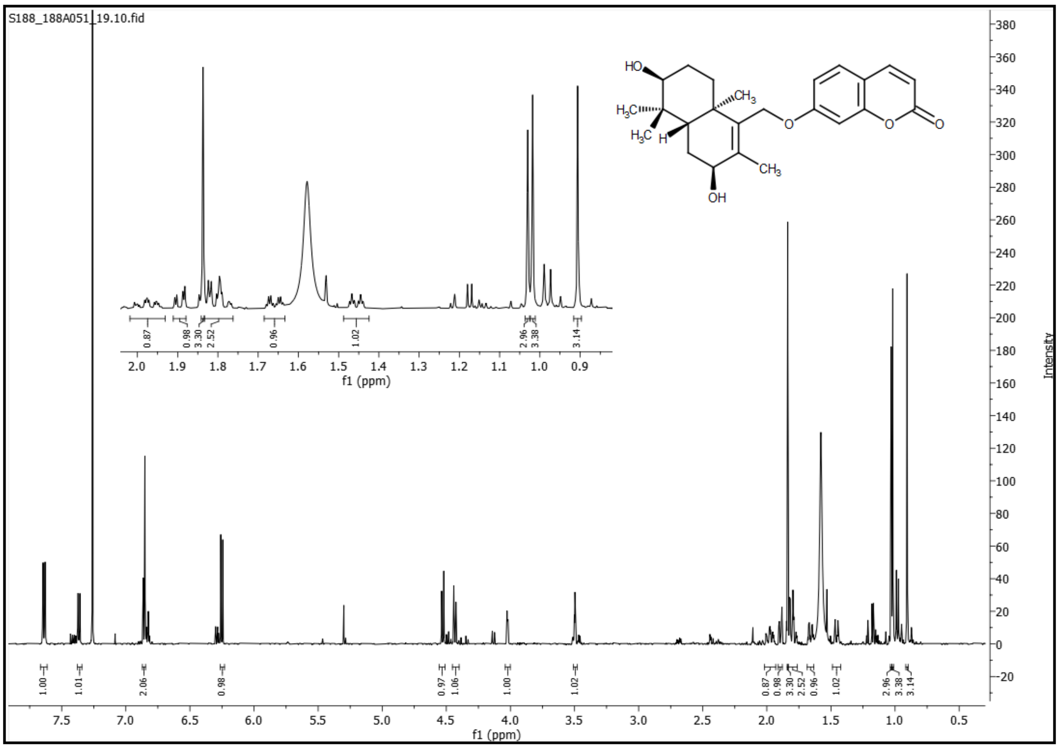

Figure S62: ^{1}H NMR spectrum (600 MHz, $CDCl_3$) of samarkandicin K (**14**).

Samarcandicin K: ^{1}H NMR (600 MHz, $CDCl_3$) δ (ppm): 7.64 (d, *J*: 9.8 Hz, 1H, C_4-H), 7.36 (d, *J*: 8.3 Hz, 1H, C_5-H), 6.87-6.85 (m, 2H, C_6-H, and C_8-H), 6.25 (d, *J*: 9.4 Hz, 1H, C_3-H), 4.53 (d, *J*: 9.8 Hz, 1H, $C_{11'\alpha}$-H), 4.43 (d, *J*: 9.8 Hz, 1H, $C_{11'\beta}$-H), 4.02 (br t, *J*: 3 Hz, 1H, $C_{7'}$-H), 3.5 (t, *J*: 3 Hz, 1H, $C_{3'}$-H), 1.97 (tt, *J*: 14.5; 3.2 Hz, 1H, $C_{2'\alpha}$-H), 1.89 (dd, *J*: 12; 3 Hz, 1H, $C_{5'}$-H), 1.84 (s, 3H, $C_{12'}$-H), 1.83-1.76 (m, 3H, $C_{1'\beta}$-H, $C_{6'\alpha}$-H, and $C_{6'\beta}$-H), 1.66 (dq, *J*: 14.5; 3.3 Hz, 1H, $C_{2'\beta}$-H), 1.46 (dt, *J*: 13.1; 3.6 Hz, 1H, $C_{1'\alpha}$-H), 1.03 (s, 3H, $C_{13'}\underline{H_3}$), 1.02 (s, 3H, $C_{15'}\underline{H_3}$), 0.91 (s, 3H, $C_{14'}\underline{H_3}$).

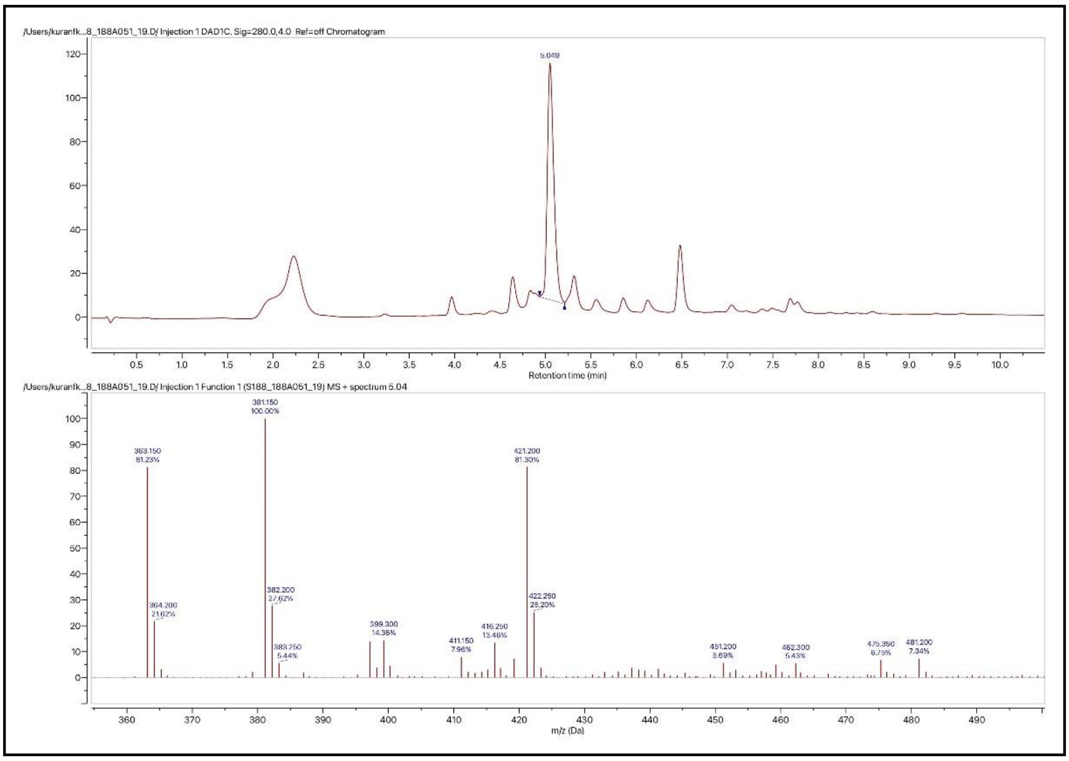

Figure S63: LC-MS spectrum of samarkandicin K (**14**).

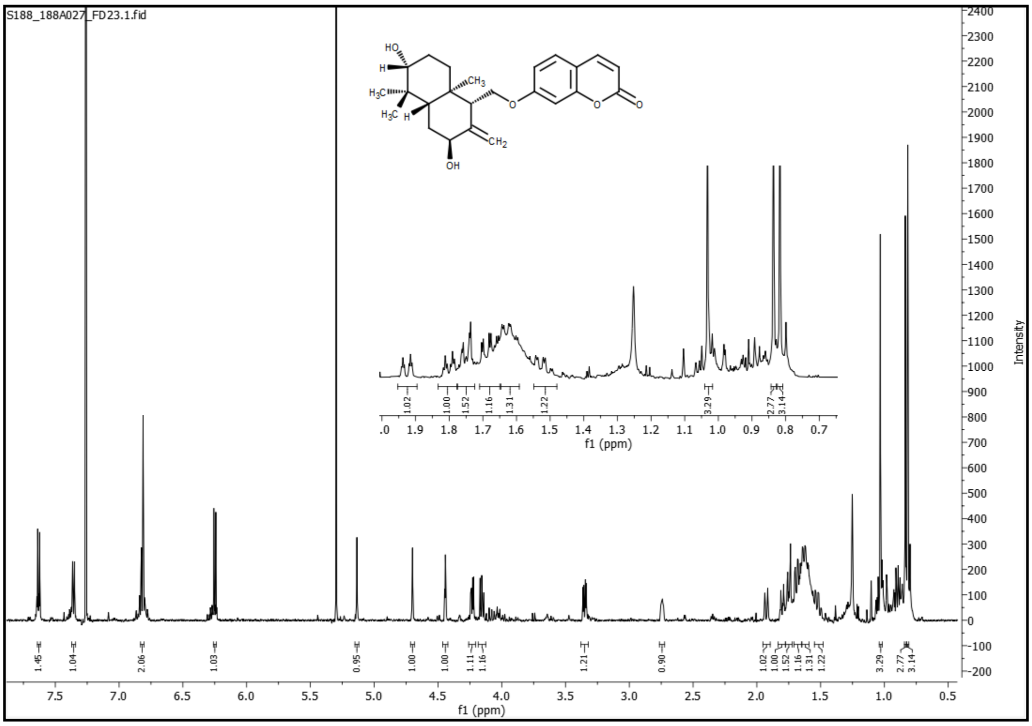

Figure S64: ^{1}H-NMR spectrum (600 MHz, $CDCl_3$) of samarkandicin J (**15**).

Samarcandicin J: ^{1}H NMR (600 MHz, $CDCl_3$) δ (ppm): 7.63 (d, *J*: 9.5 Hz, 1H, C_4-H), 7.36 (d, *J*: 7.5 Hz, 1H, C_5-H), 6.83-6.80 (m, 2H, C_6-H, and C_8-H), 6.25 (d, *J*: 9.5 Hz, 1H, C_3-H), 5.14 (s, 1H, $C_{12'\alpha}$-H), 4.70 (s, 1H, $C_{12'\beta}$-H), 4.44 (t, *J*: 3.0 Hz, 1H, $C_{7'}$-H), 4.23 (dd, *J*: 9.8; 4.2 Hz, 1H, $C_{11'\alpha}$-H), 4.16 (t, *J*: 8.9 Hz, 1H, $C_{11'\beta}$-H), 3.35 (dd, *J*: 11.7; 4.2 Hz, 1H, $C_{3'}$-H), 2.76-2.72 (m, 1H, $C_{9'}$-H), 1.93 (dt, *J*: 13.6; 3.0 Hz, 1H, $C_{6'\beta}$-H), 1.80 (dt, *J*: 13.0; 3.5 Hz, 1H, $C_{1'\alpha}$-H), 1.75 (br d, *J*: 13.2 Hz, 1H, $C_{2'\beta}$-H), 1.74 (br d, *J:* 13.2 Hz, 1H, $C_{5'}$-H), 1.68 (td, *J*: 13.4; 3.2 Hz, 1H, $C_{6'\alpha}$-H), 1.62 (td, *J*: 12.8; 3.3 Hz, 1H, $C_{2'\alpha}$-H), 1.52 (td, *J*: 13.0; 3.5 Hz, 1H, $C_{1'\beta}$-H), 1.03 (s, 3H, $C_{13'}\underline{H_3}$), 0.84 (s, 3H, $C_{15'}\underline{H_3}$), 0.82 (s, 3H, $C_{14'}\underline{H_3}$).

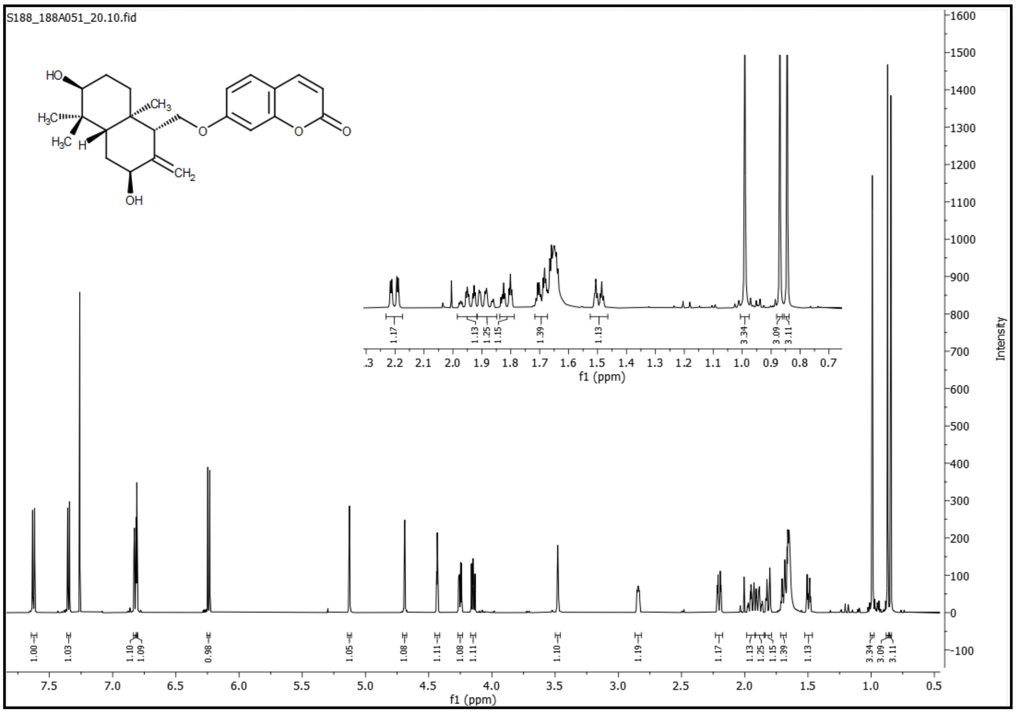

Figure S65: ^{1}H NMR spectrum (600 MHz, $CDCl_3$) of ferubungeanol A (**16**).

Ferubungeanol A: ^{1}H NMR (600 MHz, $CDCl_3$) δ (ppm): 7.63 (d, *J*: 9.4 Hz, 1H, C_4-H), 7.35 (d, *J*: 8.4 Hz, 1H, C_5-H), 6.82 (dd, *J*: 8.4; 2.4 Hz, 1H, C_6-H), 6.8 (d, *J*: 2.4 Hz, 1H, C_8-H), 6.24 (d, *J*: 9.4 Hz, 1H, C_3-H), 5.13 (s, 1H, $C_{12'\alpha}$-H), 4.69 (s, 1H, $C_{12'\beta}$-H), 4.43 (t, *J*: 2.9 Hz, 1H, $C_{7'}$-H), 4.25 (dd, *J*: 9.6; 4.1 Hz, 1H, $C_{11'\alpha}$-H), 4.15 (dd, *J*: 9.6; 7.7 Hz, 1H, $C_{11'\beta}$-H), 3.48 (t, *J*: 3.3 Hz, 1H, $C_{3'}$-H), 2.84 (br dd, *J*: 7.8; 4.0 Hz, $C_{9'}$-H), 2.20 (dd, *J*: 13.4; 2.9 Hz, 1H, $C_{5'}$-H), 1.95 (tt, *J*: 13.6; 3.0 Hz, 1H, $C_{2'\alpha}$-H), 1.89 (ddd, *J*: 13.8; 13.0; 3.0 Hz, 1H, $C_{1'\beta}$-H), 1.81 (dt, *J*: 14.1; 2.9 Hz, 1H, $C_{6'\beta}$-H), 1.69 (dq, *J*: 13.0; 3.4 Hz, 1H, $C_{2'\beta}$-H), 1.64 (ddd, *J*: 14.0; 13.0; 3.0 Hz, 1H, $C_{6'\alpha}$-H), 1.5 (dt, *J*: 12.6; 3.2 Hz, 1H, $C_{1'\alpha}$-H), 0.99 (s, 3H, $C_{13'}\underline{H_3}$), 0.87 (s, 3H, $C_{14'}\underline{H_3}$), 0.84 (s, 3H, $C_{15'}\underline{H_3}$).

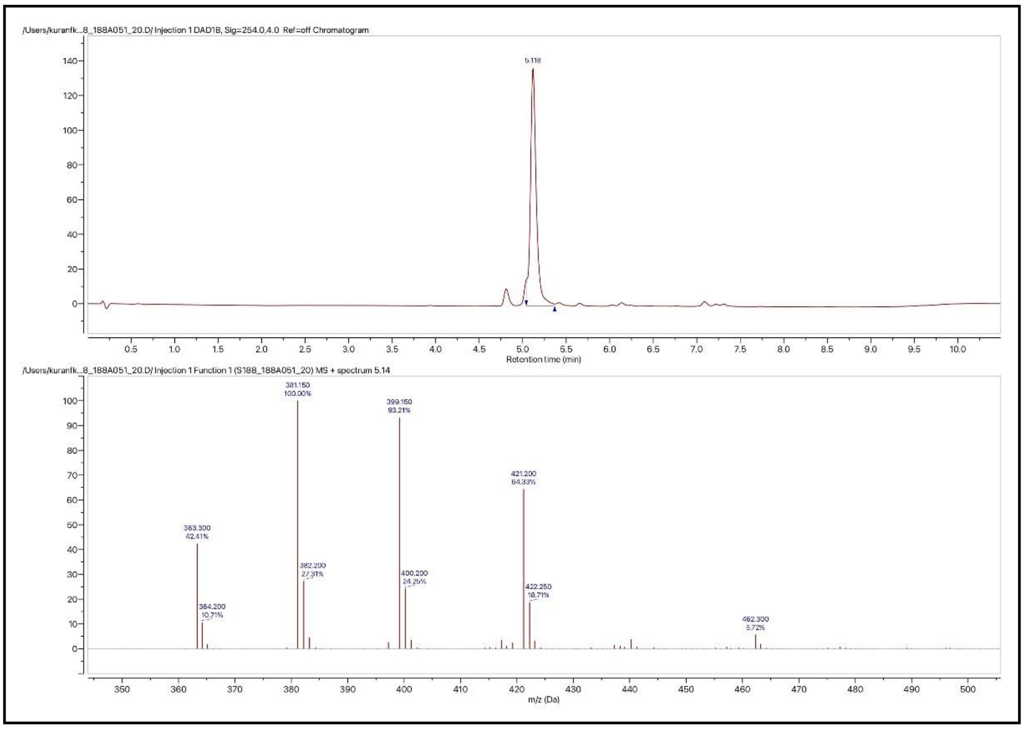

Figure S66: LC-MS spectrum of ferubungeanol A (**16**).

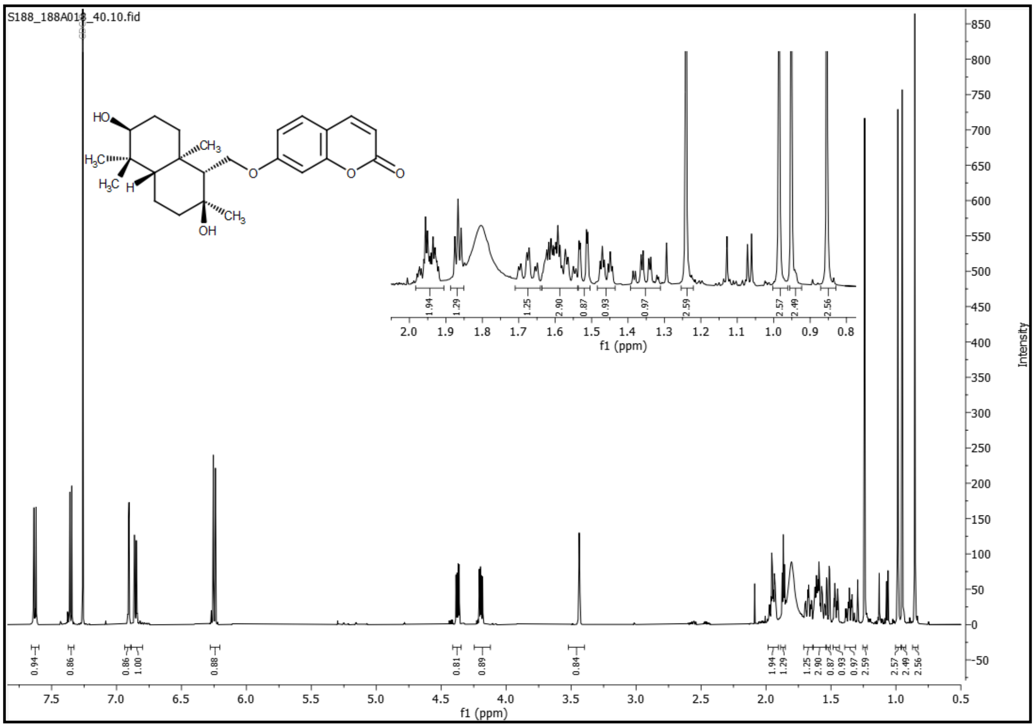

Figure S67: ^{1}H NMR spectrum (600 MHz, $CDCl_3$) of samarcandin (**17**).

Samarcandin: ^{1}H NMR (600 MHz, CDCl$_3$) δ (ppm): 7.63 (d, *J*: 9.5 Hz, 1H, C_4-H), 7.35 (d, *J*: 8.6 Hz, 1H, C_5-H), 6.91 (d, *J*: 2.4 Hz, 1H, C_8-H), 6.85 (dd, *J*: 8.6; 2.4 Hz, 1H, C_6-H), 6.25 (d, *J*: 9.5 Hz, 1H, C_3-H), 4.37 (dd, *J*: 9.8; 4.8 Hz, 1H, $C_{11'\beta}$-H), 4.19 (dd, *J*: 9.8; 5.5 Hz, 1H, $C_{11'\alpha}$-H), 3.44 (t, *J*: 2.8 Hz, 1H, $C_{3'}$-H), 1.94 (m, 2H, $C_{2'\beta}$-H, and $C_{7'\alpha}$-H), 1.87 (t, *J*: 5.1 Hz, 1H, $C_{9'}$-H), 1.67 (td, *J*: 13.3; 3.7 Hz, 1H, $C_{1'\beta}$-H), 1.64-1.54 (m, 3H, $C_{2'\alpha}$-H, $C_{6'\beta}$-H, and $C_{7'\beta}$-H), 1.52 (br d, *J*: 12.2 Hz, 1H, $C_{5'}$-H), 1.46 (dt, *J*: 13.3; 3.7 Hz, 1H, $C_{1'\alpha}$-H), 1.35 (dq, *J*: 13.2, 3.8 Hz, 1H, $C_{6'\alpha}$-H), 1.24 (s, 3H, $C_{12'}\underline{H_3}$), 0.99 (s, 3H, $C_{13'}\underline{H_3}$), 0.95 (s, 3H, $C_{15'}\underline{H_3}$), 0.85 (s, 3H, $C_{14'}\underline{H_3}$).

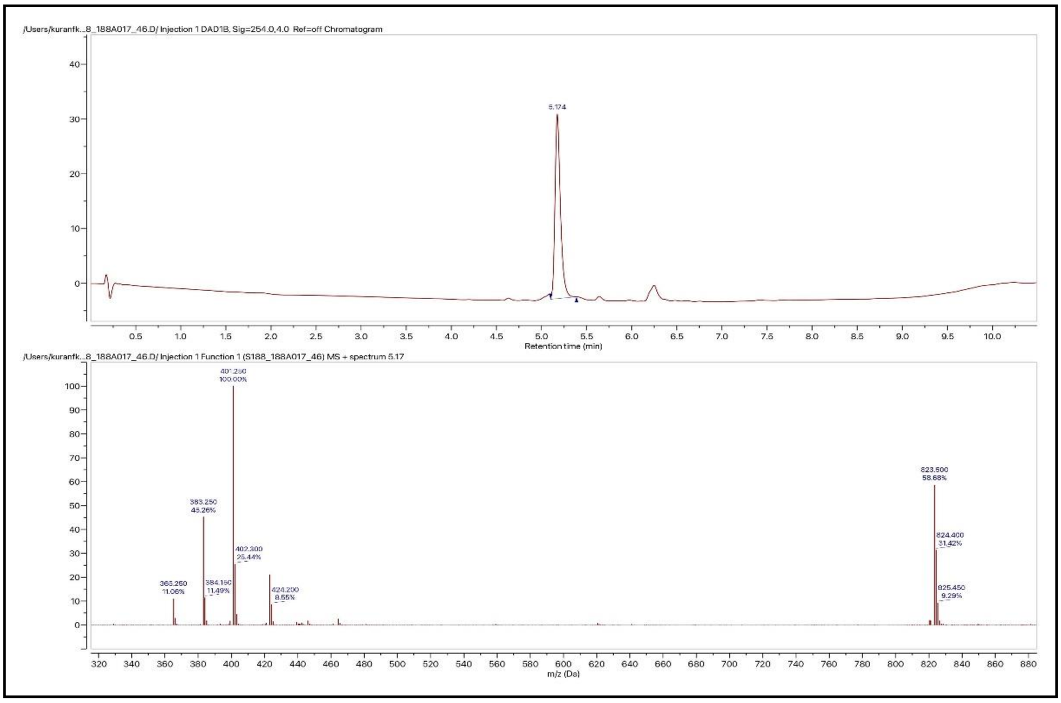

Figure S68: LC-MS spectrum of samarcandin (**17**).

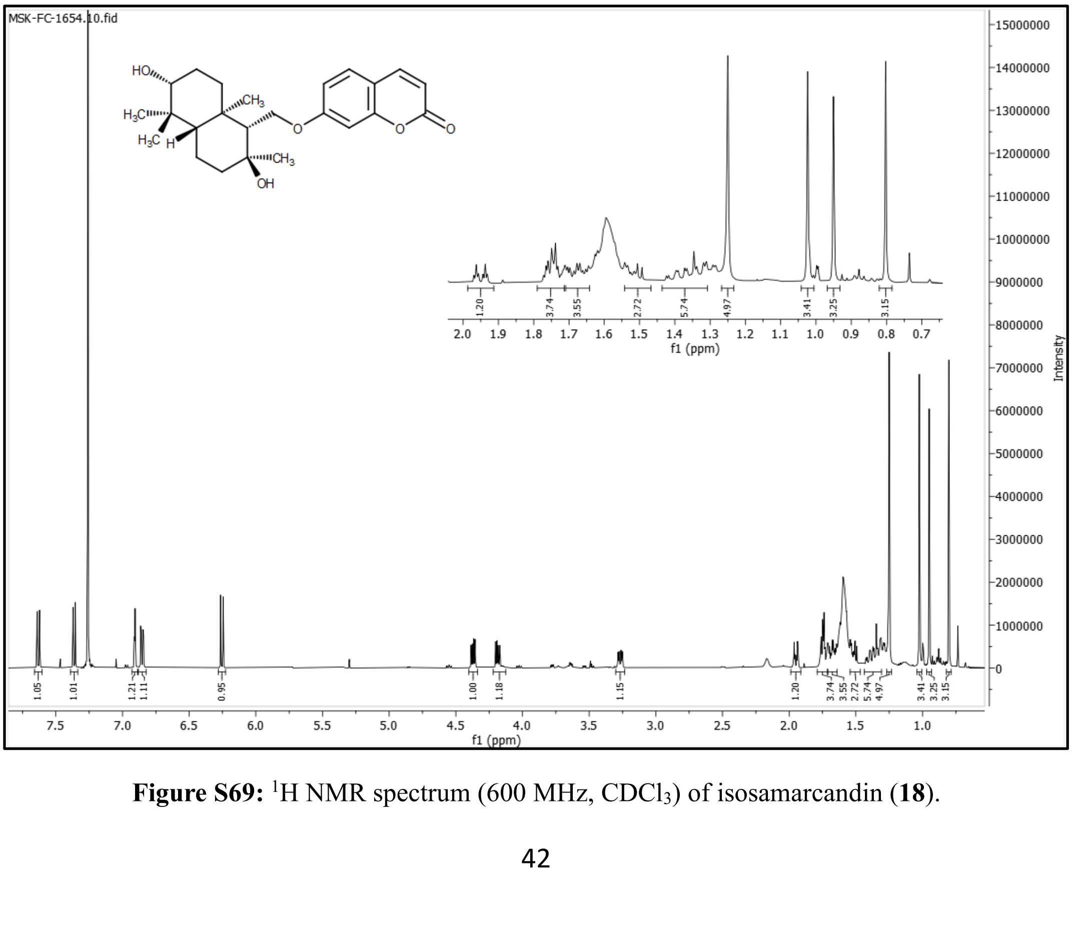

Figure S69: ^{1}H NMR spectrum (600 MHz, CDCl$_3$) of isosamarcandin (**18**).

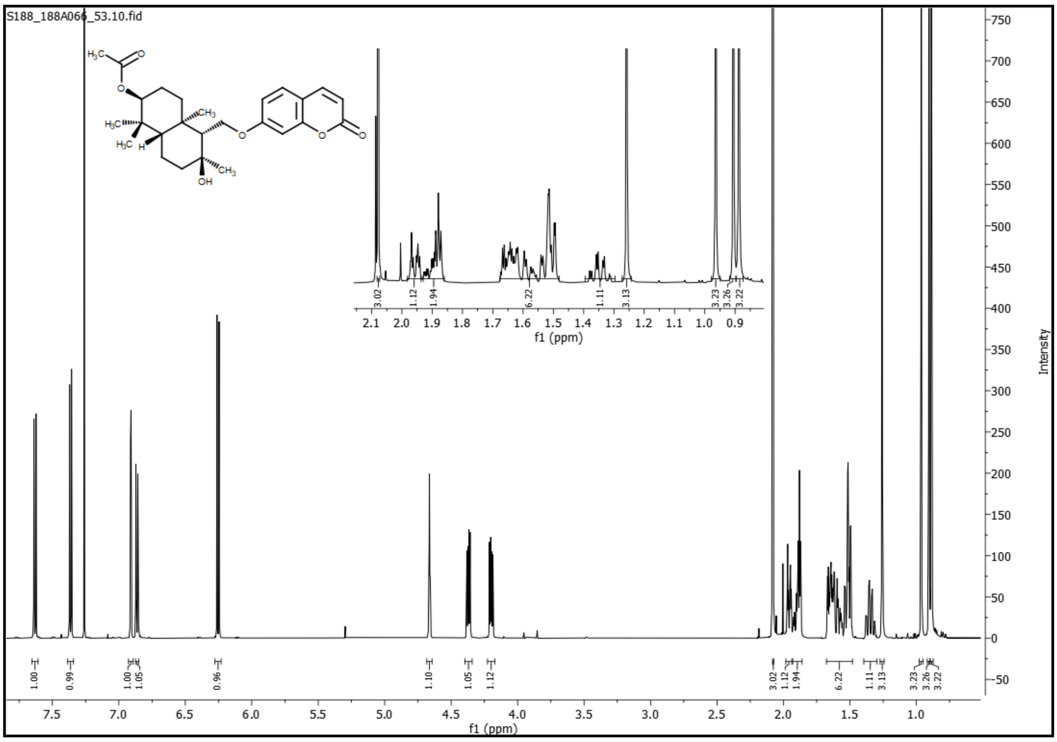

Figure S70: ^{1}H NMR spectrum (600 MHz, $CDCl_3$) of samarcandin acetate (**19**).

Samarcandin acetate: ^{1}H NMR (600 MHz, $CDCl_3$) δ (ppm): 7.63 (dd, *J*: 9.4 Hz, 1H, C_4-H), 7.36 (d, *J*: 8.6 Hz, 1H, C_5-H), 6.91 (d, *J*: 2.4 Hz, 1H, C_8-H), 6.86 (dd, *J*: 8.5, 2.42 Hz, 1H, C_6-H), 6.25 (d, *J*: 9.4 Hz, 1H, C_3-H), 4.66 (t, *J*: 2.84 Hz, 1H, $C_{3'}$-H), 4.37 (dd, *J*: 9.77; 5.08 Hz, 1H, $C_{11'\beta}$-H), 4.20 (dd, *J*: 9.77; 5.32 Hz, 1H, $C_{11'\alpha}$-H), 2.08 (s, 3H, C$\underline{H_3}$-(OAc)), 1.96 (dt, *J*: 12.7; 2.9 Hz, 1H, $C_{7'\alpha}$-H), 1.93 – 1.86 (m, 2H, $C_{2'\beta}$-H, and $C_{9'}$-H), 1.68 – 1.48 (m, 6H, $C_{1'\alpha}$-H, $C_{1'\beta}$-H, $C_{2'\alpha}$-H, $C_{5'}$-H, $C_{6'\beta}$-H, $C_{7'\beta}$-H), 1.34 (dq, *J*: 12.7; 3.9 Hz, 1H, $C_{6'\alpha}$-H), 1.26 (s, 3H, $C_{12'}\underline{H_3}$), 0.96 (s, 3H, $C_{15'}\underline{H_3}$), 0.91 (s, 3H, $C_{14'}\underline{H_3}$), 0.89 (s, 3H, $C_{13'}\underline{H_3}$).

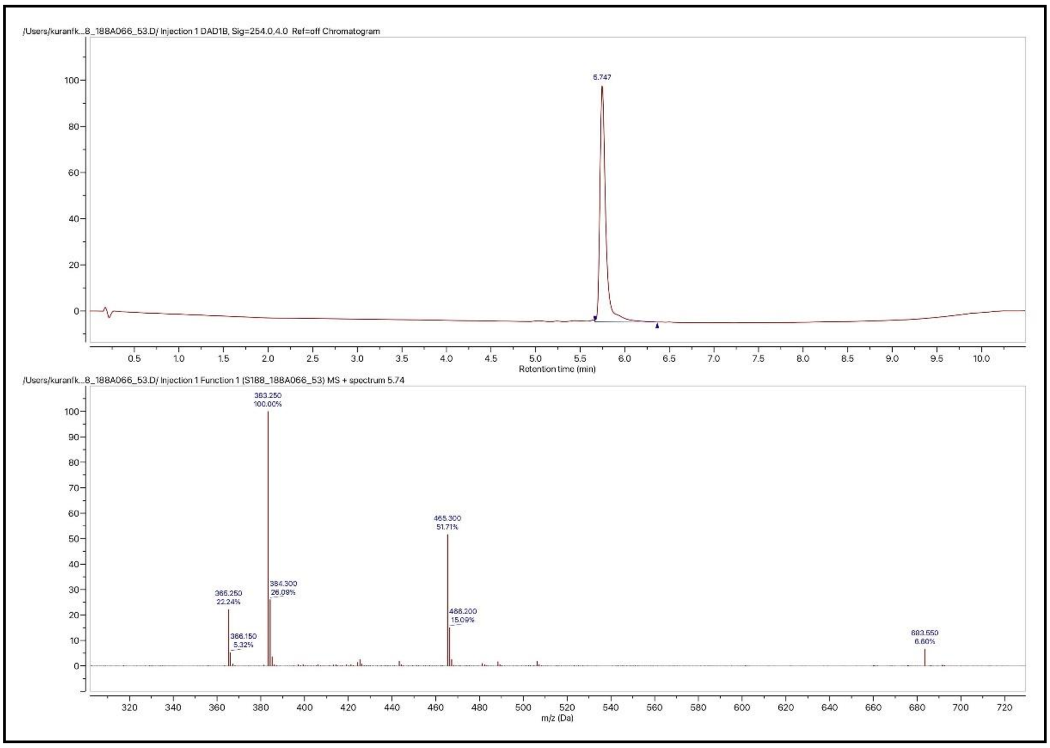

Figure S71: LC-MS spectrum of samarcandin acetate (**19**).

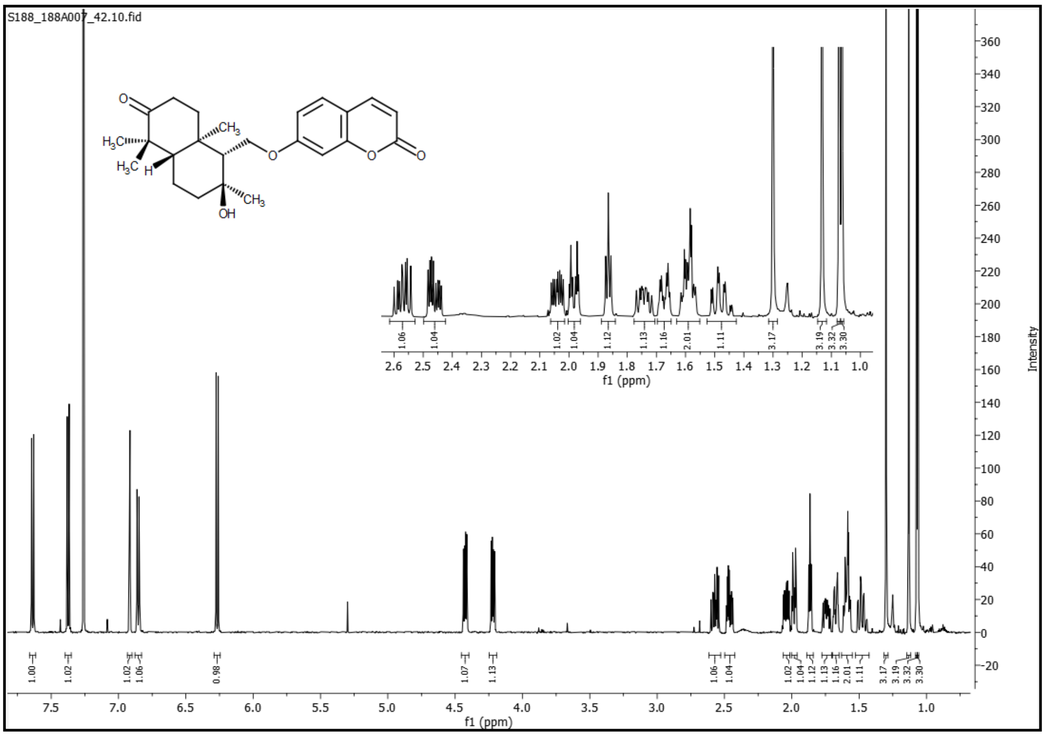

Figure S72: ^{1}H NMR spectrum (600 MHz, $CDCl_3$) of samarcandone (**20**).

Samarcandone: ^{1}H NMR (600 MHz, $CDCl_3$) δ (ppm): 7.64 (d, *J*: 9.4 Hz, 1H, C_4-H), 7.37 (d, *J*: 8.6 Hz, 1H, C_5-H), 6.92 (d, *J*: 2.4 Hz, 1H, C_8-H), 6.85 (dd, *J*: 8.6; 2.4 Hz, 1H, C_6-H), 6.27 (d, *J*: 9.4 Hz, 1H, C_3-H), 4.43 (dd, *J*: 9.9; 5.2 Hz, 1H, $C_{11'\beta}$-H), 4.22 (dd, *J*: 9.9; 5.5 Hz, 1H, $C_{11'\alpha}$-H), 2.57 (ddd, *J*: 16.0; 10.8; 7.3 Hz, 1H, $C_{2'\alpha}$-H), 2.46 (ddd, *J*: 16.0; 7.0; 3.9 Hz, 1H, $C_{2'\beta}$-H), 2.04 (ddd, *J*: 13.54; 7.31; 3.92 Hz, 1H, $C_{1'\alpha}$-H), 1.98 (dt, *J*: 12.66; 3.13 Hz, 1H, $C_{7'\alpha}$-H), 1.86 (t, *J*: 5.3 Hz, 1H, $C_{9'}$-H), 1.74 (ddd, *J*: 13.53; 10.94; 7.04 Hz, 1H, $C_{1'\beta}$-H), 1.67 (dq, *J*: 13.52; 3.14 Hz, 1H, $C_{6'\alpha}$-H), 1.63-1.55 (m, 2H, $C_{5'}$-H, and $C_{7'\beta}$-H), 1.48 (qd, *J*: 13.4; 3.2 Hz, 1H, $C_{6'\beta}$-H), 1.30 (s, 3H, $C_{12'}\underline{H_3}$), 1.13 (s, 3H, $C_{13'}\underline{H_3}$), 1.07 (s, 3H, $C_{15'}\underline{H_3}$), 1.06 (s, 3H, $C_{14'}\underline{H_3}$).

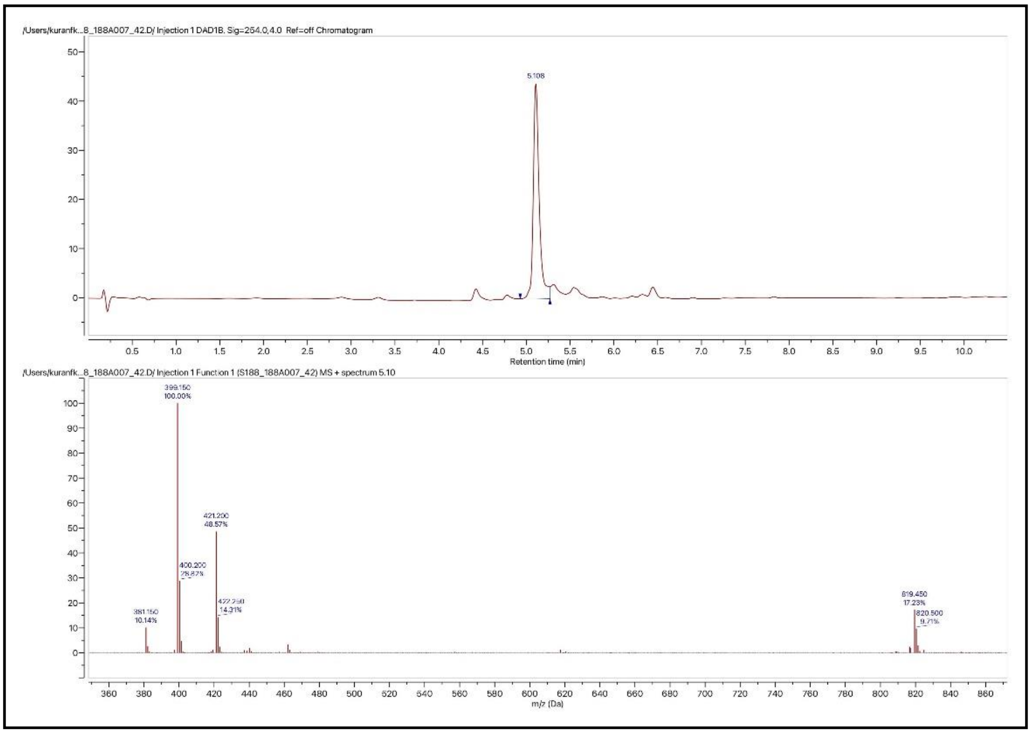

Figure S73: LC-MS spectrum of samarcandone (**20**).

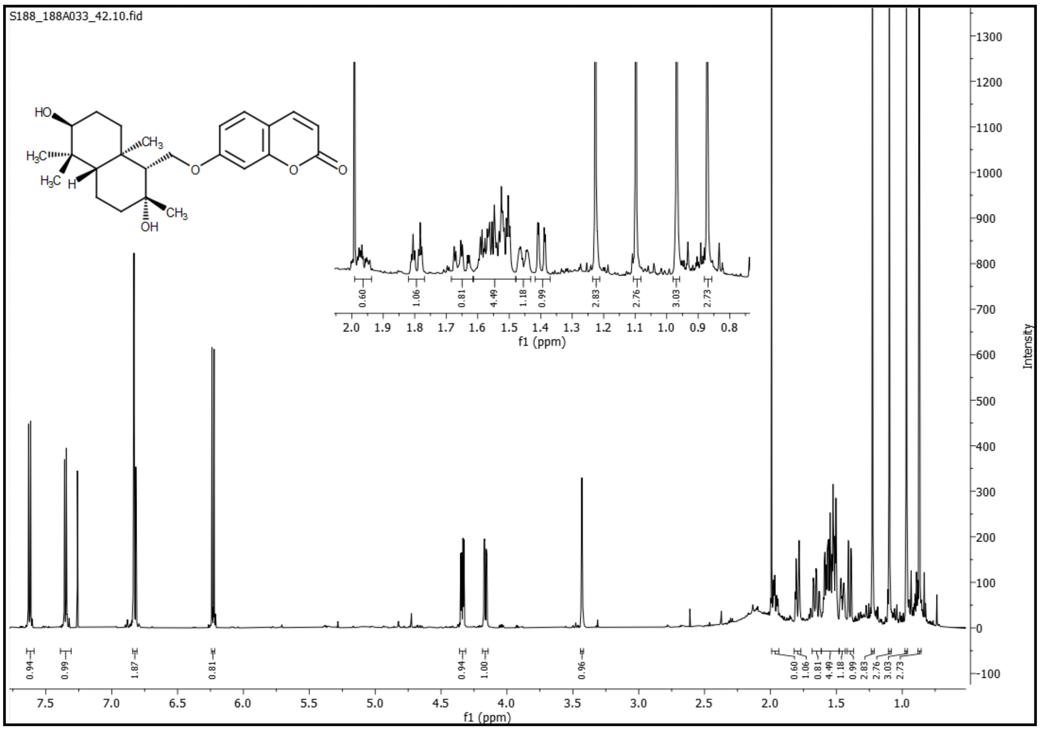

Figure S74: ^{1}H NMR spectrum (600 MHz, $CDCl_3$) of feshurin (**21**).

Feshurin: ^{1}H NMR (600 MHz, $CDCl_3$) δ (ppm): 7.62 (d, *J*: 9.5 Hz, 1H, C_4-H), 7.35 (d, *J*: 8.3 Hz, 1H, C_5-H), 6.84-6.81 (m, 2H, C_6-H, and C_8-H), 6.23 (d, *J*: 9.5 Hz, 1H, C_3-H), 4.34 (dd, *J*: 10.0; 4.0 Hz, 1H, $C_{11'\beta}$-H), 4.16 (dd, *J*: 10.0; 3 Hz, 1H, $C_{11'\alpha}$-H), 3.43 (t, *J*: 2.8 Hz, 1H, $C_{3'}$.H), 1.99-1.94 (m, 1H, $C_{2'\beta}$-H), 1.79 (dt, *J*: 13.6; 3.05 Hz, 1H, $C_{7'}$-H), 1.65 (td, *J*: 13.0; 3.3 Hz, 1H, $C_{1'\beta}$-H), 1.61-1.48 (m, 5H, $C_{2'\alpha}$-H, $C_{5'}$-H, $C_{6'\beta}$-H, $C_{8'}$-H, and $C_{9'}$-H), 1.45 (dt, *J*: 13,0; 4,0 Hz, 1H, $C_{1'\alpha}$-H), 1.40 (br dd *J*: 12.3; 2.11, 1H, $C_{6'\alpha}$-H), 1.23 (s, 3H, $C_{12'}\underline{H_3}$), 1.1 (s, 3H, $C_{13'}\underline{H_3}$), 0.97 (s, 3H, $C_{15'}\underline{H_3}$), 0.87 (s, 3H, $C_{14'}\underline{H_3}$).

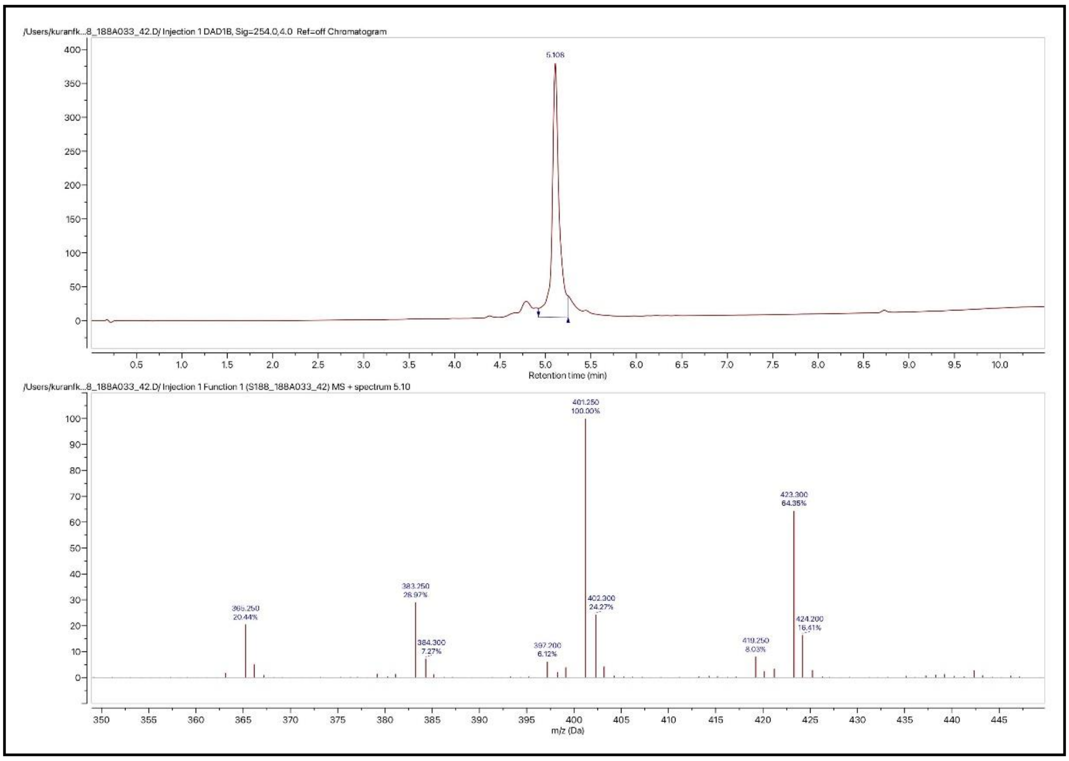

Figure S75: LC-MS spectrum of feshurin (**21**).

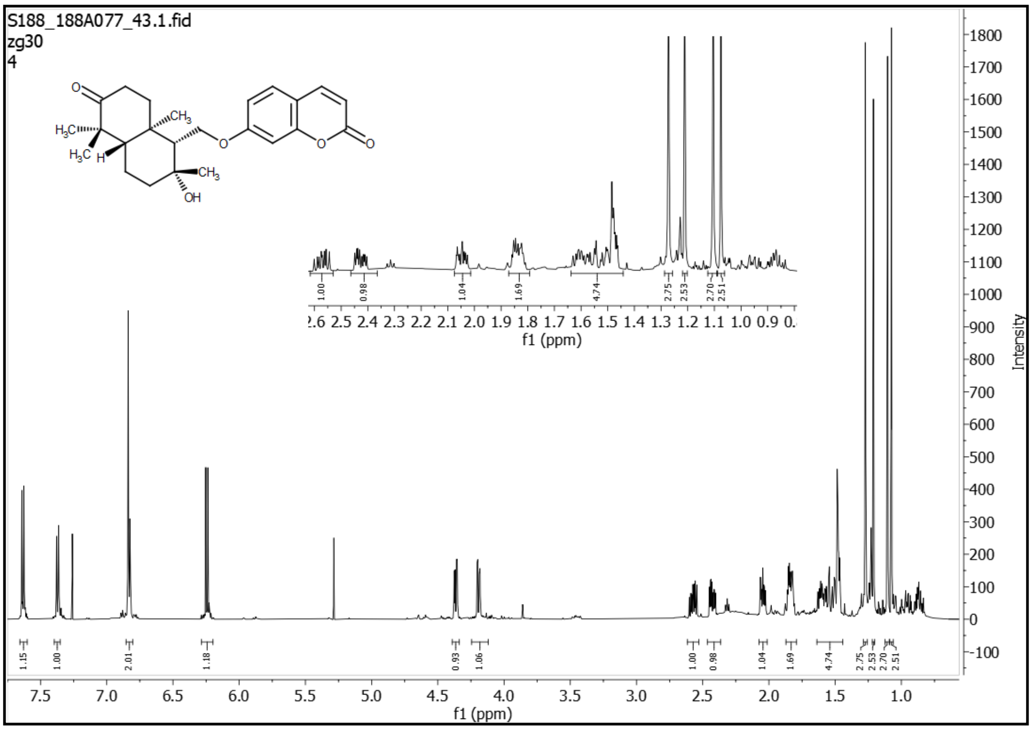

Figure S76: ^{1}H NMR spectrum (600 MHz, $CDCl_3$) of feshurone (nevskone) (**22**).

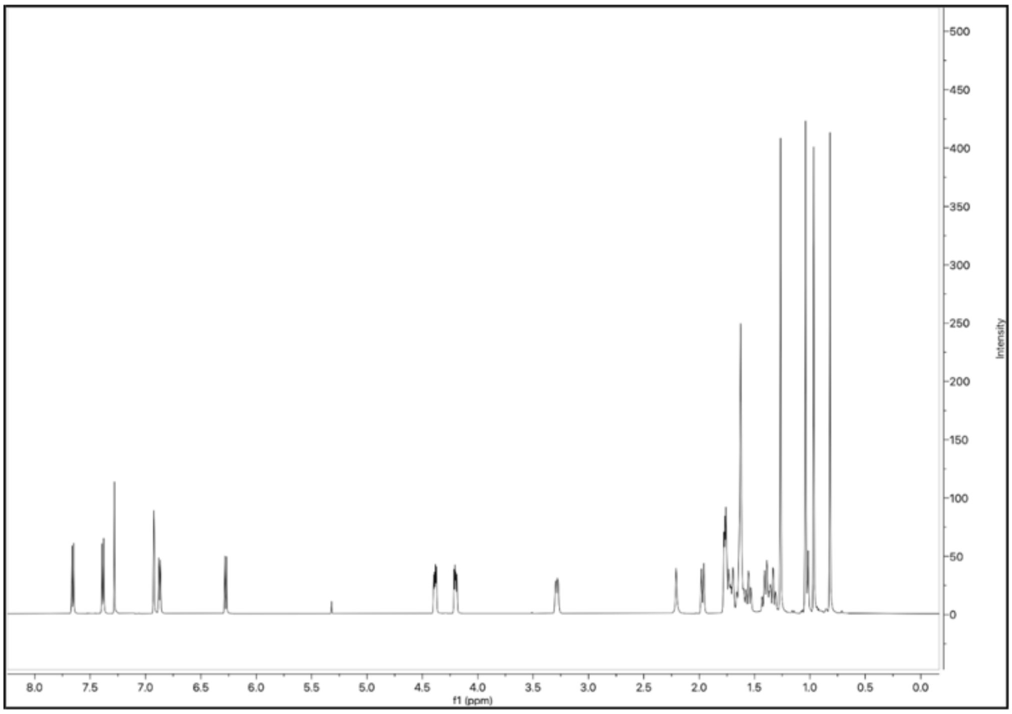

Figure S77: ^{1}H NMR spectrum (600 MHz, $CDCl_3$) of nevskin (**23**).

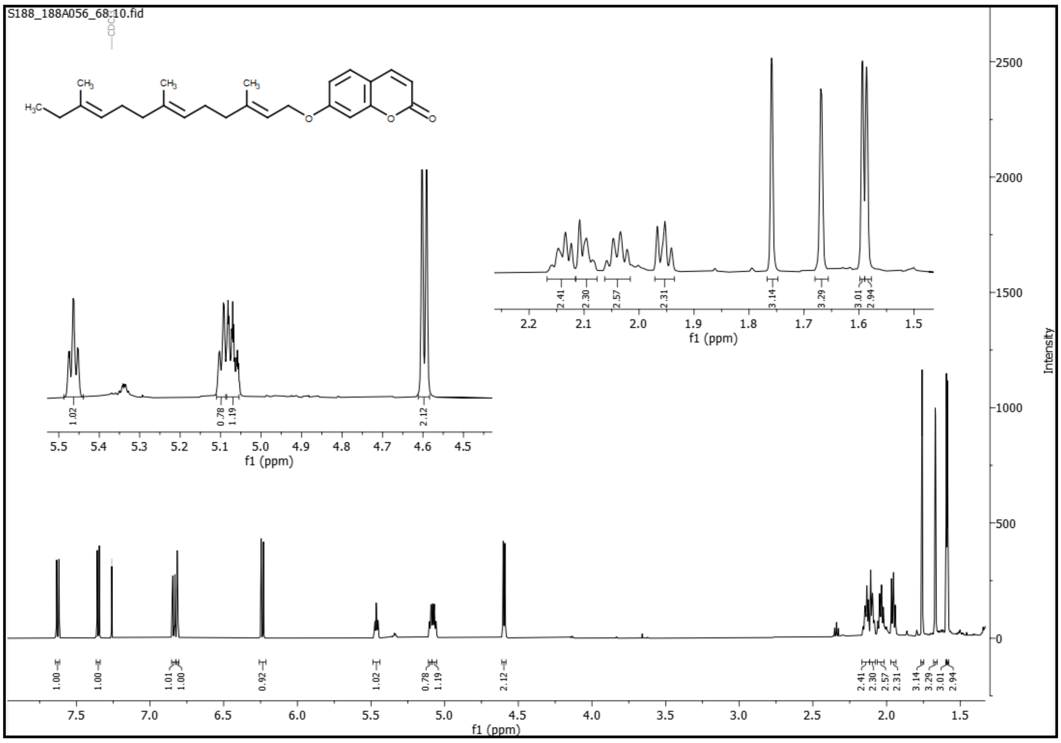

Figure S78: ^{1}H NMR spectrum (600 MHz, $CDCl_3$) of umbelliprenin (**24**).

Umbelliprenin: ^{1}H NMR (600 MHz, $CDCl_3$) δ (ppm): 7.63 (d, *J*: 9.4 Hz, 1H, C_4-H), 7.35 (d, *J*: 8.5 Hz, 1H, C_5-H), 6.84 (dd, *J*: 8.5; 2.4 Hz, 1H, C_6-H), 6.81 (d, *J*: 2.4 Hz, 1H, C_8-H), 6.24 (d, *J*: 9.4 Hz, 1H, C_3-H), 5.46 (td, *J*: 6.6; 1.3 Hz, 1H, $C_{2'}$-H), 5.10 (dq, *J*: 6.6; 1.3 Hz, 1H, $C_{10'}$-H), 5.07 (ddq, *J*: 8.5; 5.6;1.4 Hz, 1H, $C_{6'}$-H), 4.6 (d, *J*: 6.6 Hz, 2H, $C_{1'}\underline{H_2}$), 2.16 – 2.12 (m, 2H, $C_{5'}\underline{H_2}$), 2.12 – 2.08 (m, 2H, $C_{4'}\underline{H_2}$), 2.04 (q, *J*: 7.5 Hz, 2H, $C_{8'}\underline{H_2}$), 1.95 (dd, *J*: 9.11; 6.17 Hz, 2H, $C_{9'}\underline{H_2}$), 1.76 (s, 3H, $C_{12'}\underline{H_3}$), 1.67 (s, 3H, $C_{15'}\underline{H_3}$), 1.59 (s, 3H, $C_{13'}\underline{H_3}$), 1.58 (s, 3H, $C_{14'}\underline{H_3}$).

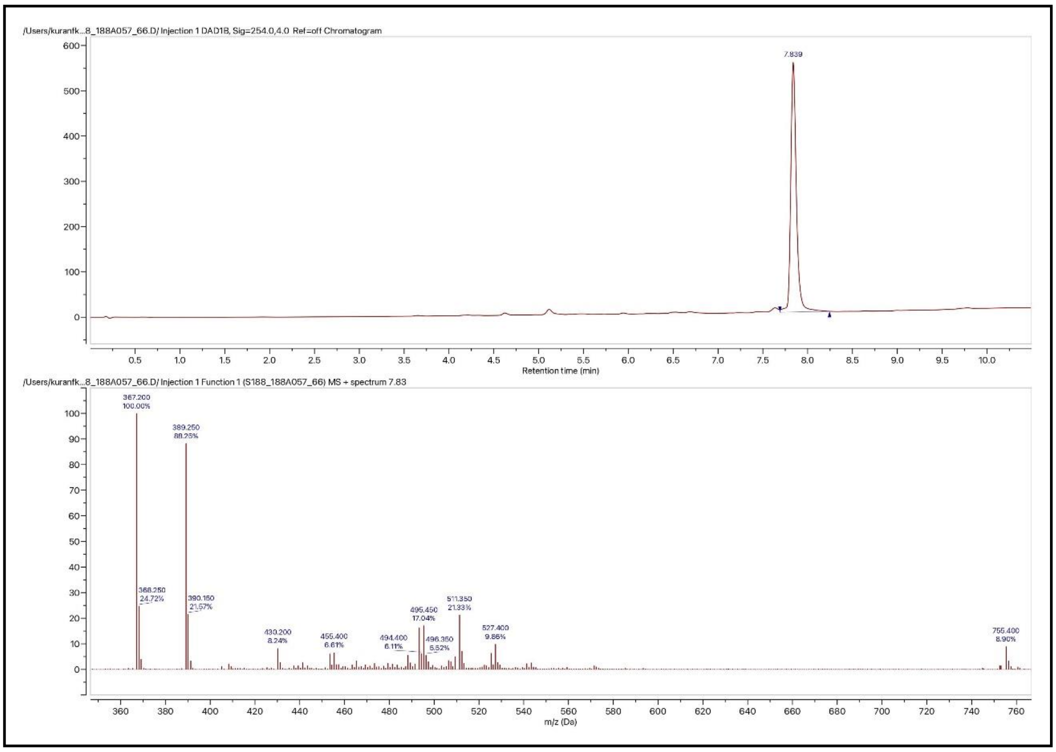

Figure S79: LC-MS spectrum of umbelliprenin (**24**).

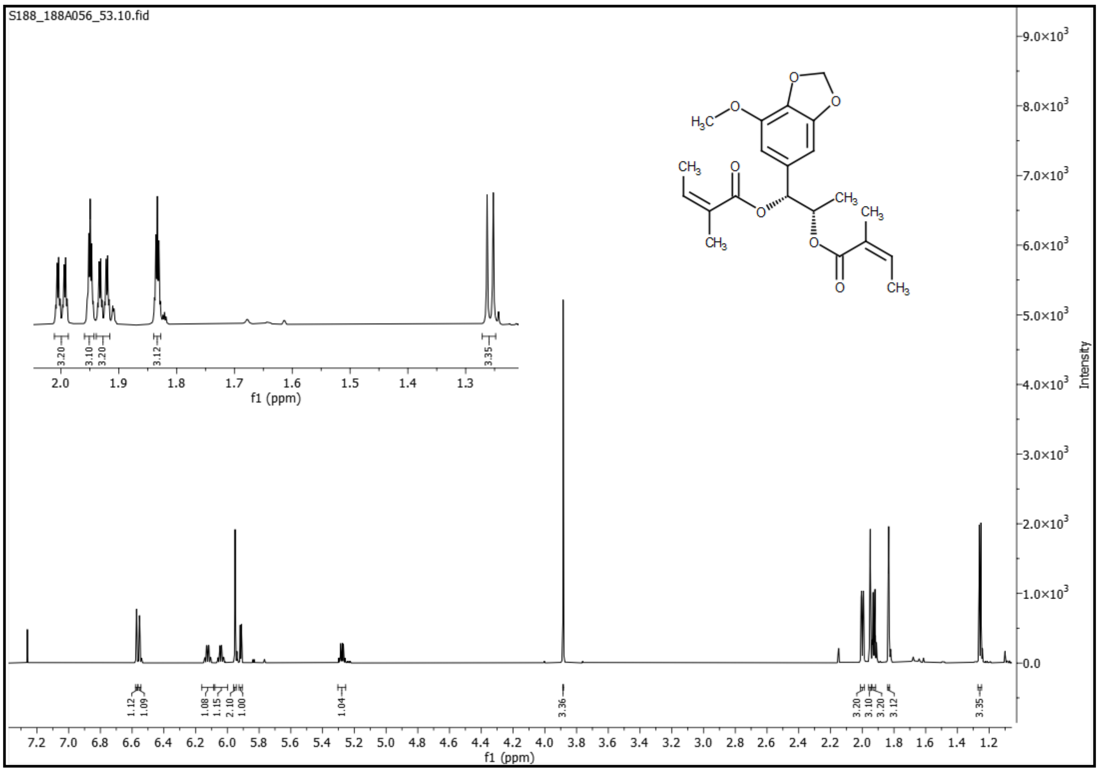

Figure S80: ^{1}H NMR spectrum (600 MHz, $CDCl_3$) of 2-epilaserine (**25**).

2-Epilaserine: ^{1}H NMR (600 MHz, $CDCl_3$) δ (ppm): 6.57 (d, *J*: 1.4 Hz, 1H, $C_{2'}$-H), 6.55 (d, *J*: 1.4 Hz, 1H, $C_{6'}$-H), 6.13 (qq, *J*: 7.24; 1.46 Hz, 1H, $C_{3''}$H), 6.04 (qq, *J*: 7.25; 1.5 Hz, 1H, $C_{3'''}$H), 5.96 (s, 2H, $C_{7'}\underline{H_2}$), 5.92 (d, *J*: 4.41 Hz, 1H, C_1-H), 5.28 (qd, *J*: 6.4; 4.4 Hz, 1H, C_2-H), 3.89 (s, 3H, $C_{3'}\underline{H_3}$), 2.00 (dq, *J*: 7.26; 1.55 Hz, 3H, $C_{4''}\underline{H_3}$), 1.95 (t, *J*: 1.5 Hz, 3H, $C_{5''}\underline{H_3}$), 1.93 (dq, *J*: 7.2; 1.5 Hz, 3H, $C_{4'''}H_3$), 1.84 (t, *J*: 1.5 Hz, 3H, $C_{5'''}\underline{H_3}$), 1.26 (d, *J*: 6.4 Hz, 3H, $C_3\underline{H_3}$).

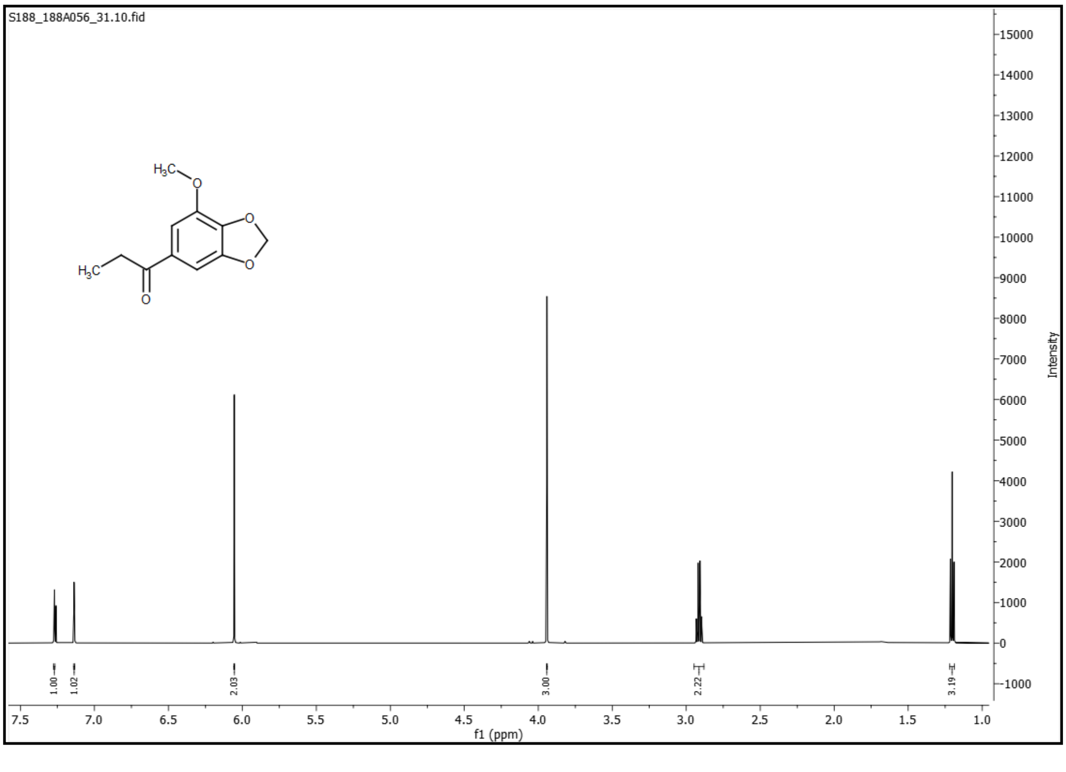

Figure S81: ^{1}H NMR spectrum (600 MHz, $CDCl_3$) of crocatone (**26**).

Crocatone: ^{1}H NMR (600 MHz, $CDCl_3$) δ (ppm): 7.27 (d, *J*: 1.4 Hz, 1H, C_2-H), 7.14 (d, *J*: 1.4 Hz, 1H, C_6-H), 6.05 (s, 2H, O-C$\underline{H_2}$-O), 3.94 (s, 3H, C_3-OC$\underline{H_3}$), 2.91 (q, *J*: 7.2 Hz, 2H, $C_{2'}\underline{H_2}$), 1.20 (t, *J*: 7.25 Hz, 3H, $C_{3'}\underline{H_3}$).

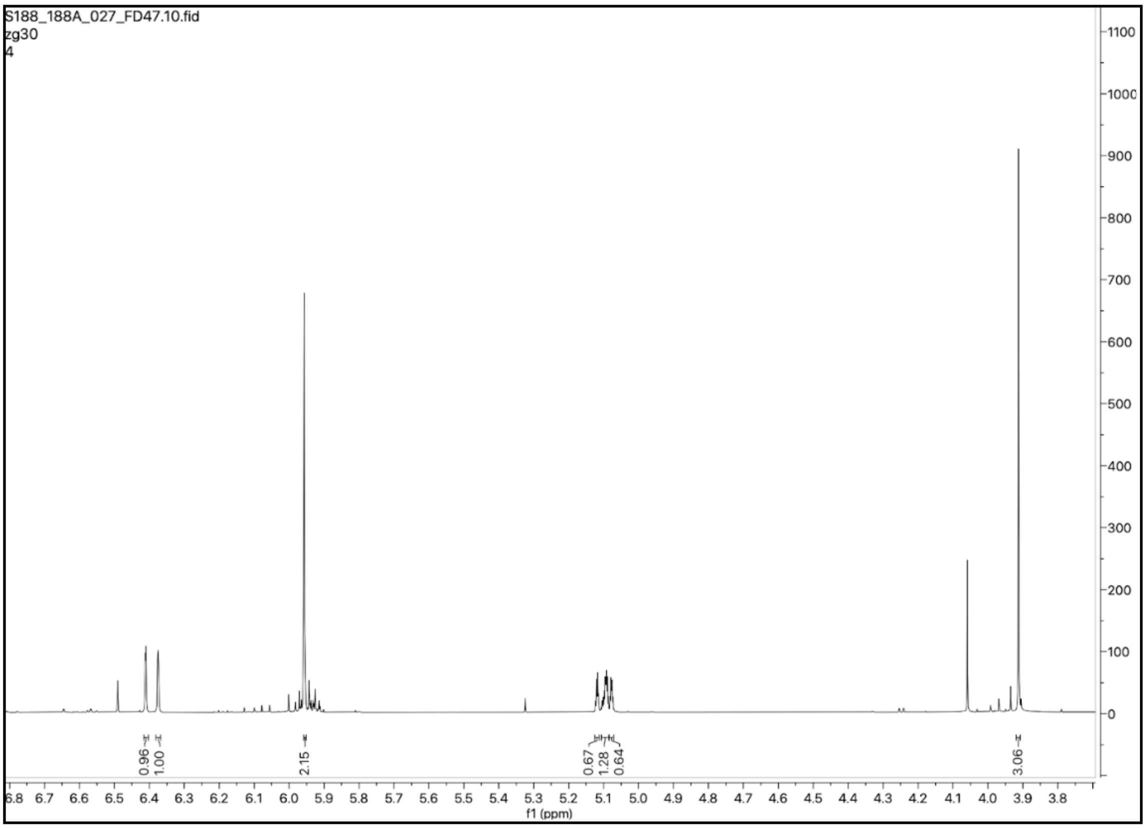

Figure S82: ^{1}H NMR spectrum (600 MHz, $CDCl_3$) of myristicin (**27**).

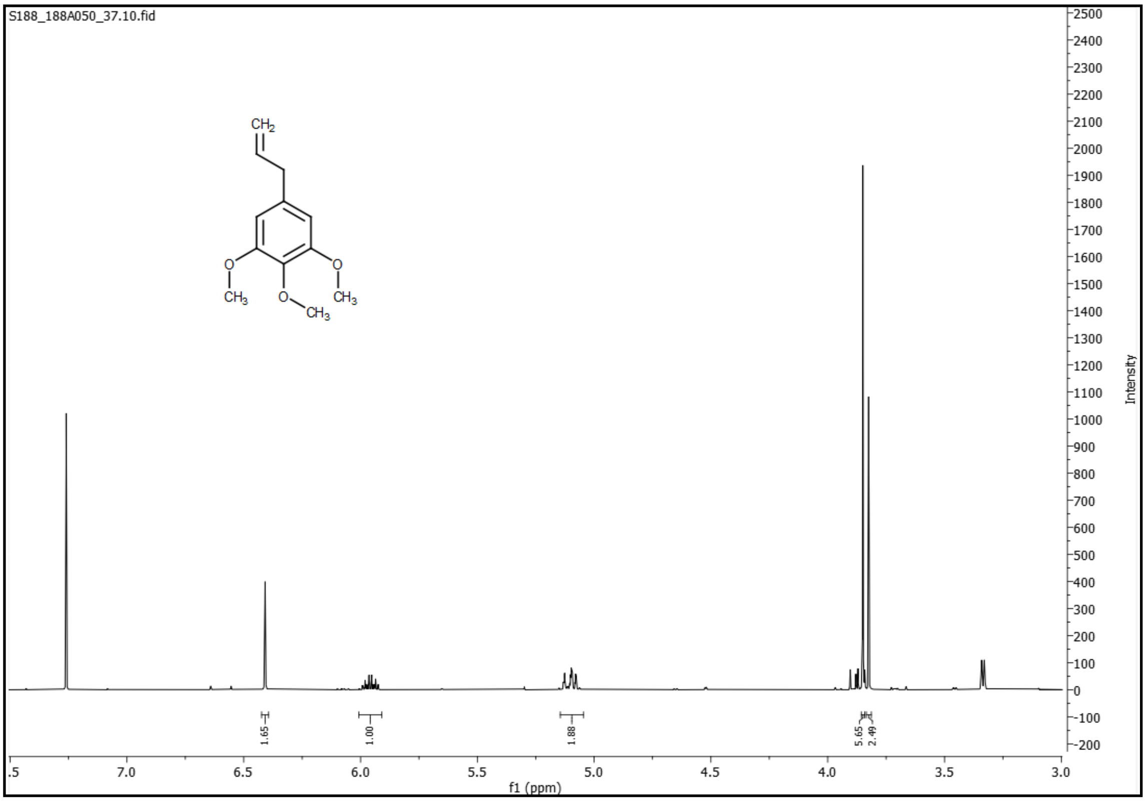

Figure S83: ^{1}H NMR spectrum (600 MHz, $CDCl_3$) of elemicin (**28**).

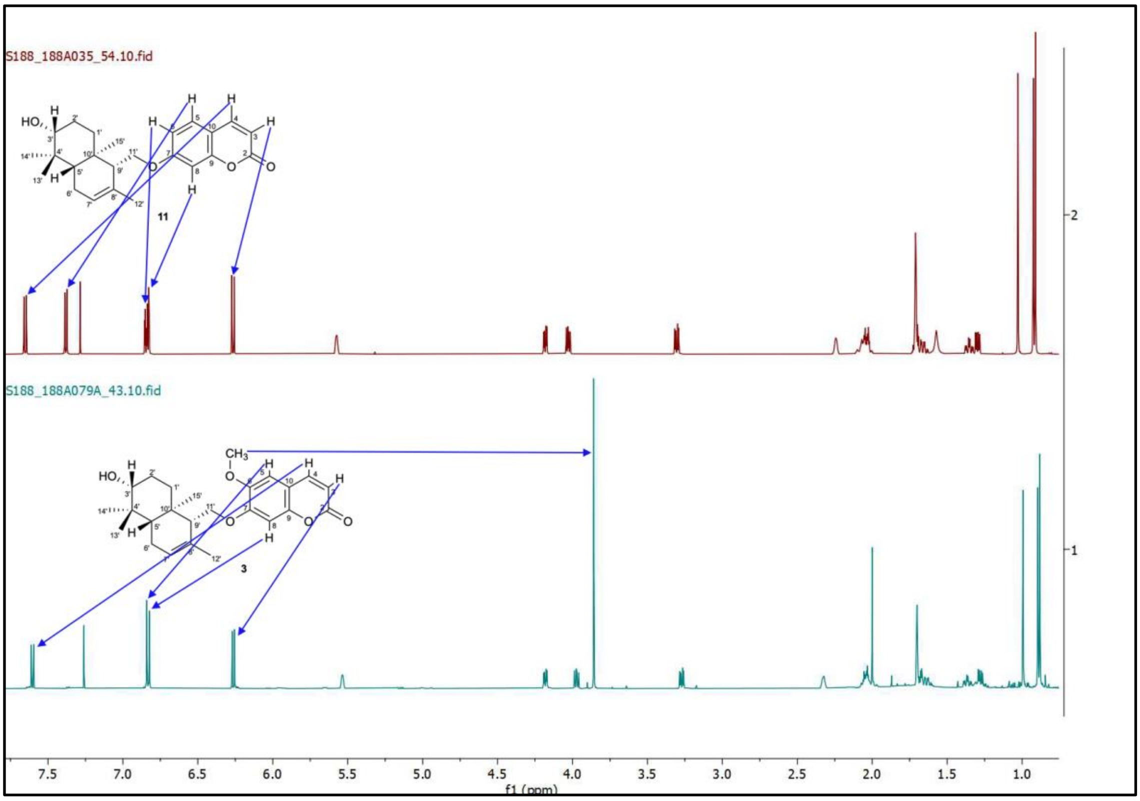

Figure S84: Stacked ^{1}H NMR spectra of feselol (**11**, top) and druscoferol (**3**, bottom).

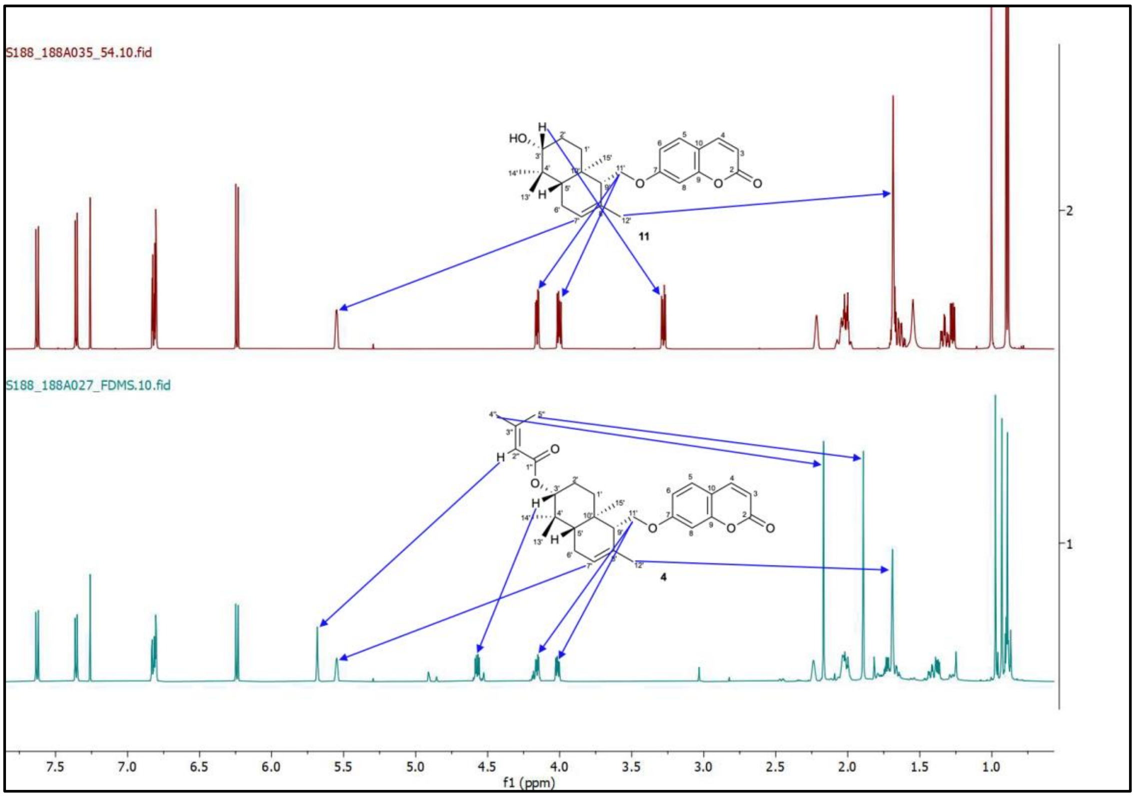

Figure S85: Stacked ^{1}H NMR spectra of feselol (**11**, top) and feselol senecioate (**4**, bottom).

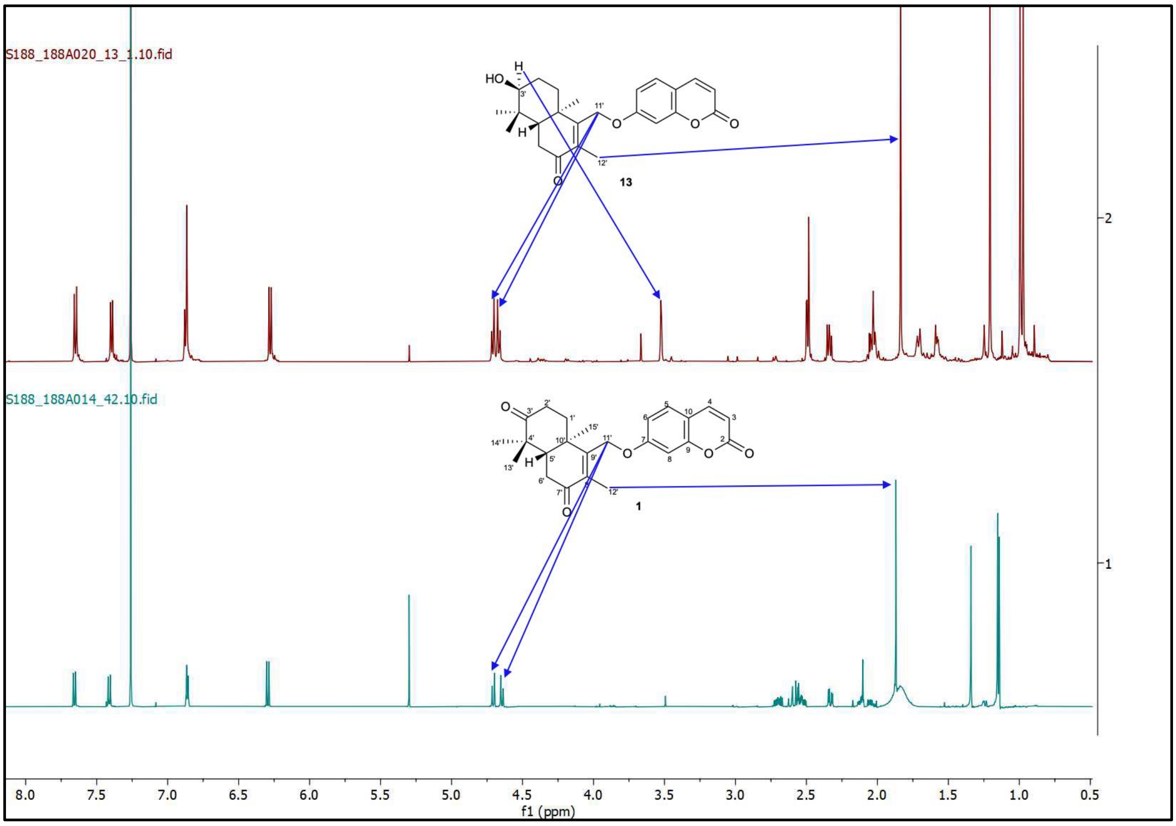

Figure S86: Stacked ^{1}H NMR spectra of druferone (**1**, bottom) and ferubungeanol G (**13**, top).

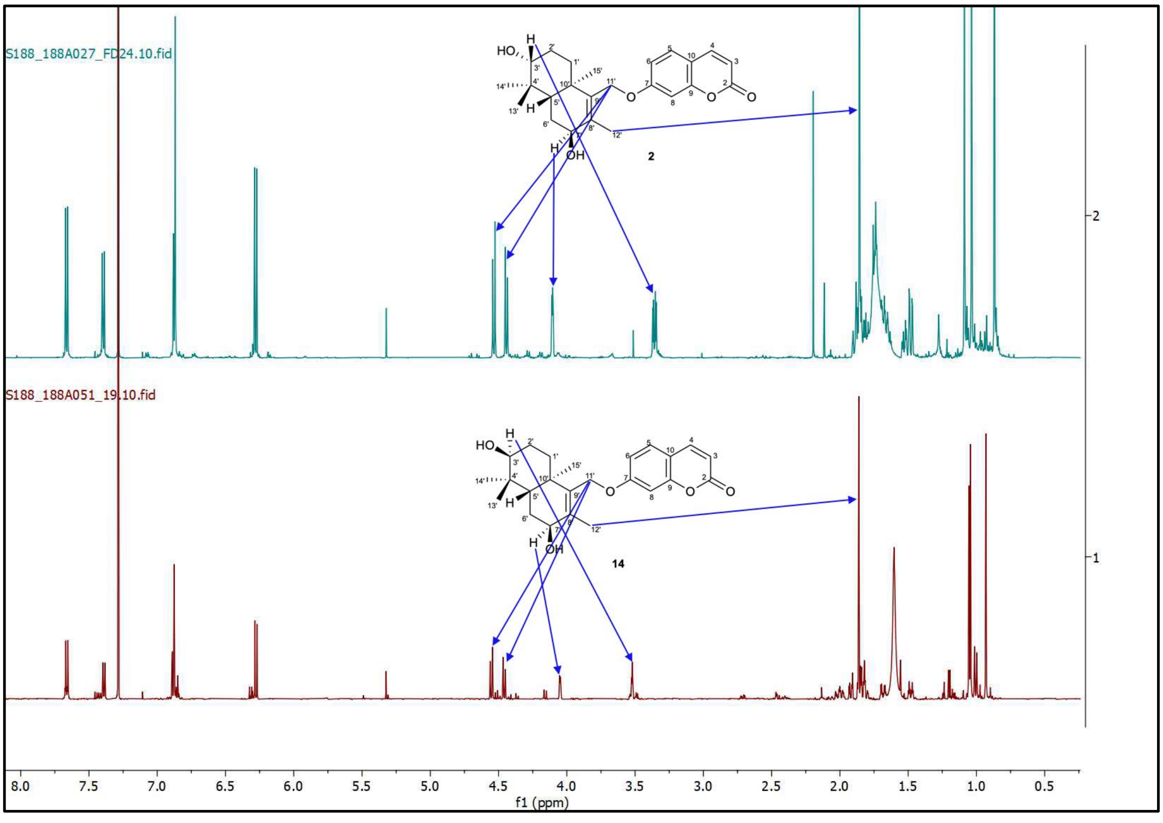

Figure S87: Stacked ^{1}H NMR spectra of druferol (**2**, top) and samarcandicin K (**14**, bottom).

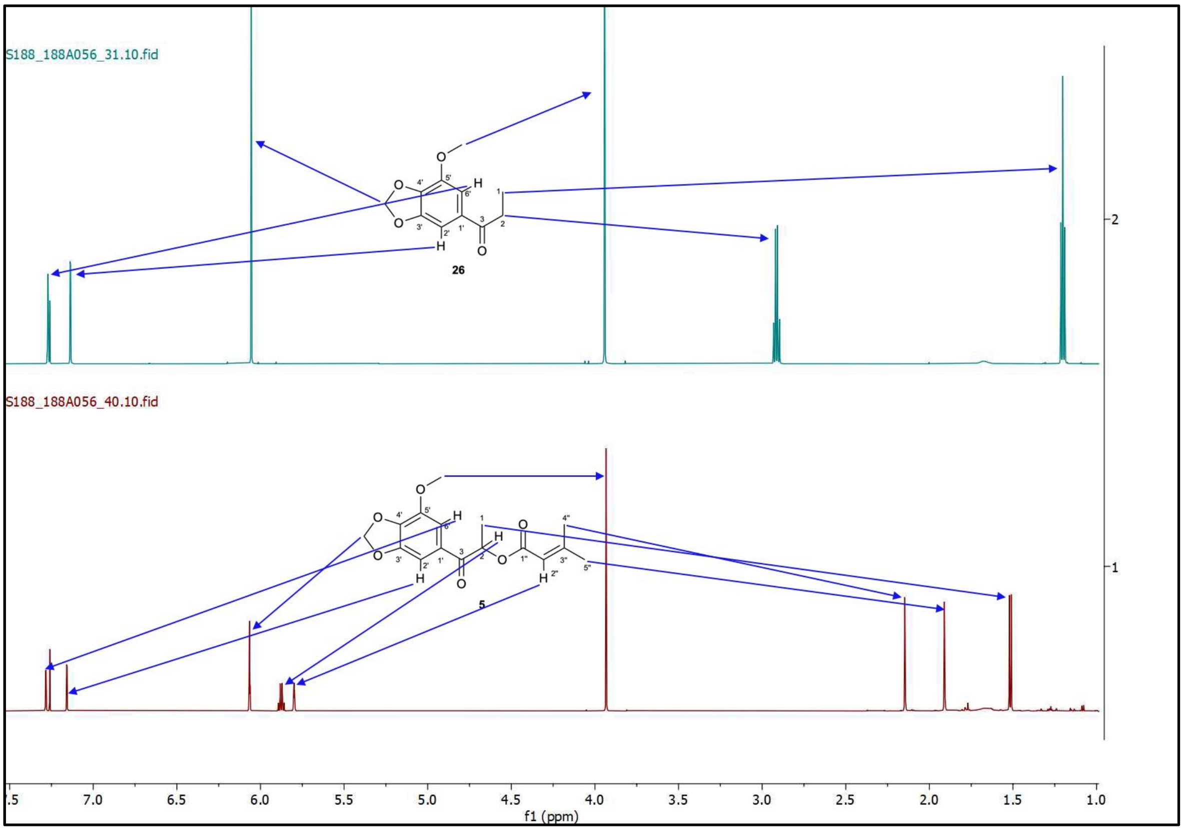

Figure S88: Stacked ^{1}H NMR spectra of crocatone (**26**, top) and drudeanone (**5**, bottom).

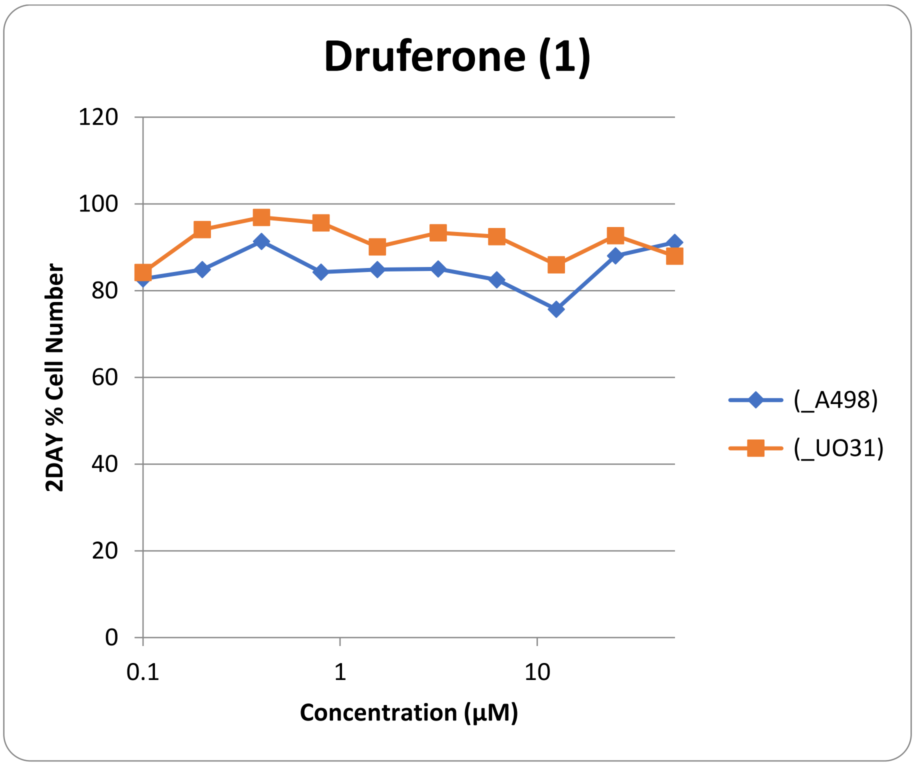

Figure S89: Concentration-dependent cytotoxic effects of druferone (**1**) on A498 and UO31 cell lines.

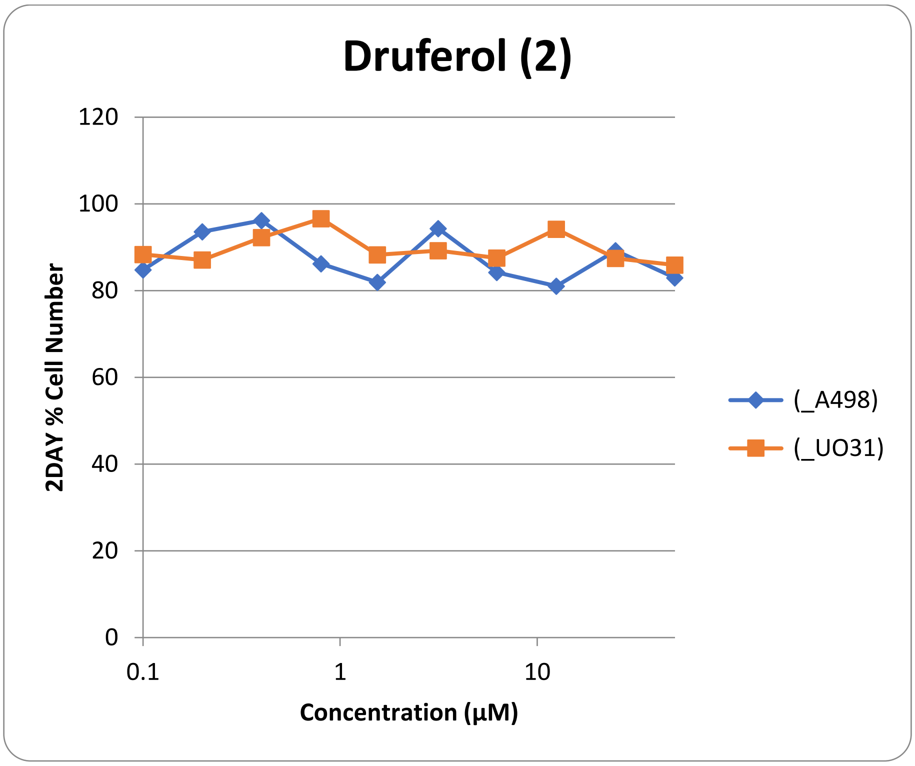

Figure S90: Concentration-dependent cytotoxic effects of druferol (**2**) on A498 and UO31 cell lines.

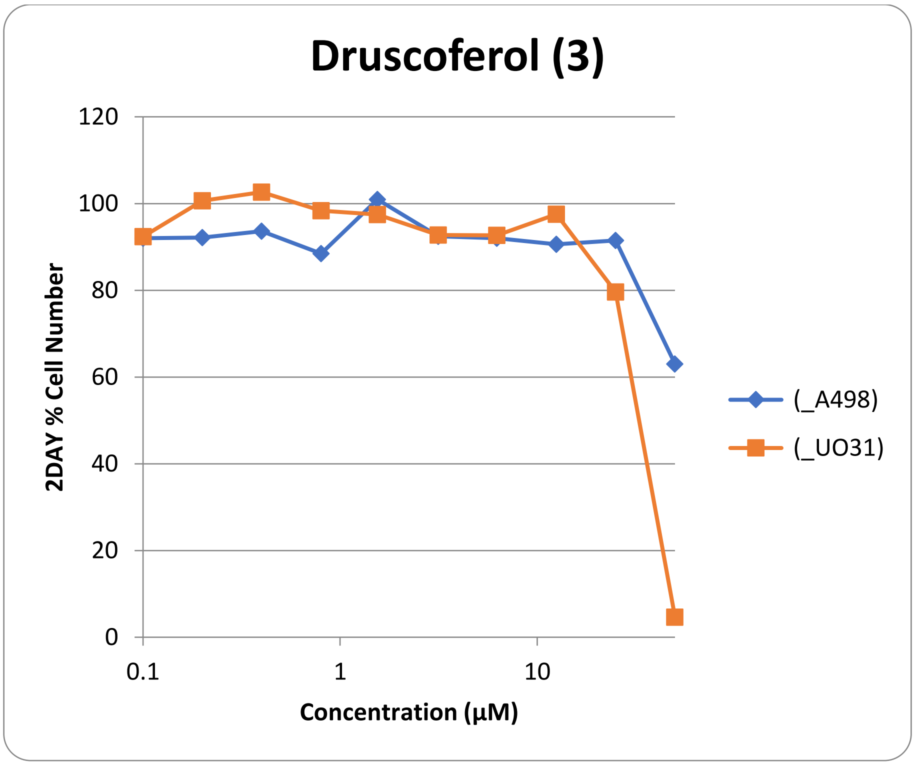

Figure S91: Concentration-dependent cytotoxic effects of druscoferol (**3**) on A498 and UO31 cell lines.

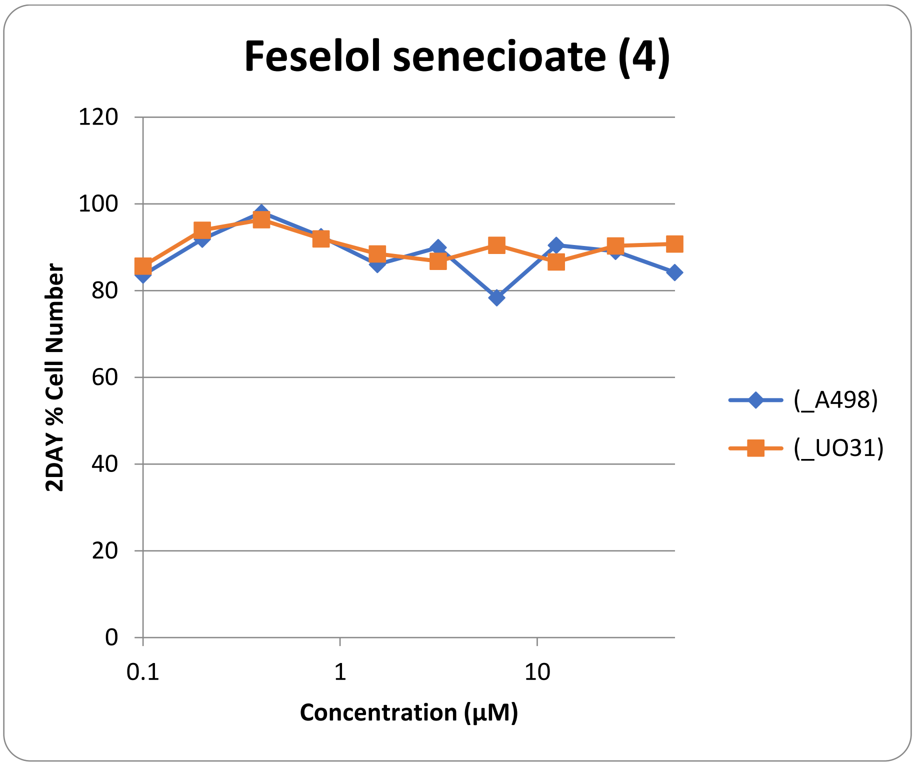

Figure S92: Concentration-dependent cytotoxic effects of feselol senecioate (**4**) on A498 and UO31 cell lines.

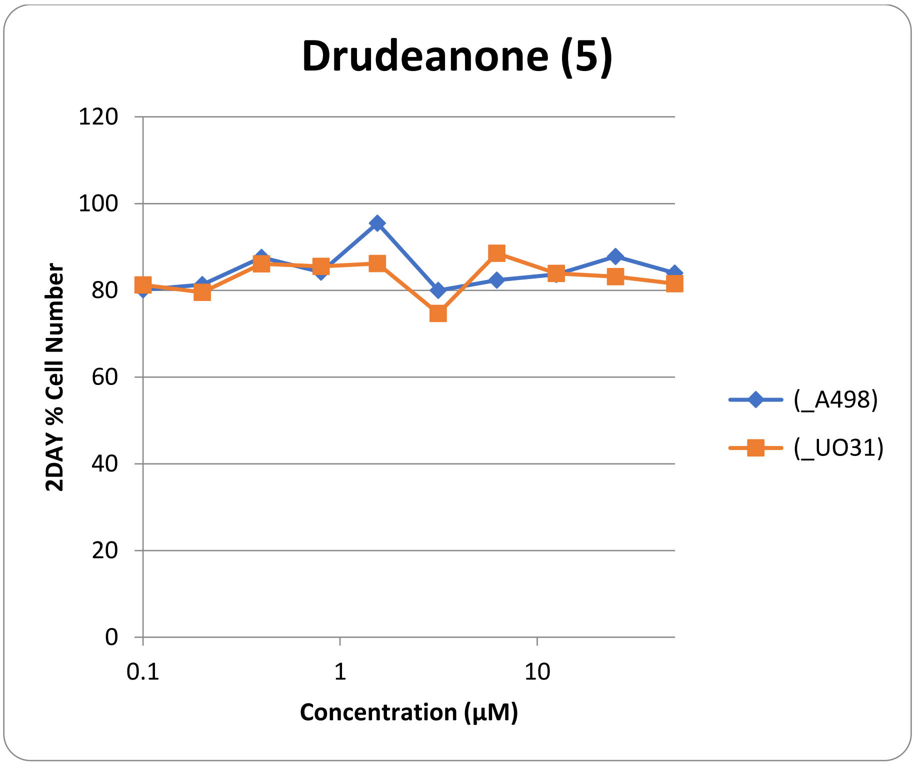

Figure S93: Concentration-dependent cytotoxic effects of drudeanone (**5**) on A498 and UO31 cell lines.

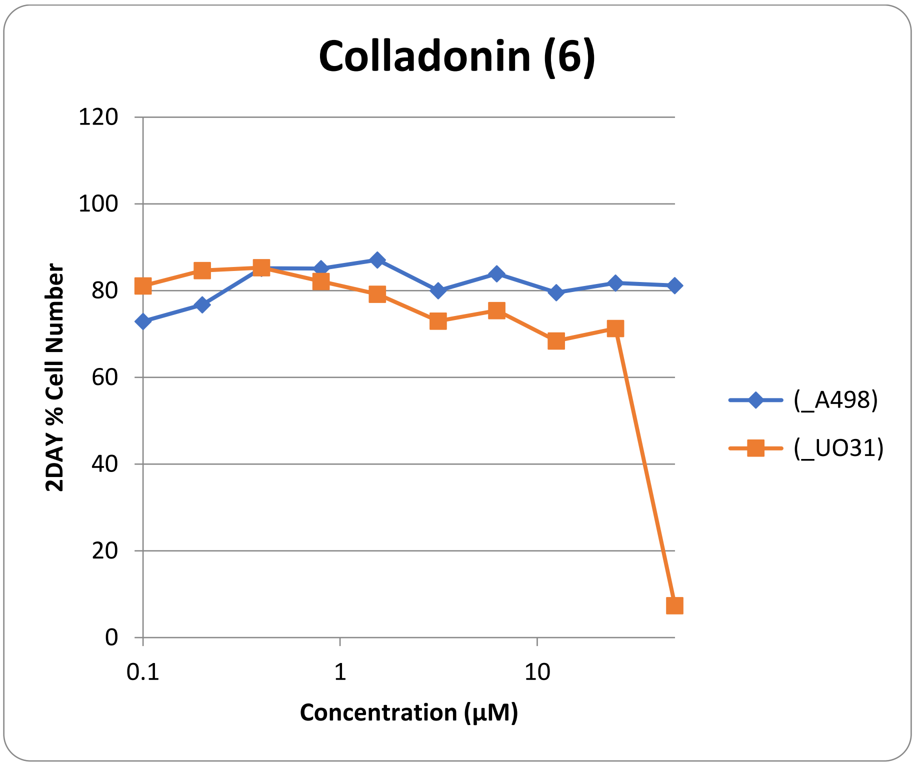

Figure S94: Concentration-dependent cytotoxic effects of colladonin (**6**) on A498 and UO31 cell lines.

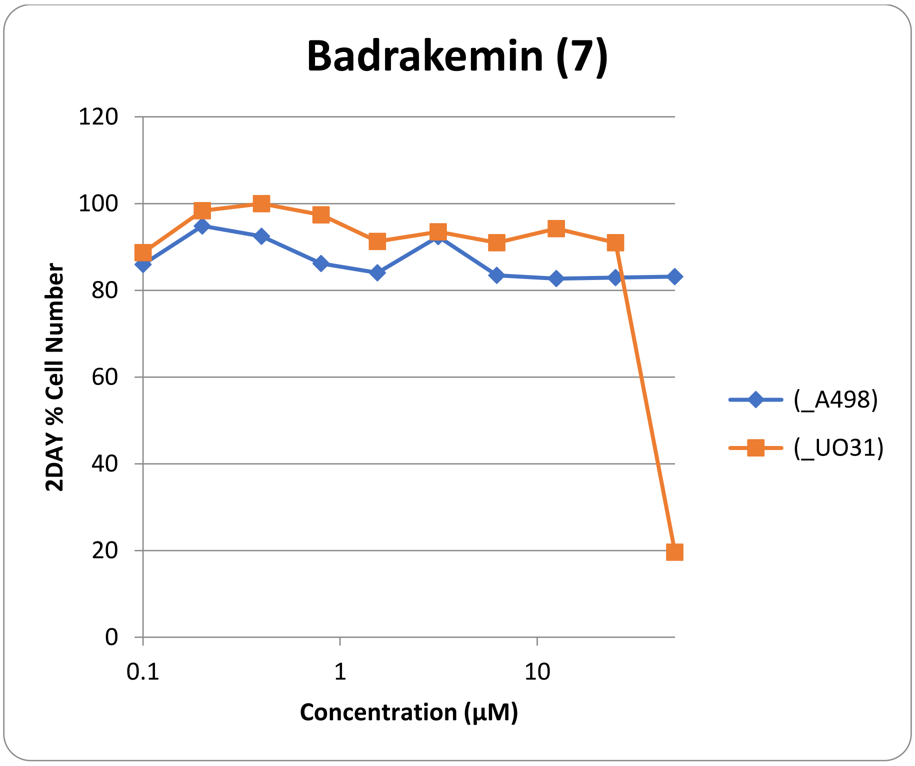

Figure S95: Concentration-dependent cytotoxic effects of badrakemin (**7**) on A498 and UO31 cell lines.

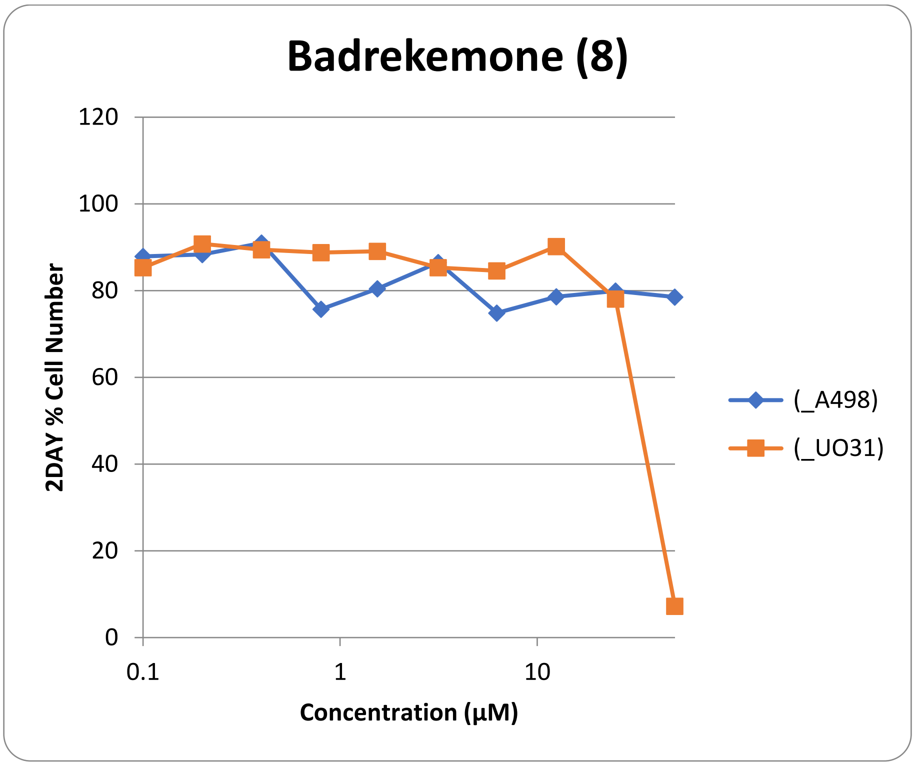

Figure S96: Concentration-dependent cytotoxic effects of badrakemone (**8**) on A498 and UO31 cell lines.

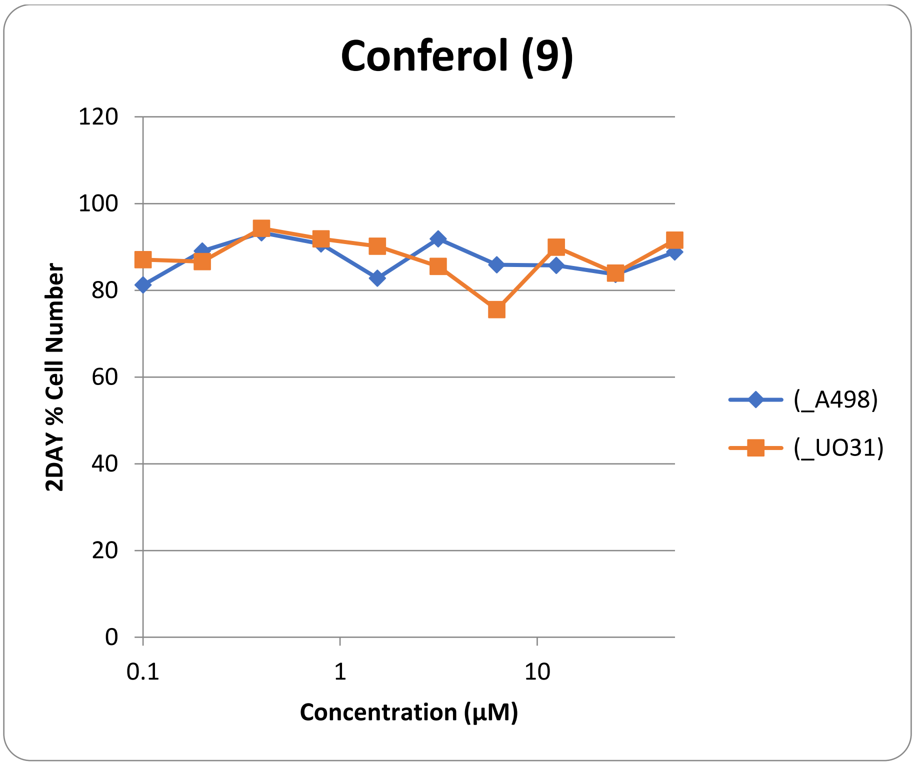

Figure S97: Concentration-dependent cytotoxic effects of conferol (**9**) on A498 and UO31 cell lines.

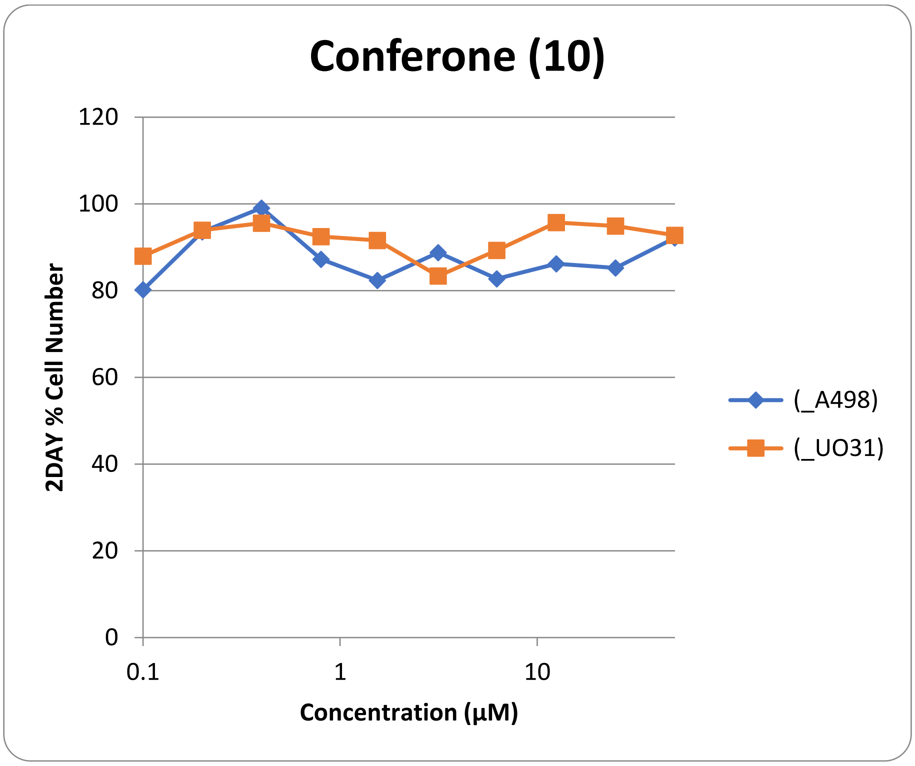

Figure S98: Concentration-dependent cytotoxic effects of conferone (**10**) on A498 and UO31 cell lines.

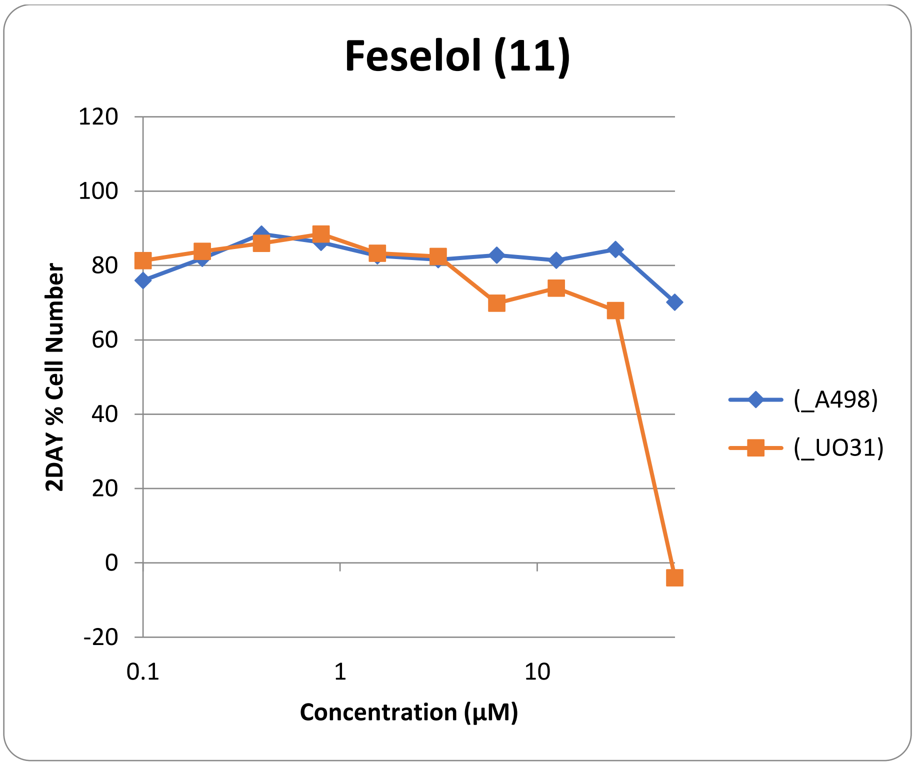

Figure S99: Concentration-dependent cytotoxic effects of feselol (**11**) on A498 and UO31 cell lines.

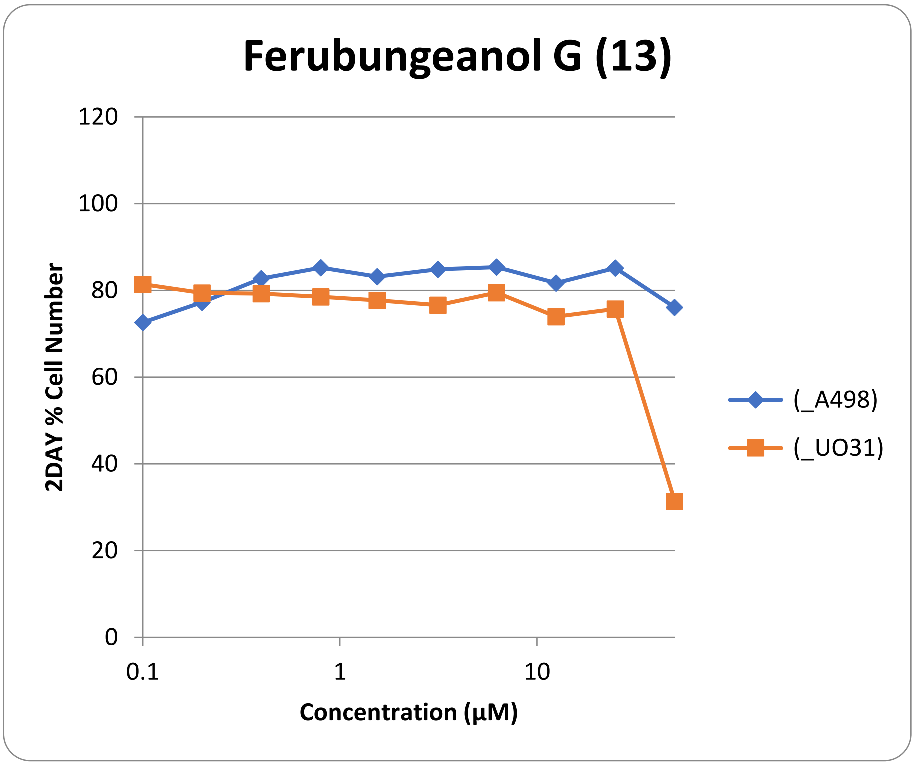

Figure S100: Concentration-dependent cytotoxic effects of ferubungeanol G (**13**) on A498 and UO31 cell lines.

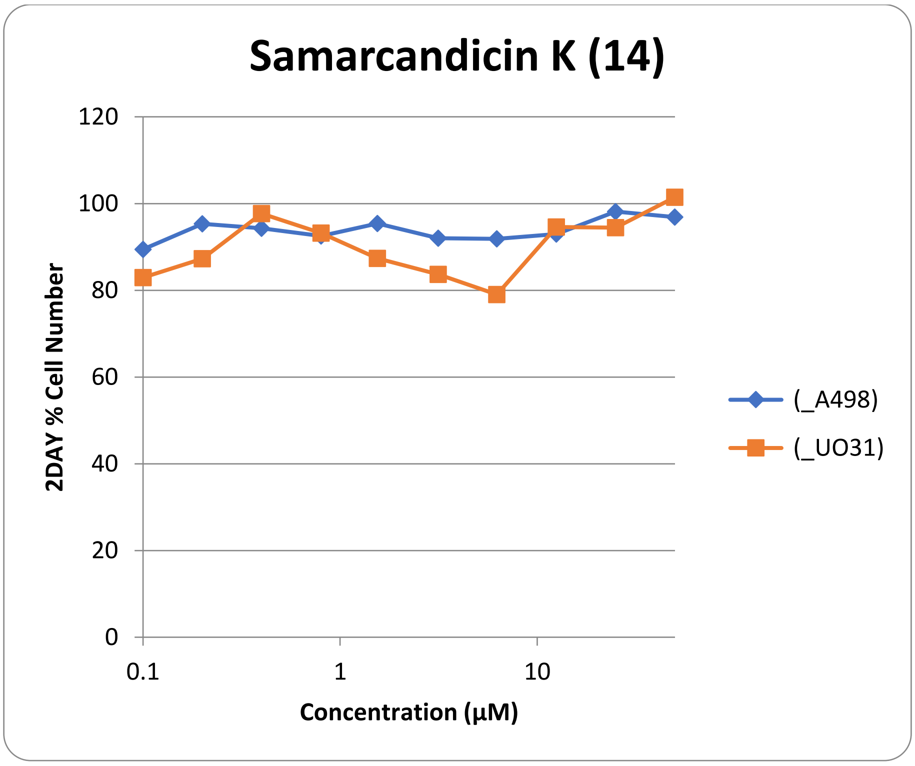

Figure S101: Concentration-dependent cytotoxic effects of samarcandicin K (**14**) on A498 and UO31 cell lines.

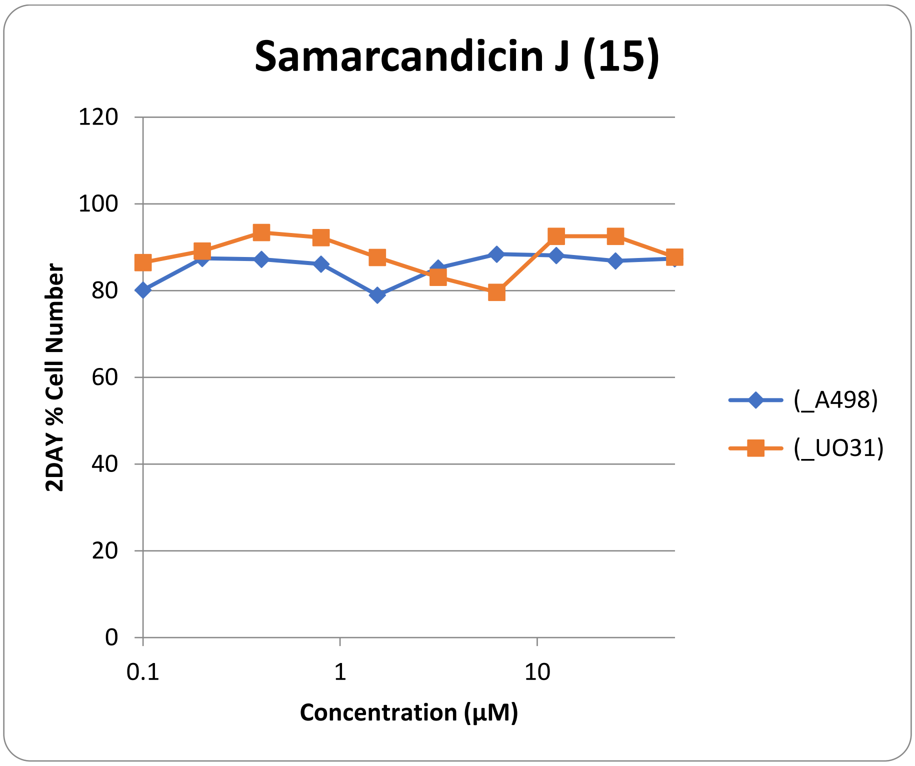

Figure S102: Concentration-dependent cytotoxic effects of samarcandicin J (**15**) on A498 and UO31 cell lines.

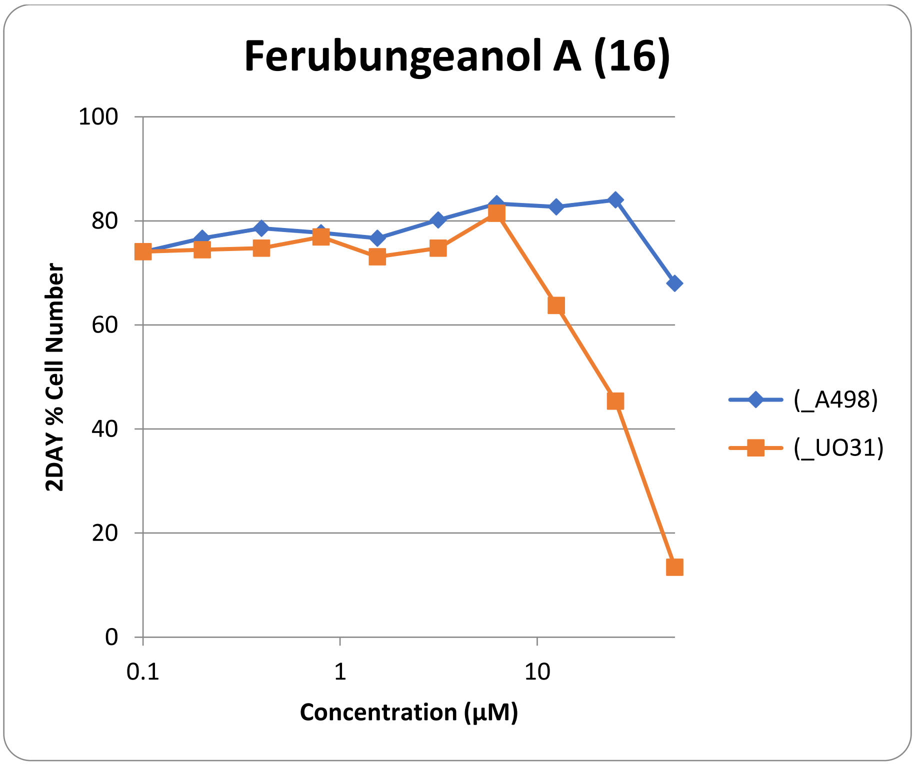

Figure S103: Concentration-dependent cytotoxic effects of ferubungeanol A (**16**) on A498 and UO31 cell lines.

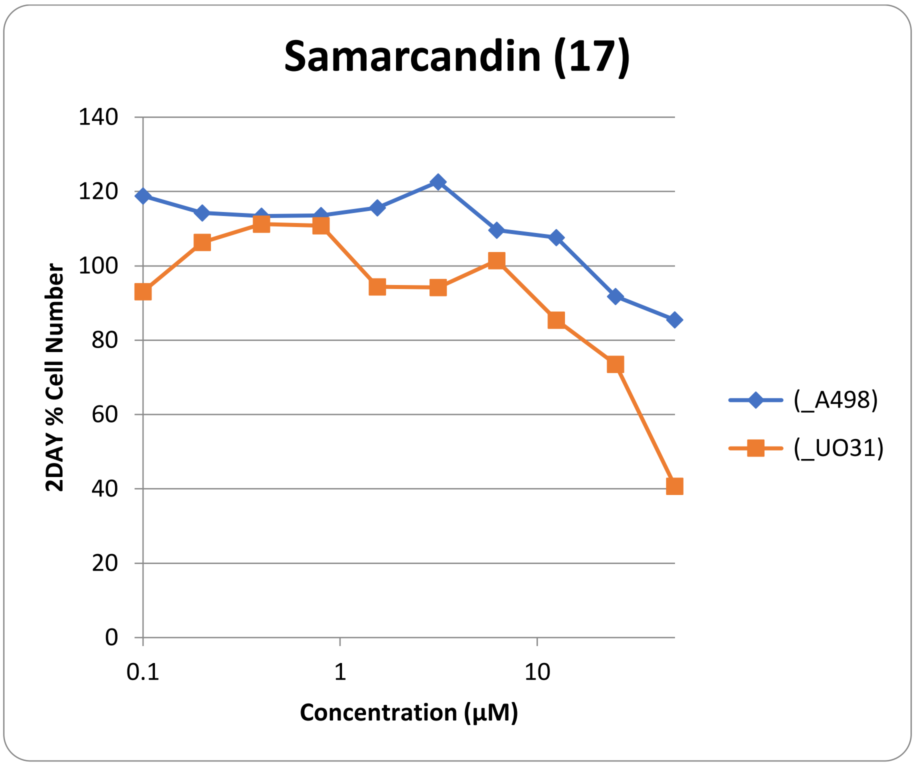

Figure S104: Concentration-dependent cytotoxic effects of samarcandin (**17**) on A498 and UO31 cell lines.

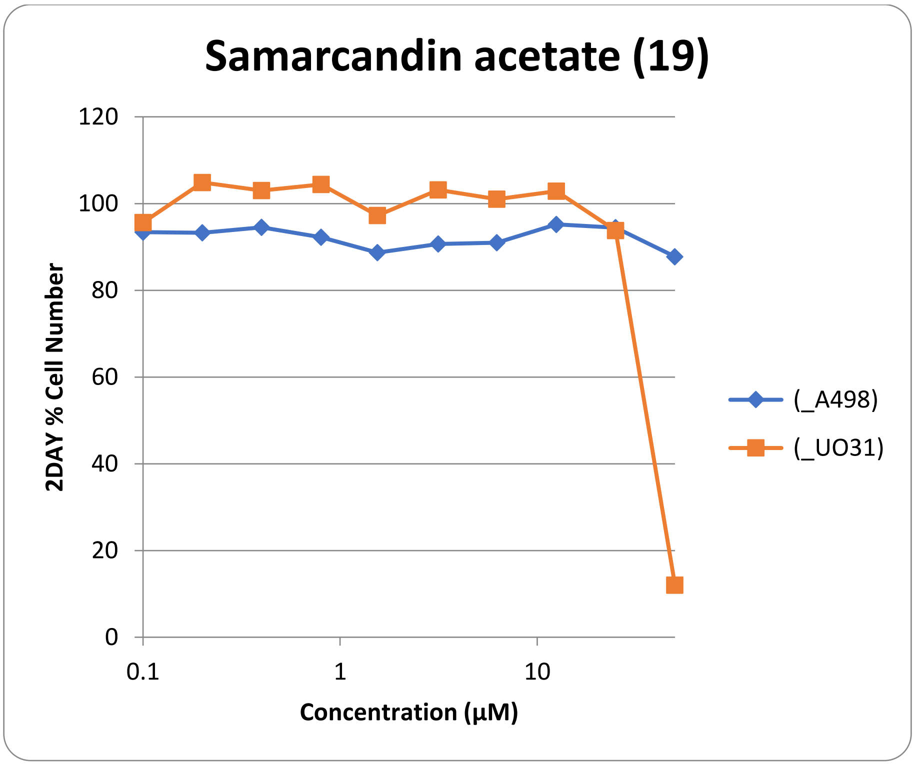

Figure S105: Concentration-dependent cytotoxic effects of samarcandin acetate (**19**) on A498 and UO31 cell lines.

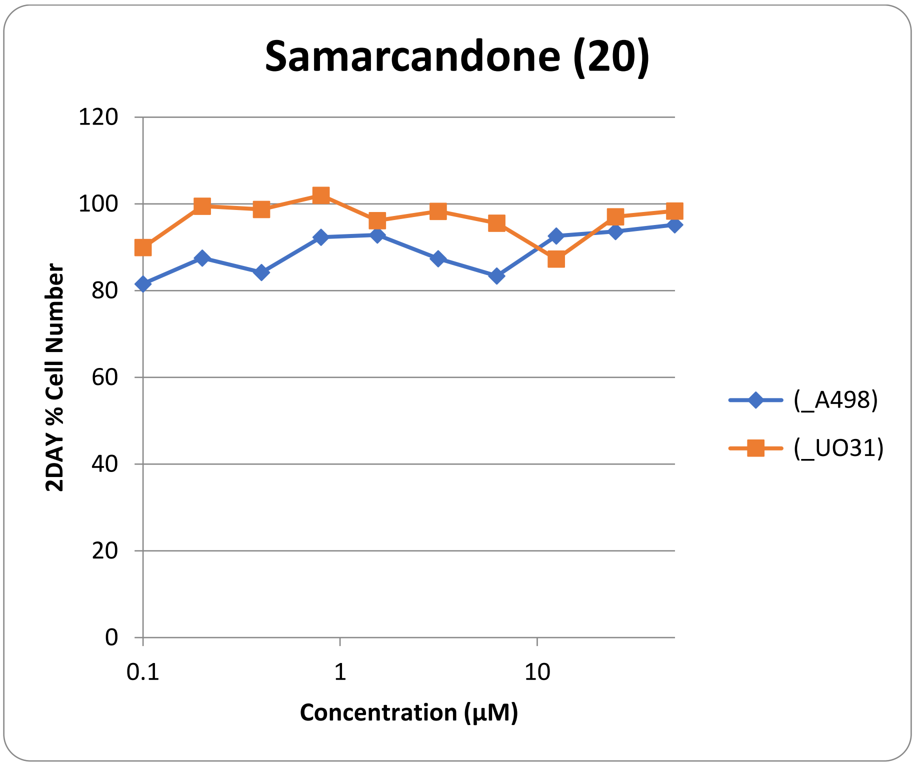

Figure S106: Concentration-dependent cytotoxic effects of samarcandone (**20**) on A498 and UO31 cell lines.

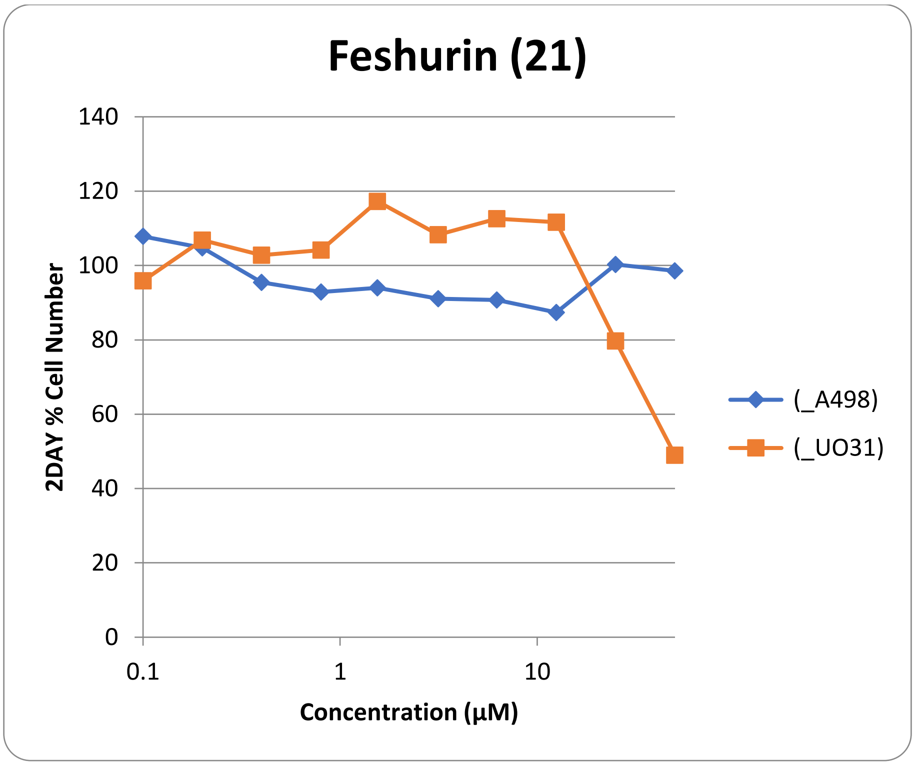

Figure S107: Concentration-dependent cytotoxic effects of feshurin (**21**) on A498 and UO31 cell lines.

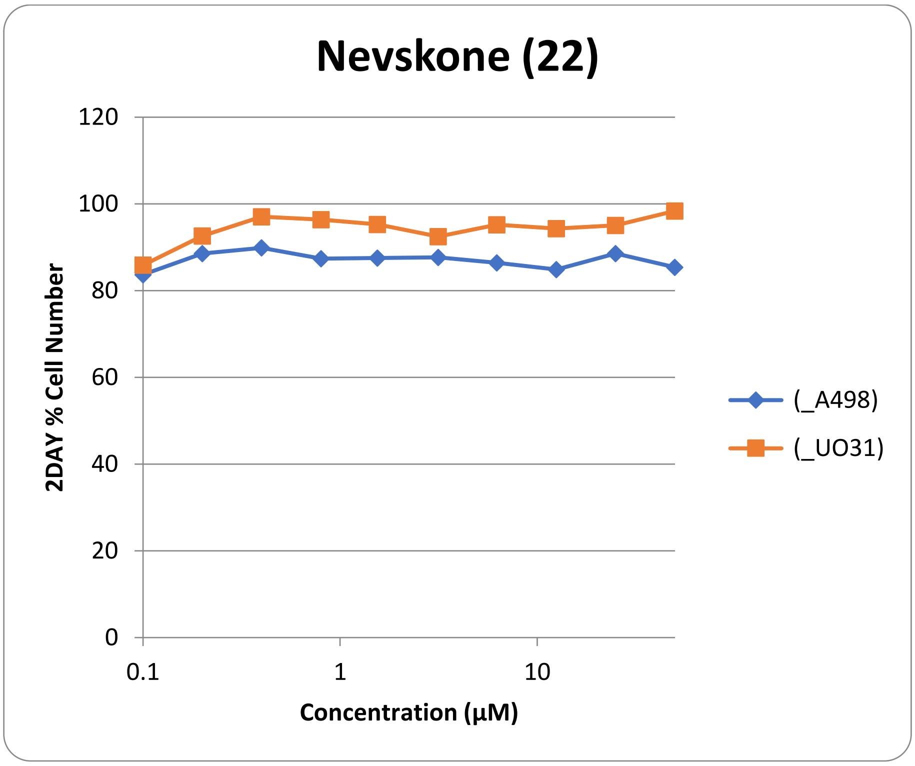

Figure S108: Concentration-dependent cytotoxic effects of nevskone (**22**) on A498 and UO31 cell lines.

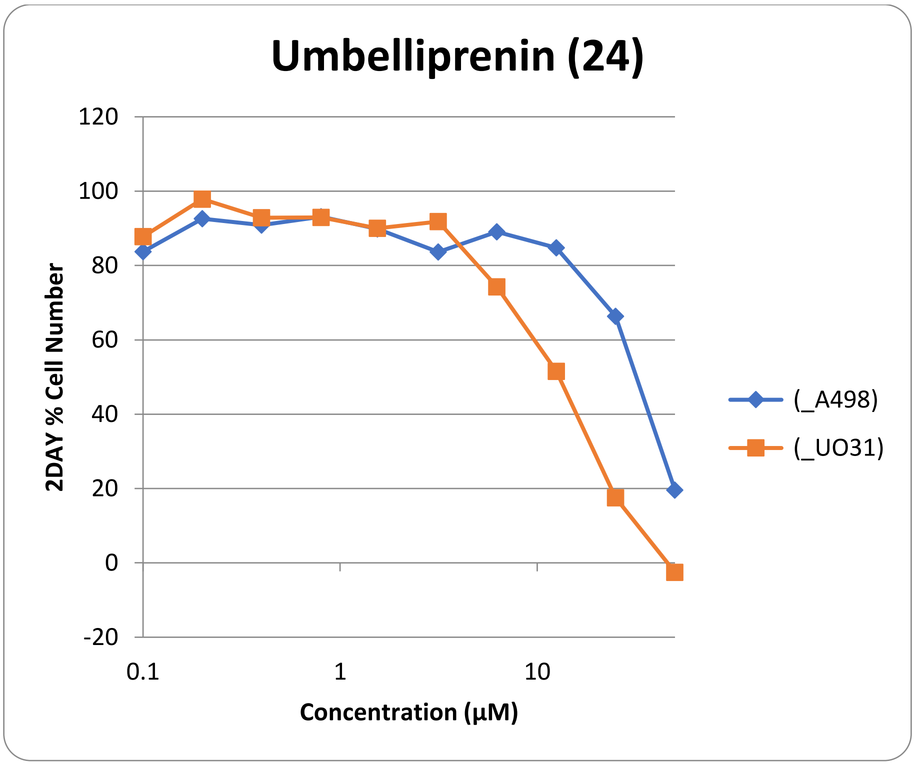

Figure S109: Concentration-dependent cytotoxic effects of umbelliprenin (**24**) on A498 and UO31 cell lines.

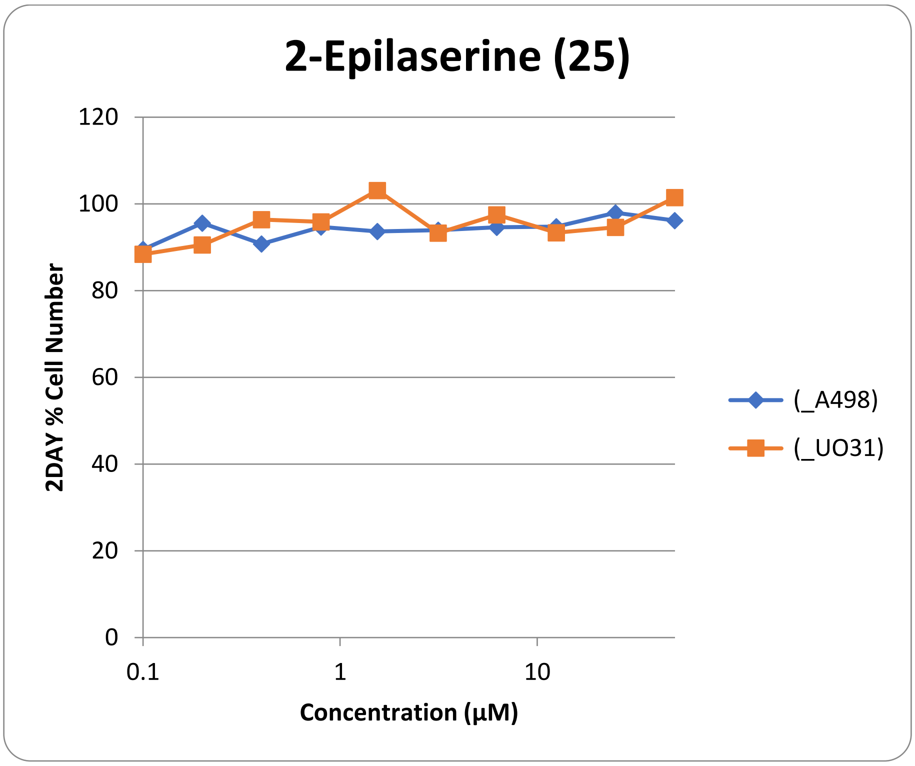

Figure S110: Concentration-dependent cytotoxic effects of 2-epilaserine (**25**) on A498 and UO31 cell lines.

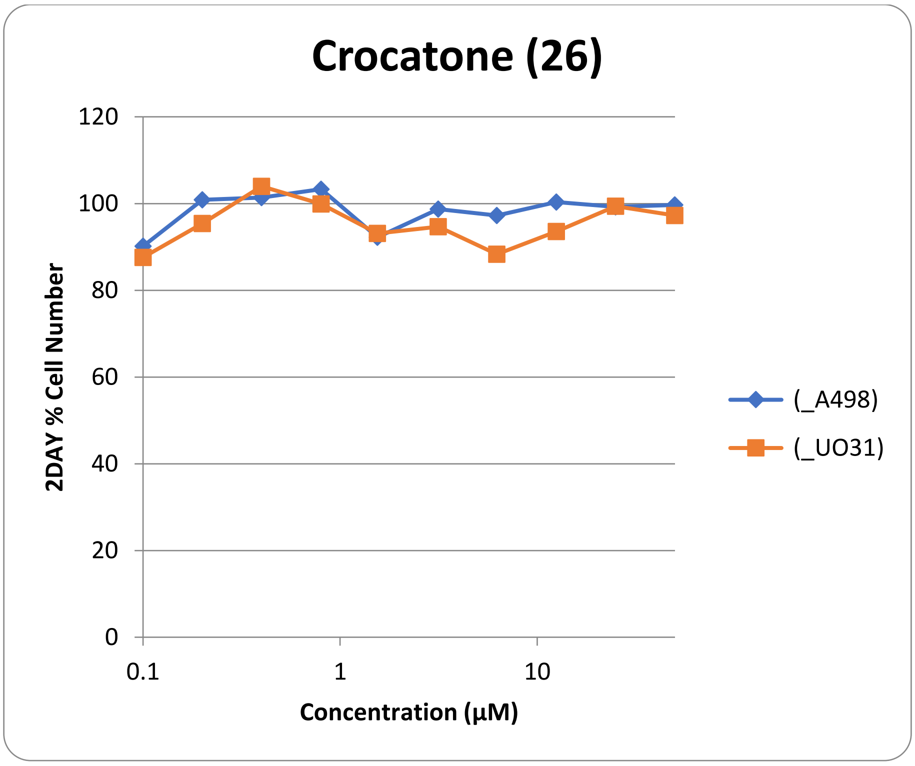

Figure S111: Concentration-dependent cytotoxic effects of crocatone (**26**) on A498 and UO31 cell lines.

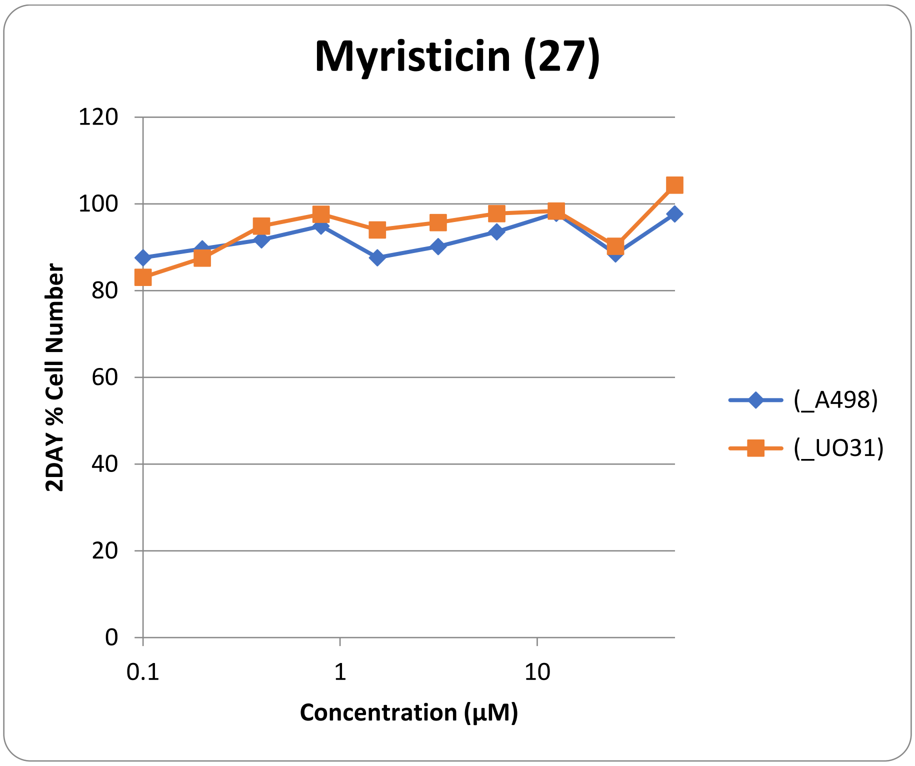

Figure S112: Concentration-dependent cytotoxic effects of myristicin (**27**) on A498 and UO31 cell lines.

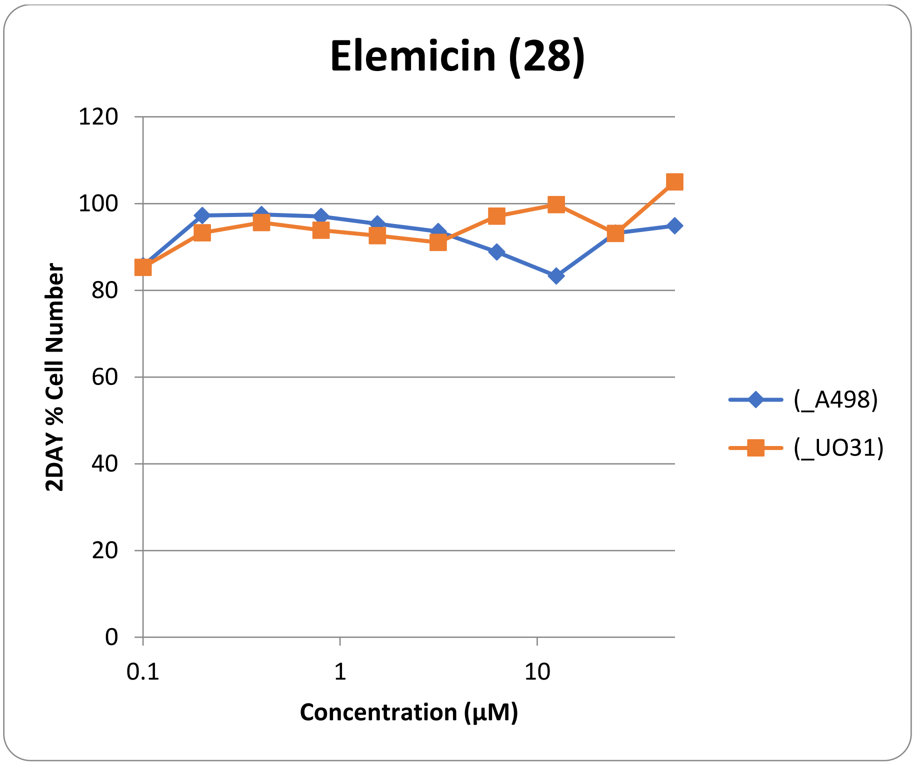

Figure S113: Concentration-dependent cytotoxic effects of elemicin (**28**) on A498 and UO31 cell lines.

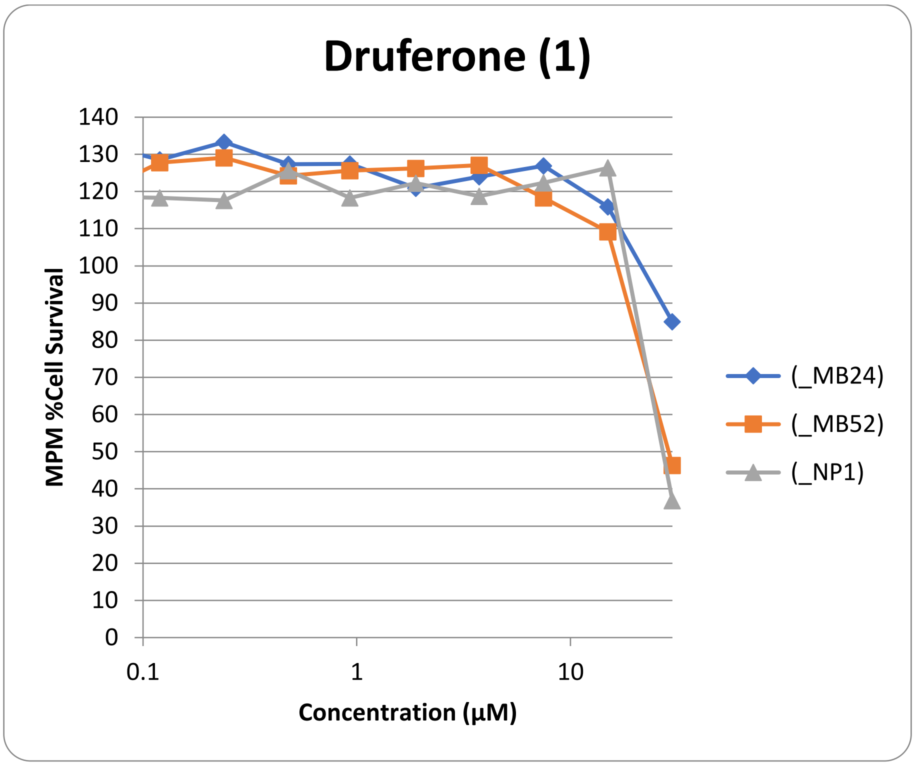

Figure S114: Concentration-dependent cytotoxic effects of druferone (**1**) on MB24, MB52, and NP1 cell lines.

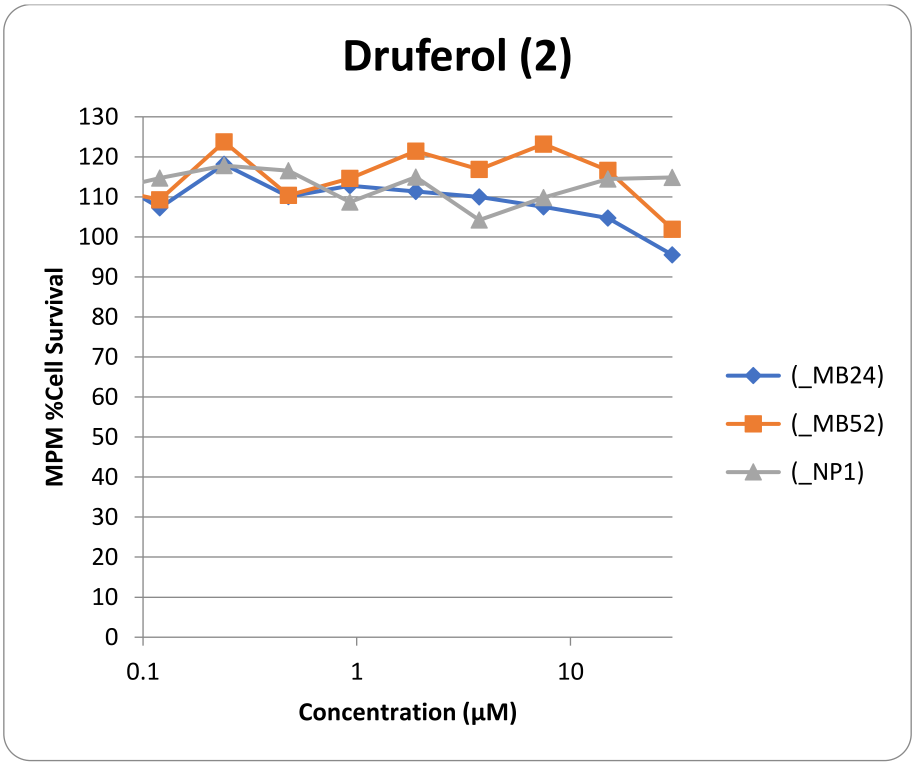

Figure S115: Concentration-dependent cytotoxic effects of druferol (**2**) on MB24, MB52, and NP1 cell lines.

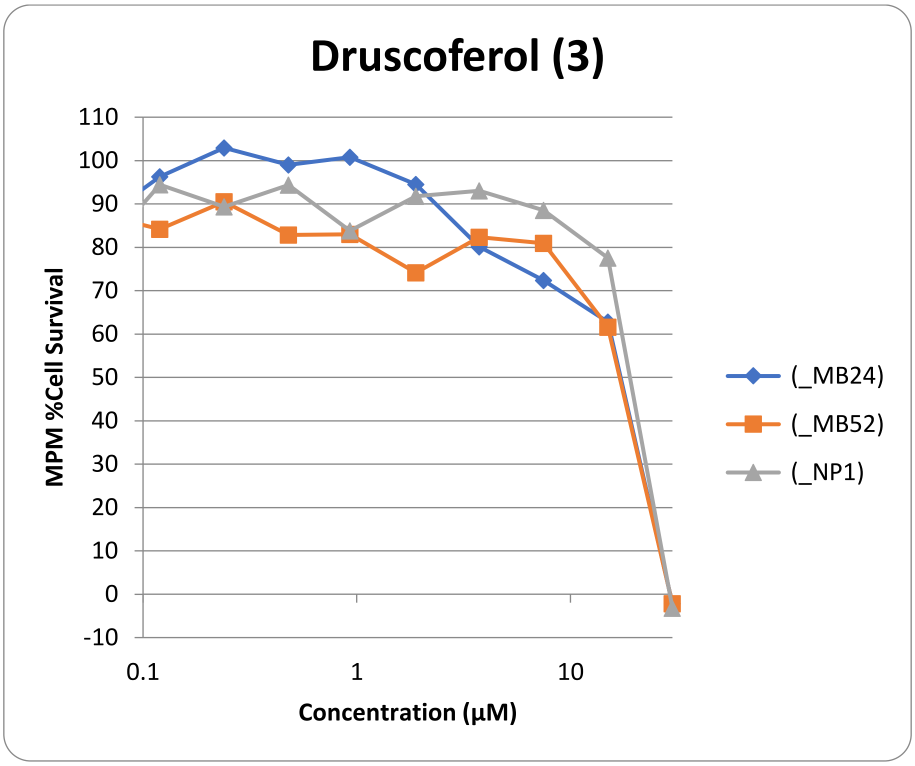

Figure S116: Concentration-dependent cytotoxic effects of druscoferol (**3**) on MB24, MB52, and NP1 cell lines.

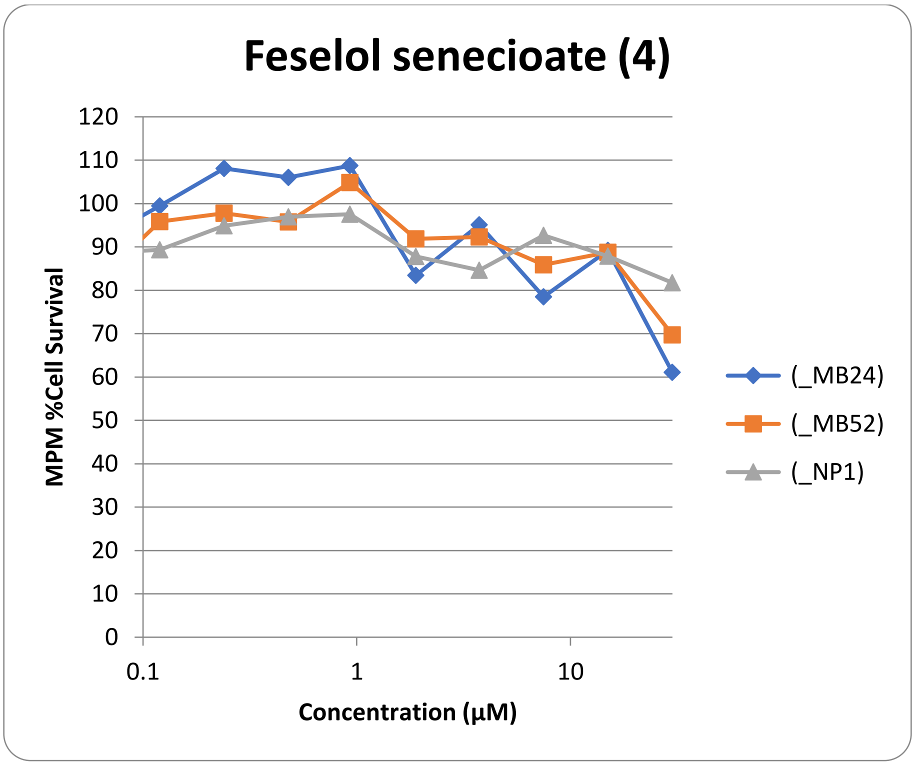

Figure S117: Concentration-dependent cytotoxic effects of feselol senecioate (**4**) on MB24, MB52, and NP1 cell lines.

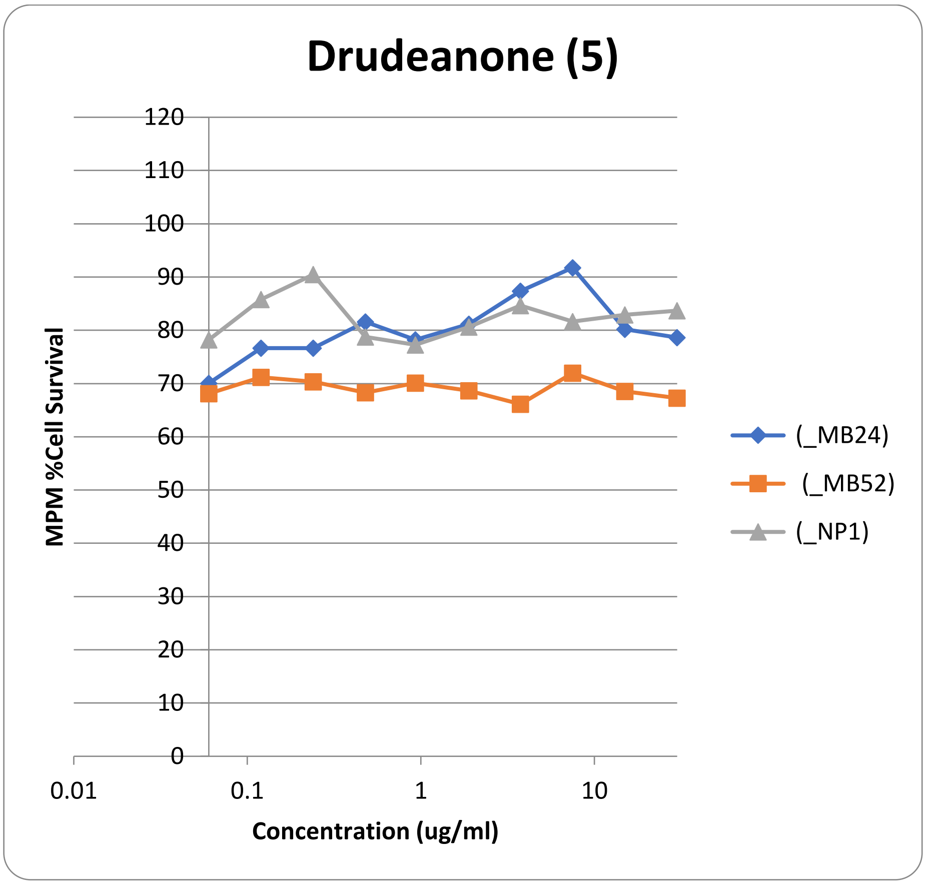

Figure S118: Concentration-dependent cytotoxic effects of drudeanone (**5**) on MB24, MB52, and NP1 cell lines.

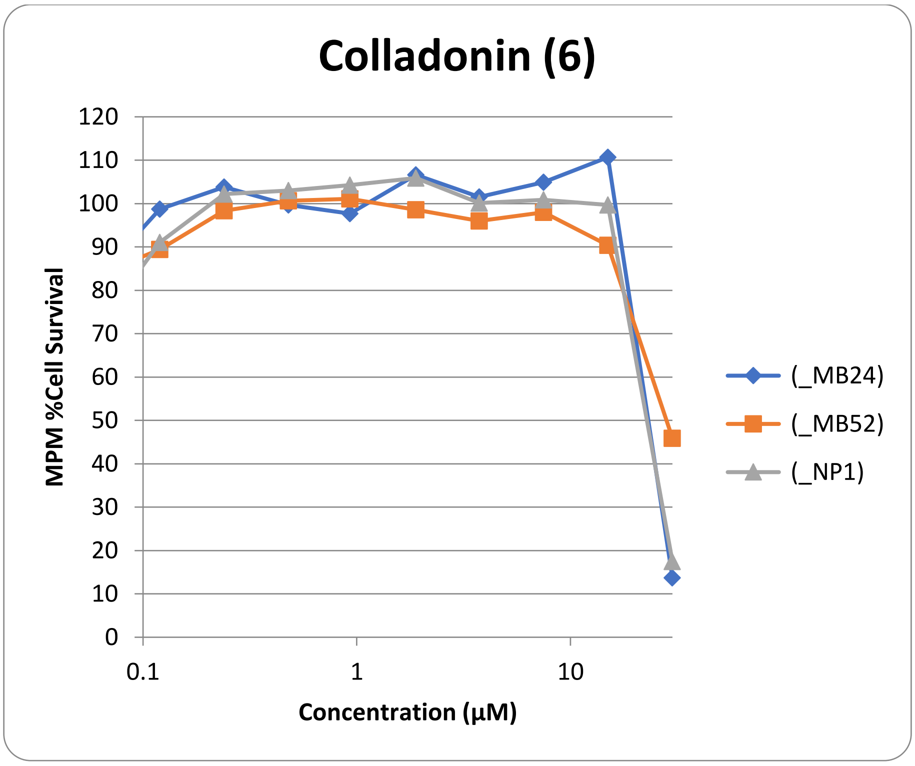

Figure S119: Concentration-dependent cytotoxic effects of colladonin (**6**) on MB24, MB52, and NP1 cell lines.

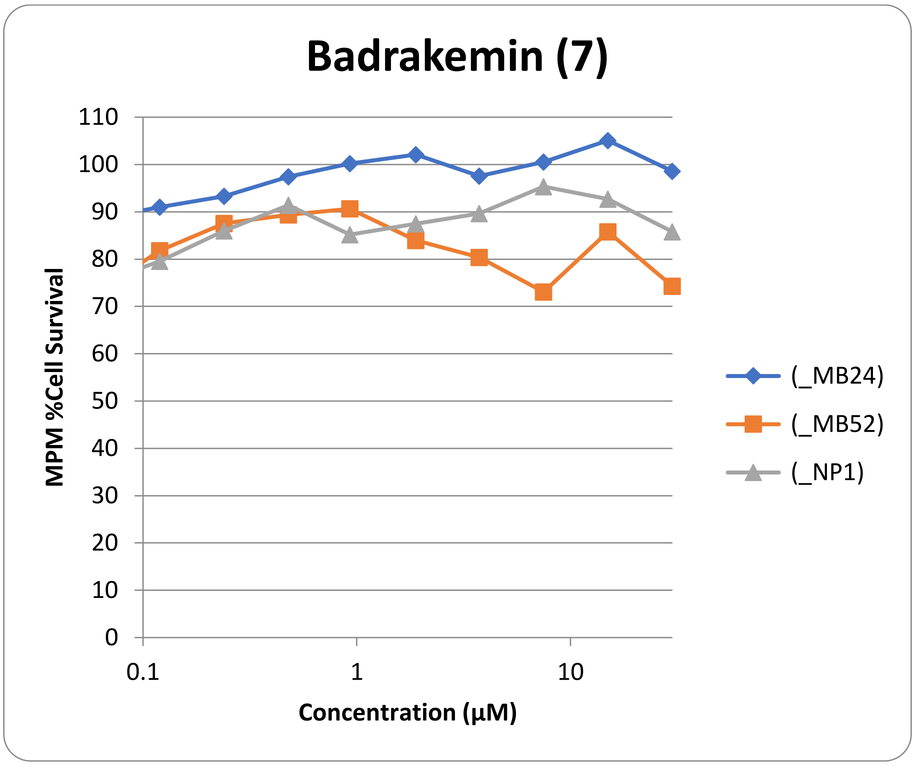

Figure S120: Concentration-dependent cytotoxic effects of badrakemin (**7**) on MB24, MB52, and NP1 cell lines.

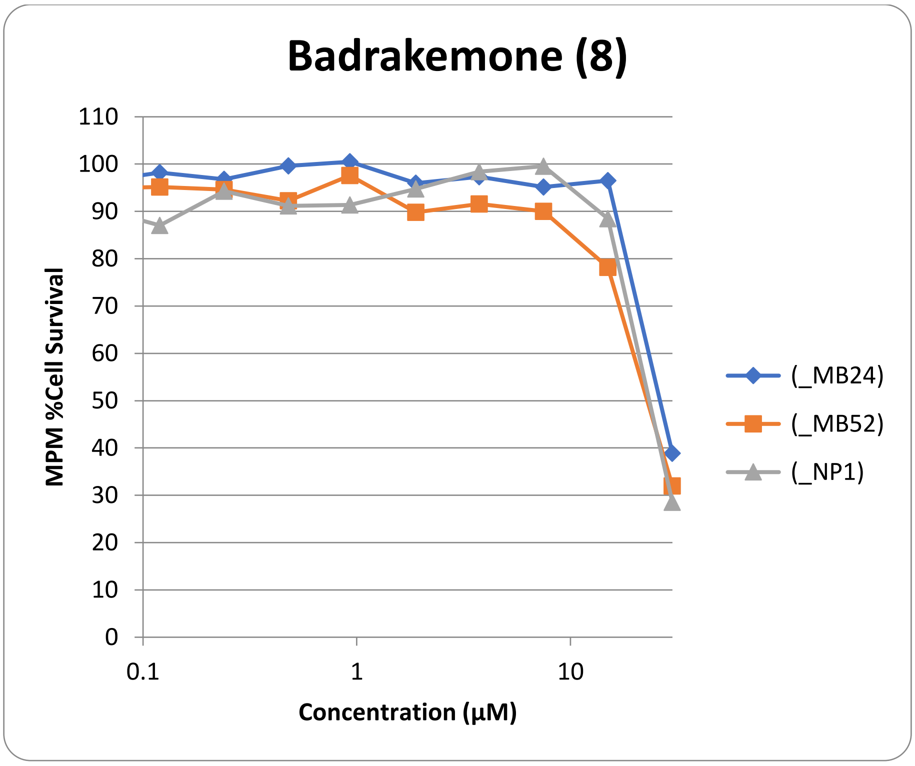

Figure S121: Concentration-dependent cytotoxic effects of badrakemon (**8**) on MB24, MB52, and NP1 cell lines.

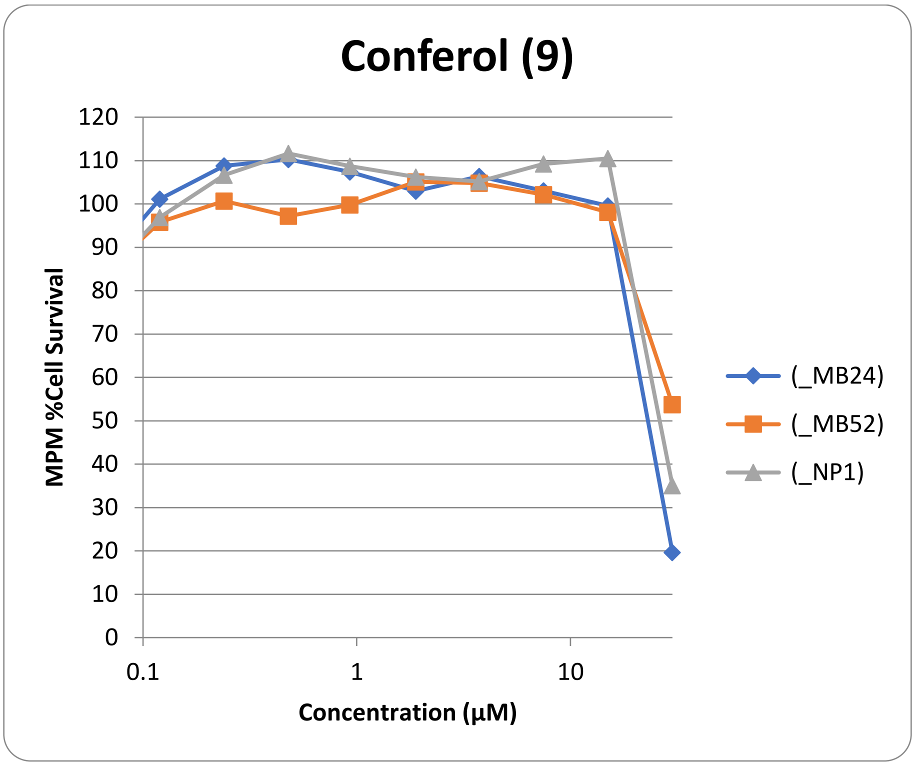

Figure S122: Concentration-dependent cytotoxic effects of conferol (**9**) on MB24, MB52, and NP1 cell lines.

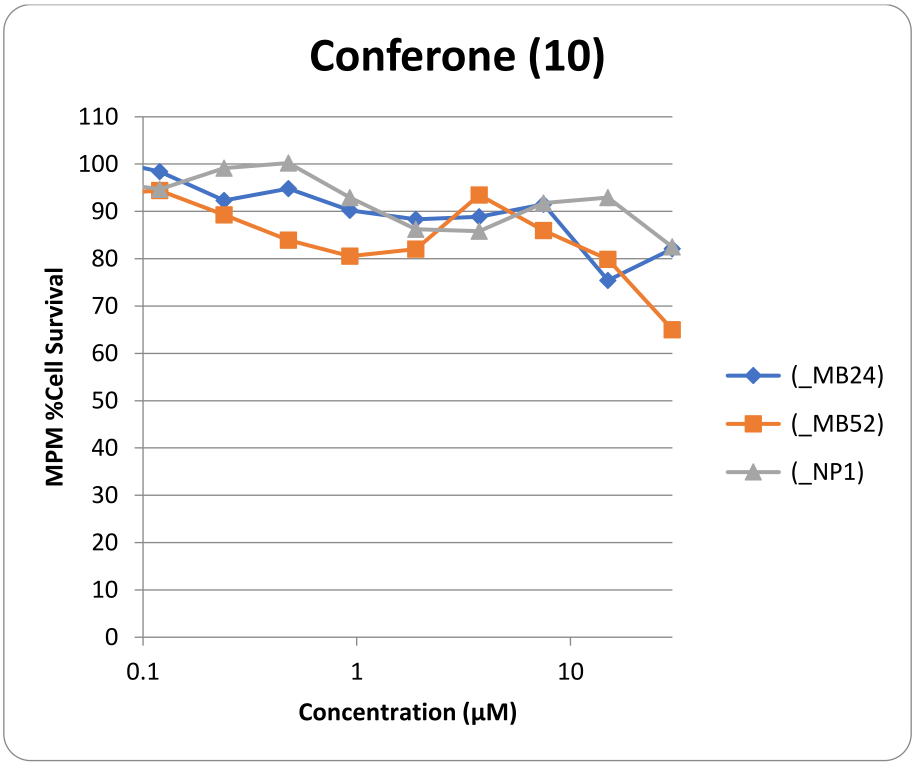

Figure S123: Concentration-dependent cytotoxic effects of conferone (**10**) on MB24, MB52, and NP1 cell lines.

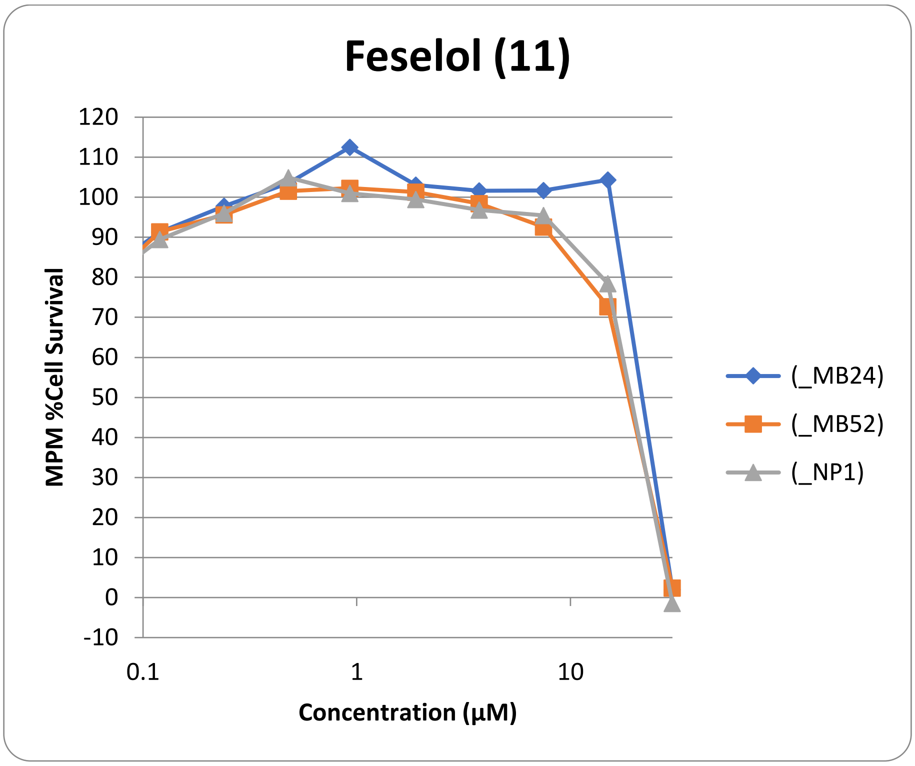

Figure S124: Concentration-dependent cytotoxic effects of feselol (**11**) on MB24, MB52, and NP1 cell lines.

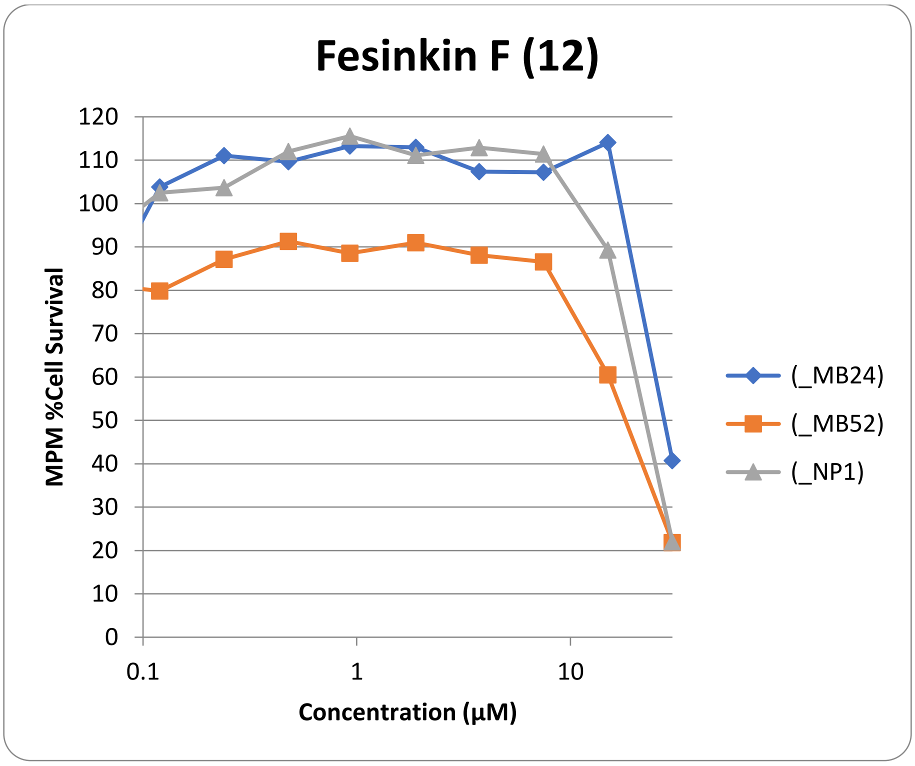

Figure S125: Concentration-dependent cytotoxic effects of fesinkin F (**12**) on MB24, MB52, and NP1 cell lines.

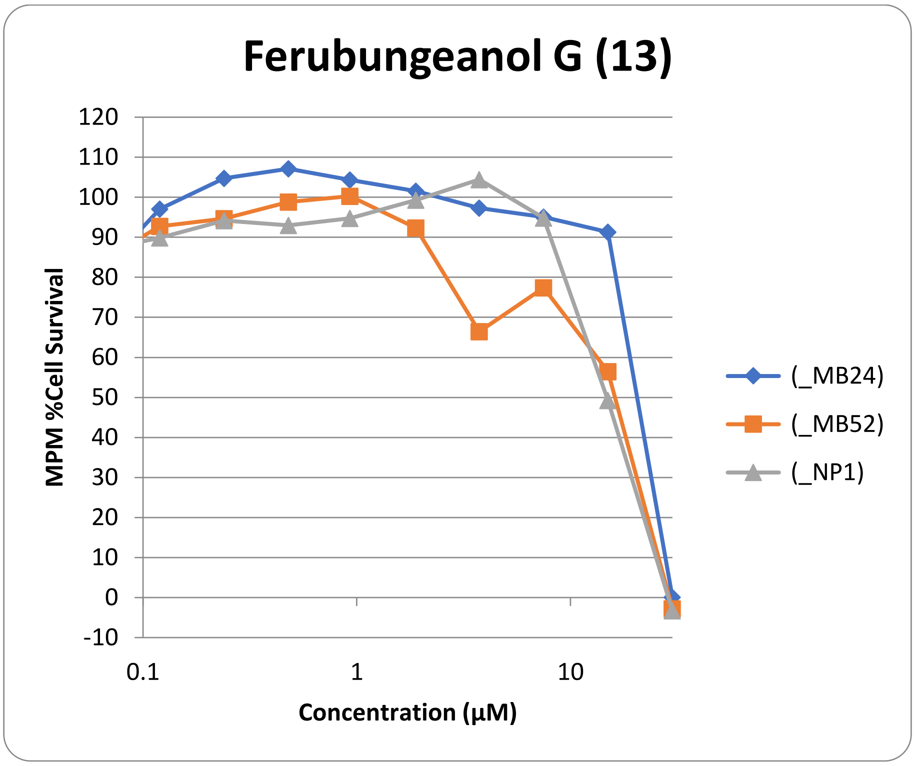

Figure S126: Concentration-dependent cytotoxic effects of ferubungeanol G (**13**) on MB24, MB52, and NP1 cell lines.

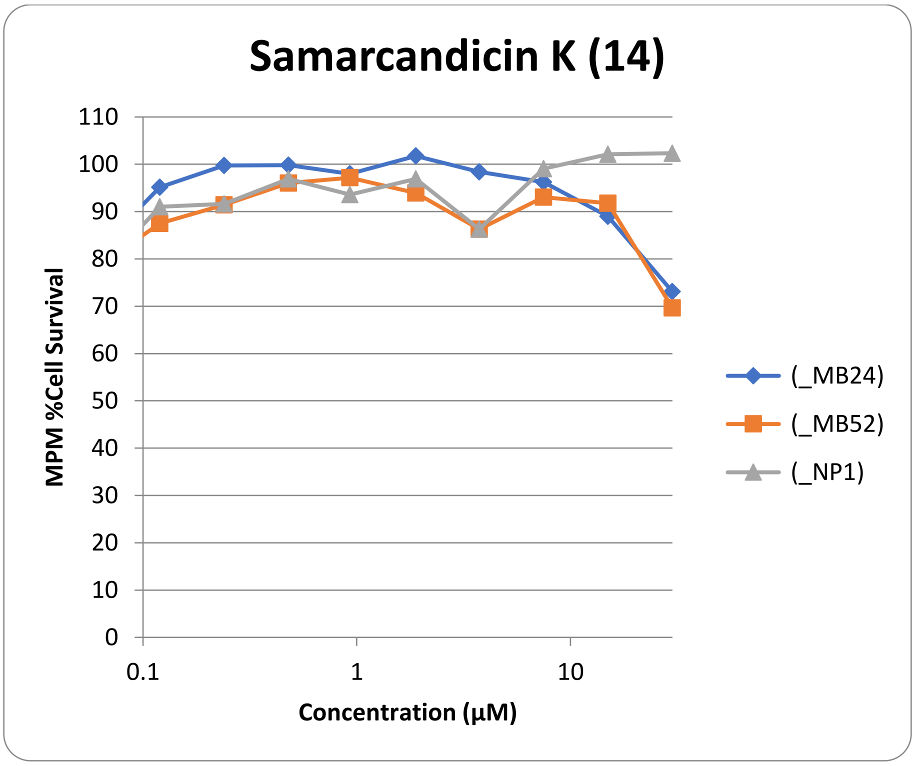

Figure S127: Concentration-dependent cytotoxic effects of samarcandicin K (**14**) on MB24, MB52, and NP1 cell lines.

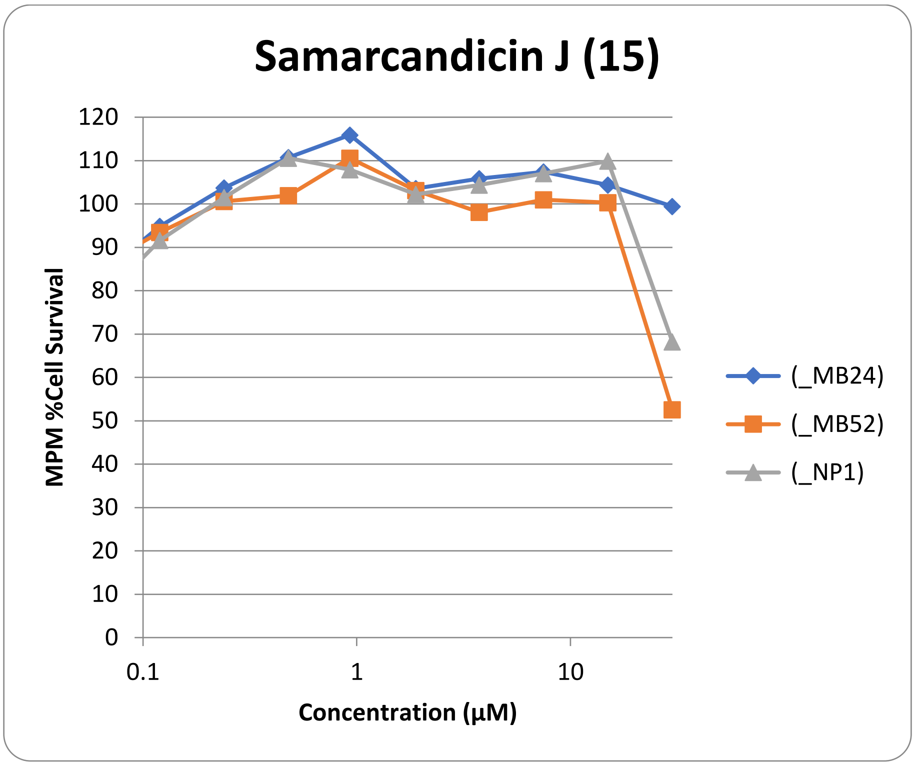

Figure S128: Concentration-dependent cytotoxic effects of samarcandicin J (**15**) on MB24, MB52, and NP1 cell lines.

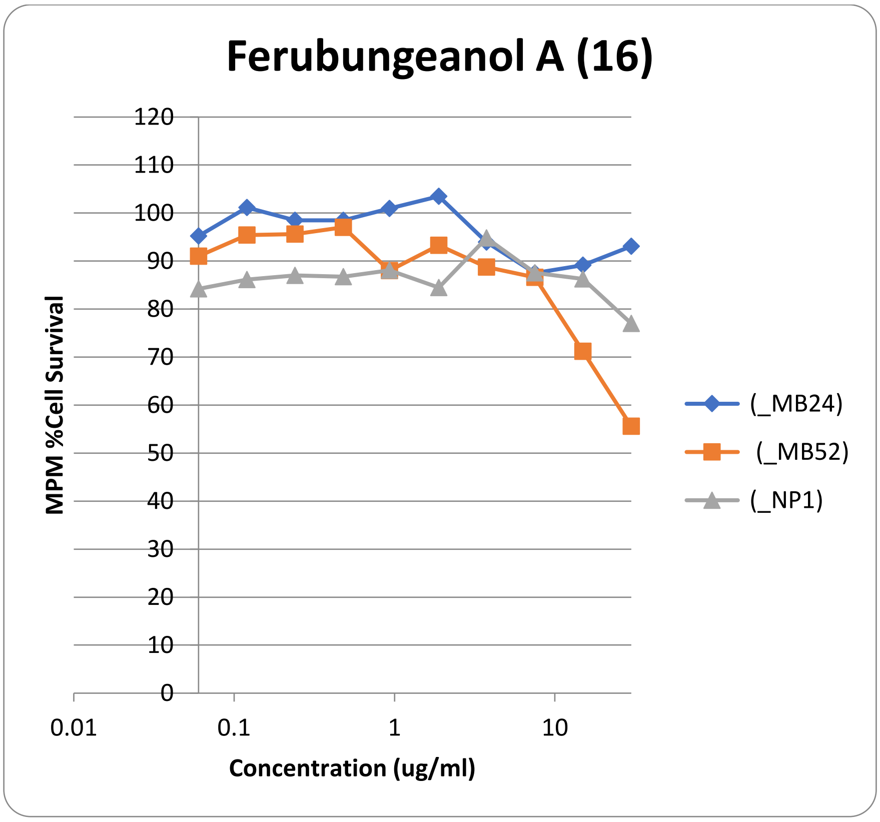

Figure S129: Concentration-dependent cytotoxic effects of ferubungeanol A (**16**) on MB24, MB52, and NP1 cell lines.

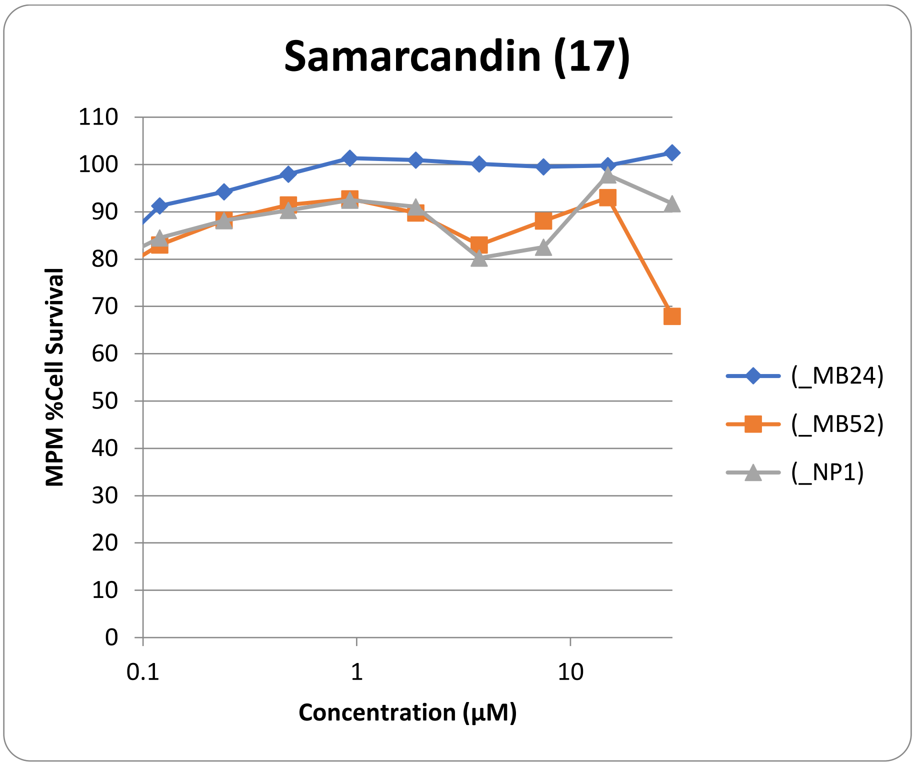

Figure S130: Concentration-dependent cytotoxic effects of samarcandin (**17**) on MB24, MB52, and NP1 cell lines.

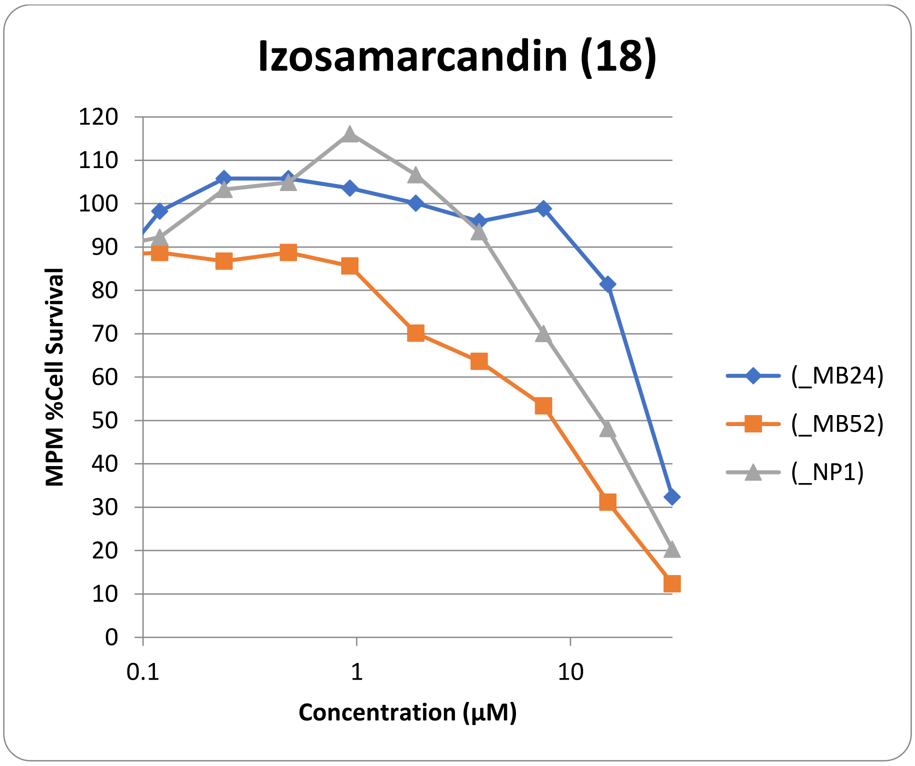

Figure S131: Concentration-dependent cytotoxic effects of isosamarcandin (**18**) on MB24, MB52, and NP1 cell lines.

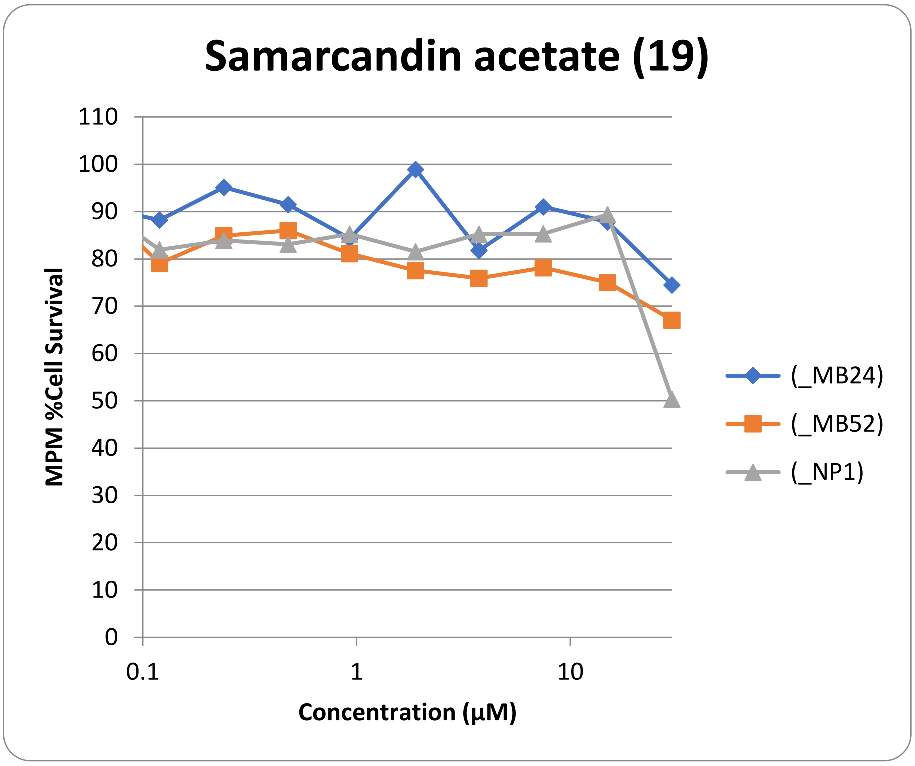

Figure S132: Concentration-dependent cytotoxic effects of samarcandin acetate (**19**) on MB24, MB52, and NP1 cell lines.

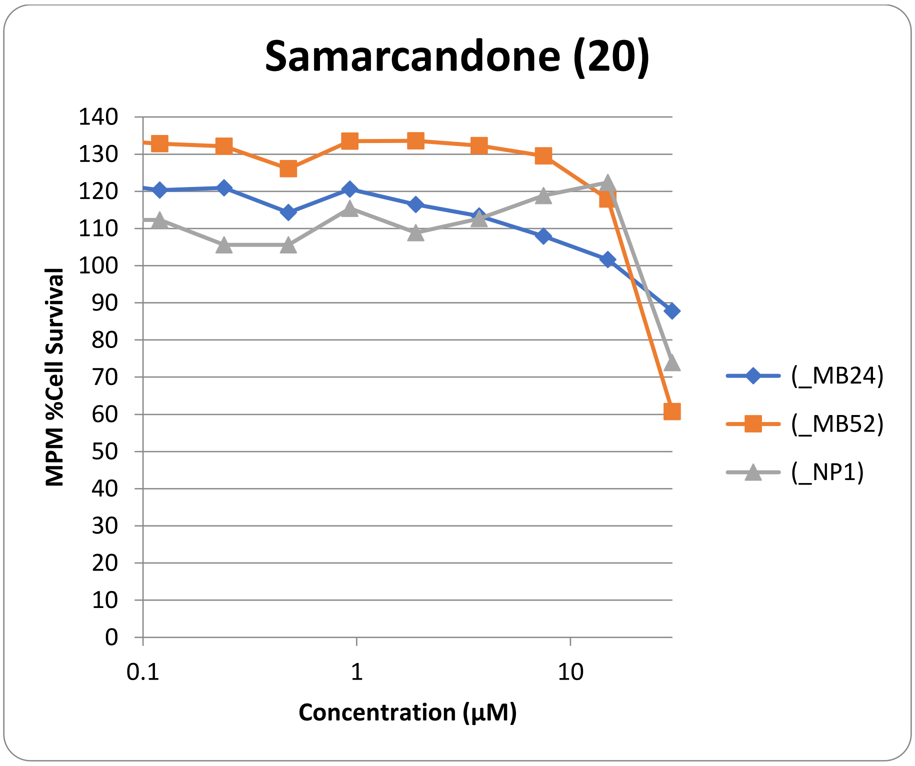

Figure S133: Concentration-dependent cytotoxic effects of samarcandone (**20**) on MB24, MB52, and NP1 cell lines.

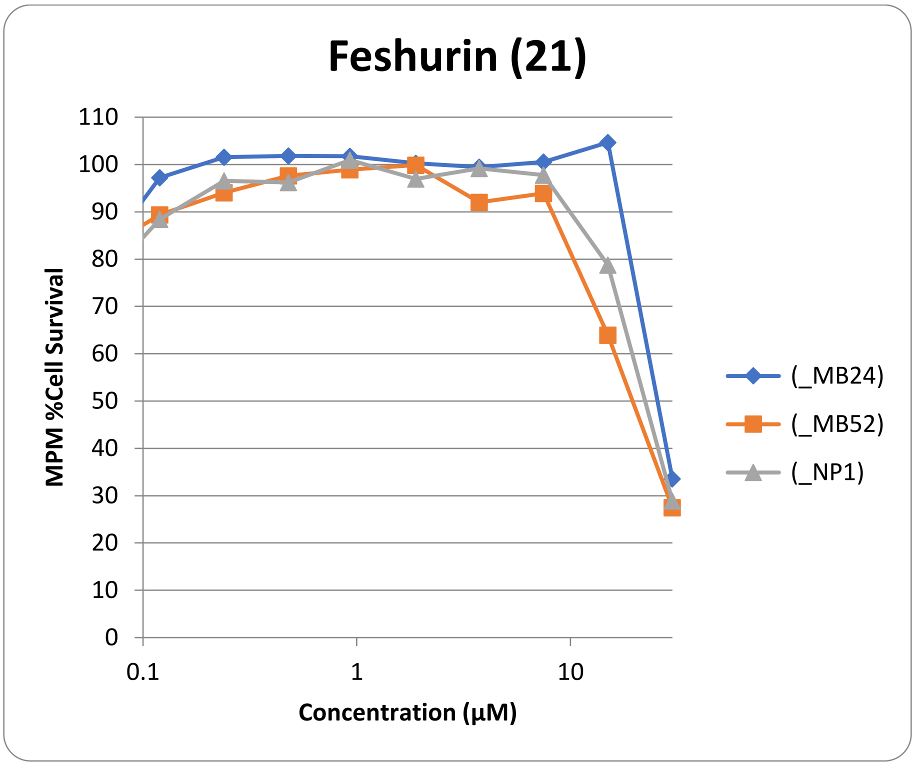

Figure S134: Concentration-dependent cytotoxic effects of feshurin (**21**) on MB24, MB52, and NP1 cell lines.

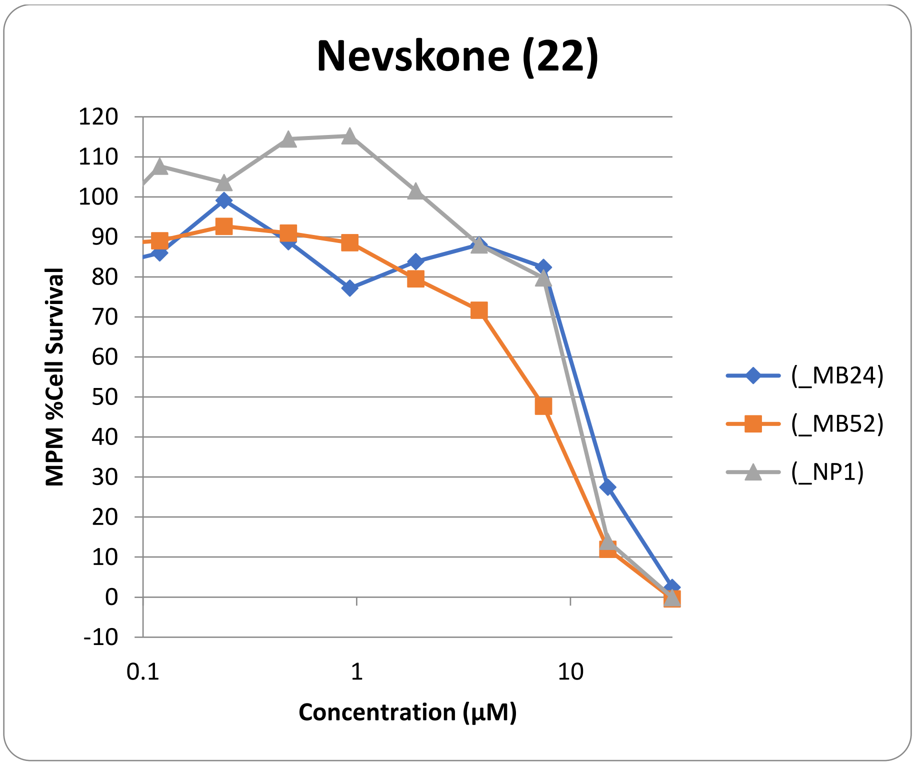

Figure S135: Concentration-dependent cytotoxic effects of feshurone (nevskone) (**22**) on MB24, MB52, and NP1 cell lines.

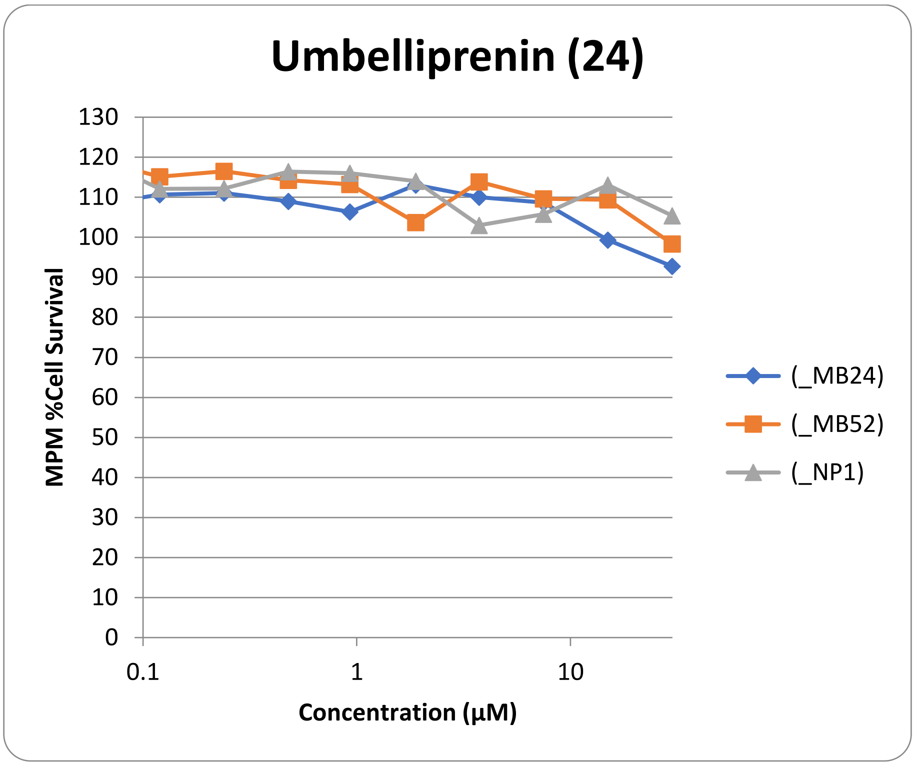

Figure S136: Concentration-dependent cytotoxic effects of umbelliprenin (**24**) on MB24, MB52, and NP1 cell lines.

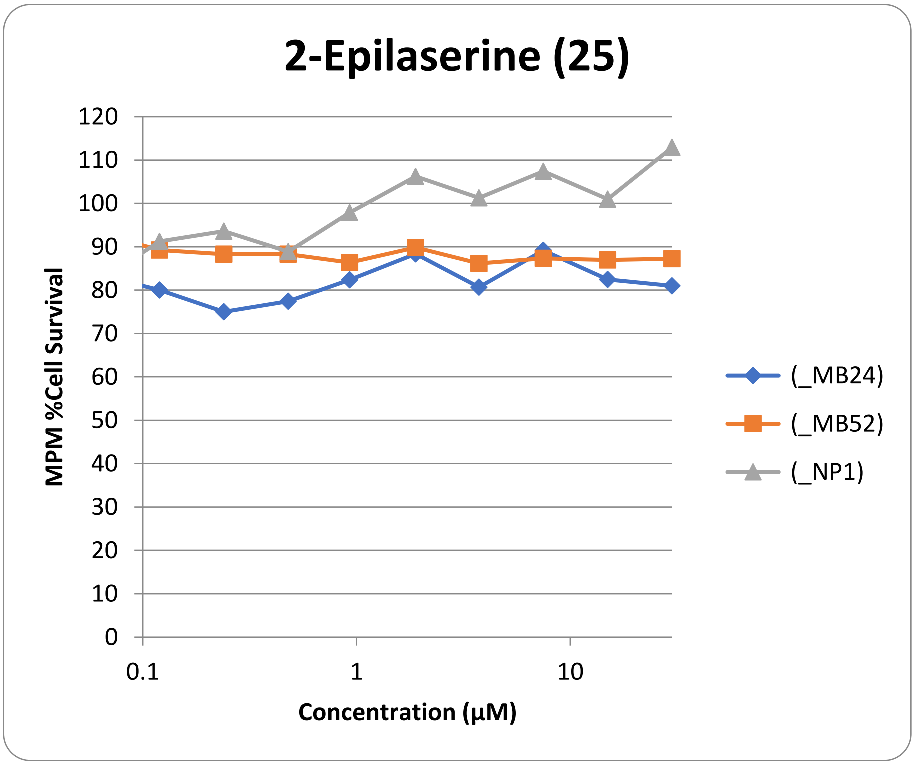

Figure S137: Concentration-dependent cytotoxic effects of 2-epilaserine (**25**) on MB24, MB52, and NP1 cell lines.

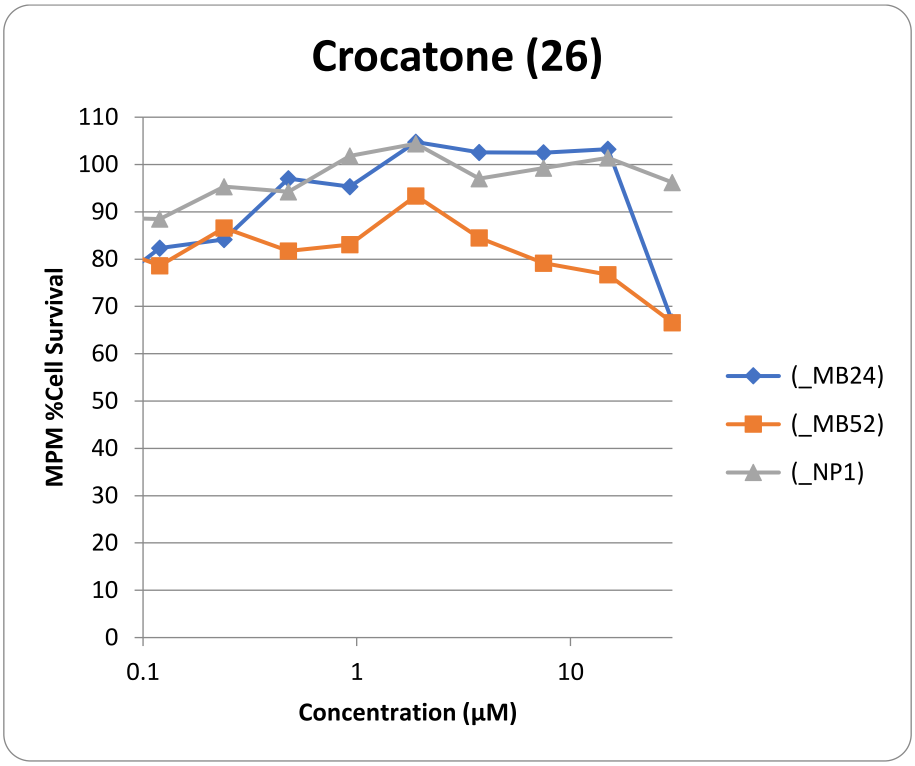

Figure S138: Concentration-dependent cytotoxic effects of crocatone (**26**) on MB24, MB52, and NP1 cell lines.

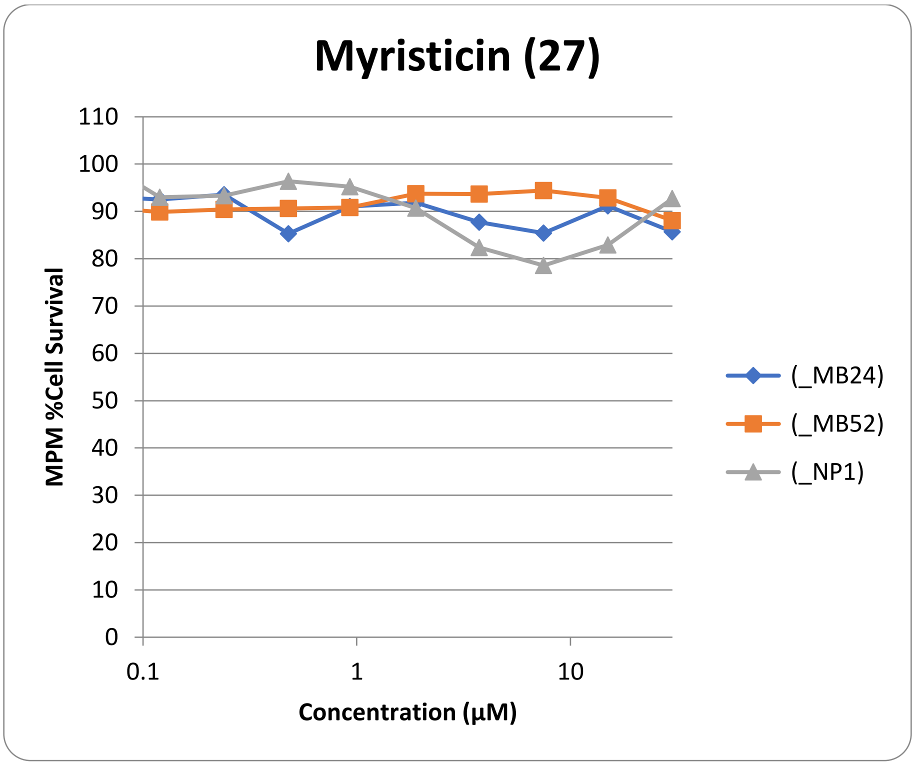

Figure S139: Concentration-dependent cytotoxic effects of myristicin (**27**) on MB24, MB52, and NP1 cell lines.

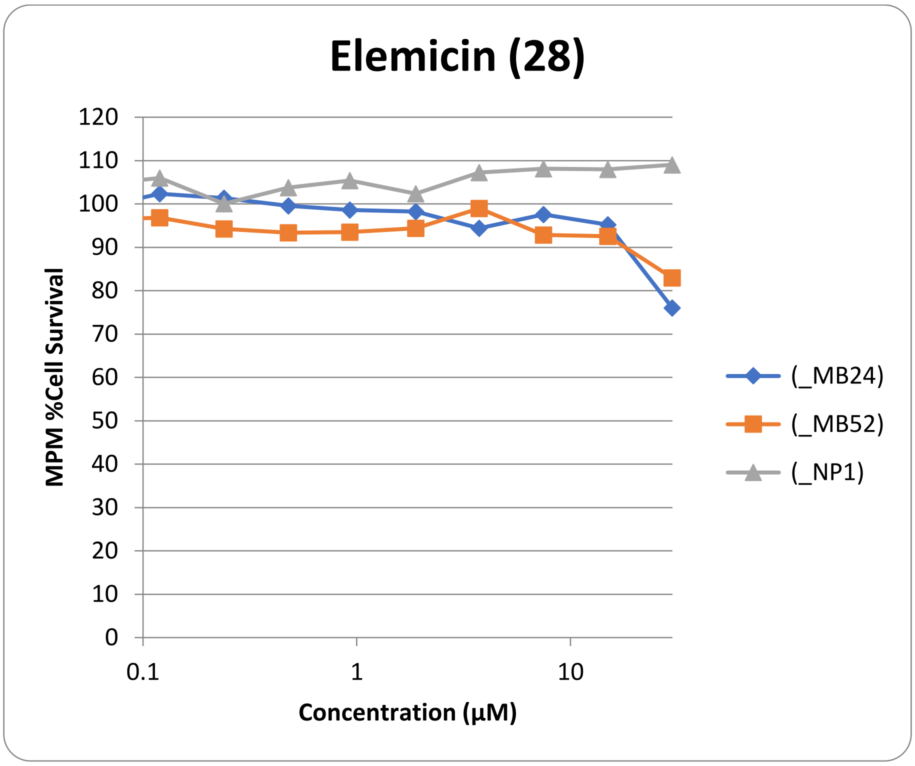

Figure S140: Concentration-dependent cytotoxic effects of elemicin (**28**) on MB24, MB52, and NP1 cell lines.

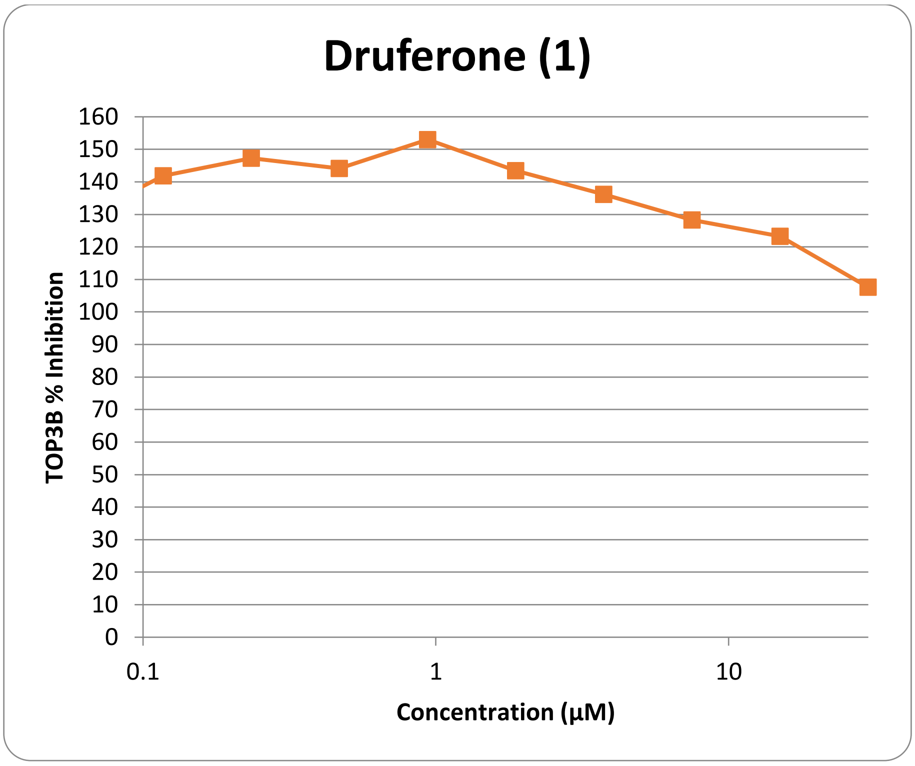

Figure S141: Concentration-dependent cytotoxic effects of druferone (**1**) on HCT-116 cell line.

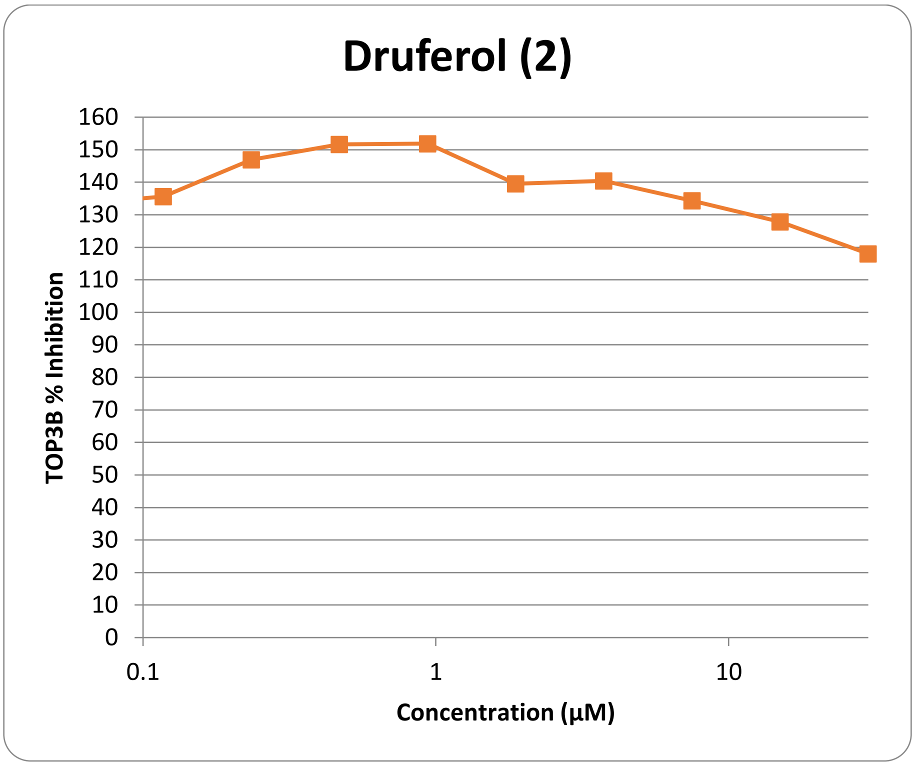

Figure S142: Concentration-dependent cytotoxic effects of druferol (**2**) on HCT-116 cell line.

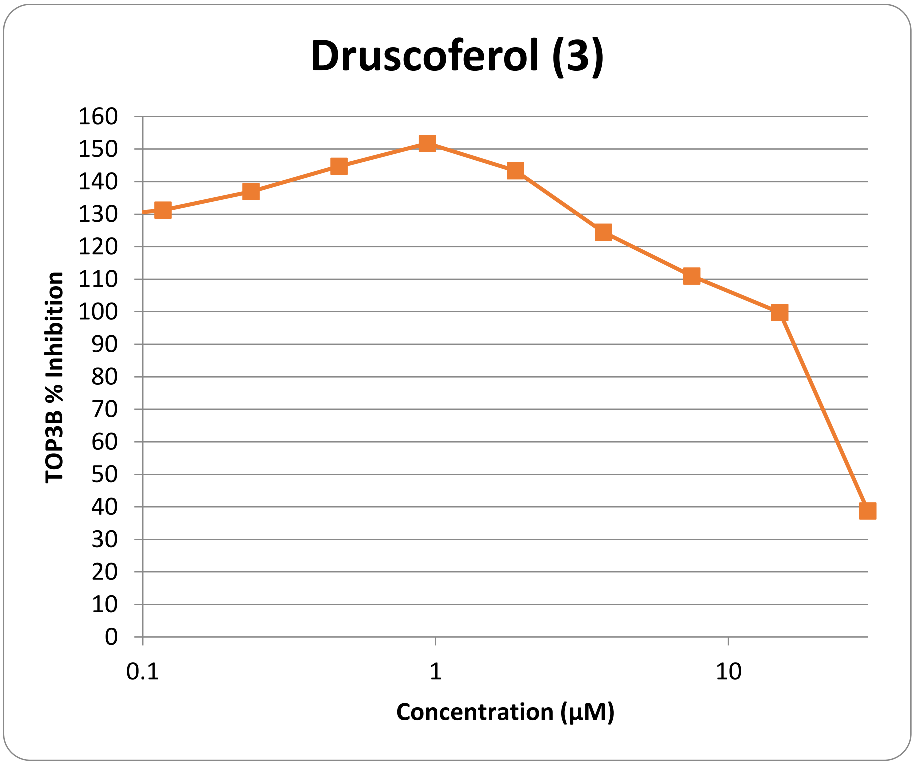

Figure S143: Concentration-dependent cytotoxic effects of druscoferol (**3**) on HCT-116 cell line.

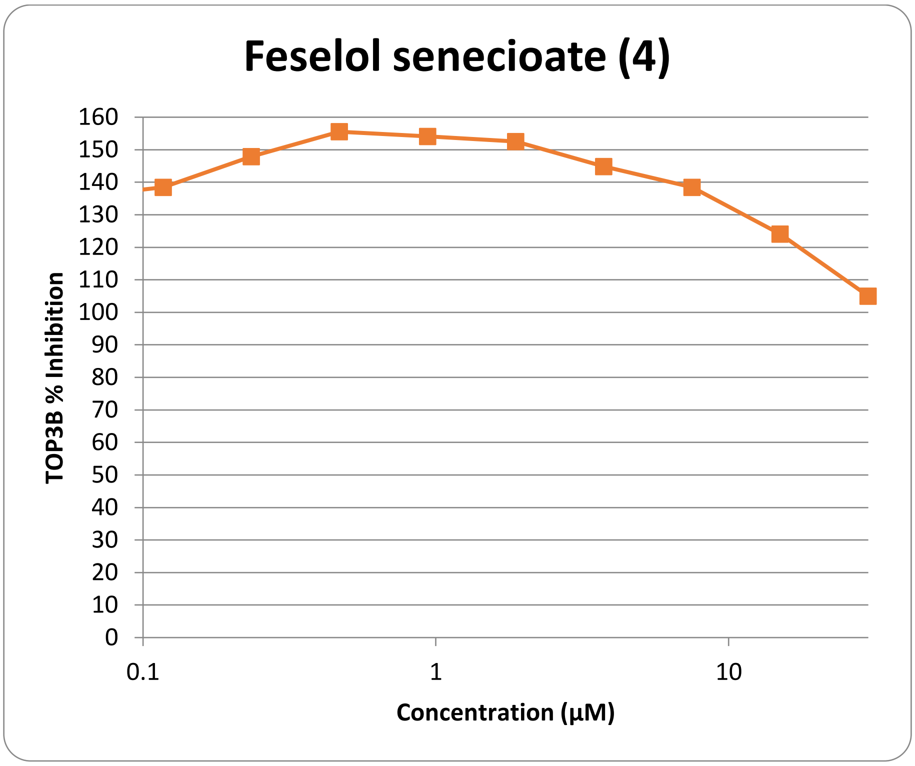

Figure S144: Concentration-dependent cytotoxic effects of feselol senecioate (**4**) on HCT-116 cell line.

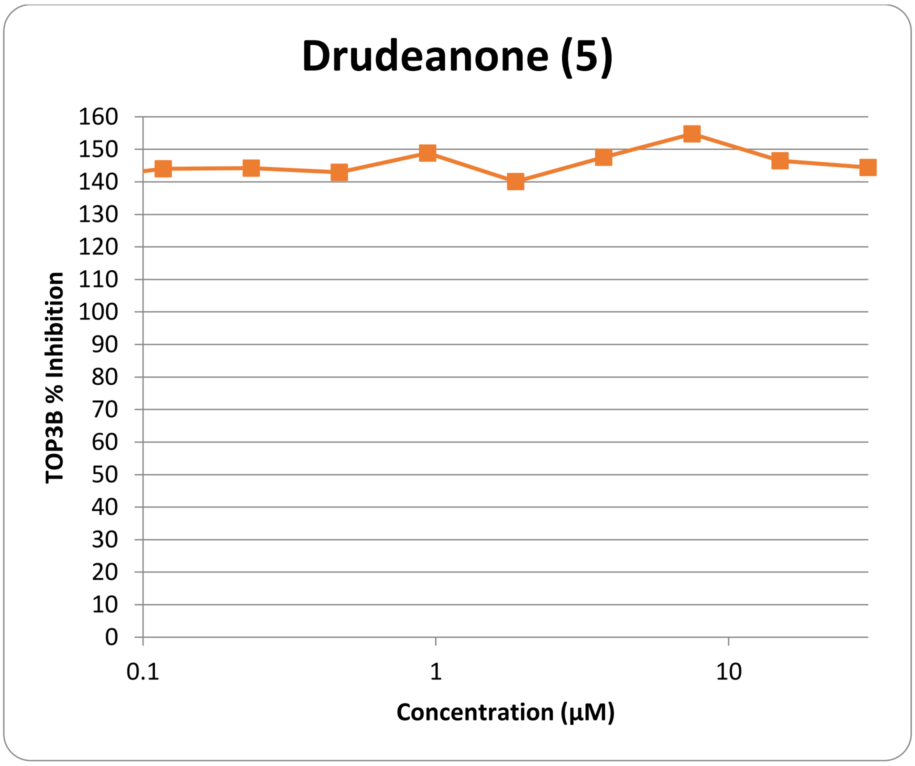

Figure S145: Concentration-dependent cytotoxic effects of drudeanone (**5**) on HCT-116 cell line.

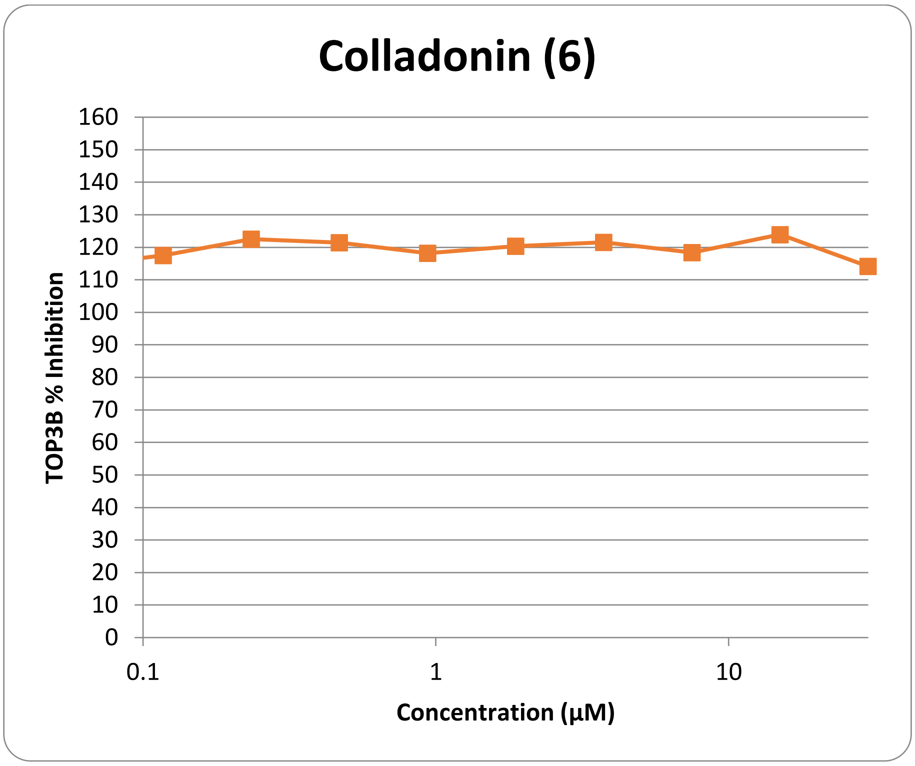

Figure S146: Concentration-dependent cytotoxic effects of colladonin (**6**) on HCT-116 cell line.

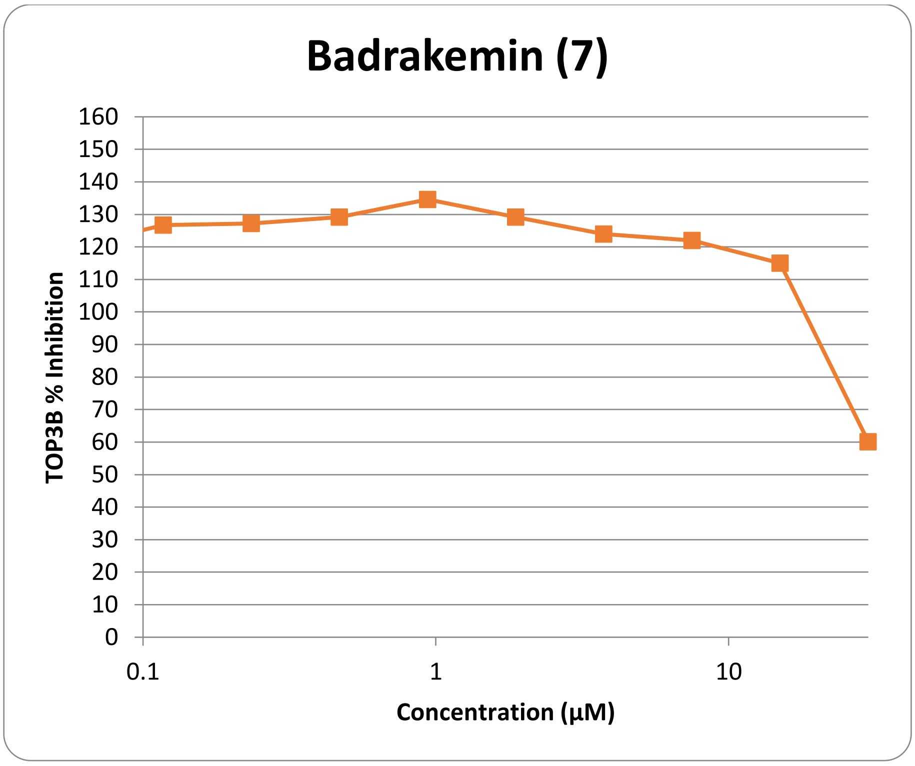

Figure S147: Concentration-dependent cytotoxic effects of badrakemin (**7**) on HCT-116 cell line.

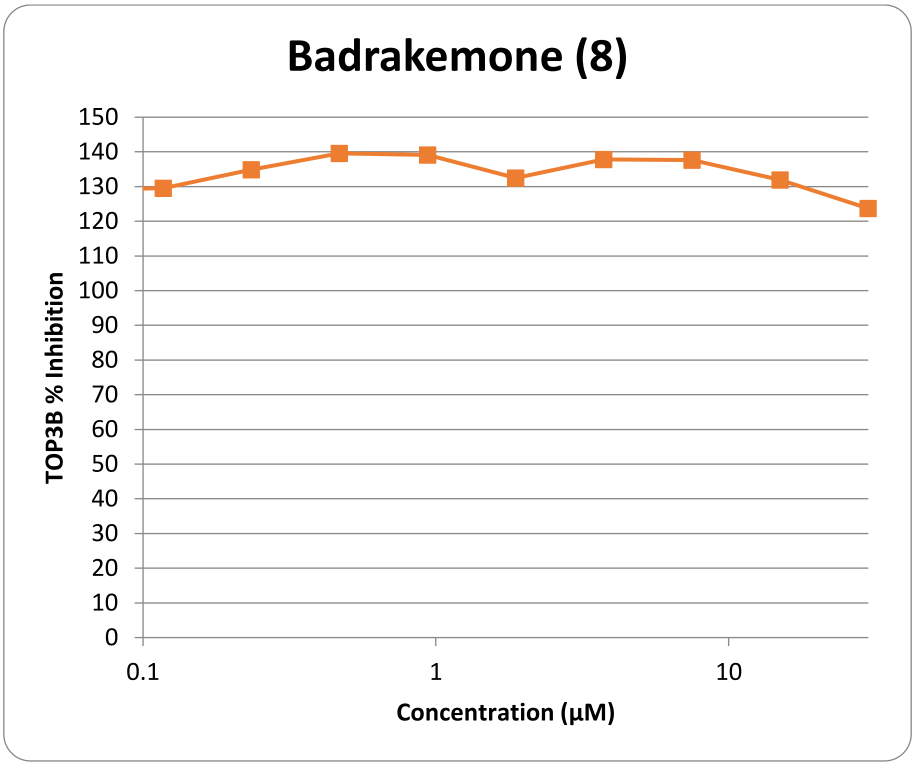

Figure S148: Concentration-dependent cytotoxic effects of badrakemone (**8**) on HCT-116 cell line.

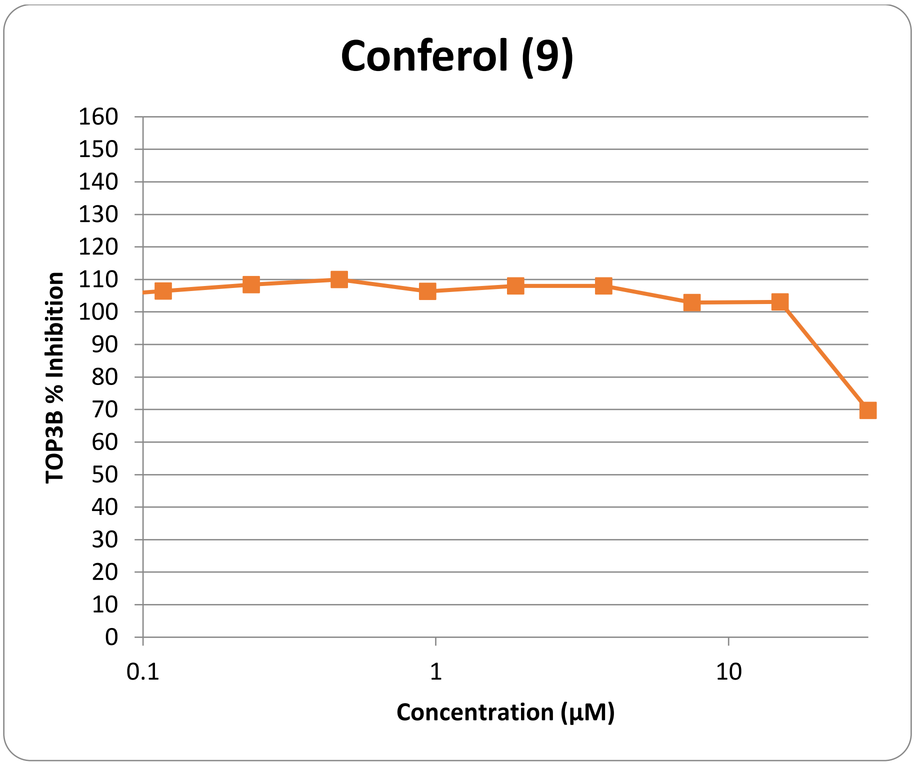

Figure S149: Concentration-dependent cytotoxic effects of conferol (**9**) on HCT-116 cell line.

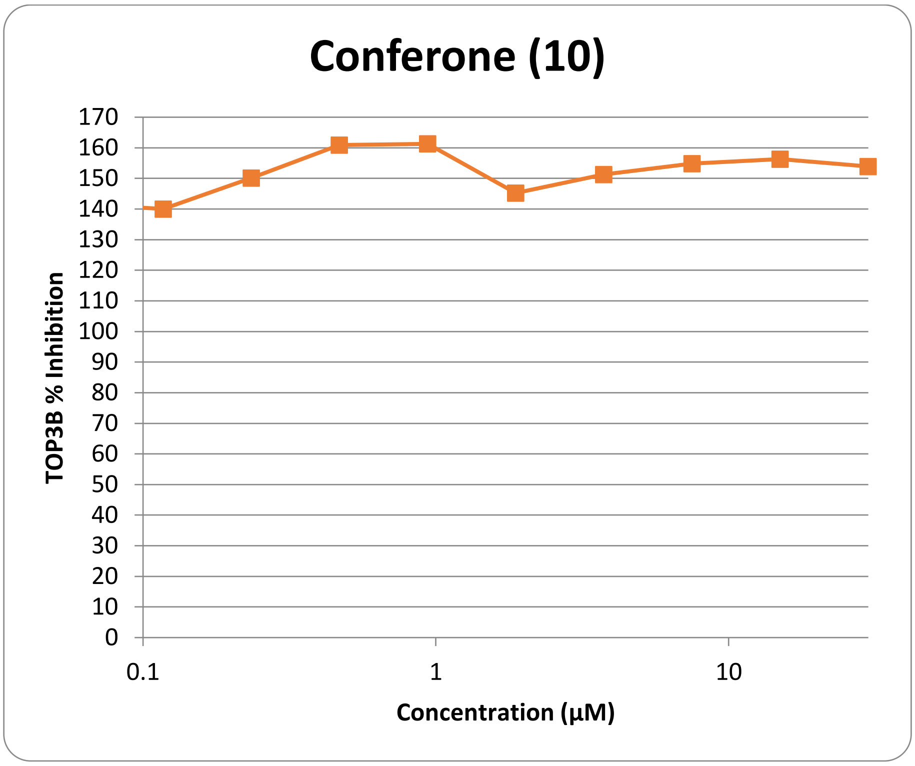

Figure S150: Concentration-dependent cytotoxic effects of conferone (**10**) on HCT-116 cell line.

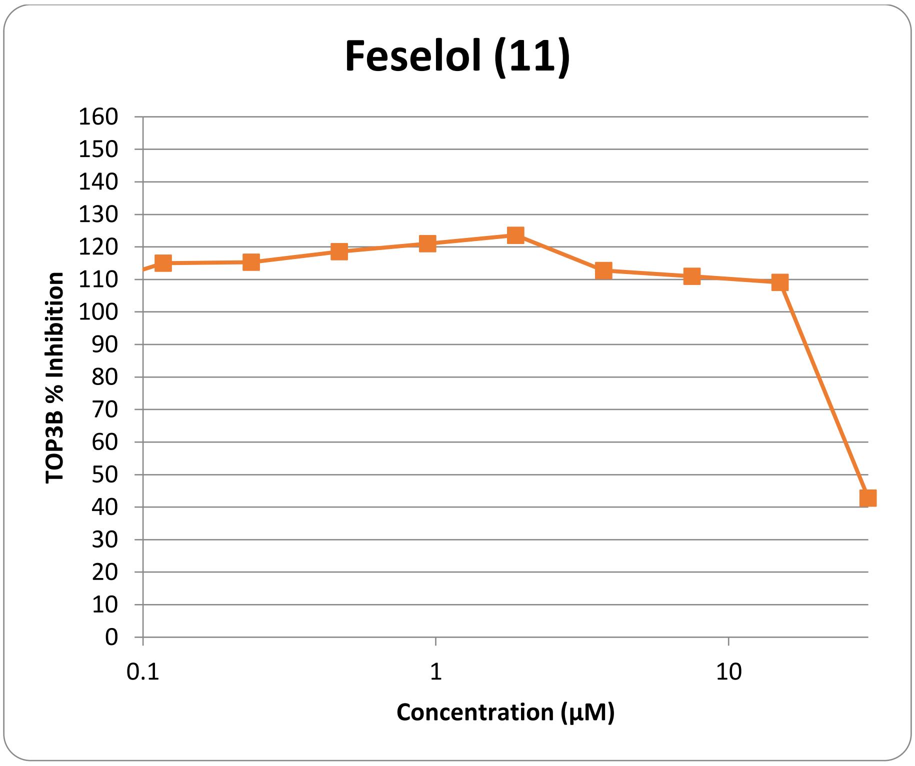

Figure S151: Concentration-dependent cytotoxic effects of feselol (**11**) on HCT-116 cell line.

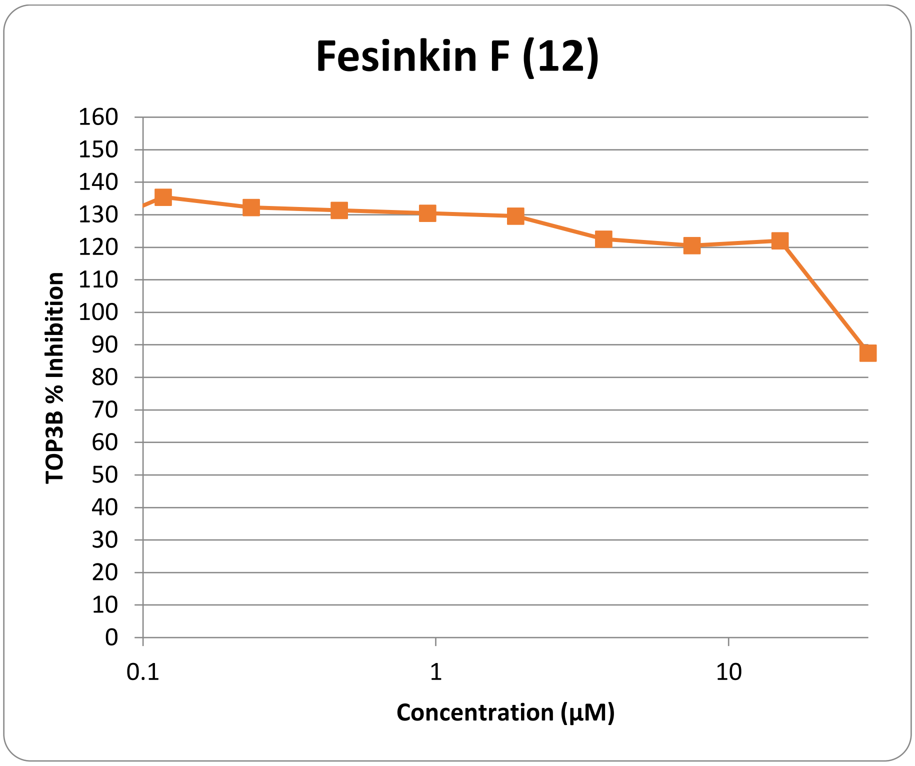

Figure S152: Concentration-dependent cytotoxic effects of fesinkin F (**12**) on HCT-116 cell line.

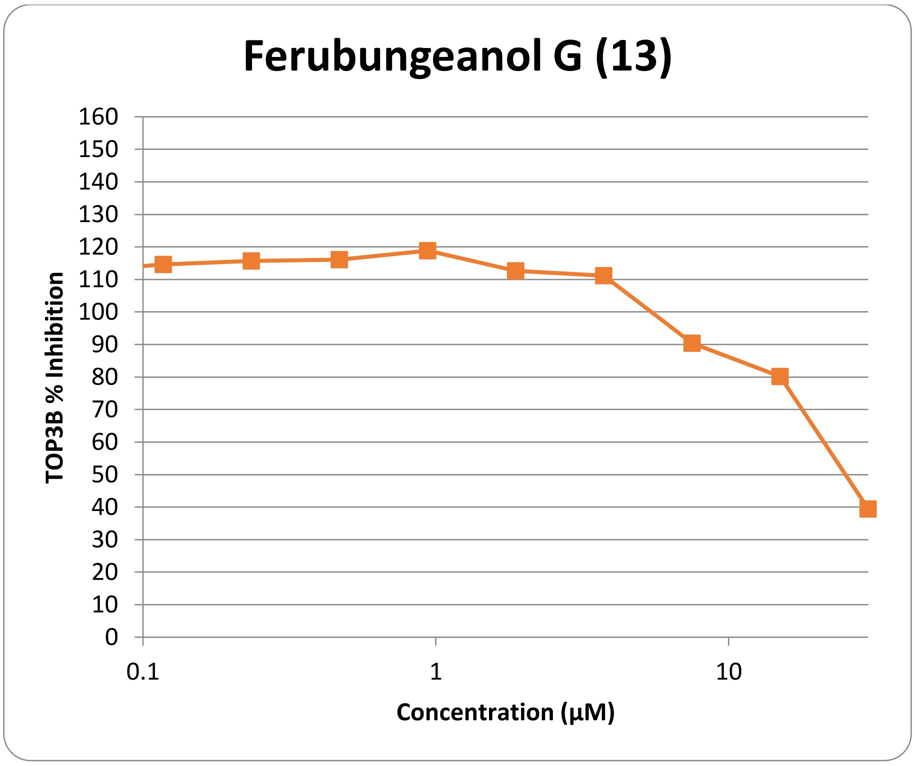

Figure S153: Concentration-dependent cytotoxic effects of ferubungeanol G (**13**) on HCT-116 cell line.

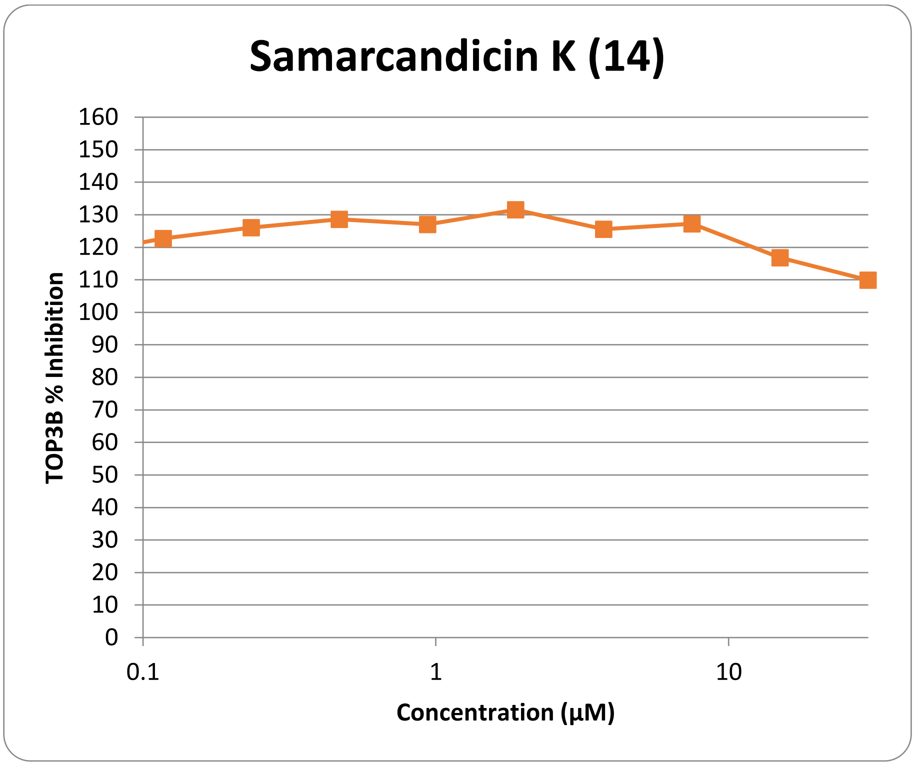

Figure S154: Concentration-dependent cytotoxic effects of samarcandicin K (**14**) on HCT-116 cell line.

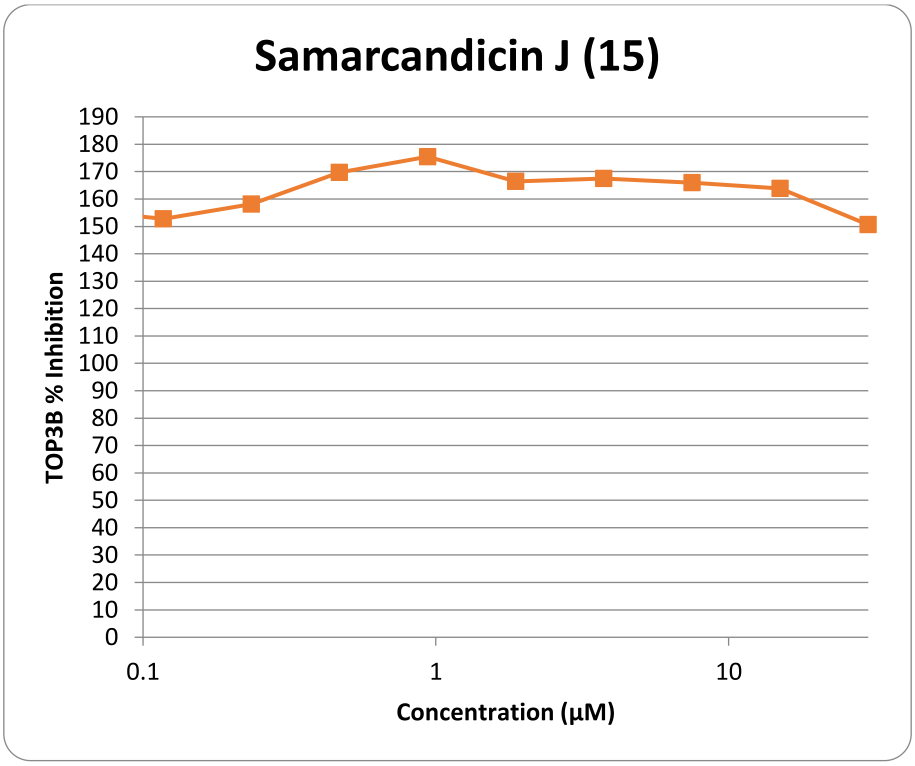

Figure S155: Concentration-dependent cytotoxic effects of samarcandicin J (**15**) on HCT-116 cell line.

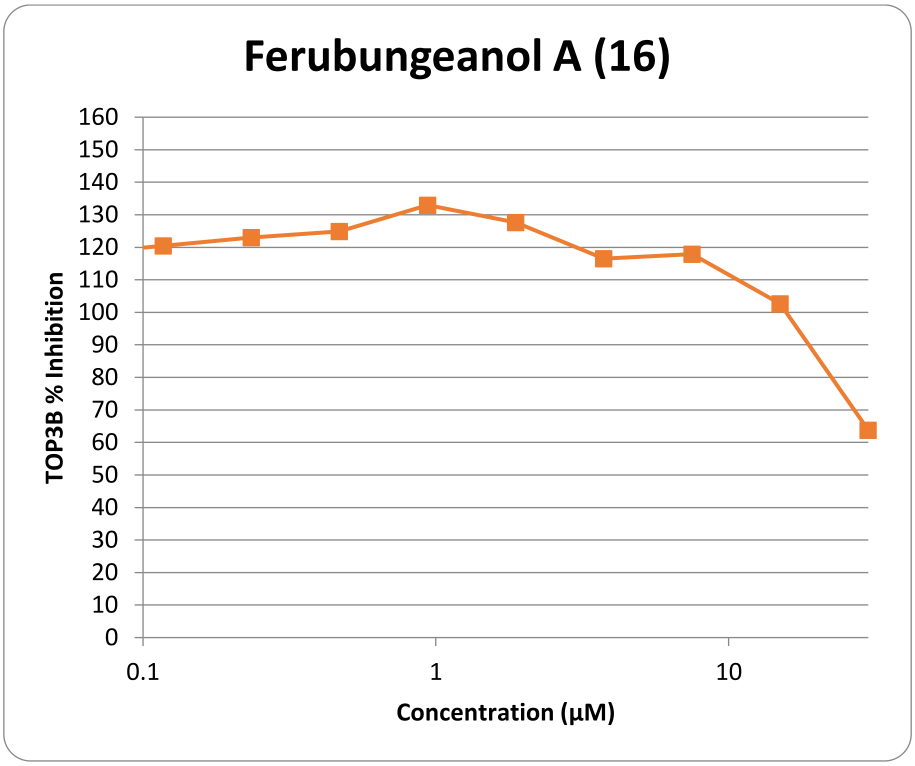

Figure S156: Concentration-dependent cytotoxic effects of ferubungeanol A (**16**) on HCT-116 cell line.

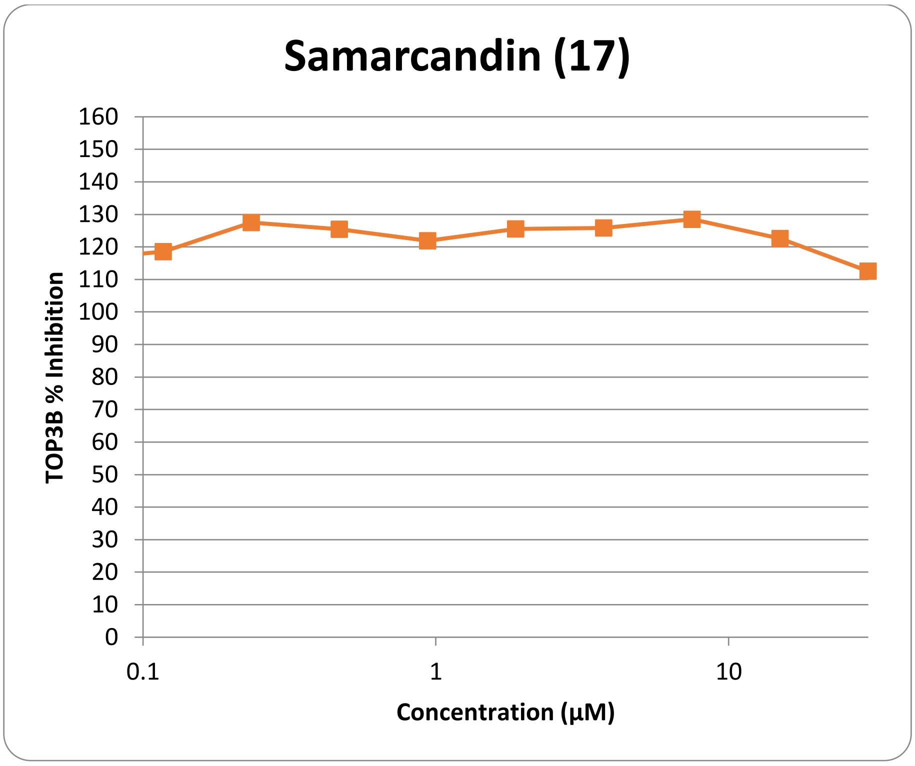

Figure S157: Concentration-dependent cytotoxic effects of samarcandin (**17**) on HCT-116 cell line.

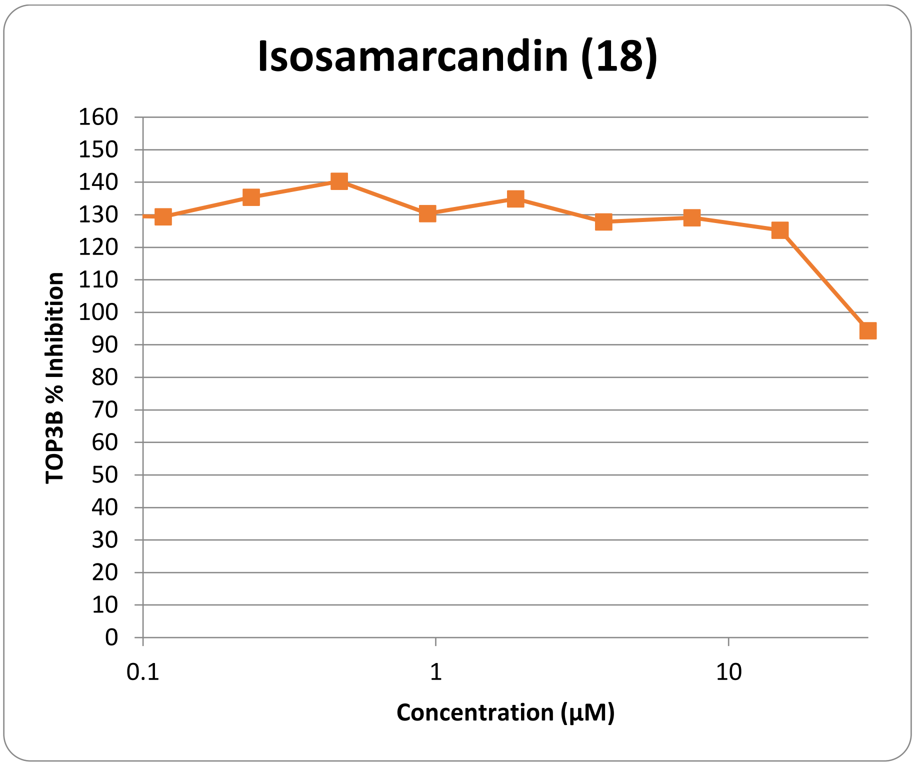

Figure S158: Concentration-dependent cytotoxic effects of isosamarcandin (**18**) on HCT-116 cell line.

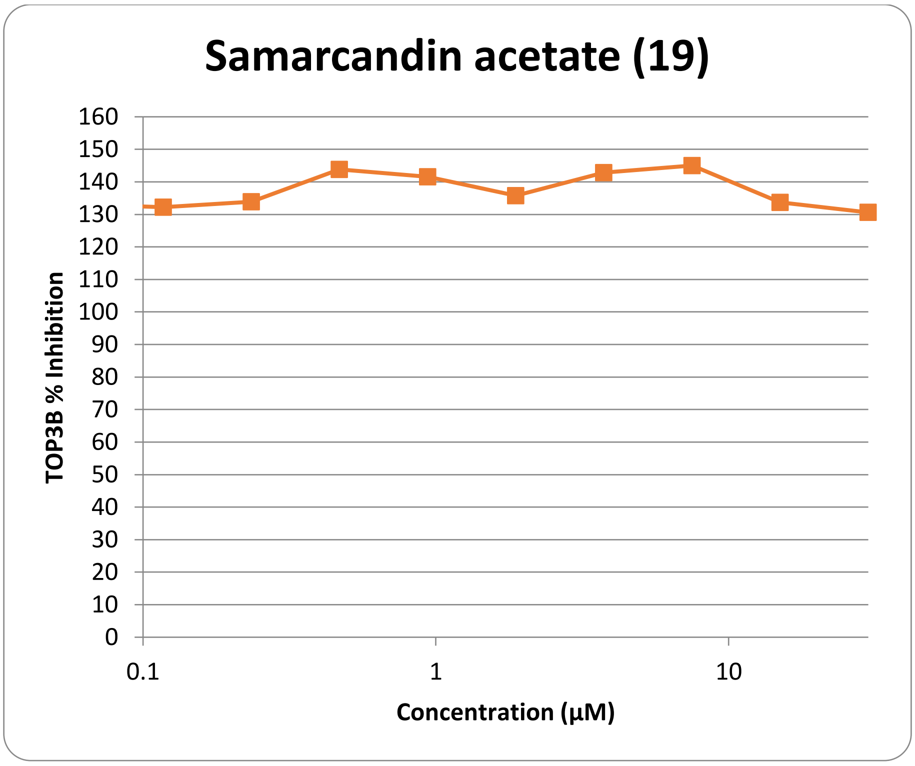

Figure S159: Concentration-dependent cytotoxic effects of samarcandin acetate (**19**) on HCT-116 cell line.

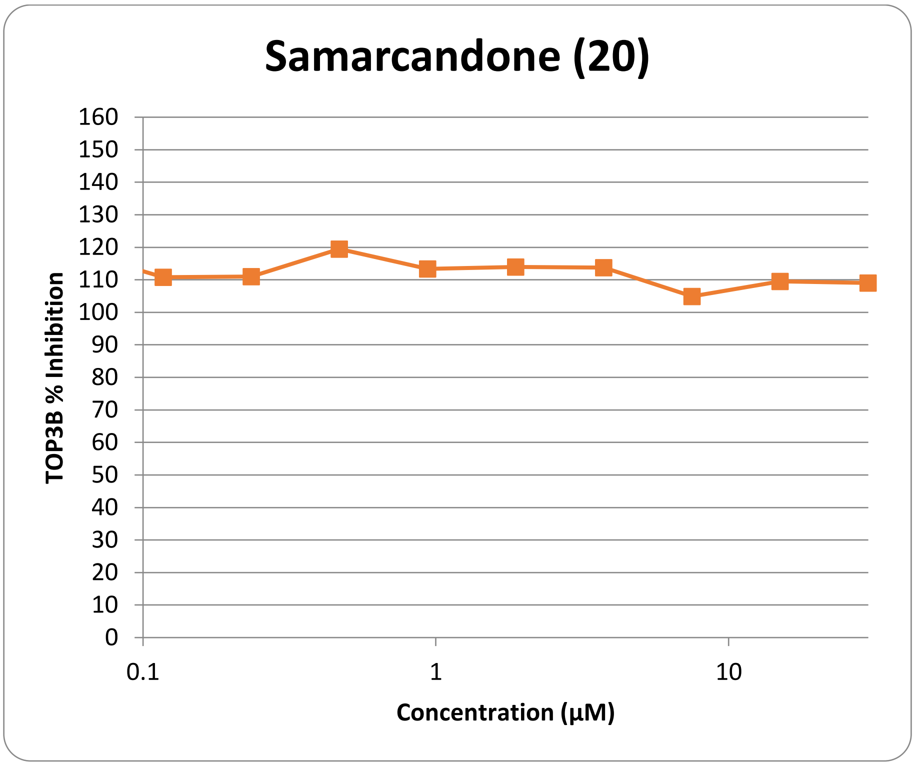

Figure S160: Concentration-dependent cytotoxic effects of samarcandone (**20**) on HCT-116 cell line.

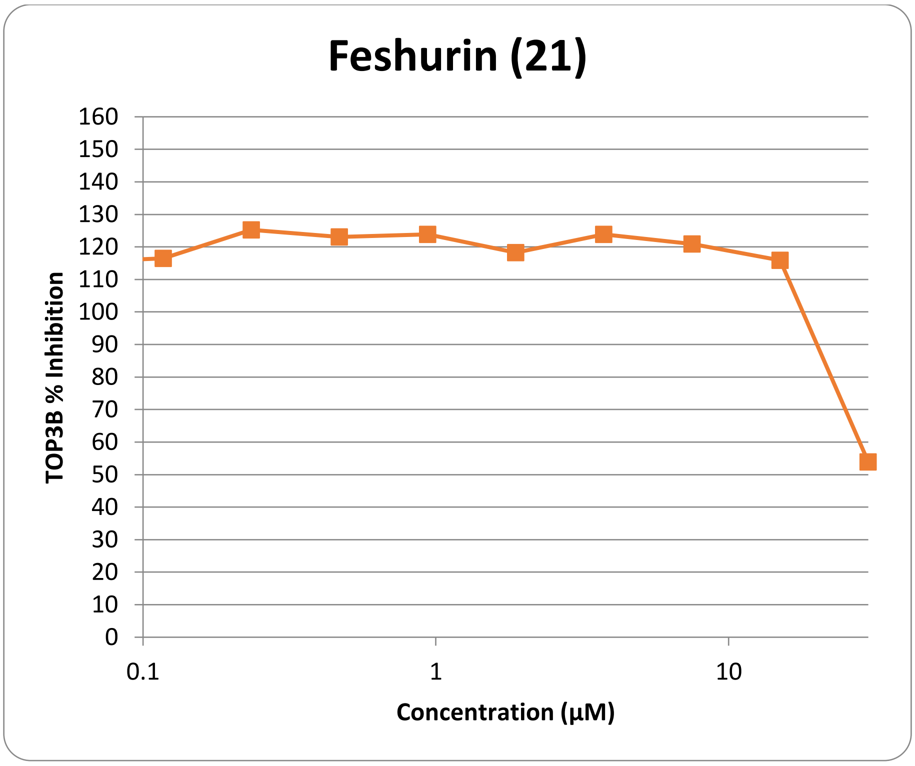

Figure S161: Concentration-dependent cytotoxic effects of feshurin (**21**) on HCT-116 cell line.

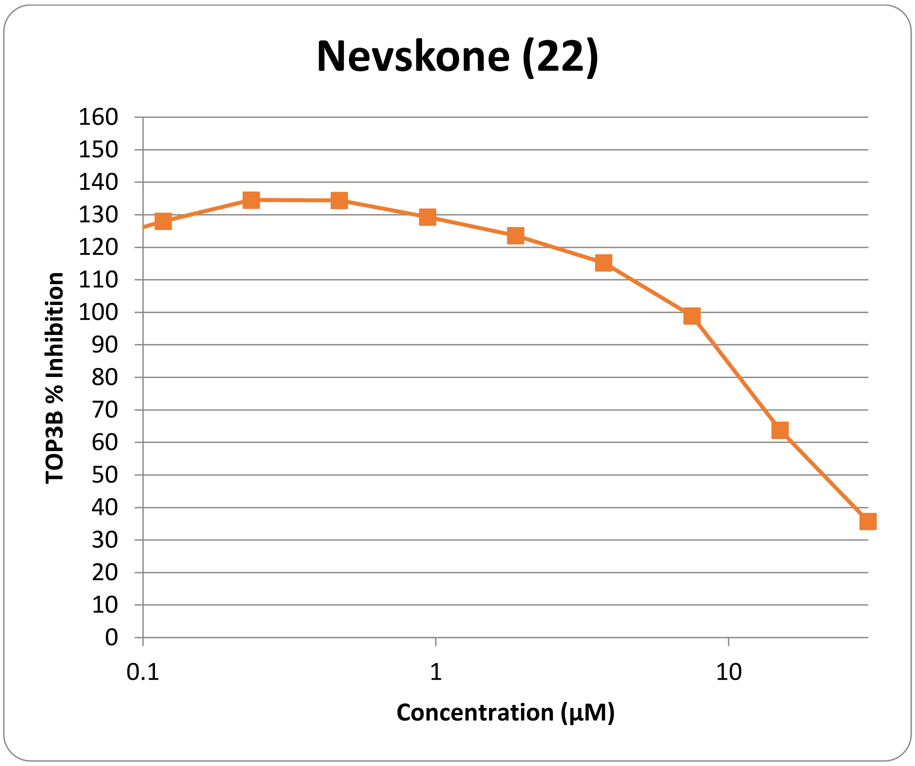

Figure S162: Concentration-dependent cytotoxic effects of nevskone (**22**) on HCT-116 cell line.

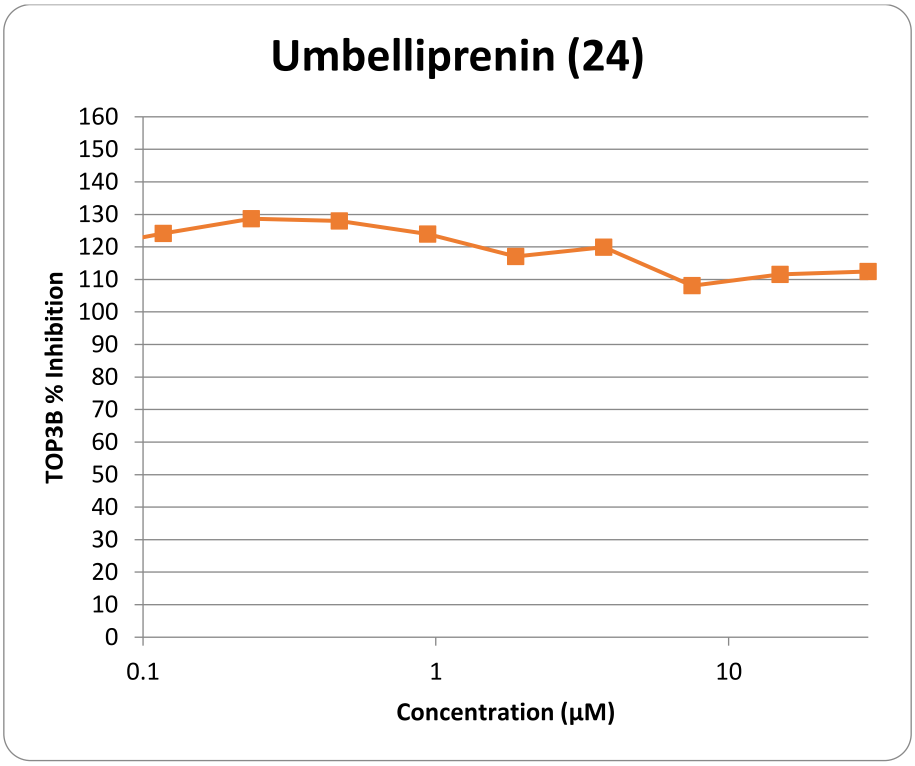

Figure S163: Concentration-dependent cytotoxic effects of umbelliprenin (**24**) on HCT-116 cell line.

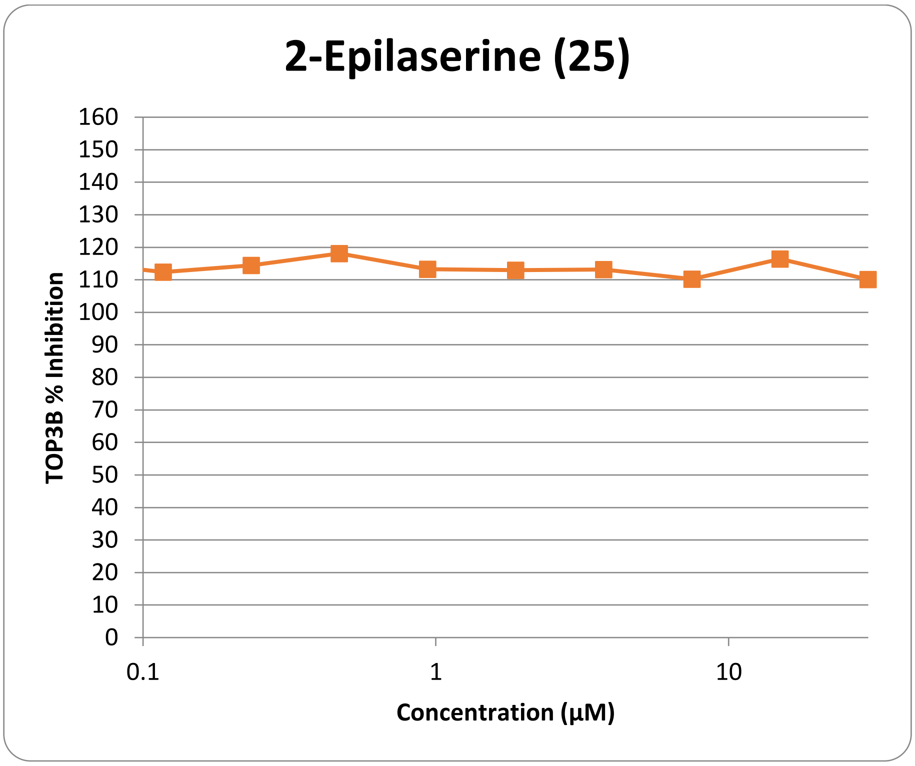

Figure S164: Concentration-dependent cytotoxic effects of 2-epilaserine (**25**) on HCT-116 cell line.

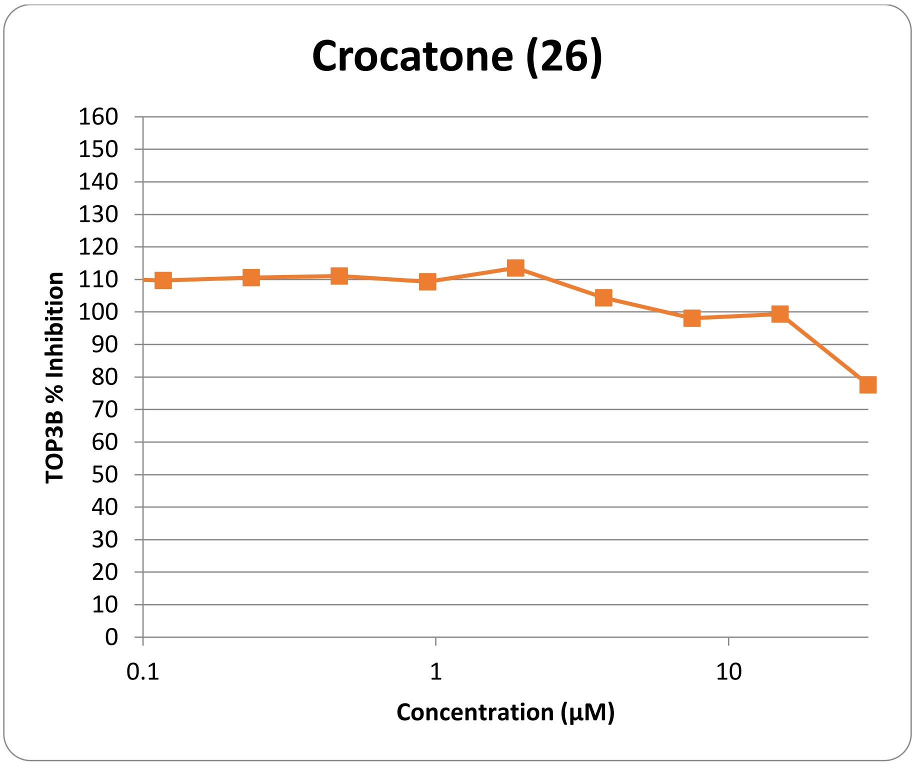

Figure S165: Concentration-dependent cytotoxic effects of crocaton (**26**) on HCT-116 cell line.

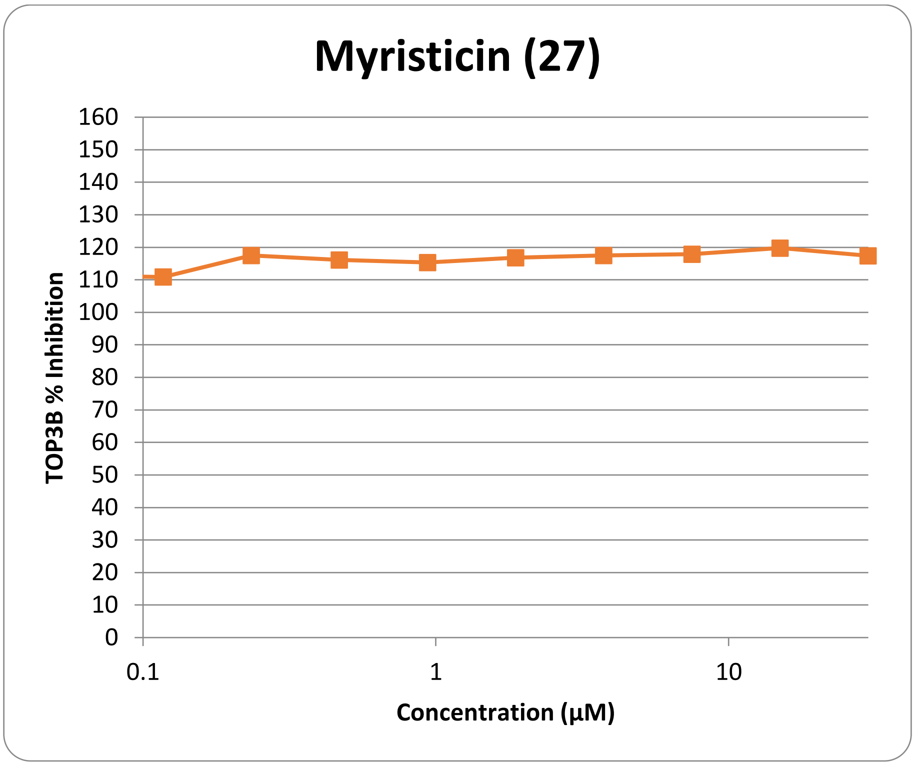

Figure S166: Concentration-dependent cytotoxic effects of myristicin (**27**) on HCT-116 cell line.

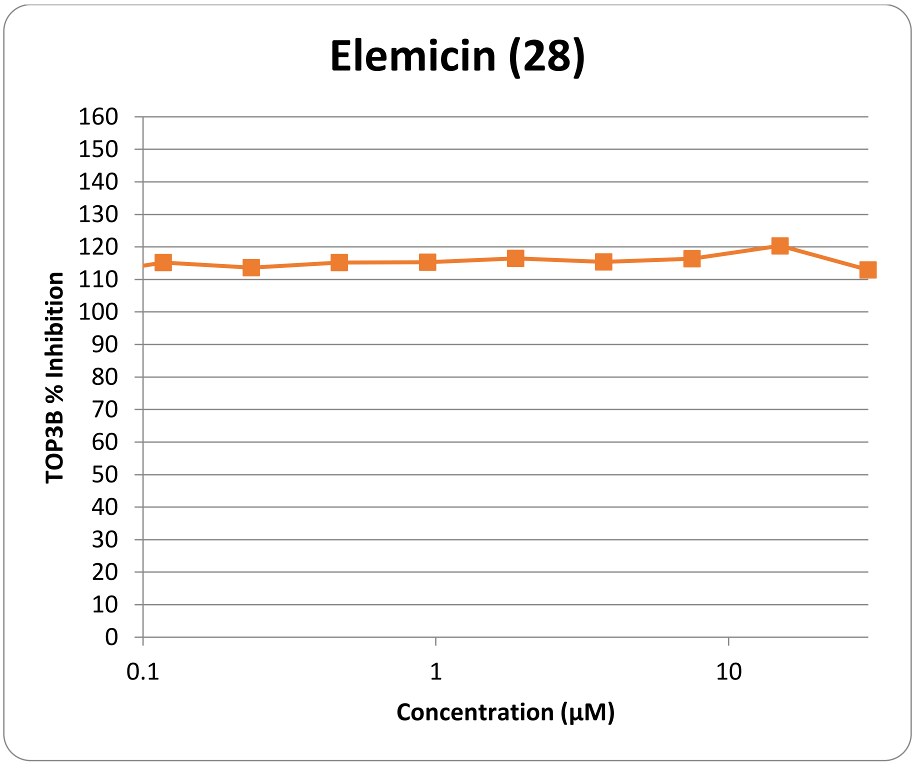

Figure S167: Concentration-dependent cytotoxic effects of elemicin (**28**) on HCT-116 cell line.

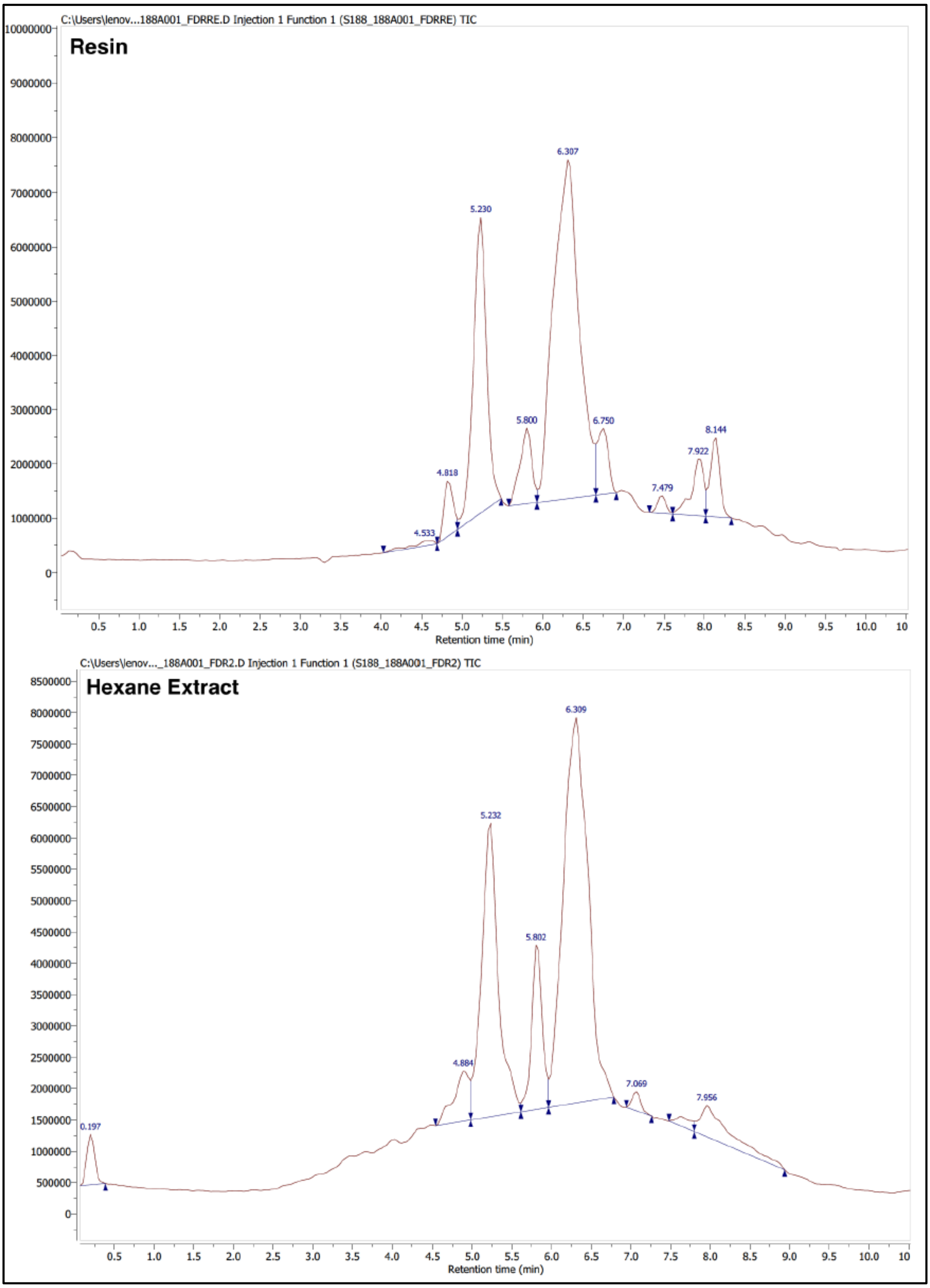

Figure S168: HPLC profiles of the resin and the hexane extract of *F. drudeana*.

Synthesis and chiral separation of 1-(7-methoxybenzo[d][1,3]dioxol-5-yl)-1-oxopropan-2-yl 3-methylbut-2-enoate (5)

S1.1. Starting material synthesis

The synthesis of the starting materials followed the previously reported procedures [1] with modifications.

Step-1:

In an oven-dried two neck flask, the commercial 4-methoxybenzo[d][1,3]dioxole (**1**) (100 mg, 1 equiv.) was dissolved in 2 mL dry THF at 0 °C followed by the dropwise addition of a 3M solution of ethylmagnesium bromide in diethyl ether (0.6 mL). The reaction mixture was allowed to warm up to room temperature and stirred for 4 h. After the reaction was completed, it was quenched by dropwise addition of saturated ammonium chloride solution then the crude product was extracted with EtOAc and washed with water. Then, the organic layer was evaporated under reduced pressure. The crude product was subjected to the next step without further purification.

Step-2:

In a round-bottom flask, the crude benzyl alcohol **2** (117 mg, 1 equiv.) was taken in 10 mL of DCM followed by the addition of PCC (180 mg, 1.5 equiv.). The reaction mixture was stirred at room temperature for 4 h. After the reaction was completed, the reaction mixture was filtered by a Celite bed. The crude product was subjected to the next step without further purification.

Step-3:

The above synthesized propiophenone derivative **3** (34.7 mg, 1 equiv.) was taken in 5 mL of DMSO and I_2 (12.7 mg, 0.3 equiv.) was added to the solution. The reaction was stirred for 16 h at 50 °C under air. After the completion of the reaction, the reaction mixture was cooled down to room temperature, quenched with $Na_2S_2O_3$ solution, extracted with EtOAc, and washed with water. The organic layer was evaporated by rotavapor, and the crude product was further purified by prep HPLC using a Gemini 5 µm NX-18 column (250 × 21.2 mm) eluted with MeCN/H_2O with 0.1% TFA (30% to 100% gradient MeCN) at a flow rate of 10 mL/min to afford the α-chloroketone derivative **4** (21.1 mg, 52% yield).

2-Chloro-1-(7-methoxybenzo[d][1,3]dioxol-5-yl)propan-1-one (4): White power; ^{1}H NMR data (600 MHz, DMSO-d_6): δ_H (ppm) 7.36 (d, J = 1.6 Hz, 1H), 7.31 (d, J = 1.6 Hz, 1H), 6.15 (s, 2H), 5.77 (q, J = 6.6 Hz, 1H), 3.89 (s, 3H), 1.58 (d, J = 6.6 Hz, 3H); ^{13}C NMR data (150 MHz, DMSO-d_6): δ_C (ppm) 192.1, 148.8, 143.2, 140.0, 128.4, 109.9, 103.1, 102.7, 56.5, 53.4, 20.2; ESIMS m/z 243.0, $[M + H]^+$.

S1.2. Esterification

To a solution of the α-chloroketone derivative **4** (21.0 mg, 1 equiv.) in dry MeCN (2 mL), 3-methylcrotonic acid (26.0 mg, 3 equiv.) and Et_3N (72 µL, 6 equiv.) were added. The reaction mixture was stirred at 80 °C for 16 h. After the completion of the reaction, the reaction mixture was cooled down to room temperature and dried down over N_2 flow. The crude product was further purified by prep HPLC using a Gemini 5 µm NX-18 column (250 × 21.2 mm) eluted with MeCN/H_2O with 0.1% TFA (40% to 100% gradient MeCN) at a flow rate of 10 mL/min to afford the target ester **5** (20.2 mg, 76% yield).

1-(7-methoxybenzo[d][1,3]dioxol-5-yl)-1-oxopropan-2-yl 3-methylbut-2-enoate (5): Colorless film; ^{1}H NMR data (600 MHz, DMSO-d_6): δ_H (ppm) 7.30 (s, 2H), 6.14 (s, 2H), 6.00 (q, J = 7.0 Hz, 1H), 5.79

(s, 1H), 3.89 (s, 3H), 2.07 (s, 3H), 1.90 (s, 3H), 1.38 (d, J = 7.0 Hz, 3H); ^{13}C NMR data (150 MHz, DMSO-d_6): δ_C (ppm) 195.0, 165.0, 158.4, 148.9, 143.3, 139.9, 128.4, 114.9, 109.1, 102.6 (2C), 70.5, 56.5, 27.0, 19.9, 17.3.

S1.3. Chiral HPLC

The ester **5** (20 mg) was separated by semiprep HPLC using a Lux cellulose-2 column (250 × 10.0 mm) eluted with 40% MeCN/ H_2O with 0.1% TFA at a flow rate of 4 mL/min to afford the pure enantiomers **5a** (9.1 mg, 46% yield) and **5b** (8.5 mg, 43% yield).

(*S*)-1-(7-methoxybenzo[d][1,3]dioxol-5-yl)-1-oxopropan-2-yl 3-methylbut-2-enoate (5a): Colorless film; $[\alpha]_D^{22}$ +30 (c 0.04, MeOH); ECD (c 0.82 mM, MeCN) λmax (Δε) 207 (-0.45), 232 (-0.2), 289 (0.4) nm; HRESIMS m/z 307.1182, [M + H]+ (calcd. for $C_{16}H_{19}O_6$, m/z 307.1176).

(*R*)-1-(7-methoxybenzo[d][1,3]dioxol-5-yl)-1-oxopropan-2-yl 3-methylbut-2-enoate (5b): Colorless film; $[\alpha]_D^{22}$ −40 (c 0.05, MeOH); ECD (c 0.82 mM, MeCN) λmax (Δε) 205 (0.45), 232 (0.2), 289 (-0.4) nm; HRESIMS m/z 307.1181, [M + H]+ (calcd. for $C_{16}H_{19}O_6$, m/z 307.1176).

Computational Details

Conformational searches were performed in an energy window of 3 kcal/mol by ComputeVOA using the GMMX mechanic model for **(*S*)-5**. The low-energy conformers were optimized with Gaussian 16 using DFT/B3LYP/DGDZVP in MeCN for **(*S*)-5**. The optimized conformers with Boltzmann distributions (%) greater than 1% were further submitted to the ECD calculation to generate the simulated ECD spectra using TDDFT/B3LYP/DGDZVP in gas phase for **(*S*)-5**. The simulated ECD spectra were further averaged based on the Boltzmann distributions to afford the theoretical ECD spectra for **(*S*)-5**.

Table S1. DFT/B3LYP/DGDZVP optimized conformers of model structure **5a** submitted for ECD simulations at TDDFT/B3LYP/DGDZVP in gas phase.

No	Conformer name	Gibbs free energy	Boltzmann population at 25 ºC (%)
1	21	-1072.0162719	21.5928688
2	16	-1072.0162684	21.5129219
3	24	-1072.0162368	20.8043846
4	15	-1072.0161413	18.8017859
5	28	-1072.0160621	17.2880388

Cartesian coordinates of conformers shown in **Table S1.**

Conformer 1

C 3.747324 0.802676 -1.416872
C 3.476586 -0.251853 -0.553451
C 2.198966 -0.733024 -0.334176
C 1.154510 -0.089333 -1.041200
C 1.427556 0.981078 -1.920395
C 2.732857 1.448450 -2.129739
O 5.092306 1.051969 -1.465515
C 5.667631 0.237279 -0.413702
O 4.661468 -0.718442 -0.022526
O 3.105133 2.464914 -2.957903
C 2.070396 3.134784 -3.702183
C -0.268739 -0.500415 -0.898533

C	-0.608708	-1.757698	-0.076528
O	-1.994422	-1.726104	0.327524
O	-1.177817	0.112559	-1.461333
C	-0.432539	-3.024184	-0.918123
C	-2.332055	-0.825598	1.296515
C	-3.766630	-0.943715	1.605807
C	-4.481086	-0.187517	2.475237
O	-1.501111	-0.070064	1.795148
C	-5.951268	-0.466373	2.661839
C	-3.944579	0.944826	3.309444
H	2.035478	-1.558039	0.349014
H	0.592083	1.439973	-2.434895
H	5.914252	0.879973	0.438343
H	6.538872	-0.289288	-0.803698
H	1.347979	3.605131	-3.027414
H	2.581024	3.899538	-4.287577
H	1.557822	2.436186	-4.371282
H	0.006655	-1.804677	0.822804
H	0.607335	-3.129327	-1.236554
H	-1.069729	-2.986856	-1.806688
H	-0.703240	-3.899538	-0.321170
H	-4.277221	-1.730688	1.057027
H	-6.294330	-1.314016	2.063869
H	-6.164146	-0.669110	3.718913
H	-6.538873	0.419332	2.389853
H	-4.581306	1.828111	3.178864
H	-4.008121	0.673032	4.371283
H	-2.914158	1.200592	3.073131

Conformer 2

C	-4.274526	-2.081108	-0.713643
C	-3.322718	-2.474477	-1.646518
C	-1.967101	-2.269881	-1.467481
C	-1.581541	-1.626042	-0.266642
C	-2.546649	-1.227284	0.683473
C	-3.916237	-1.449837	0.481153
O	-5.530588	-2.429604	-1.130789
C	-5.364476	-2.886172	-2.496649
O	-3.953774	-3.101818	-2.701024
O	-4.925353	-1.112935	1.333073
C	-4.570381	-0.461663	2.567342

C	-0.157376	-1.337989	0.054558
C	0.946677	-1.887032	-0.868545
O	2.169004	-1.143836	-0.674449
O	0.162932	-0.711222	1.066243
C	1.268548	-3.344603	-0.528561
C	2.198661	0.130565	-1.163499
C	3.516329	0.732112	-0.900353
C	3.912361	1.995337	-1.192789
O	1.231264	0.626145	-1.736432
C	5.313197	2.429905	-0.842634
C	3.072012	3.053981	-1.855466
H	-1.260892	-2.592509	-2.223356
H	-2.198381	-0.736454	1.584058
H	-5.899983	-3.826686	-2.627906
H	-5.718650	-2.104939	-3.177742
H	-4.065819	0.490360	2.373735
H	-5.513517	-0.281780	3.083440
H	-3.930501	-1.106958	3.177742
H	0.652962	-1.791752	-1.914699
H	0.389465	-3.975213	-0.681072
H	1.588369	-3.434329	0.513832
H	2.070315	-3.702600	-1.180366
H	4.221823	0.063797	-0.413660
H	5.899983	1.624725	-0.394211
H	5.831313	2.789032	-1.740577
H	5.279843	3.274936	-0.143545
H	2.036082	2.753418	-1.995277
H	3.109493	3.975213	-1.261626
H	3.505538	3.299557	-2.833755

Conformer 3

C	-3.375225	2.587523	-0.200595
C	-3.089819	1.752193	0.872483
C	-1.817188	1.281837	1.139402
C	-0.795324	1.693291	0.249741
C	-1.082679	2.543577	-0.840109
C	-2.380830	3.013334	-1.086136
O	-4.702625	2.921412	-0.211072
C	-5.314159	2.077494	0.796273
O	-4.244751	1.530508	1.593867
O	-2.764119	3.841200	-2.098730

C	-1.748674	4.297834	-3.011748
C	0.618257	1.256242	0.411192
C	1.026361	0.491928	1.684226
O	2.264131	-0.216866	1.461973
O	1.479758	1.533977	-0.425120
C	1.282789	1.459351	2.842768
C	2.206262	-1.319723	0.659178
C	3.543388	-1.927536	0.555072
C	3.884679	-3.044213	-0.133924
O	1.154693	-1.688203	0.141284
C	5.319021	-3.510215	-0.121377
C	2.951270	-3.904233	-0.943247
H	-1.642128	0.630586	1.987549
H	-0.264301	2.826965	-1.490827
H	-5.965005	2.684489	1.425880
H	-5.853677	1.264332	0.298712
H	-2.265963	4.937835	-3.726633
H	-0.982229	4.874783	-2.484330
H	-1.287771	3.454116	-3.535325
H	0.260860	-0.236163	1.955085
H	0.372422	2.012263	3.086377
H	2.068247	2.174273	2.580307
H	1.593932	0.895585	3.726633
H	4.316960	-1.396311	1.103305
H	5.965005	-2.856058	0.468998
H	5.380066	-4.527789	0.284324
H	5.707023	-3.559502	-1.146463
H	3.001738	-4.937835	-0.578601
H	1.919198	-3.562469	-0.914329
H	3.293906	-3.928973	-1.985582

Conformer 4

C	-2.368336	-3.502795	-0.634269
C	-2.054922	-2.557800	-1.602242
C	-0.931009	-1.755121	-1.530304
C	-0.094006	-1.942832	-0.403718
C	-0.410796	-2.903470	0.581279
C	-1.557185	-3.704848	0.486458
O	-3.527853	-4.154608	-0.949793
C	-3.987962	-3.569356	-2.194230
O	-3.019495	-2.573018	-2.585995

O	-1.955291	-4.657861	1.376399
C	-1.132842	-4.874505	2.537678
C	1.148539	-1.150148	-0.199338
C	1.623754	-0.197471	-1.312318
O	2.517320	0.796393	-0.766355
O	1.833561	-1.272078	0.818081
C	2.419014	-0.957209	-2.376908
C	1.955489	1.760601	0.020855
C	2.996072	2.695944	0.478592
C	2.820539	3.811118	1.229480
O	0.751042	1.776341	0.263035
C	4.019270	4.645941	1.604155
C	1.506648	4.327174	1.752405
H	-0.729841	-1.027257	-2.307339
H	0.263433	-3.007429	1.422676
H	-4.038192	-4.349198	-2.957844
H	-4.956357	-3.091114	-2.026612
H	-1.064269	-3.964148	3.141996
H	-1.632206	-5.658734	3.106804
H	-0.130610	-5.207231	2.248791
H	0.775396	0.319373	-1.762469
H	1.790102	-1.711526	-2.855835
H	3.284109	-1.454555	-1.928361
H	2.766157	-0.257264	-3.141996
H	4.001350	2.434752	0.158699
H	4.956357	4.216503	1.242036
H	3.913180	5.658734	1.195641
H	4.076330	4.754214	2.694438
H	1.511920	4.280973	2.849301
H	1.399940	5.386542	1.489499
H	0.648890	3.771353	1.380508

Conformer 5

C	3.597596	-0.912241	-1.213761
C	3.651270	0.057799	-0.216827
C	2.555831	0.812729	0.147255
C	1.348305	0.555914	-0.546669
C	1.289444	-0.425669	-1.559411
C	2.421812	-1.179897	-1.915793
O	4.826615	-1.486824	-1.392522
C	5.644007	-0.984257	-0.306301

O	4.939566	0.134010	0.269667
O	2.477822	-2.145150	-2.877248
C	1.277157	-2.427623	-3.619340
C	0.159121	1.362598	-0.160361
C	-1.129985	1.268252	-0.999280
O	-2.257563	1.736779	-0.228532
O	0.200280	2.148436	0.788271
C	-1.044289	2.171396	-2.232426
C	-2.687455	0.929936	0.785388
C	-3.847541	1.545524	1.451091
C	-4.528200	1.065250	2.520790
O	-2.139262	-0.140791	1.036482
C	-5.698659	1.846670	3.062908
C	-4.231032	-0.214130	3.256032
H	2.605897	1.570972	0.920525
H	0.359065	-0.618999	-2.076015
H	6.601094	-0.646571	-0.704510
H	5.758927	-1.772110	0.445784
H	0.936961	-1.540431	-4.163391
H	1.549446	-3.211284	-4.326428
H	0.484167	-2.787679	-2.955842
H	-1.325759	0.236169	-1.291943
H	-0.223089	1.855382	-2.880123
H	-0.877610	3.211284	-1.936134
H	-1.977993	2.106606	-2.798026
H	-4.162744	2.489011	1.013268
H	-5.895763	2.754893	2.488432
H	-6.601093	1.222687	3.060113
H	-5.513602	2.122646	4.108567
H	-4.119979	-0.001593	4.326427
H	-5.089552	-0.891975	3.164165
H	-3.339574	-0.720317	2.892857

References

1. Altia, M.; Anbarasan, P., Regioselective synthesis of 2,3-disubstituted indoles via Interrupted Heyns rearrangement involving C-C bond cleavage. *Chemistry: An Asian Journal* **2024,** *19* e202400731.
